# Functional status following the COVID-19 lockdown and five-year survival among older adults with pre-existing functional dependence: a cohort study

**DOI:** 10.3389/fragi.2026.1868924

**Published:** 2026-10-22

**Authors:** Vicente Martín Moreno, Asunción García Mateos, Amanda Martín Fernández, Laura Flórez Busto, Beatriz Alonso Serna, Annly Luzmila Vásquez Alay, María del Carmen Cubero Sobrados, Jorge Hurtado Gallar, Juana Marcos Guerra, María Inmaculada Martínez Sanz, Irene Sánchez González, Elena Sánchez Rodríguez, Miriam Fernández Gallardo, Elena Pérez Rico

**Affiliations:** 1 Orcasitas Healthcare Center, Research Institute of the Doce de Octubre Hospital, Madrid, Spain; 2 Orcasitas Healthcare Center, Madrid, Spain; 3 Polibea Concert, Madrid, Spain; 4 UNAD Group Manager, Orcasitas Healthcare Center, Madrid, Spain

**Keywords:** activities of daily living, COVID-19, disability evaluation, functional dependence, functional status, living arrangements, neighborhood disadvantage, resilience

## Abstract

**Introduction:**

Lockdown for the COVID-19 pandemic in Spain (14 March 2020 - 21 June 2020) represented a situation of prolonged stress; this study analyzed the association between changes in functional status observed after the lockdown and 5-year survival.

**Materials and Methods:**

A prospective longitudinal cohort study conducted between 22 June 2020, and 30 June 2025. As of 1 March 2020, Orcasitas (Madrid, Spain) had 150 residents with functional dependence. Four people died during the lockdown. Another 19 were excluded, leaving a final sample of 127 participants. Pre- and post-lockdown functional trajectories were analyzed in relation to the ability to perform basic (ADLs) and instrumental (IADLs) activities of daily living.

**Results:**

After lockdown for the COVID-19 pandemic, 62.2% of these individuals improved their functional capacity; 50.6% did so to a clinically significant degree (25–75 points); and 43.3% stopped meeting criteria for functional dependence according local protocol (Barthel>60). This improvement was associated with less dependence on performing ADLs and IADLs. At the 3-year follow-up, 50% maintained a better score compared to their pre-lockdown Barthel Index, but only 22.2% did so compared to their post-lockdown Barthel Index. A lower Barthel Index score before and after lockdown was associated with a lower probability of survival at 5 years. Maintaining functional dependence (Barthel≤60) after the lockdown was also associated with a higher risk of mortality (HR 2.130; CI 1.322–3.434). Age, having no formal education, living with children, being unable to leave the house independently, and being unable to shop on one’s own, were associated with a lower probability of survival.

**Conclusion:**

Functional trajectories observed after lockdown in this cohort showed that the outcome associated with survival was not the improvement in functional capacity or the intensity of that improvement, but rather exceeding the 60-point threshold on the Barthel Index. In this cohort, a combined analysis of ADLs and IADLs might better reflect the potential capacity to make these changes than an analysis of the Barthel Index alone. The observed functional trajectories suggest as an exploratory hypothesis of a latent intrinsic capacity, construct that would encompass functional reserves not routinely utilized in daily life.

## 1 Introduction

In 2015, the World Health Organization (WHO) introduced the concept of intrinsic capacity to define the distinctive characteristics that enable older adults to live independently and achieve a high level of wellbeing in their environment (Beyene et al., 2024). This concept, which englobes the physical and mental capacities of the individual, covers five areas (cognition, mobility, vitality, sensory, and psychological), and is a fundamental element within the framework of healthy ageing, as well as the concept of overall wellbeing in older adults (Bautmans et al., 2022). However, the concept of intrinsic capacity itself includes aspects that are abstract and difficult to measure and standardize. Therefore, it is necessary to establish biologically plausible and clinically useful subdomains that allow for efficient social and health planning (Beard et al., 2019).

In this regard, functional capacity reflects the interaction between the areas included in intrinsic capacity and the environment and can be approached from two perspectives: functional capacity itself, which refers to a person’s potential or theoretical ability to perform a specific activity; and functional performance, which reflects what the person actually does in daily life and within their real-world environment. On the other hand, dependence on others to perform basic activities of daily living (ADLs) or instrumental activities of daily living (IADLs) reflects the level of assistance needed from another person to perform that activity, thereby reflecting functional performance capacity. This functional performance, in turn, could be defined according to the environment, encompassing basic activities of daily living that take place within the person’s home, and instrumental activities, which include activities that can be performed both inside and outside the home. The concept of environment also includes the community’s living conditions and housing situation, which can facilitate assistance in performing activities. In functional performance, the influence of the environment is greatest, to the point of altering the final outcome, whereas in functional capacity, the influence of the environment may be less pronounced, since it measures internal resources.

Within this capacity scale, each step—having the capacity, truly executing it, needing help to do so, and whether or not that help is available—allows for a different assessment of the individual, which must be integrated to evaluate how life changes influence the final outcome, whether in terms of health or survival.

But other aspects likely need to be addressed as well. Although the terms “latent intrinsic capacity” and “residual intrinsic capacity” are not currently recognized in scientific circles, their existence cannot be ruled out. Latent intrinsic capacity would represent the hidden biological or psychological potential that a person possesses in their genetic code or in their structure (physical and psychological), but which is neither active nor in use until triggered by a stimulus. Unlike residual intrinsic capacity—which would represent the remaining functional capacity that persists in a person as a result of aging or following an illness—latent intrinsic capacity would allow for the reactivation of dormant functions or skills to improve performance, without the need to develop new skills from scratch. However, given current limitations, this construct—latent intrinsic capacity—is merely a hypothesis that requires validation through specific studies.

This new construct, latent intrinsic capacity, could be defined as the combination of physical, mental, and psychosocial capabilities that a person possesses but does not routinely use in daily life, and which serve as an indicator of the person’s intrinsic robustness of baseline health and biological reserve. It would encompass the biological, physiological, and functional reserves that a person does not use in daily life; contribute to explaining aspects of adaptive and compensatory capacity in response to situations; and be one of the pillars of resilience—including physical resilience—and recovery potential. At this time, proposing this approach based on already recognized concepts can only be done within the framework of exploratory hypotheses, which, as previously mentioned, would require a specific approach in future studies.

Furthermore, among the areas included in the concept of intrinsic capacity, one of them, mobility, is particularly relevant because its impairment can be both a cause and a consequence of impairment in other areas. Mobility influences functional capacity, and lower functional capacity is directly associated with lower intrinsic capacity and, inversely, with higher mortality (Sánchez-Sánchez et al., 2024a). However, although functional impairment reduces intrinsic capacity, this impairment may mask a latent intrinsic capacity, different from residual capacity, which could manifest itself in certain situations that cause significant stress for the person. These situations could include the struggle for survival or the search for a way out of unexpected situations involving changes in the environment that alter baseline conditions. Both situations occurred during the lockdown due to the COVID-19 pandemic. This lockdown meant that governments decreed social isolation and established restrictive rules on access to food, which particularly affected the functionally dependent population (Nestola et al., 2020; Blanco-Rambo et al., 2024). On the other hand, the high mortality rate observed among the elderly during this pandemic caused a fear of contagion that encouraged social self-isolation, which also played a role (Gokseven et al., 2022; Moustakopoulou et al., 2023).

The role that these factors may have played on intrinsic capacity, or functional capacity itself, has not been sufficiently addressed, requiring analysis that allows us to better understand the mechanisms that influence resilience, and how intrinsic capacity and responsiveness, or lack thereof, influence survival.

Regarding resilience, several factors play a role in this. These include the presence of protective psychosocial factors (Morse et al., 2023), acquired during the stage of life prior to exposure to the new stressor posed by the COVID-19 pandemic; or the way in which individuals perceive their own aging process, whose objective and subjective burden shapes their actions; or, finally, associated with the life stage in which older adults find themselves, the role of the subjective proximity of death (Petashnick et al., 2022; Westerhof et al., 2023). Also involved is a personal way of coping with adversity, which, within the Sense of Coherence described in Antonovsky’s Health Pattern (Rivera de los Santos et al., 2011; Seah et al., 2022), would encompass all the aforementioned factors within a same construct. All of this is framed within a specific socioeconomic environment and life trajectory, which also influences the results (Tang et al., 2025; Yu et al., 2023).

At the same time, other factors are also involved. Loss of mobility leads to functional dependence, both for performing basic and instrumental activities of daily living. Once functional dependence is established, the current criterion for assessing the mobility domain of intrinsic capacity by evaluating the ability to walk a certain distance likely oversimplifies the wide range of possibilities inherent in this action and likely presents limitations in populations with functional dependence. Expanding the analysis would provide relevant information on intrinsic capacity and allow for the establishment of subgroups susceptible to specific interventions. Among the available options is the assessment of the ability to perform activities of daily living. Although there are various scales that assess this functional capacity, the Barthel Index is the most widely used. Early monitoring with these standardized tools (Tan et al., 2024) can provide relevant information about an individual’s potential capacity to respond to adversity and about their subsequent evolution (Beard et al., 2019; Sánchez-Sánchez et al., 2024a). However, the way in which the Barthel Index assesses mobility is a subject of debate, as it has been suggested that this index may underestimate the importance of mobility (Martín Moreno et al., 2025). Despite this limitation, its use for assessing functional capacity is not in question, as it provides information on functional capacity within the home that is relevant for establishing the potential intrinsic capacity of a subgroup of individuals for whom the classic assessment of the mobility domain—based on the ability to walk a certain distance—also has limitations.

On the other hand, when assessing intrinsic capacity, it may also be necessary to analyze the ability to perform instrumental activities (Wang Q. et al., 2025; Yu et al., 2021a). Indirectly, these activities influence the assessment made using the Barthel index and directly allow for the evaluation of the mobility domain of intrinsic capacity.

Analyzing how the mobility domain of intrinsic capacity affects the outcome in terms of mortality, and how the presence of latent intrinsic capacity in this domain interacts to influence outcomes, is a relevant objective within a potential multisystemic model that attempts to reflect the characteristics of ageing (Silva et al., 2023; Wang Y. et al., 2025; Shin et al., 2025).

All of this is particularly relevant because intrinsic capacity and functional dependence are factors that influence healthy ageing, quality of life, and mortality in older adults (Beyene et al., 2024; Beard et al., 2019; Moustakopoulou et al., 2023; Wang Q. et al., 2025; Waris et al., 2022a). It is therefore necessary to analyze the factors that influence the maintenance of ideal baseline conditions to conduct appropriately scaled health planning and implement specific actions that contribute to the health and quality of life of the older population (Sánchez-Sánchez et al., 2024a; Petashnick et al., 2022; Tan et al., 2024; Silva et al., 2023; Wang Y. et al., 2025; Shin et al., 2025; Waris et al., 2022a; Yang et al., 2024; Yu et al., 2021b). Aspects that take on special significance within the domain of “mobility” of intrinsic capacity, where adverse outcomes are most frequently observed in these aspects (Bautmans et al., 2022; Yu et al., 2021b).

The presence of latent intrinsic capacity could help stratify older adults into heterogeneous groups that allow for a specific approach, with the aim of delaying adverse outcomes (Yu et al., 2021b).

Taking all these premises into consideration, the objectives of this study were: 1: To analyze the functional trajectory for performing activities of daily living during the COVID-19 pandemic lockdown in a population with functional dependence, assessed using the Barthel Index. 2: To analyze the association between the functional changes observed during the lockdown and their persistence at the 3-year follow-up with 5-year survival. 3: As exploratory, hypothesis-generating analyses, to examine the role of Barthel Index activities that require mobility, as well as certain instrumental activities that also require mobility, as estimators of the mobility domain of intrinsic capacity in a population with functional dependence, and their association with 5-year survival. 3: Also, as a hypothesis-generating exploratory analysis, to examine whether the functional trajectory during lockdown might be associated with the presence of “latent intrinsic capacity” in this population, describing the factors that constitute it and its association with 5-year survival.

## 2 Materials and methods

### 2.1 Study population and study design

At the onset of the COVID-19 pandemic in Spain in March 2020, it emerged that there was a need to analyze how the lockdown decreed by the government would affect, in the short and long term, the functional status and survival of people with functional dependence in performing basic activities of daily living who resided in the Orcasitas neighborhood of Madrid (Spain). As of March 2020, this neighborhood had a population of 22,452 residents, of whom 4,308 were aged 65 or older. The group of older adults with functional dependence (n = 150) represented 3.5% of the total older adult population. The entire population of Orcasitas is served by the Orcasitas health center. Among the institutionally recognized contextual and historical aspects, upon which the allocation of social and welfare resources is based, this neighborhood, located in the southern area of the city of Madrid and characterized by a mix of multifamily residential buildings and single-family homes, is considered within the city of Madrid to be a socioeconomically disadvantaged area, where the majority of older adults have a low level of education. Its older population largely consisted of immigrants who arrived after the Spanish Civil War (1936–1939) and settled in shantytowns in this area. During the 1970s and 1980s, neighborhood movements succeeded in enabling residents to participate in the design of their new homes, which they currently occupy. These historical characteristics may have contributed to a strong sense of community and may provide contextual hypotheses regarding resilience, although this was not directly assessed in the present study.

To address the study’s objectives, a prospective cohort study was designed (Figure 1), which analyzed the functional trajectories of individuals with functional dependence in the Orcasitas neighborhood of Madrid (Spain), as reflected in changes in Barthel Index scores and levels of dependence before and after the lockdown, as well as their association with long-term survival (5 years), through longitudinal follow-up. The study design also included a retrospective phase, in which Barthel Index scores prior to the lockdown were obtained; these scores were used to analyze functional changes in relation to the ability to perform basic activities of daily living after the lockdown and at the 3-year mark. Thus, the study design is ambispective.

**FIGURE 1:**
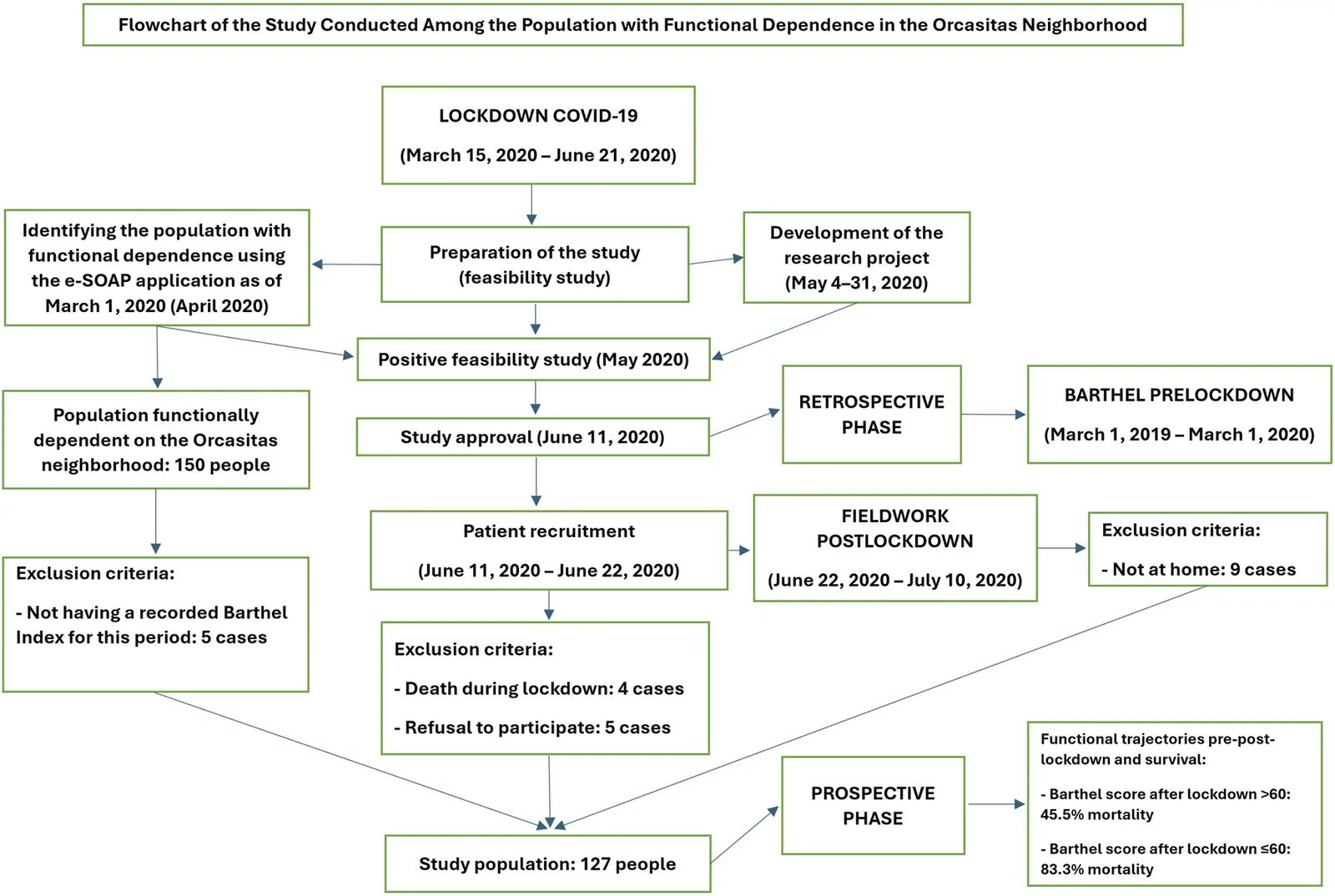
Flowchart of the study design conducted among the population with functional dependence in the Orcasitas neighborhood.

This study included the entire population aged 65 and older who had been diagnosed with functional dependence prior to the lockdown due to the COVID-19 pandemic, establishing 1 March 2020, as the cutoff date for this diagnosis. On the other hand, this date precedes the onset of the COVID-19 pandemic in Spain, where a lockdown was imposed from March 15 to 21 June 2020. This population had been included in the Madrid Health Service’s Protocol for the Care of the Functionally Dependent Population, a protocol integrated into the e-SOAP application at the Orcasitas health center.

Regarding the schedule for the preparation and conduct of the study (Figure 1), in the last week of April 2020, after obtaining the necessary authorizations, we obtained a list of individuals over the age of 65 with functional dependence from the e-SOAP application, and in May 2020, we created a database that included the variables under study. A survey was also developed to collect these variables, which was approved by the Ethics Committee in May 2020. To complete the project’s feasibility study, an initial draft was developed as a research project with three main lines of inquiry, which were submitted to the Local Research Commission, the public entity responsible for approval during the pandemic. Following successive reviews, the final documents were approved in June 2020.

Once health authorities confirmed the end date of the lockdown, teams of two nurses were formed to conduct the fieldwork, and training sessions were held to standardize the administration of the survey and ensure that any questions patients might have were addressed using consistent explanations. Once this research project was approved, the participant recruitment period began, taking place between June 11 and 22, 2020. Finally, the fieldwork was conducted between June 22 and 10 July 2020. Figure 1 illustrates the workflow from study design through implementation.

The inclusion criteria were being aged 65 or over, having a functional capacity assessment using the Barthel index prior to 1 March 2020, with a maximum age of 1 year, and being registered as functionally dependent in the Madrid Health Service’s e-SOAP application (Barthel ≤60). There were 150 patients in this application. The decision to accept a Barthel Index score up to 1 year old as valid was based on the following considerations: 1: The care protocol for individuals with functional dependence in our healthcare system includes an annual assessment of the Barthel Index. 2: The population in this cohort was monitored according to a standardized protocol, with visits every 3 months, or more frequently if a justified request was made. During these visits, information on functional status is collected, and a new Barthel Index is performed when healthcare professionals detect further deterioration compared to the previous visit. This reassessment is also performed when the dependent individual or their family reports a deterioration in baseline conditions and requests a new visit for evaluation. 3: Consequently, a 1-year duration of the recorded Barthel Index score represents stability in the level of functional dependence within our healthcare system.

Between the extraction of the database of individuals with functional dependence (obtained with a date of 1 March 2020) and the conduct of the fieldwork (second half of June 2020), the first wave of the COVID-19 pandemic occurred in Madrid (Spain). During these 3 months of the first wave, four patients died; the cause of their deaths is unknown. The mortality rate in this cohort of individuals with functional dependence for performing activities of daily living during the first wave of the COVID-19 pandemic was 2.7% (n = 4). After the first wave and at the start of the fieldwork, 146 patients were alive.

Additionally, the absence of a recorded Barthel score in the past year (n = 5), the patient’s refusal to participate in the study (n = 5), and not being at their usual residence during the study period (n = 9) were also exclusion criteria. The point in the workflow where these exclusion criteria were applied is clearly shown in Figure 1. None of the patients included in the study had cancer, recent fractures, recent hospitalization, or a COVID-19 infection. Nor was any patient receiving rehabilitation or participating in any type of activity involving functional recovery; such activities were, moreover, suspended during the lockdown due to the COVID-19 pandemic. Although no patients were excluded for these reasons, other exclusion criteria included having cancer or a terminal illness, having been hospitalized within the last month, being in the recovery phase from a concurrent illness, or having experienced a decline in baseline status within the last 15 days. After accounting for deaths and applying the exclusion criteria, 127 patients ultimately participated in this study.

### 2.2 Data collection and definition of variables

Regarding the data collected in the survey (Supplementary Appendix 1), most of it consisted of information that was already routinely and protocolized recorded in the health system’s software prior to the lockdown, as part of standard work procedures. Nevertheless, after designing the survey, a pilot study was conducted with a population of the same age who were not functionally dependent, and it was not necessary to adapt any of the questions. Although the results of this pilot study do not establish its validity in the target population, the lockdown measures imposed during the first wave of the COVID-19 pandemic and the restriction on contact with vulnerable groups—unless medically justified—led to the decision to conduct this pilot study among a population in the same age group who voluntarily agreed to participate when they visited the health center for an appointment.

The nurses responsible for administering the survey had extensive prior experience in recording this data and in how to approach the questions, as this activity was part of their daily routine. The only difference from their daily work routine was the format for recording the information, which became dual: on paper (the survey) and in the protocol included as software in the healthcare system. In addition to this prior experience, four training sessions were held in May 2020 and two in June 2020, with the goal of standardizing criteria regarding how to administer the survey, how to resolve potential issues in interpreting the responses of patients or their family members, and how to enter data into each team’s database. The interpretation of information provided by patients and family members was particularly important, not only because of potential recall bias, but also because of the tendency for patients to perceive their condition as better than it actually is, and for family members to perceive it as worse than the patient describes. In other cases, the situation was the opposite. In these situations, a direct examination of the assessed function, or the negotiation of an agreement between the parties based on a 1-week recall, was established as the criterion for assigning the final result. Furthermore, in patients living with a partner, their children, or other people, this negotiation criterion was also used when the accompanying person showed signs of doubt regarding the response given by the dependent person. In the presence of a Barthel Index score <20 prior to lockdown, or cognitive impairment, the family member’s criterion prevailed over that of the dependent person.

The teams responsible for administering the questionnaire and assessing functional capacity using the Barthel Index consisted of two nurses. To avoid bias, patient assignment was randomized so that the nurse who administered the questionnaire and assessed the Barthel Index was not the patient’s usual nurse, and this nurse was unaware of the patient’s previous Barthel Index score. Additionally, to establish trust with the patient, one of the nurses on this team was the patient’s regular nurse, whose role was to introduce the nurse who would conduct these assessments and to visually confirm whether any changes observed during her colleague’s examination corresponded to a real functional change compared with their pre-lockdown examination. In our community, nursing staff do not care for the entire population of the health center; rather, like family physicians, they are assigned a specific patient population, which designates that nurse as their primary nurse for care and follow-up. As a result, the masking strategy is effective, since they do not care for any population other than the one assigned to them.

Each nursing team subsequently recorded the results in a team-specific, password-protected database. Three of the professionals participating in the study were responsible for coordinating the teams’ records. One of them was in charge of transferring these records to the study’s central database at the health center, and the other two were responsible for verifying that the data had been transferred correctly. Once this was verified, the data was anonymized by assigning a registration number in the central database—which contained no patient-identifying information—and the same number was assigned to the collected survey, from which the top section of the first page was removed along the dotted line.

On the other hand, although most of the data collected by the survey referred to situations occurring in the present, some questions involved retrospective data collection to compare pre- and post-pandemic situations. These questions referred to the instrumental activities of daily living included in the study and the use of mobility aids. Also of a retrospective nature, though with a shorter recall period, were changes or loss of home care assistants for household tasks and personal care.

The data collected through the survey included the following sociodemographic variables: 1: Gender, categorized as male or female. 2: Marital status, categorized as married, widowed, or single (including unmarried, separated, and divorced). 3: Educational level: In the context previously described regarding the social characteristics of the Orcasitas neighborhood, educational level was assessed in two categories: having an education (having completed elementary school or a higher level of education) and insufficient education (illiterate; able to read and write but having no formal education; and not having completed elementary school). 4: Housing situation in the community: This was classified into three categories: living independently (alone or with a partner), living with children, and living with other people who are neither a partner nor children. For practical purposes, the categories “lives with children” and “lives with other people” were combined in some analyses to make comparisons between “living independently” and not doing so. 5: Living arrangements in the community, which were classified as having the ability to leave home or being homebound. For those able to leave the home, the analysis examined whether they did so independently or accompanied by others.

When it came to the ability to perform instrumental activities of daily living, we examined both the current situation and the situation prior to the lockdown. The activities examined were the ability to leave the house, with two response options (YES/NO); the ability to leave the house to go for a walk or engage in leisure activities, which also offered only two response options (YES/NO); and the ability to do the shopping necessary for daily needs, which offered three response options: they do so independently, they need assistance from another person, and they are unable to do the shopping necessary for their daily sustenance. Using the same methodology, the use of mobility aids—such as crutches-cane, walkers, or wheelchairs—was recorded, and participants were asked whether they had already been using that specific device regularly before the lockdown or whether they had been using a different one. The use of one of these devices was not mutually exclusive; a single patient could use more than one, depending on their mobility situation.

Regarding the availability at the patient’s home of caregivers for the dependent person who were not family members, the presence of personal caregivers was recorded. Three categories of caregivers were established: 1: Caregivers assigned by Social Services. 2: Private caregivers hired by the dependent persons or their family members who do not reside at the same address. 3: Internal caregivers. Retrospectively, participants were asked whether there had been any changes in their hired personal caregiver during the lockdown, or whether caregiving activities had ceased.

These three aspects—ability to go shopping, use of mobility aids, and availability of personal caregivers—had already been documented in their medical records prior to the lockdown as part of the clinical and social follow-up of these individuals, and the responses provided can be verified.

Regarding the assessment of functional capacity, in our healthcare system, the level of functional dependence is linked to access to social and economic benefits intended to support these individuals. For this reason, the standard practice regarding these patients established prior to the lockdown, as part of the standard operating procedure, that the assessment of this level of dependence must be conducted under baseline conditions. This criterion implied that the patient’s functional status had not changed in the last month, and also that during this month they had not experienced any intercurrent events that could have affected that functional assessment, such as a hospital admission; a surgical procedure; a change of residence, or temporary admission to a nursing home; a medical condition that, in the healthcare professional’s judgment, could interfere with their functional status; or the loss of a spouse. If any of these situations occurred, the protocol recommends repeating the Barthel Index assessment after 1 month; if the changes persist at that time, this new status is considered the baseline. With regard to this study, no Barthel Index measurements were excluded for any of these reasons.

The Community of Madrid’s Protocol for the Care of Dependent Persons, which is integrated into the health center’s computer system, establishes a Barthel Index score of 60 or lower as the criterion for functional dependence in performing activities of daily living. Functional dependency was categorized into two levels: moderate (Barthel 40–60) and severe (Barthel 0–35). The scores assigned to each level of ability to perform each activity on the Barthel Index and the approach taken regarding the use of mobility aids are outlined in Supplementary Appendix 2: Barthel Index.

In our environment, a score of 60 or lower is the criterion for inclusion in the referred protocol. The Barthel Index ranges from 0 to 100 points. Thus, individuals in this cohort have a margin for improvement in functional capacity that includes the interval between 60 and 100 points; conversely, there is no such margin for loss of functional capacity, since individuals who are already at 0 cannot lose any more points. This situation affects the interpretation of the results in terms of the degree of improvement or loss in functional capacity that can be recorded, as well as the comparisons made when these two situations are contrasted.

Therefore, for operational purposes, we empirically established the following classification of the clinical significance of the increase in the Barthel Index score that might be observed between assessments conducted before and after the lockdown due to the COVID-19 pandemic: 1: Moderate clinical improvement: an increase in the Barthel Index score of between 5 and 20 points. 2: Significant clinical improvement: an increase in the Barthel Index score of between 25 and 95 points. The cutoff point of 25 was established empirically, as it was considered that reaching this threshold necessarily requires improvement in at least three activities on the Barthel Index, thereby preventing a one-time improvement in a single activity from introducing bias.

Once these criteria were established, the following steps were taken to assess functional capacity:

- The Barthel Index score recorded in the health center’s computer system was obtained for the period between 1 March 2019, and 1 March 2020. When multiple scores were available, the one closest to 1 March 2020, was recorded.
- A new Barthel Index assessment was conducted between June 22 and 10 July 2020, following the end of the lockdown due to the COVID-19 pandemic. The difference between the pre- and post-lockdown scores made it possible to establish the following outcome categories: 1: The score worsened after the COVID-19 pandemic lockdown; 2: The score improved after the COVID-19 pandemic lockdown; and 3: The score remained the same after the COVID-19 pandemic lockdown.
- Finally, in 2023, midway through the follow-up period, a new assessment of functional capacity was conducted using the Barthel Index, to verify whether any differences observed in 2020 at the midpoint of the study period persisted, and to compare these findings with both the post-lockdown changes and the final 5-year results.

Functional capacity encompasses intrinsic capacity, the environment, and context. If intrinsic capacity can be broadly defined as the set of physical and mental abilities a person has at a given moment, the term “latent” would include in this definition those abilities that the individual also potentially has but does not actively manifest until an event of sufficient intensity occurs. These unmanifested capacities, when they refer to functional capacities to perform basic activities of daily living, could be measured through changes in the Barthel Index; changes whose intensity or lack of a recognized justification for their manifestation could reveal the presence of this latent intrinsic capacity.

In order to distinguish “normal” changes in the course of patients with functional dependence from those that could be considered associated with the manifestation of a latent intrinsic capacity, classification criteria were established. These criteria were designed empirically to classify the changes observed in the functional trajectories of patients in this cohort and evaluate their relevance from a personal, clinical, and social perspective:

First, for operational purposes, the following classification was established for the clinical significance of the increase in the Barthel Index score observed between assessments conducted before and after the lockdown due to the COVID-19 pandemic. This classification included two levels: 1: Clinically moderate improvement: an increase in the Barthel Index score of between 5 and 20 points. 2: Clinically relevant improvement: an increase in the Barthel Index score of between 25 and 95 points. The cutoff point of 25 was established empirically, as it was considered that reaching this threshold would likely require improvement in half of the activities on the Barthel Index, or at least significant improvement in at least three activities. This criterion made it more likely to observe an overall functional response, as opposed to a specific improvement in a single activity that could introduce bias into the results.

The second approach did not refer to the score itself, but rather to the level of functional dependence into which that score allowed the patient to be classified. The score obtained allowed for reclassification within the dependency levels established in our health system’s Functional Dependency Protocol. This Protocol only includes individuals with a Barthel Index of 60 or lower. As we have previously mentioned, in our context, functional dependence entails monitoring and benefits provided by both the healthcare system and social services. Having or losing access to these services and benefits is highly relevant. The reclassification of patients could have an impact on their daily lives if these changes were confirmed in subsequent follow-ups.

For this reason, the perspective of this second classification was no longer merely clinical, but also personal and social. Assessment criteria that could be standardized were needed. To integrate both perspectives—that crossing the 60-point threshold on the Barthel Index entails changes in social and healthcare benefits, and marks a turning point in the individual’s functional trajectory, and the perspective that, in the face of an unexpected situation, abilities that are not normally used may emerge—abilities that could serve as an indicator of the intrinsic robustness of the individual’s baseline health and biological reserve, thereby supporting the hypothesis of a latent intrinsic capacity—the following operational classification was established: 1: The patient continues to meet the criteria for functional dependence after the lockdown according to the local functional dependence protocol (Barthel ≤60); this situation could result from a decline in the patient’s pre-lockdown functional capacity, from maintaining that capacity within the levels of functional dependence, or from an improvement in the pre-lockdown functional level while still maintaining a Barthel Index score of ≤60. Or 2: The patient’s pre-lockdown functional level improved beyond the level of moderate functional dependence, a situation that would entail a Barthel Index score greater than 60, thereby no longer classifying the patient as a person with functional dependence according to the protocol in effect in our healthcare system. Statistical analyses were conducted using this classification as a reference.

The difference between the two classification systems is that in the first, the classification is based on the intensity of the response as measured by a score. In the second, since this new score translates into a specific level of functional dependence—or the absence of such a level, according to our protocol—the classification is based on the impact this new situation would have on the level of health and social care the person previously received.

On the other hand, maintaining a higher Barthel Index score in 2023 compared to the score prior to the lockdown implied that functional improvement was sustained over time, a situation that could make it possible to rule out other types of adaptive responses to acute stress.

Furthermore, as part of this study’s objective to conduct an exploratory analysis that would allow for the formulation of hypotheses regarding the indirect assessment of the mobility domain of intrinsic capacity through the activities of the Barthel Index, we analyzed the influence of the capacity to perform the four activities of this index for which mobility is an essential requirement: walking, going up and down stairs, chair-to-bed transfers, and toilet use, on the observed results, both in relation to changes observed in Barthel Index scores during the 100-day lockdown at home due to the COVID-19 pandemic and in relation to survival. For operational purposes, these variables were dichotomized, establishing two groups: 1: Dependent or requiring significant assistance to perform that activity; 2: Independent or requiring minimal assistance to perform that activity. To facilitate reading the text, Group 1 was designated “Dependent” and Group 2 “Independent.” This criterion was established based on the size of certain sample subgroups, which would make statistical analysis difficult, and the type of regrouping was justified on the grounds that being independent or requiring minimal assistance would imply maintaining mobility to a greater or lesser degree, whereas being dependent or requiring significant assistance would imply that such mobility is limited or nonexistent.

As part of this exploratory analysis of the mobility domain of intrinsic capacity through aspects related to how this mobility is achieved, an analysis was conducted on the relationship between the study variables and the use of mobility aids, such as crutches-cane, walkers, or wheelchairs.

Finally, regarding the assessment of mobility, the ability to perform certain instrumental activities of daily living was analyzed. In these activities, mobility was an essential criterion for their performance and categorizing them into two groups made it easy to compare two opposing functional situations with respect to mobility. First, the ability to leave home was analyzed, as opposed to being unable to do so and having to live homebound. Second, we analyzed how this activity was carried out outside the home, distinguishing between the ability to leave home for leisure activities or walks, to purchase necessities, and to make these purchases independently or under the supervision of another person. These variables was analyzed dichotomously (Yes/No).

In addition, data regarding income level was obtained from the Individual Health Card (IHC) application within the health center’s computer system. This application established two income categories: 1) over 11,200 euros per year; and 2) under 11,200 euros per year. This cutoff point served as the criterion for copayments for pharmacy services, with such services being provided free of charge when income was less than 11,200 euros per year. This cutoff point was established by the Madrid Regional Health Department. Therefore, having an income of less than 11,200 euros per year was equivalent to having a low income.

Data on the burden of chronicity, the number of prescribed drugs and body mass index were also obtained from the computer system via the AP-Madrid application. Regarding chronic diseases, the criteria of the Community of Madrid’s Strategy for the Care of People with Chronic Diseases (SCPCD) (Consejería de Políticas Sociales y Familia, 2017) of the Community of Madrid for 2014–2017, extended through 2021, including in the analysis the presence of type 2 diabetes mellitus, chronic obstructive pulmonary disease, stroke, hypertension, myocardial infarction, heart failure, asthma, obesity, dyslipidemia, and renal failure. Statistical analyses were conducted using two criteria: 1. Number of chronic diseases; 2. Burden of chronicity. To determine the burden of chronicity, a cutoff point was empirically established to allow for better management of this confounding variable in subsequent analyses; this cutoff was set at having at least half of the diseases included in the SCPCD, and this variable was dichotomized into two categories: 1: Having fewer than five chronic diseases; and 2: Having five or more chronic diseases, which was defined as having a high disease burden. In the latter situation, patients are likely to present greater clinical complexity, which would include higher medication use and a higher level of risk and intervention assigned by the healthcare system.

Under this Strategy, patients are classified into risk levels and intervention levels by the Community of Madrid’s health system using automated algorithms built into the computer system. The operational definitions of these levels are as follows:

1. Risk Levels: The risk level is calculated automatically using tools (Adjusted Morbidity Groups) that assess the burden of disease based on the electronic health record:
  1.a. Low Risk: Patients with chronic conditions in the early stages or considered to be of low complexity.
  1.b. – Medium Risk: Patients with multiple conditions that, taken together, represent moderate complexity but require periodic monitoring to prevent their condition from worsening.
  1.c. – High Risk: Complex chronic patients with high comorbidity, a high probability of decompensation, frequent admissions, and significant social and healthcare needs.

For operational purposes related to the sample size of the potential subgroups, this variable was dichotomized in this study: 1: High risk. 2: Low or moderate risk.

2. Levels of Intervention: Primary care professionals assign the level of intervention required by the patient by combining the risk data automatically provided by the computer system with their actual knowledge of the patient and his or her social environment. Classification Levels:
  2.a. Low Intervention: Objective: To slow the progression of the disease and promote independence. Strategy: Promotion of self-management and health education.
  2.b. Medium Intervention: Objective: To actively manage chronic disease. Strategy: Specific disease management programs with scheduled visits and monitoring of risk factors.
  2.c. High-Level Intervention: Objective: To stabilize complex patients, prevent hospital admissions, and coordinate social resources. Strategy: Case management, intensive personalized care plans, and close coordination between primary care and specialized care.

For operational purposes related to the sample size of the potential subgroups, this variable was dichotomized in this study: 1: High level of intervention. 2: Low or moderate level of intervention.

Regarding the number of prescribed drugs the patient was taking, polypharmacy was defined as having five or more prescribed drugs, and the absence of polypharmacy as having fewer than five prescribed drugs. This variable was analyzed dichotomously, following the protocol established in our healthcare system for classifying patients as polymedicated: Polymedicated: prescribed five or more drugs. 2: Not polymedicated: prescribed fewer than five drugs.

Finally, regarding mortality, the date of death for the participants in this study was obtained from the records in the Madrid Health Service’s computer system. This system provided the exact date of death, and overall 5-year mortality was analyzed. The start date for mortality follow-up was set as the day following their inclusion in the study, after they signed the informed consent form and underwent the examinations included in the study. The administrative censoring date was set as 30 June 2025, following mortality until that date. The mortality follow-up period for each participant was 5 years ±10 days. All 127 study participants were followed during this period, with no losses to follow-up recorded. The researcher who conducted this follow-up did not have access to the participants’ medical records or to the data collected in the surveys. Similarly, the healthcare professionals participating in this study did not have access to the computer systems where the date of death was recorded.

### 2.3 Data analysis

The SPSS® 18.0 statistical package was used for data analysis. A descriptive analysis of the cohort was performed using mean/standard deviation for continuous variables and numbers/proportions for categorical variables. The normality of the data distribution was analyzed using the Kolmogorov-Smirnov test. In the case of a non-normal data distribution, the Wilcoxon signed-rank test was used for the before-and-after comparisons. Differences between continuous variables were analyzed using Student’s t-test or the Mann-Whitney U test, and differences between categorical variables were analyzed using the chi-square test. With regard to the chi-square test and expanding on the standard criteria for using Fisher’s exact test, this study also applied Fisher’s exact test when the observed frequencies were less than 10. As a measure of effect size, the strength of the association observed between two categorical variables using the chi-square test was analyzed using the odds ratio (OR). To control for baseline severity in the two groups analyzed—those whose Barthel Index scores improved and those whose scores worsened after lockdown—the pre-lockdown baseline score was included as a covariate in an Analysis of Covariance (ANCOVA) model using linear regression. This allows for adjusting the final group differences based on the initial value. In this study, any p < 0.05 was considered significant.

### 2.4 Survival analysis

In this study, the primary outcome measure is time to death. Covariates were selected based on clinical and social criteria. From a clinical perspective, the criteria included being a polymedicated patient, having a high chronicity burden, being assigned a high-risk level, and being assigned a high-intervention level. In our healthcare system, these covariates correspond to complex chronic patients, for whom poorer health outcomes are expected. Covariates that were also clinically relevant but had a clear social dimension—due to their primary management by social services—included educational level, income level, marital status (married or widowed), living independently or with children, availability of caregivers, use of mobility aids, and age. These social covariates, with cumulative effects, empirically represent potential situations in our setting that may increase health risks (poverty, illiteracy or low educational attainment, loneliness, mobility difficulties, and reliance on social services for personal care).

The survival analysis was performed using Cox regression, adjusted for baseline severity, as represented by the level of functional dependence prior to lockdown. In the model development process, in a first step, the three main covariates that justify this study (improvement or worsening of functional capacity, intensity of functional improvement, and dependent or independent status after confinement according to local protocol) were introduced into separate models for each of them. Subsequently, we introduced into each of these three models the four clinically relevant covariates, along with age and sex; finally, we included the remaining clinical and social covariates within the proportional hazards model used by SPSS®. The resulting model was summarized using the estimated coefficients, p-values, and hazard ratios (HR) with their 95% confidence intervals. By default, SPSS® uses the Breslow method to resolve ties.

With regard to tied values, SPSS® treats tied times as if they occurred sequentially but assumes that all subjects with the event at that moment share the same risk set. If there are too many ties, this situation could slightly bias the coefficients β and standard errors toward zero. With a sample of 127 individuals and 23 ties (18.1%) distributed over time, with a maximum of five ties per month of follow-up (one case), two cases with four ties/month, nine cases with three ties/month, and 10 cases with two ties/month over a 60-month period, the volume of ties per unit of time is considered low compared to the available risk data; therefore, the Breslow default method is generally appropriate, and it is expected that it will not significantly bias the results. This method was routinely used in this study. However, for the covariate “level of improvement in the Barthel Index score after lockdown,” the Efron method was used to resolve ties. This decision was justified by the small sample size of the male cohort (n = 27).

As for the censored cases, the censoring criterion was the status variable linked to survival, dichotomized as “alive” or “deceased.” With regard to the assumption of independent or non-informative censoring, since the final status of patients could take three forms (continuing to live in the community, living in a nursing home, or having died), in order to control for censoring, the data analysis was performed by comparing these three statuses with one another. Reasons for censoring were documented through active follow-up, and the Cox analyses assume non-informative censoring conditional on the available covariates. The mortality data included the exact date of death.

To avoid overfitting the model, given the small sample size, the minimum required sample size was calculated using the following formula:

$$Nmin=\frac{10\;x\;k}{P}$$

where k is the number of independent predictor variables evaluated. The prevalence value (P) used was 0.75, which represented the expected 5-year mortality rate derived from preexisting and previously published data within the Community of Madrid’s healthcare system (75% 5-year mortality rate for the population with moderate functional dependence and 78% for the population with severe functional dependence) (Consejería de Políticas Sociales y Familia, 2017). This proportion was based on previously reported 5-year mortality estimates for the total population of individuals with functional dependence residing in the Community of Madrid, a group that included the population with functional dependence in the Orcasitas neighborhood.

Furthermore, to estimate the incremental prognostic value of the functional change that might be observed after lockdown, and to analyze whether that change could provide predictive information beyond that provided by the pre-lockdown baseline, stepwise predictive modeling was performed using logistic regression. These steps were: 1: Create a baseline clinical-social model to analyze the effect of the variables included on survival (Clinical-Social Baseline). 2: Add to this model the initial functional status, defined by the pre-lockdown Barthel score (Line of Base) 3: To this model defined as the line of base, the difference between the pre- and post-lockdown Barthel Index scores was added (Incremental Model). The incremental model was analyzed over two time periods: 1: June–July 2020, following the end of the lockdown; 2: June 2023, after 3 years of follow-up. The metrics used to evaluate the incremental value were: 1: Discrimination of which model best classifies the occurrence of an event, using the area under the ROC curve (C-statistic). 2: Assessment of the model’s overall fit to determine whether the functional change provides real predictive information that may have clinical and/or social implications, using the Akaike Information Criterion (AIC) and the Bayesian Information Criterion (BIC).

## 3 Ethics statement

All subjects participated voluntarily in the study, were informed of its objectives, and signed an informed consent form to participate. Participants with decision-making capacity provided written informed consent themselves. When a legal guardian was present and proxy consent was required, the legal guardian signed the consent form. In specified cases of severe dependence or cognitive impairment, a family member provided additional authorization. Participant assent was sought whenever applicable. The data collected were anonymized and entered into databases in Excel® and SPSS®. The study adhered to the regulations set forth in Spain’s Data Protection Act 3/2018 and the 2013 version of the Declaration of Helsinki, as in effect in 2020, as well as the regulations of our healthcare institution. Since this study is part of a broader project, the data necessary to interpret the results have been included in the text of the manuscript or in its appendices.

The data collected were anonymized and entered into databases in Excel® and SPSS®. The study adhered to the regulations set forth in Spain’s Data Protection Act 3/2018 and the 2013 version of the Declaration of Helsinki, as in effect in 2020.

During the first wave of the COVID-19 pandemic in Madrid (Spain), the high number of proposed research studies overwhelmed the capacity of the Ethics Committees. To ensure the feasibility of these studies, those originating from primary care were reassigned to be reviewed by the Local Primary Care Research Committees, which were granted the same decision-making authority and criteria as the Ethics Committees. This study was approved by the Local Research Commission of the Assistance Directorate Center dependent on the Primary Care Management of the Department of Health of the Community of Madrid (Spain), resolution 14/20-C, of 11 June 2020. The Ethics Committee of Hospital Universitario Doce de Octubre endorsed this as sufficient approval, resolution 23/501 of 26 September 2023.

## 4 Results

To maintain the line of reasoning described above, in relation to the stated objectives, this section has been divided into the following subsections:

1. Sociodemographic data from the Orcasitas cohort.
2. Functional trajectories observed during the pre- and post-lockdown periods and the persistence of functional changes over the long term.
3. Survival analysis at 5 years of follow-up.
4. Analysis of confounding, concurrent, and mediating factors. Associations and exploratory analyses that generate hypotheses.
5. Exploratory analysis of the role of mobility-related activities in the Barthel Index, as a basis for the hypothesis of an expanded model that allows for the assessment of the mobility domain of intrinsic capacity in individuals with functional dependence.

## 5 Sociodemographic data from the orcasitas cohort

The sociodemographic characteristics of this cohort, known as the Orcasitas cohort, are summarized in Table 1. This cohort had a high proportion of women (78.7%) and had a low level of education (88.1%), and 47.2% of its members having low incomes. Regarding marital status, the predominant status was widowed (64.6%), with 30.7% being married. Although the majority (44.9%) lived with their children, 37% continued to live independently, either alone or with a partner, and one in three people lived homebound. The most commonly used mobility aid was crutches-cane (42.5%), followed by a walker (35.4%) and a wheelchair (32.3%). Most people in this cohort were unable to independently perform the daily shopping necessary for their subsistence (85%), delegating such shopping to another person (71.6%), or performing it under the supervision of another person (13.4%). These characteristics defined a cohort in which 70.1% had moderate dependency and 29.9% had severe dependency.

**TABLE 1:** Sociodemographic and socioeconomic data of the functionally dependent population of Orcasitas (Orcasitas cohort).

Sociodemographic and socioeconomic data of the orcasitas cohort			
Variable	Value	Variable	Value
Sex (n = 127):	​	Education^a^ (n = 126):	​
Male	21.3% (27)	Not education	88.1% (111)
Female	78.7% (100)	Had education	11.9% (15)
Income level (n = 127):	​	Living arrangements (n = 127):	​
Less income (<11.200 euros/year)	47.2% (60)	Leaving home	64.6% (82)
Non low income (≥11.200 euros/year)	52.8% (67)	Homebound	35.4% (45)
Marital status (n = 127):	​	Housing situation (n = 127):	​
Married	30.7% (39)	Lives independently	37% (47)
Widower	64.6% (82)	Lives with children	44.9% (57)
Others	4.7% (6)	Lives with others who are not children	18.1% (23)
Live with a partner (n = 125):	​	Widower (n = 67):	​
Yes	31.2% (39)	Lives independently	32.8% (22)
No	68.8% (86)	Lives with children	67.2% (45)
Married (n = 34):	​	Lives with others who are not children (n = 20):	​
Lives independently	70.6% (24)	Married	25% (5)
Lives with children	29.4% (10)	Widower	75% (15)
Classic barthel index level (n = 127):	​	Leaves home (n = 84):	​
Severe (under 40)	29.9% (38)	Alone	19.1% (16)
Moderate (40–60)	70.1% (89)	Accompanied	80.9% (68)
Mobility aids^b^:	​	Shopping (n = 127):	​
Wheelchair	32.3% (41)	Independently	15% (19)
Walker	35.4% (45)	Help of another person	13.4% (17)
Crutches-cane	42.5% (54)	Dependent on another person	71.6% (91)

^a^Education level: Had education: having completed elementary school or a higher level of education; Not education: illiterate; able to read and write but having no formal education; and not having completed elementary school.

^b^ The use of mobility aids is not mutually exclusive; therefore, a single person may use more than one device, depending on their mobility needs.

## 6 Functional trajectories observed during the pre- and post-lockdown periods and the persistence of functional changes over the long term

### 6.1 Functional trajectories observed following the COVID-19 pandemic lockdown. A comparative analysis of the intensity of the functional response relative to the pre-lockdown functional status

Following the framework proposed in the Materials and Methods section, we present the results of the functional changes observed after the COVID-19 pandemic lockdown, compared to pre-pandemic baseline values, in the following order of analysis: 1: Improvement or worsening of functional capacity to perform basic activities of daily living. 2: Among the group that shows an improvement in functional capacity, an analysis of the intensity of this improvement in terms of score, classifying it as moderate (5–20 points) or clinically relevant (25–95 points). 3: The Barthel Index score obtained after the lockdown assigns a functional level: 1: Functional dependence persists (the individual maintains a severe or moderate level of functional dependence). 2: The patient does not meet the criteria for functional dependence according to the local protocol (the level of functional improvement does not allow the patient to be classified within the two levels of dependence included in that protocol: moderate and severe). The descriptive statistics for these analyses are summarized in Table 2.

**TABLE 2:** Descriptive statistics for each functional group and assessment period of functional capacity using the Barthel Index.

Descriptive statistics of functional capacity estimated according to the barthel index in each functional group					
Timeline of functional capacity assessments according to the barthel index	Status of functional capacity assessments	Descriptive statistics			
		Mean ± SD^a^	95% confidence interval	Interquartile range	Minimum-maximum (range)
Follow-up periods					
Barthel index score	Prior lockdown^b^ (n = 127)	43.28 ± 19.017	39.97–46.59	30	0–60 (60)
	After lockdown^c^ (n = 127)	54.41 ± 26.115	49.87–58.95	40	0–95 (95)
​	June 2023 (n = 54)	48.52 ± 21.884	42.68–54.36	25	0–95 (95)
Changes in functional capacity between pre- and post-lockdown					
Change in barthel index scores between pre- and post-lockdown (n = 112)	Improvement after lockdown (n = 79)	44.19 ± 18.646	40.01–48.37	25	0–60 (60)
​	Worsening after lockdown (n = 33)	43.79 ± 17.546	37.57–50.01	28	5–60 (55)
Intensity of the improvement in barthel index scores between pre- and post-lockdown (n = 79)	Improves their score by 5 to 20 points after lockdown (n = 39)	48.46 ± 17.250	42.87–54.05	20	0–60 (60)
	Improves their score by 25 to 95 points after lockdown (n = 40)	40.03 ± 19.219	33.88–46.17	34	0–60 (60)
Change in functional level between pre- and post-lockdown (n = 127)	Not dependent after lockdown^d^ (n = 55)	52.98 ± 11.601	49.85–56.12	10	0–60 (60)
​	Dependent after lockdown^e^ (n = 72)	35.86 ± 20.266	31.10–40.62	35	0–60 (60)
Changes in functional capacity between pre- and June 2023					
Change in barthel index scores between pre- and June 2023 (n = 47)	Improvement after lockdown (n = 33)	57.73 ± 18.628	51.12–64.33	20	20–95 (75)
	Worsening after lockdown (n = 14)	31.43 ± 18.649	20.66–42.20	28	0–55 (55)
Intensity of the improvement in barthel index scores between pre-lockdown and June 2023 (n = 33)	Improves their score by 5 to 20 points after lockdown (n = 13)	52.69 ± 15.359	43.41–61–97	18	20–75 (55)
	Improves their score by 25 to 95 points after lockdown (n = 20)	61.00 ± 20.170	51.56–70.44	28	20–95 (75)
Change in functional level between pre-lockdown and June 2023 (n = 54)	Not dependent after lockdown^d^ (n = 31)	58.39 ± 17.672	51.90–64.87	20	20–95 (75)
	Dependent after lockdown^e^ (n = 23)	35.22 ± 20.140	26.51–43.93	30	0–75 (75)

^a^ SD, Standard deviation.

^b^ Pre-lockdown: Barthel Index estimated between 1 March 2019, and 28 February 2020.

^c^ Post-lockdown: Barthel Index estimated in June–July 2020.

^d^ Not dependent after lockdown: improved their functional capacity and no longer meet the local protocol criterion for functional dependence (Barthel >60).

^e^ Dependent after lockdown: Maintains the functional dependence after the lockdown as it had before the lockdown (Barthel ≤60).

Prior to these analyses, the distribution of pre-lockdown Barthel Index scores did not meet the criteria for a normal distribution (Kolmogorov-Smirnov 0.231; p < 0.001). Neither did the post-lockdown scores (Kolmogorov-Smirnov 0.112; p < 0.001). The Wilcoxon signed-rank test for comparing pre-lockdown Barthel Index scores (mean ± standard deviation 43.28 ± 19.017; range 0–60) and post-lockdown scores (54.41 ± 26.115; range 0–95) showed a significant difference (z = −5.276; p < 0.001). The median before the lockdown was 50, while the median after the lockdown was 60.

Regarding the overall analysis of the changes observed after the lockdown, compared to their baseline values prior to the lockdown, it was observed that 62.2% (n = 79) of these individuals showed an improvement in their functional capacity. Another 26% (n = 33) experienced worsening functional capacity compared to pre-lockdown values, and the remaining 11.8% (n = 15) maintained their baseline scores. When analyzing whether there were differences in baseline severity, it was observed that the differences between the mean scores on the pre-lockdown Barthel Index in the group that improved its functional capacity (44.19 ± 18.646; CI 40.01–48.37) and the group that worsened (43.79 ± 17.546; CI 37.57–50.01) were not significant (Mann-Whitney U = 1218.500; z = −0.555; p = 0.579). These results, in greater detail, are presented in Section 1 of Supplementary Appendix 3: Orcasitas Study: Descriptive statistics for pre-lockdown Barthel scores regarding the outcomes “improvement or worsening in Barthel score after the COVID-19 pandemic lockdown.”

Since an improvement in functional capacity to perform basic activities of daily living following the COVID-19 pandemic lockdown was defined as a positive outcome, the influence of baseline severity was analyzed using ANCOVA and linear regression. Based on this analysis (Section 2 of Supplementary Appendix 3: ANCOVA for pre-lockdown Barthel scores regarding the outcomes “improvement or worsening in Barthel score after the COVID-19 pandemic lockdown”), Levene’s statistic (0.147; p = 0.702) indicated homogeneity of variances, allowing for a comparison between the two groups; meanwhile, the differences in the mean pre-lockdown Barthel Index scores between the groups that improved and those that worsened in functional capacity during the lockdown, as well as the tests for inter-subject effects (p = 0.988), were not significant.

Following this initial assessment, an analysis of the magnitude of this improvement in terms of scores and clinical significance showed that 49.4% (n = 39) of the individuals who showed an improvement in functional capacity when comparing pre- and post-lockdown assessments experienced a moderate improvement, increasing their Barthel Index score by between 5 and 20 points. Meanwhile, 50.6% (n = 40) showed a clinically relevant increase in functional ability, with an improvement of between 25 and 75 points. In contrast, 75.8% (n = 25) of the individuals whose functional ability worsened between the two assessments did so to a moderate degree, and 24.2% (n = 8) to a clinically significant degree, with losses of between 25 and 40 points on the Barthel Index.

Among the group that improved their functional capacity after lockdown, the intensity of the response in terms of scores showed that those who improved moderately (5–20 points) had a higher mean baseline score prior to lockdown (48.46 ± 17.250; CI 42.87–54.05) than those who showed an increase of 25–95 points, considered clinically relevant (40.03 ± 19.219; CI 33.88–46.17) (Section 3 of Supplementary Appendix 3: Orcasitas Study: Descriptive statistics for “Intensity of improvement in functional capacity to perform basic activities of daily living following the COVID-19 pandemic lockdown”), with the differences being statistically significant (Mann-Whitney U = 556.500; Z = −2.258; p = 0.024).

It was also relevant to the purpose of this study to determine the magnitude of these changes in functional capacity following the lockdown for each participant (Section 4 of Supplementary Appendix 3: Orcasitas Study: Descriptive statistics on the “Magnitude of change in Barthel Index scores at the individual level between the pre-lockdown baseline and the assessments conducted after the lockdown and in 2023”). Following the lockdown, the greatest loss of functional ability was 40 points on the Barthel Index, while the greatest improvement observed at the individual level was 75 points. Compared to pre-lockdown values, in June 2023 the greatest individual functional decline was 65 points, while some individuals maintained their capacity for functional improvement, with the greatest increase relative to their pre-lockdown value being 60 points. When this analysis was conducted with respect to post-lockdown values, the greatest individual loss observed was 55 points, and the greatest individual functional recovery was 45 points.

These points could be particularly relevant in the subsequent interpretation of the results, since individuals whose baseline functional capacity was close to the established threshold of 60 required a lower response intensity—in terms of score—to improved their functional capacity to a level of not being functionally dependent according to the local protocol (Barthel >60). Therefore, this factor was analyzed, revealing that the improvement in functional capacity required to exceed this 60-point threshold involved an increase of between 5 and 20 points in 47.3% (n = 26) of cases, while the remaining 52.7% (n = 29) had to improve their functional capacity by between 25 and 95 points to reach this threshold. These differences based on the intensity of the response were not statistically significant (χ^2^ = 0.318; p = 0.573).

Finally, in the analysis of the functional levels achieved following the lockdown due to the COVID-19 pandemic, among those who improved their functional capacity after the lockdown, 69.6% (n = 55) improved to such a significant degree that their score exceeded the threshold of 60 established in the protocol. Across the entire cohort, this result meant that 43.3% of the individuals who, prior to lockdown, were assessed as functionally dependent for performing basic activities of daily living, no longer met the criteria for functional dependence, according to our healthcare system’s Protocol for the Care of Dependent Persons.

When this last result was analyzed in greater detail (Section 5 of Supplementary Appendix 3: Orcasitas Study: Descriptive statistics for “Remains functionally dependent after lockdown due to the COVID-19 lockdown (Barthel score of 60 or lower) versus no longer meeting the criteria for functional dependence established in the protocol and achieving independence (Barthel score >60)”), individuals with functional dependence in this cohort who achieved a clinically relevant functional improvement that allowed them to be excluded from the health center’s functional dependence protocol (Barthel >60) had a higher Barthel Index score prior to lockdown (52.98 ± 11.601; CI 49.85–56.12) than those who did not achieve this outcome (35.86 ± 20.266; CI 31.10–40.62) (Mann-Whitney U = 918.500; Z = −5.280; p < 0.001).

Using the level of functional dependence prior to lockdown as a reference—rather than the score—it was observed that among people with severe dependence, 68.8% saw an improvement in their functional capacity during lockdown, while 31.3% showed a worsening. These percentages were 71.3% and 28.8%, respectively, among people with moderate dependency. Functional improvement was observed in both levels of dependency, with no significant differences between them (χ^2^ = 0.069; p = 0.793). The same result was observed with regard to response intensity in terms of score (χ^2^ = 3.757; p = 0.053). While only 17.9% (n = 7) of those who improved their baseline score by 5–20 points (n = 39) had severe dependency, 37.5% (n = 15) of those who improved by more than 25 points had this level of dependency. These percentages were 82.1% (n = 32) and 62.5% (n = 25), respectively, among individuals with moderate dependency. However, the level of dependence did influence whether this improvement in functional capacity allowed participants to achieve a Barthel Index score greater than 60. Among those who remained dependent after the lockdown (n = 72), 47.2% (n = 34) had severe dependence, while among those who no longer meet the local protocol criterion for functional dependence (Barthel >60; n = 55), only 10.5% (n = 4) had that level of dependence (χ^2^ = 23.733; p < 0.001).

### 6.2 Persistence of changes in functional capacity during longitudinal follow-up. functional and vital trajectories at 3 years

As part of this follow-up, a new functional assessment was carried out in 2023 using the Barthel Index. Of the initial cohort of 127 people, 42.5% (n = 54) were still living in the community, 41.7% (n = 53) had died, 12.6% (n = 16) were living in a nursing home, and 3.2% (n = 4) were still living in the community but refused to participate.

Among those who were still alive but residing in a nursing home, 75% (n = 12) were individuals whose functional capacity scores had improved during the lockdown; this improvement meant that 58.3% (n = 7) of them no longer meet the local protocol criterion for functional dependence (Barthel >60). Another 13.3% (n = 2) were people whose functional capacity had worsened, and finally, 13.3% (n = 2) were people who maintained the same score on the Barthel index. Regarding the intensity of this improvement, 50% (n = 6) showed an improvement of between 5 and 20 points, and the other 50% (n = 6) showed an improvement of between 25 and 95 points. When comparing these results with those observed among participants who continued to live in the community (42.1%; n = 16 and 57.9%; n = 22, respectively), the differences were not significant (χ^2^ = 0.231; p = 0.631; Fisher’s exact test 0.743). Nor were there differences between the two groups regarding whether this improvement meant no longer meeting the functional dependence criteria of the local protocol (Barthel >60; χ^2^ = 0.978; p = 0.323; Fisher’s exact test p-value 0.400). Compared to the 57.6% (n = 34) of individuals who reached this level and lived in the community, among those living in a nursing home, this percentage was 43.7% (n = 7).

In this study, no significant differences were observed between whether or not functional capacity improved after the lockdown, nor between the intensity of that improvement in relation to the score or functional level achieved after the lockdown, with respect to the final outcome at 3 years of continuing to live in the community or living in a nursing home.

Of the cohort that continued to reside in the community and was re-evaluated using the Barthel index in 2023, 61.1% (n = 33) were people who had improved their functional capacity during lockdown in 2020, and 25.9% (n = 14) were people whose functional capacity had worsened. The remaining 13% (n = 7) were individuals who maintained the same Barthel index score during lockdown.

The analysis of functional change within the same individual with respect to the two previous Barthel Index assessments, pre- and post-lockdown, using the Wilcoxon signed-rank test showed that the pre-lockdown assessment (43.28 ± 19.017; range 0–60, median 50) and the one conducted in 2023 (48.52 ± 21.884; range 0–95, median 50), the differences were not significant (z = −0.832; p = 0.405). However, when the post-lockdown values (54.41 ± 26.115; range 0–95, median 60) were compared with those observed in 2023 (48.52 ± 21.884; range 0–95, median 50), these differences were indeed significant (z = −4.454; p < 0.001).

Expanding the scope of analysis, when comparing the Barthel Index scores obtained before the lockdown with those obtained in 2023, only 33.3% (n = 18) had seen their scores worsen during this time period, while 16.7% (n = 9) maintained the same score as before the lockdown, and 50% (n = 27) maintained an improvement in their functional capacity score in 2023 compared to their pre-lockdown score. In other words, compared to pre-lockdown values, half of the individuals who were still alive and living in the community in 2023 were those who had improved their functional capacity and maintained that improvement. When the mean Barthel Index scores from before the lockdown and 2023 were compared, the differences were not significant (t = 0.440; p = 0.662; CI -4.881–7.622). No association was observed between these two variables when they were compared using Pearson’s correlation coefficient (r = 0.234; p = 0.089).

However, using the score obtained after the COVID-19 pandemic lockdown as a baseline, 70.4% (n = 38) of the individuals in this 2023 cohort saw their scores worsen over this 3-year period, 7.4% (n = 4) maintained the same score, and 22.2% (n = 12) had a higher Barthel Index score in 2023 than the one observed after the lockdown. In this case, the difference in means was significant (t = 5.123; p < 0.001; CI 8.620–19.713), and Pearson’s correlation coefficient revealed a strong association between them (r = 0.612; p < 0.001).

When post-pandemic data were compared with those obtained in 2023, one in five people (22.2%; n = 12) remained outside the functional dependence criteria of our healthcare system’s protocol (Barthel >60). However, when this comparison was made with the pre-pandemic baseline data, half of the people in the 2023 cohort continued to show functional improvement.

At the same time, monitoring the intensity of the response in terms of scores following the lockdown revealed that, among those who did not meet the criteria for functional dependence according to our protocol in 2023 (Barthel >60) and had improved their scores following the lockdown (n = 11), 72.7% (n = 8) were individuals whose scores had improved by between 25 and 95 points. They also constituted the majority (55.6%; n = 10) among those who, after improving their scores following the lockdown, had moderate dependence in 2023 (n = 18). However, among individuals with severe dependence (n = 4), 50% improved their functional capacity by 0–20 points, and the remaining 50% by 25–95 points.

However, among those whose functional capacity worsened during the lockdown (n = 15 in the 2023 cohort) and who continued to live in the community, 50% (n = 7) had severe functional dependence and 50% (n = 7) had moderate functional dependence.

In 2023, 91.7% of the individuals who continued to fail to meet the criteria for functional dependence according to the local protocol (Barthel >60; n = 12) and who continued to live in the community were individuals whose functional capacity had improved by between 25 and 95 points after the lockdown.

All of these results ultimately led to the formation of a cohort in 2023 in which 25.9% (n = 14) of the participants had severe functional dependence, 51.9% (n = 28) had moderate functional dependence, and 22.2% (n = 12) continued to fail to meet the local protocol criterion for functional dependence (Barthel >60), a status achieved following the lockdown due to the COVID-19 pandemic.

In summary, among the individuals who continued to live in the community, their functional trajectories followed different paths depending on their level of functional dependence prior to lockdown and the changes observed in their functional capacity after lockdown and during the 3-year follow-up. When this developmental timeline was shown graphically (Figure 2, panels one and 2), a worsening of functional capacity after the lockdown made it more likely to continue to maintain long-term functional dependence (Figure 2, panels 1A and 1B), whereas an improvement in functional capacity during lockdown made it more likely to have better functional capacity at the 3-year follow-up and to remain without functional dependence according to the local protocol in the long term (Barthel >60, Figure 2, panel 1C). In 2023, 91.7% of the individuals who remain without functional dependence according to the local protocol (n = 12) and continued to live in the community were those whose functional capacity had improved by between 25 and 95 points following the lockdown.

**FIGURE 2:**
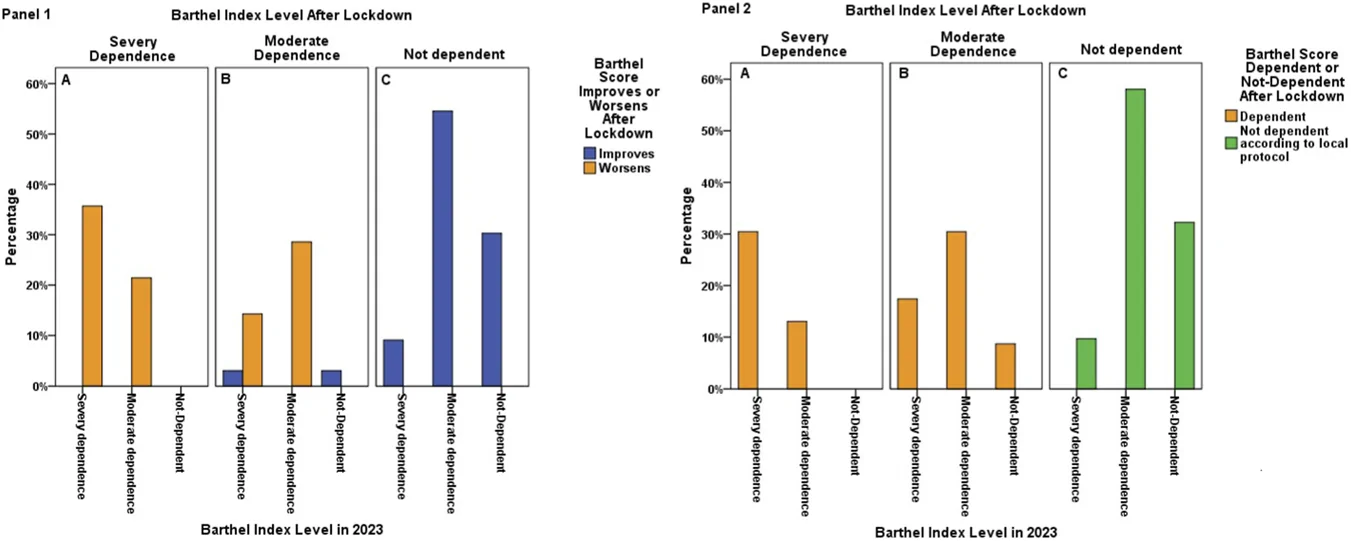
Changes observed in functional capacity during the COVID-19 pandemic lockdown and their impact on the level of functional dependence. The impact of improved or worsened functional capacity after lockdown (panel 1), or of being dependent or non-dependent after lockdown (panel 2), on the level of functional dependence observed after lockdown and at 3 years, shown for the three levels of functional capacity: severe dependence (panel A, Barthel <40), moderate dependence (Barthel 40–60, Panel B), and not dependency according to the local protocol (Barthel >60, Panel C).

Similar results were observed when we analyzed how long-term functional dependence was influenced by showing such a strong functional recovery after lockdown that the individual no longer meet the local protocol’s criteria for functional dependence, compared to those who remained functionally dependent after lockdown (Figure 2, panel 2). The vast majority of individuals who remained dependent after the lockdown were still dependent 3 years later (Figure 2, panels 1A and 1B). Achieving after the lockdown a status of no longer meet the local protocol criterion for functional dependence (Barthel >60) was associated with a better long-term functional profile (Figure 2, panel 2C).

Finally, with regard to survival, when comparing the groups living in the community with those who had died, whether functional capacity improved or worsened after lockdown (χ^2^ = 1.136; p = 0.286), or the intensity of that improvement (χ^2^ = 1.795; p = 0.180), did not influence survival. However, the level of that functional improvement did (χ^2^ = 10.617; p = 0.001), with 73.1% (n = 38) of those who had died up to 2023 remaining dependent after the lockdown, whereas among those who had not died and were still living in the community, this percentage was 42.4% (n = 25). Of the overall cohort in 2023 (n = 54), 37.7% (n = 23) of those who remained functionally dependent and 68.9% (n = 31) of those who no longer meet the local protocol criterion for functional dependence (Barthel >60), were still alive.

When comparing the groups living in a nursing home or who had died, the results regarding improvement versus deterioration and the intensity of improvement in terms of Barthel Index scores were similar. And although among those residing in a nursing home, 56.3% (n = 9) were individuals who, after the lockdown, continued to have functional dependence, and among those who had died, this percentage was 73.1%, the differences were not significant (χ^2^ = 1.623; p = 0.203; Fisher’s exact test 0.228).

## 7 Survival analysis at 5 years of follow-up

When the mean pre (t = 4.161; p < 0.001; CI 7.118–20.029) and post-lockdown scores (t = 5.161; p < 0.001; CI 13.806–30.982) for individuals who were still alive at 5 years with those who had died during that period, the differences were significant. A lower score on the Barthel Index both pre-lockdown (Mann-Whitney U = 1223.500; Z = −3.481; p < 0.001) and post-lockdown (Mann-Whitney U = 972.000; Z = −4.662; p < 0.001) were associated with a lower probability of survival at 5 years.

In this study, the primary outcome measure was time to death; the dependent variable the status of survival or death at 5 years; and the primary covariate the status of “improvement or worsening of the Barthel Index score following the COVID-19 pandemic lockdown,” compared to pre-lockdown values. Within the category of improvement in functional ability, this change could be clinically and/or socially relevant; therefore, the outcome of improved functional capacity was assessed using two covariates: 1: The degree of improvement in the Barthel Index score, comparing an increase from 5 to 20 points versus an increase from 25 to 95 points. And 2: The change in the level of functional dependence reflected by the improvement in the Barthel Index score, comparing maintaining functional dependence according to our healthcare system’s protocol (Barthel <60) versus no longer meeting the criteria for functional dependence according to that protocol (Barthel >60) and referring to the covariate as “functional dependence or has no functional dependency according the local protocol criterion for functional dependence (Barthel >60).”

Survival analysis was performed using Cox regression, adjusted for baseline severity, as represented by the level of functional dependence prior to lockdown estimated using the Barthel Index, which showed different results for these three covariates. Although an improvement or worsening in the Barthel Index score did not imply a higher or lower risk of 5-year mortality in this cohort (HR 0.702; CI 0.427–1.155), achieving an improvement in functional capacity of less than 25 points—in contrast to achieving an improvement in the Barthel Index score of 25 points or more—was associated with a higher risk of 5-year mortality (HR 2.295; CI 1.213–4.343), and maintaining functional dependence after lockdown according to our healthcare system’s protocol was also associated with an increased risk of mortality (HR 1.695; CI 1.018–2.823). The complete results of these analyses are presented in Supplementary Appendix 4, “Cox Regression and Descriptive Analyses Related to Survival,” Section 1: “Data for Each Covariate Using Cox Regression.”

Further analysis (Supplementary Appendix 4, Section 2: Descriptive Analyses Regarding Survival, Supplementary Tables SE1–SE3) revealed that the differences in mean survival times between those whose functional capacity improved (40.61 ± 21.959 months; CI 35.69–45.53) or whose functional capacity worsened (35.24 ± 20.861 months; CI 27.85–42.64) after confinement (Supplementary Table SE1) were not significant (Mann-Whitney U = 1122.000; Z = −1.193, p = 0.233). Nor were the differences between the mean survival times statistically significant (Mann-Whitney U 612.000; Z = −1.717, p = 0.086) when, after confinement, the improvement in functional capacity was between 5 and 20 points (36.51 ± 22.136 months; CI 29.34–43.69) or between 25 and 95 points (44.60 ± 21.305 months; CI 37.79–51.41) (Supplementary Table SE2). However, the differences in mean survival times were significant (Mann-Whitney U = 1373.000; Z = −3.042, p = 0.002) between those who remained dependent after the lockdown (34.13 ± 21.618; CI 29.04–39.21) and those who, after lockdown, were classified as not functionally dependent according to the local protocol because they had a Barthel Index score greater than 60 (45.44 ± 20.551; CI 39.88–50.99) (Supplementary Table SE3).

Within this result, 57% (n = 45) of the people who improved their functional capacity during the COVID-19 pandemic lockdown (n = 79) died during this 5-year period. Among the people whose functional capacity worsened during lockdown (n = 33), 72.7% (n = 24) died during this period (Figure 3). Despite these differences in percentages, statistical significance was not achieved (χ^2^ = 2.446; p = 0.118).

**FIGURE 3:**
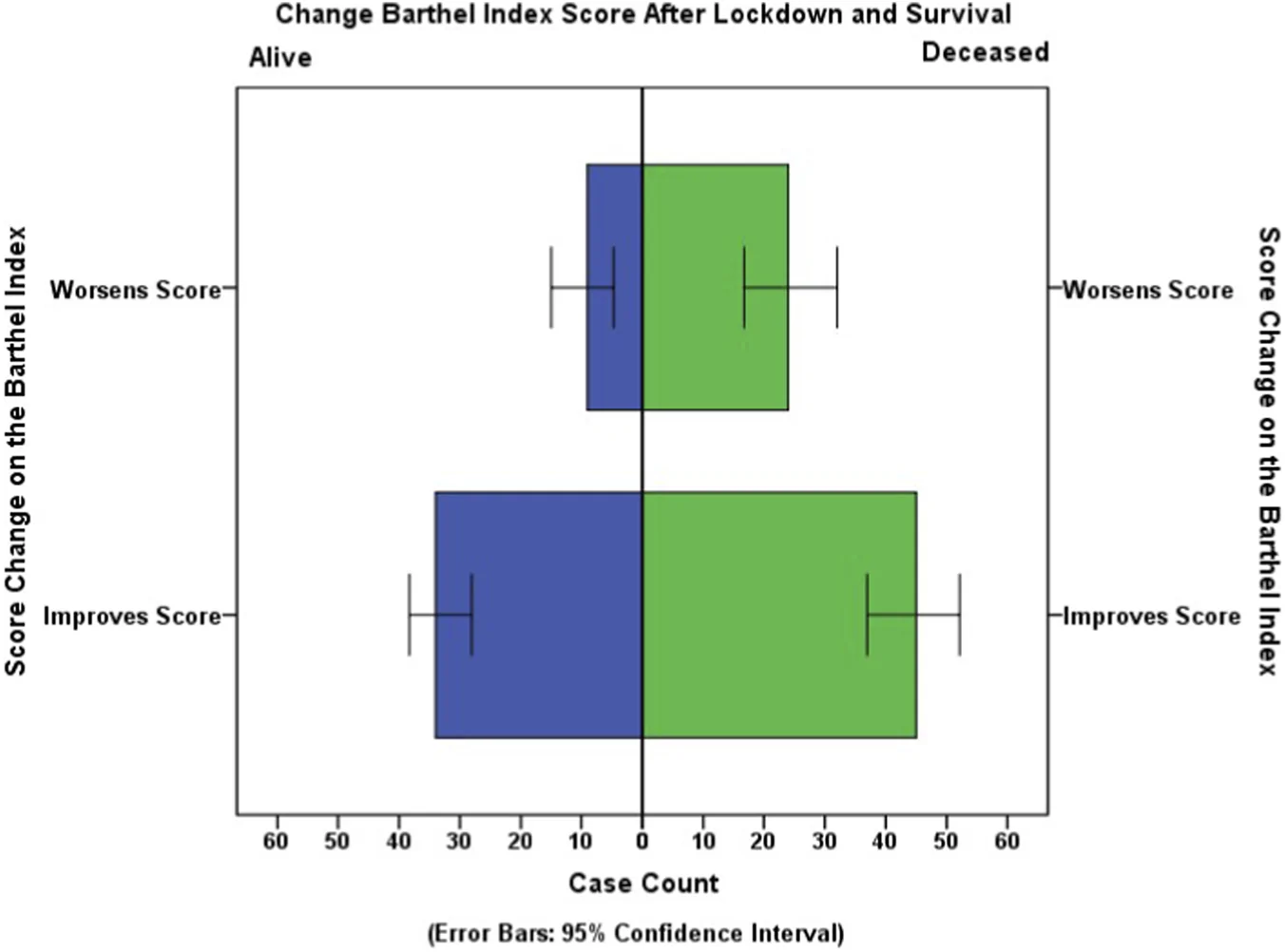
Changes in Barthel Index scores during lockdown and 5-year survival. Results relating to improved or worsened scores compared to pre-lockdown Barthel Index scores.

Nor was there a statistically significant association between achieving a clinically relevant improvement in the Barthel Index score (25–95 points) and greater 5-year survival (χ^2^ = 2.959; p = 0.085). During this period, 66.7% (n = 26) of the individuals who improved by 5–20 points (n = 39) died, as did 47.5% (n = 19) of the individuals whose improvement in functional ability, as estimated by the Barthel Index, ranged from 25 to 95 points (n = 40).

However, among those who, after lockdown, were no longer considered functionally dependent according to local protocol (n = 55), 45.5% (n = 25) had died within 5 years, compared with 73.6% (n = 53) among those who remained functionally dependent after the lockdown (n = 72) (χ^2^ = 10.432; p = 0.001). The probability of death was higher in the group of individuals who remained functionally dependent after the lockdown (OR 3.347; CI 1.587–7.057). This last result included individuals whose functional capacity worsened, remained the same, or improved while still meeting the criteria for functional dependence defined in our local protocol. It was observed that, among those who had a moderate level of functional dependence after the lockdown (30.7%, n = 39), 61.5% (n = 24) died, while among those with a severe level of functional dependence (26%; n = 33), 87.9% (n = 29) died. These results are shown in Figure 4, which also includes mortality in the group that, after the lockdown, improved their functional capacity to a level of not being functionally dependent according to the local protocol (45.5%; n = 25).

**FIGURE 4:**
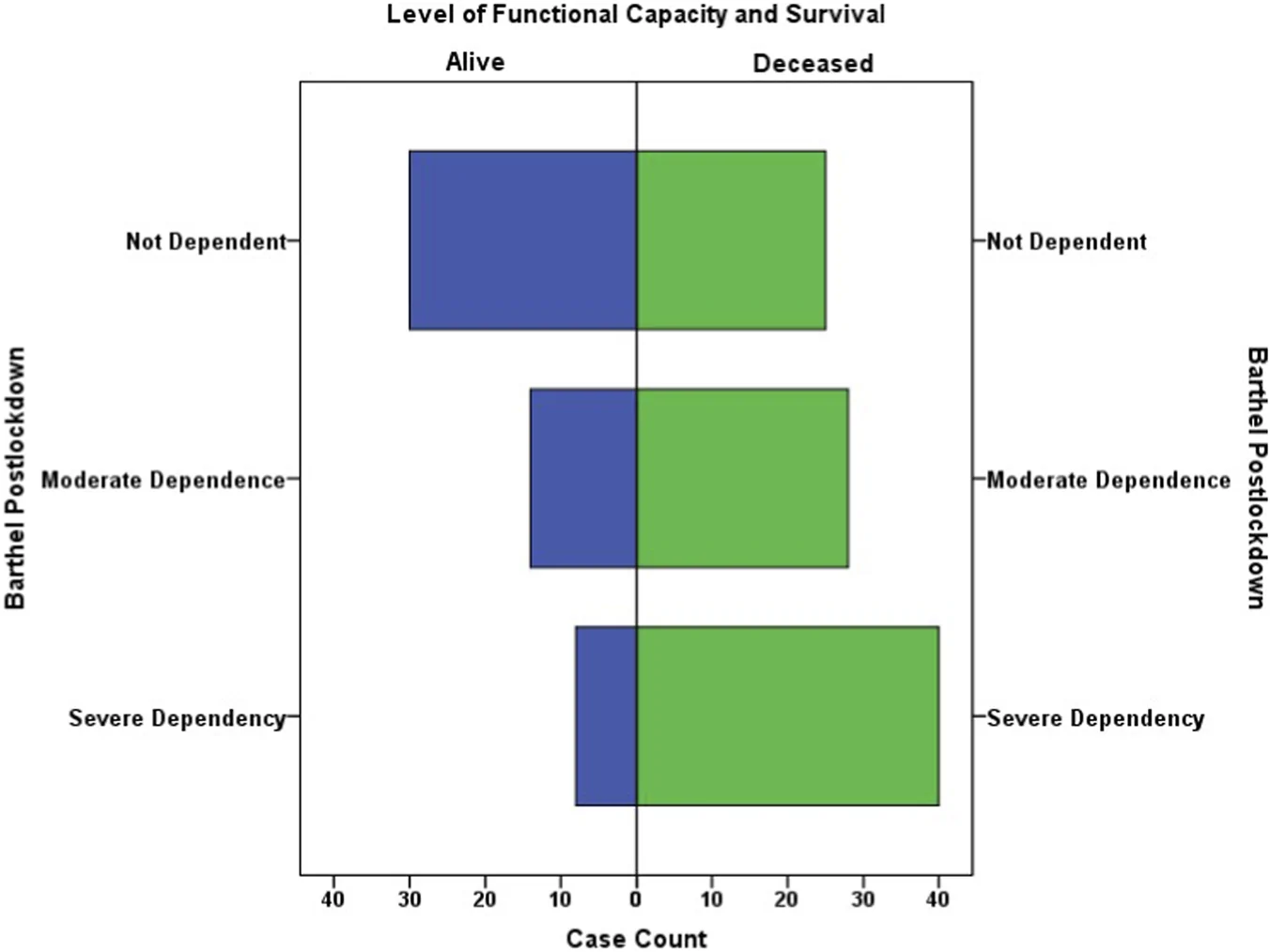
Changes observed in the level of functional dependence following the COVID-19 pandemic lockdown and 5-year survival. Not-dependent: After the lockdown, the patient has a Barthel Index score greater than 60, the cutoff point established in our healthcare system’s protocol for classification as a person with functional dependence. Moderate dependency: Barthel Index score of 40–60. Severe dependency: Barthel Index score of 0–35.

When survival was analyzed specifically within the subgroup of individuals who, after lockdown, improved their functional capacity but did not cease to be dependent (30.4%; n = 24), it was observed that 83.3% (n = 20) of the members of this subgroup died, whereas among those who no longer meet the criteria for functional dependence established in the local protocol, 45.5% (n = 25) died. Within the subgroup that improved their functional capacity after the lockdown, not improving to a level that allowed them to no longer be included in the local functional dependency protocol was associated with a higher probability of death (OR 0.167; CI 0.050–0.552). This result contrasted with that observed among the population whose functional capacity worsened after lockdown, which had a lower percentage of deaths (72.7%; n = 24) than the subgroup that showed moderate improvement in functional capacity, although in this case the differences were not statistically significant (OR 1.875; CI 0.501–7.010).

On the other hand, these results may help explain why, in the survival analysis for the 2023–2025 period regarding the level of functional dependence estimated using the Barthel Index, no significant differences were observed in mortality risk based on the level of dependence that these individuals had in 2023 (χ^2^ = 0.911; p = 0.634; Likelihood Ratio 0.628). Most members of the 2023 cohort were individuals who improved their functional capacity following the lockdown, and one in five (22.2%; n = 12) of these individuals also improved their functional capacity to a level of not being functionally dependent according to the local protocol. Between June 2023 and June 2025, 42.9% (n = 6) of the individuals who had severe functional dependence in 2023 (n = 14) died, 35.7% (n = 10) of those who had moderate functional dependence (n = 28), and 25% (n = 3) of those who, in 2023, has no functional dependency according the local protocol.

### 7.1 Development of regression models

In the development of regression models, the number of events (deaths) during the 5-year study period was 78, representing 61.4% of the cohort population. All models developed were adjusted for baseline severity, using the level of functional dependence prior to lockdown as estimated by the Barthel Index.

With regard to the censored cases, after 5 years of follow-up, the final status of the patients fell into one of three categories: 1: Still living in the community (27.6%; n = 35); 2: Living in a nursing home (11%; n = 14); or 3: Deceased (61.4%; n = 78). To handle the censored cases and facilitate the transformation of censoring in independent, these three outcomes were considered by analyzing the correlation between the covariates included in this study that could potentially influence survival and survival itself (Section 3 of Supplementary Appendix 4, Management of Censored Cases). This analysis was conducted by comparing these three outcomes with respect to these covariates. In addition, active follow-up of lost-to-follow-up patients and the recording of the exact reasons for a participant’s withdrawal (admission to a nursing home, voluntary decision, death) also contributed to this independence. The treatment of ties was previously described in the “Survival Analysis” section of the “Materials and Methods” section.

With regard to the covariate “functional dependence or has no functional dependency according the local protocol criterion for functional dependence (Barthel >60),” associated with the survival, the model adjusted for baseline severity which included the covariates “educational level,” “level of intervention assigned by the healthcare system,” and “polypharmacy”—all of which remained statistically significant—and the covariates economic status, risk level, burden of chronic diseases, use of mobility aids, and having or not having a non-family caregiver—which did not remain statistically significant—was the one associated with a higher risk of mortality (HR 1.867; CI 1.052–3.315). The complete results of these analyses are presented in Supplementary Appendix 4, Cox Regression, Section 4: Data associated with the Cox regression for the covariate “functional dependence or has no functional dependency according the local protocol criterion for functional dependence (Barthel >60).” Figure 5 shows the differences in mortality related to this covariate for each baseline severity level.

**FIGURE 5:**
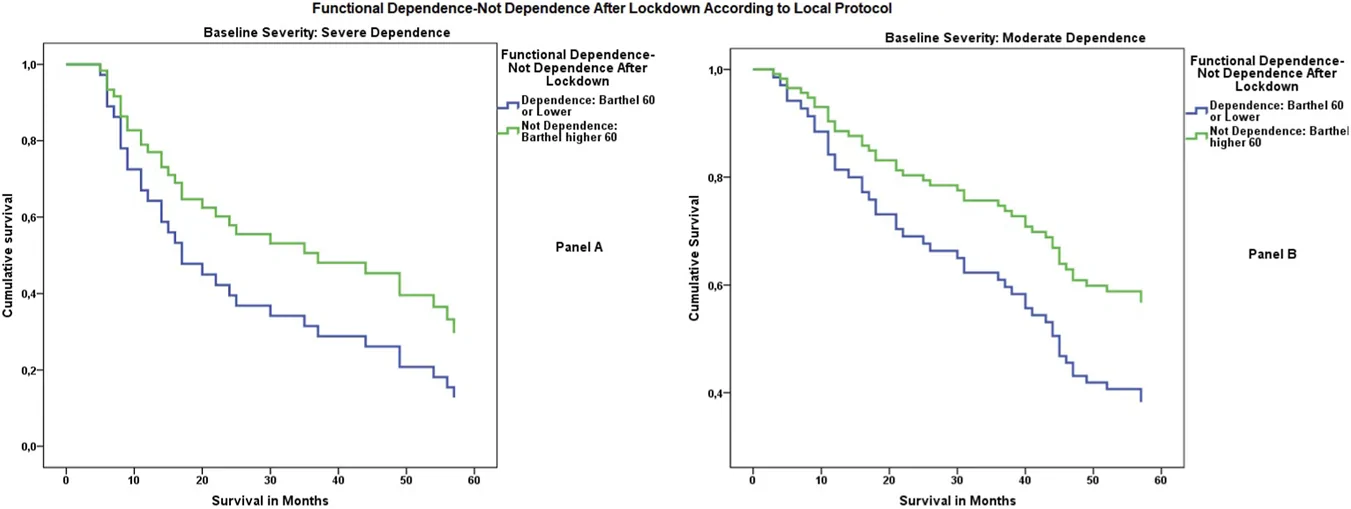
Survival analysis using Cox regression for the covariate “functional dependence or no longer met the local protocol criterion for functional dependence (Barthel >60),” based on baseline severity as determined by the level of functional dependence prior to the lockdown. Panel **(A)** Severe dependence: Barthel 0–35 points. Panel **(B)** Moderate dependence: Barthel 40–60 points. The criterion for functional dependence included in the protocol for caring for people with functional dependence is defined as a Barthel Index score of 60 or lower. The criterion for has no functional dependency used in this study is defined as a Barthel Index score higher than 60; individuals with this score do not receive the personalized care established in the protocol.

Related to the handling of collinearity for this first covariate (Supplementary Appendix 4, Cox Regression, Section 5: Collinearity for the functional dependence-independence covariate after lockdown: Variance Inflation Factor (VIF)), the variance inflation factor for the Barthel Index ranged from 1.000 to 1.017, indicating that no substantial collinearity was detected among the predictors included in the auxiliary analysis. As part of the model verification, the analysis of residues using the Schoenfeld method showed a constant effect of the variable over time for each predictor variable, as well as the absence of correlation between these residues and time (Supplementary Appendix 4, Cox Regression, Section 6: Schoenfeld method for the “functional dependence or has no functional dependency according the local protocol criterion for functional dependence (Barthel >60)” covariate, thereby validating the proportional hazards assumption in the Cox regression.

When interaction terms were included in the Cox model, such as pre-lockdown functional status*sex, pre-lockdown functional status*age, or pre-lockdown functional status*clinical conditions associated with poorer health outcomes (polypharmacy, chronicity burden, risk level, intervention level), no significant changes were observed in the estimated risk of mortality (HR 1.721; CI 1.031–2.872). However, when post-lockdown functional status was used instead of pre-lockdown functional status, the model that included educational level, being a polymedicated patient, post-lockdown functional dependency level*sex, post-lockdown functional dependency level*age, and disease burden*intervention level showed the strongest association between maintaining functional dependency after the lockdown and the risk of mortality (HR 3.143; CI 1.395–7.078) (Supplementary Appendix 4, Cox Regression, Section 7: “Cox regression models incorporating interaction terms.” In this model, the covariate 'functional dependence or has no functional dependency according to the local protocol criterion for functional dependence (Barthel >60)' and educational level remained statistically significant. However, formal interaction testing did not provide statistically significant evidence of effect modification by sex or age; the interaction involving post-lockdown functional dependence and male sex was suggestive but did not reach statistical significance (p = 0.056).

As for the second main covariate associated with survival, “level of improvement in the Barthel Index score after lockdown,” the model adjusted for baseline severity that was associated with a higher risk of mortality (HR 2.824; CI 1.458–5.467) was the one that included the covariates “level of intervention” and “sex,” which remained statistically significant, and the covariates disease burden, risk level, polypharmacy, educational level, use of mobility aids, and having or not having a non-family caregiver, which did not reach statistical significance in this model. The complete results of these analyses are presented in Supplementary Appendix 4, Cox Regression, Section 8: Data associated with the Cox regression for the covariate “level of improvement in the Barthel Index score after lockdown.” Figure 6 shows the differences in mortality related to this covariate for each baseline severity level.

**FIGURE 6:**
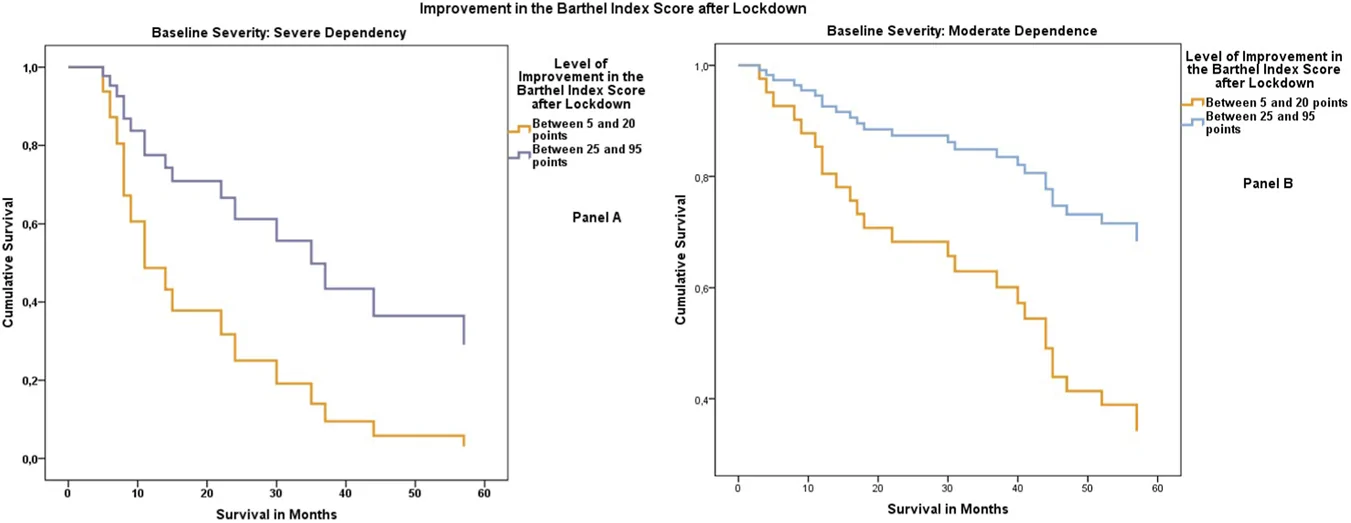
Survival analysis using Cox regression for the covariate “level of improvement in the Barthel Index score after the COVID-19 pandemic lockdown,” based on baseline severity as determined by the level of functional dependence prior to the lockdown. Panel **(A)** Severe dependence: Barthel 0–35 points. Panel **(B)** Moderate dependence: Barthel 40–60 points. The criterion for functional dependence included in the protocol for caring for people with functional dependence is defined as a Barthel Index score of 60 or lower. The criterion for functional independence used in this study is defined as a Barthel Index score higher than 60; individuals with this score do not receive the personalized care established in the protocol.

For this second main covariate, and regarding the handling of collinearity, the variance inflation factor (VIF) for the Barthel Index ranged from 1.000 to 1.007, also indicating that no substantial collinearity was detected among the predictors included in the auxiliary analysis (Supplementary Appendix 4, Cox Regression, Section 9: Collinearity for the covariate “level of improvement in the Barthel Index score after lockdown”: Variance Inflation Factor (VIF)). As part of the model verification, the Schoenfeld test of residues showed a constant effect of the variable over time for each predictor variable, and the absence of correlation between these residues and time (Supplementary Appendix 4, Cox Regression, Section 10: Schoenfeld method for the covariate “level of improvement in the Barthel Index score after lockdown”), thereby also validating the proportional hazards assumption in the Cox regression.

Regarding the incremental prognostic value of the functional change observed after lockdown, when a regression analysis was performed using a generalized linear model and the four clinical covariates relevant to the assessment of the risk of adverse events (risk level, intervention level, chronicity burden, and polypharmacy), age, sex, and the six socially relevant variables (educational level, socioeconomic status, having a caregiver, using a mobility aid, being married, being widowed, and living with children or other people) were incorporated into the model, the AIC values were 149.609 for the clinical-social baseline model, 140.264 for the baseline model, and 132.297 for the incremental model in June–July 2020; the corresponding BIC values were 188.865, 182.324, and 174.358, respectively. The difference in AIC between the pre-lockdown baseline model and the post-lockdown incremental model was 7.967, but compared to the clinical-social baseline model, the difference was 17.312—well above 10. The complete results of these analyses are presented in Supplementary Appendix 4, Cox Regression, Section 11: “Incremental Prognostic Value of Functional Change”, subsection A: “Generalized linear regression models including the clinical and social covariates analyzed in this study.”

Finally, the area under the ROC curve observed for the incremental model (0.921; p < 0.001; CI 0.876–0.967), which included the covariates analyzed in this study and the post-lockdown Barthel Index score, was greater than that observed for the baseline model (0.846; p < 0.001; CI 0.778–0.913), which included those same covariates and the baseline Barthel Index score obtained before lockdown (Figure 7). The complete results of these analyses are presented in Supplementary Appendix 4, Cox Regression, Section 11, subsection B: “Analysis of the incremental predictive value of the change in functional status observed after lockdown using the area under the ROC curve.”

**FIGURE 7:**
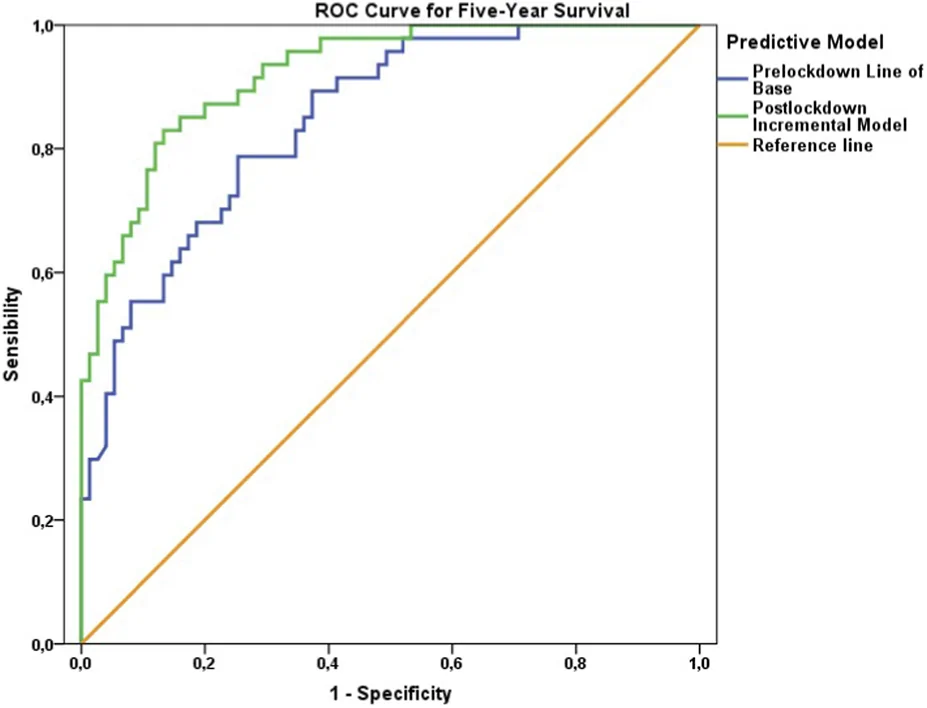
Analysis of the incremental predictive value of the change in functional status observed after lockdown using the area under the ROC curve. Comparison of the baseline pre-lockdown model, which includes the variables analyzed in this study and the individual pre-lockdown Barthel Index score, and the incremental model, which adds to the baseline model the individual differences in Barthel Index scores observed after the lockdown.

During the first wave of the COVID-19 pandemic in Spain, 2.7% (n = 4) of the individuals in this cohort died. These deaths occurred before fieldwork and the follow-up period began; therefore, this subgroup of the population was unable to participate in the study. The study population therefore comprises individuals with functional dependence in the Orcasitas neighborhood who completed the window period corresponding to the lockdown, did not die during that period, and did not meet any exclusion criteria. The follow-up period for this cohort began on the day the participants signed the informed consent form and underwent the survey and physical examination. Consequently, their enrollment took place between June 22 and 10 July 2020. As a result, the maximum differences among patients regarding the follow-up period until the event or 5-year survival are 19 days.

However, an assessment was conducted to determine whether this percentage of deaths was representatively higher than what would have been expected for the cohort during long-term follow-up. As part of this analysis, the four individuals mentioned above died during the period from March 1 to 30 June 2020. Using a similar 4-month comparison period following the end of the first wave, two people from this cohort died over the next 4 months. However, in the subsequent 4-month period, between 1 November 2020, and 28 February 2021, 12 people died, and between March 1 and 30 June 2021 —a seasonal period with characteristics similar to those of the first wave— 12 people also died. Based on these results, it cannot be concluded that the percentage of deaths during the first wave of the COVID-19 pandemic differed from that observed in this cohort over time.

## 8 Analysis of confounding, concurrent, and mediating factors. associations and exploratory analyses that generate hypotheses

Functional responsiveness during lockdown and survival could be influenced by multiple factors which were analyzed.

### 8.1 Confounding factors

#### 8.1.1 Age as a factor modulating the intensity of functional response and survival

With regard to the outcome or dependent variable—five-year survival—in this study, older age was associated with a lower probability of survival (Mann-Whitney U = 1395.000; Z = −2.561; p = 0.010), and, after adjusting for baseline severity, it showed to be an independent risk factor for mortality (HR 0.590; CI 0.372–0.934; p = 0.025) (Figures 8 and 9, and Module one of Supplementary Appendix 5, Data Behind the Figures).

**FIGURE 8:**
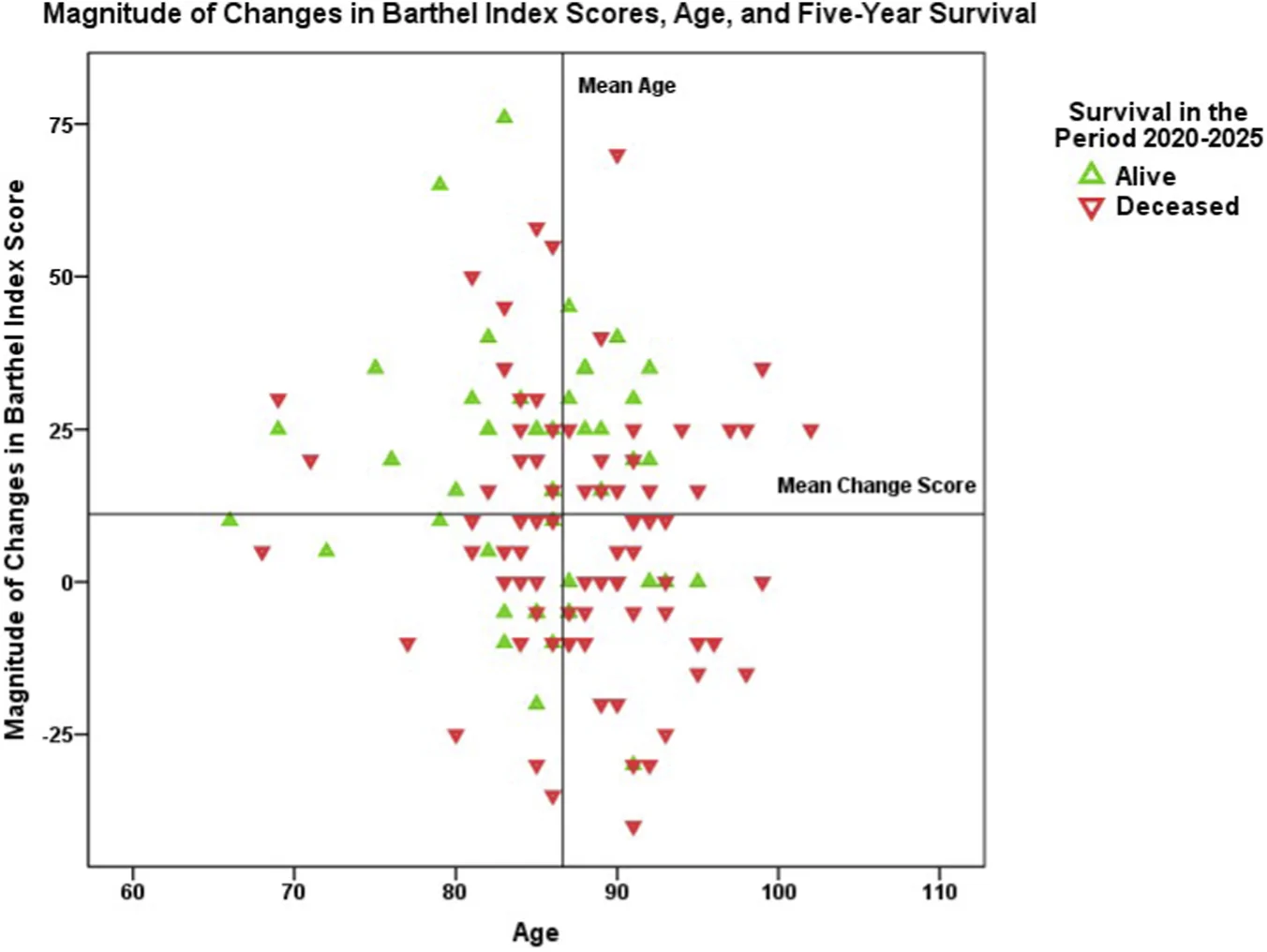
Effect of age and the magnitude of changes observed in the Barthel Index score after lockdown on 5-year survival, relative to baseline values prior to lockdown.

**FIGURE 9:**
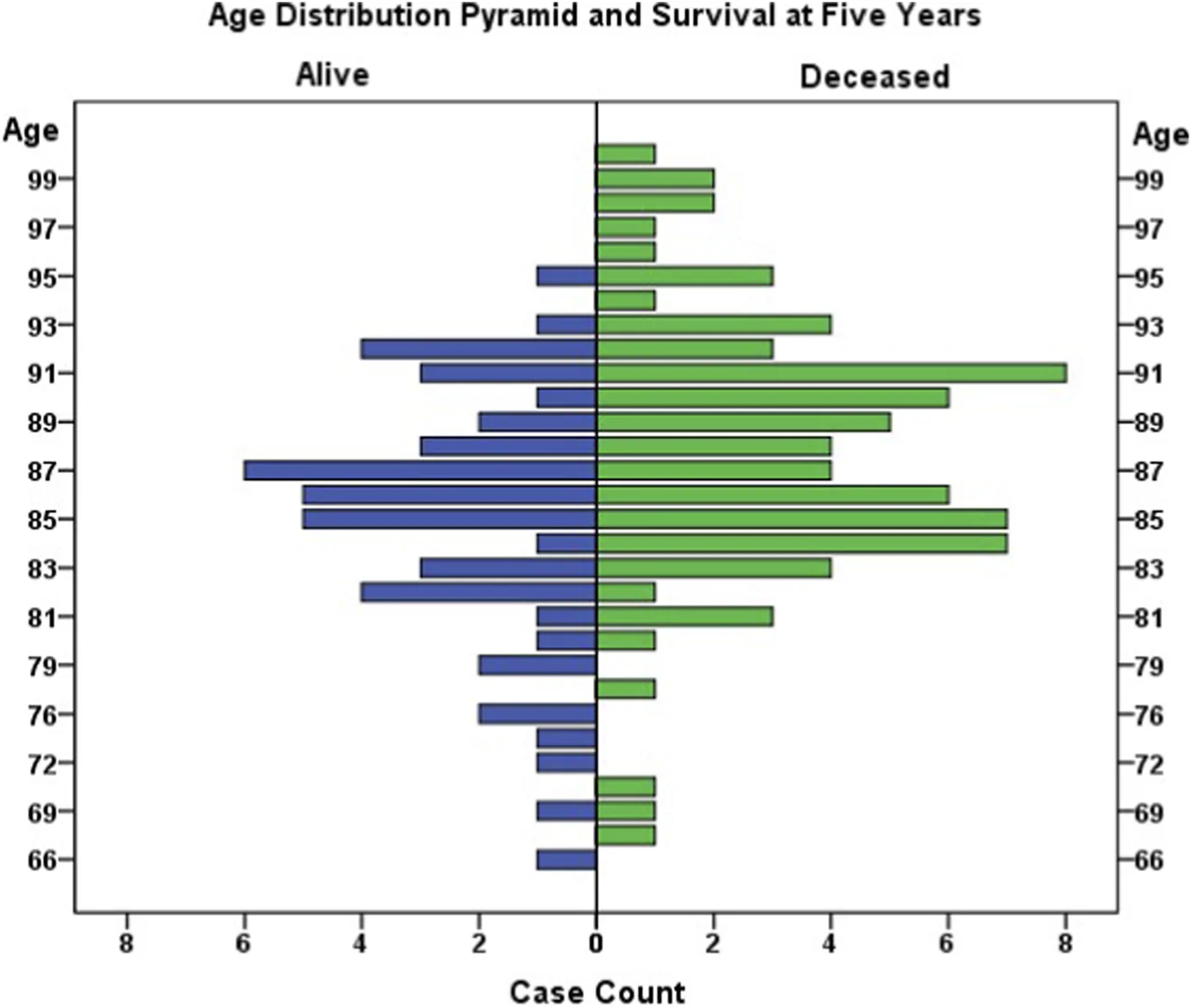
Age-specific population pyramid at the start of the study and survival-mortality after 5 years of follow-up.

When analyzing its potential influence on the results obtained after lockdown in relation to functional capacity, in this study age was not associated with whether or not there was an improvement in functional capacity (Mann-Whitney U = 1000.000; Z = −1.941; p = 0.052) (Figure 10), nor with the intensity of the observed functional response (Mann-Whitney U = 745.000; Z = −0.344; p = 0.731).

**FIGURE 10:**
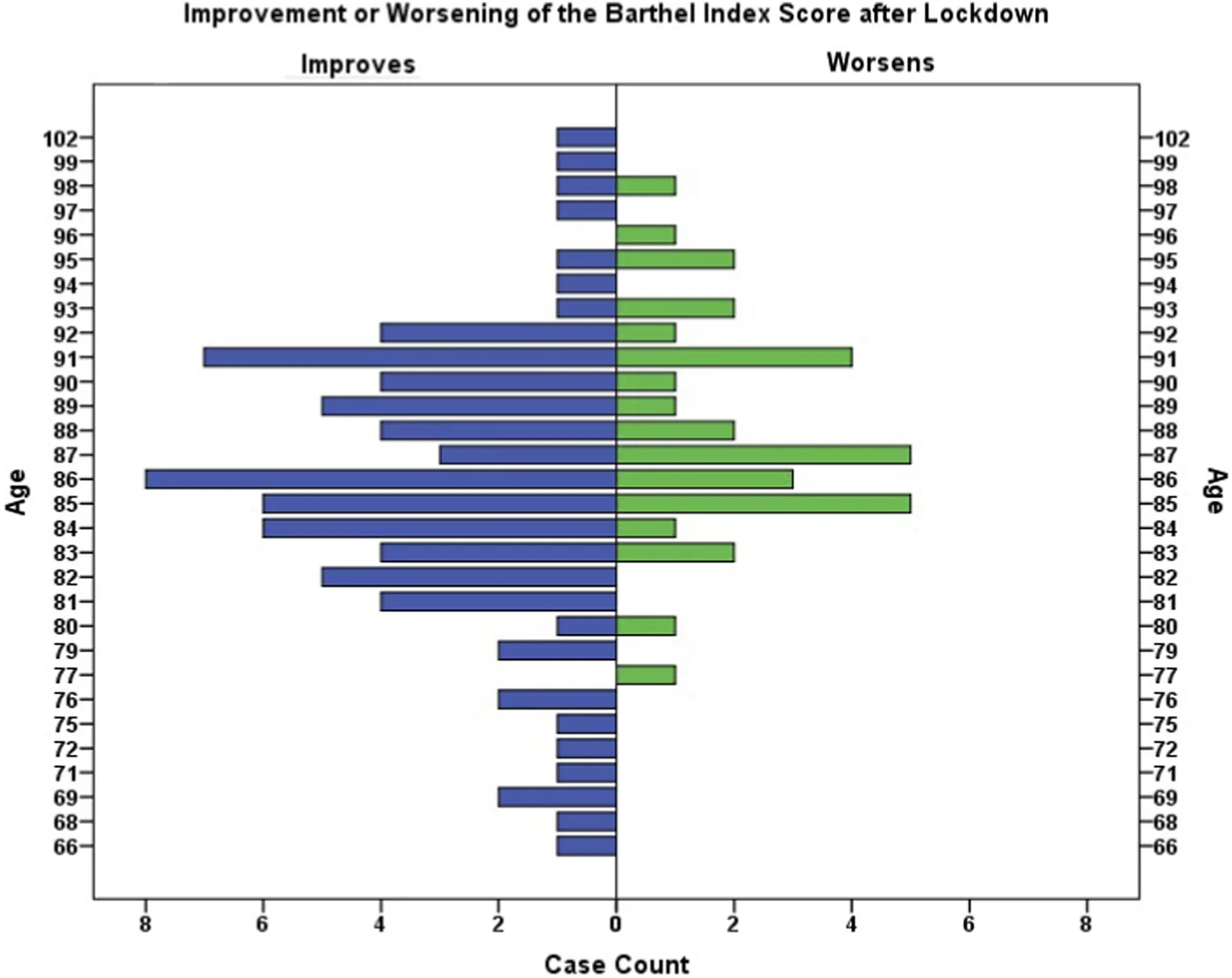
Age and changes observed in functional capacity after lockdown in the usual home due to the COVID-19 pandemic, estimated using the Barthel Index score. Improves: improvement in the Barthel Index score compared to the pre-lockdown baseline value. Worsens: worsening of the Barthel Index score compared to the pre-lockdown baseline value.

However, it did show a significant difference (Mann-Whitney U = 1568.000; Z = −2.009; p = 0.045) regarding whether this improvement in functional capacity reached a level that would allow the patient to no longer meet the criteria for functional dependence established in the local protocol. Upon analyzing this result, among those who remained functionally dependent following the COVID-19 pandemic lockdown, the mean age was 87 years (87.39 ± 6.864; 95% CI 85.78–89.00, range 68–102, interquartile range 7), whereas, among those who scored above 60 on the Barthel Index, the mean age was 85 years (85.64 ± 5.386; CI 84.18–87.09; range 66–97; interquartile range 7).

Most people who made such a significant functional change were between 79 and 92 years old, thus including people of advanced age. Younger people and those of very advanced age (over 92 years old) had greater difficulty translating this improvement into a level of not being functionally dependent according to the local protocol (Figure 11 and Module one of Supplementary Appendix 5, Data Behind the Figures).

**FIGURE 11:**
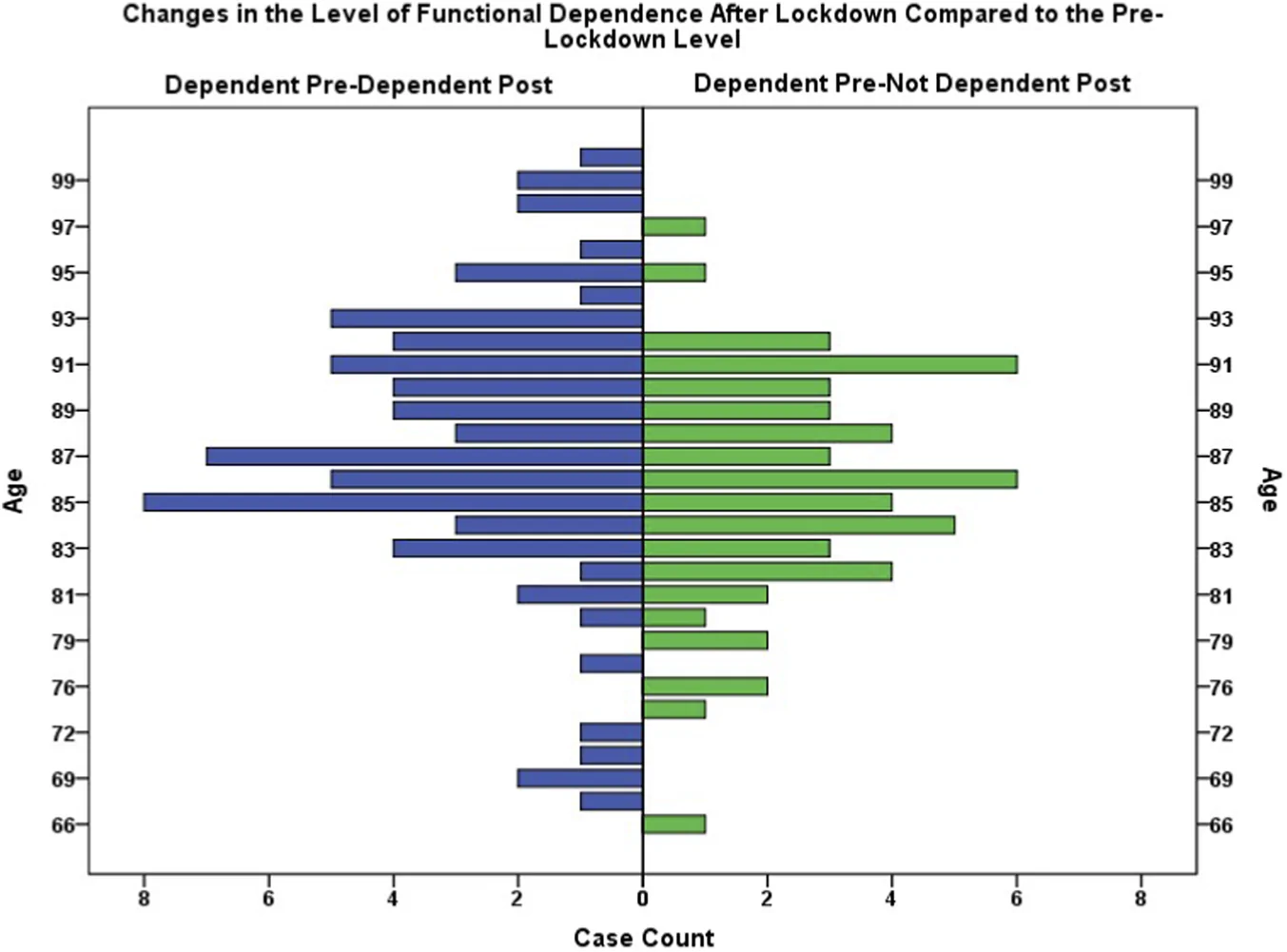
Age and change in the level of functional dependence after lockdown, compared to the pre-lockdown level of functional dependence, both estimated using the Barthel Index. Dependent Pre-Dependent Post: The individual is functionally dependent before lockdown, having a Barthel Index score of 60 or lower, and, in the assessment conducted after lockdown, continues to meet the criteria for functional dependence. Dependent Pre-Not Dependent Post: The individual is functionally dependent before lockdown, having a Barthel Index score of 60 or lower, and in the post-lockdown assessment, achieves a Barthel Index score >60, no longer meeting the criteria for functional dependence according to the protocol and is therefore considered not dependent in our care context.

To complement these analyses regarding survival, when these results were disaggregated according to whether participants remained dependent after lockdown or had improved their functional capacity to a level where they were no longer considered functionally dependent according to the local protocol, age continued to be associated with mortality in the group of individuals who, after lockdown, no longer met the local protocol’s criteria for functional dependence (Mann-Whitney U = 258.000; Z = −1.983; p = 0.047), but this association was not observed in the group that remained functionally dependent after lockdown (Mann-Whitney U = 407.500; Z = −1.229; p = 0.219) (Figure 12).

**FIGURE 12:**
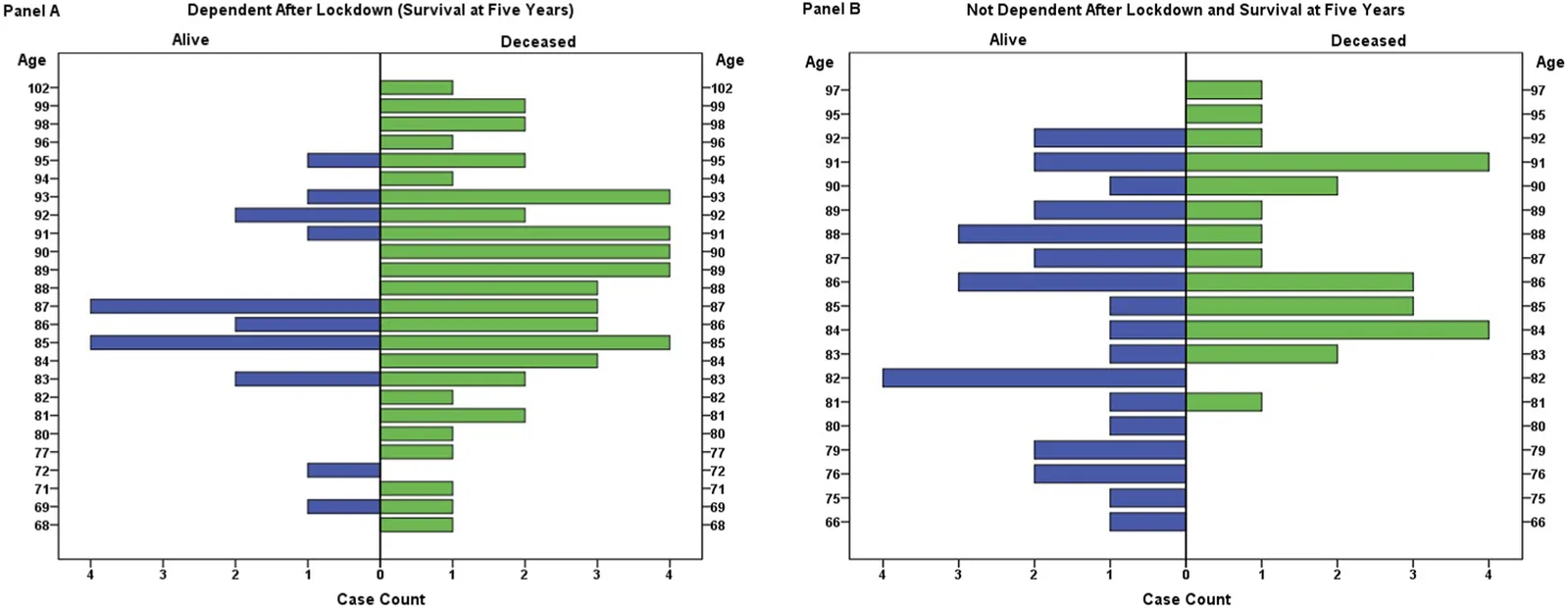
Five-year survival-mortality population pyramids by age and functional response with respect to the level of dependency observed after lockdown. Panel **(A)** Individuals who, after the COVID-19 lockdown, remained functionally dependent (Barthel score of 60 or lower). Panel **(B)** Individuals who, after lockdown, improved their functional capacity to a score above 60 on the Barthel Index, exceeding the threshold established in the protocol for the care and follow-up of individuals with functional dependence, and are therefore considered not functionally dependent.

Finally, among those who remained functionally dependent after lockdown, 91.2% (n = 31) of those with severe dependency (n = 34) and 57.9% (n = 22) of those with moderate dependency (n = 38) died (OR 0.133; CI 0.035–0.513). However, among patients who improved their functional capacity after the lockdown and no longer meet the local protocol criterion for functional dependence (Barthel >60), a lower score on this index remained associated with survival (Mann-Whitney U = 238.500; Z = −2.345; p = 0.019).

With regard to housing situations, when the age factor was analyzed in relation to whether individuals lived with their children or independently, it was observed that the mean age of dependent individuals living with their children (88.35 ± 6.382; CI 86.66–90.04) was higher than that observed among those living independently in the community (84.09 ± 5.800; CI 82.38–85.79), and this difference was statistically significant (Mann-Whitney U = 789.000; Z = −3.602; p < 0.001).

#### 8.1.2 Sex

Although this study found that, after 5 years of follow-up, 58% (n = 58) of the women (n = 100) and 74.1% (n = 20) of the men (n = 27) had died, the differences were not statistically significant (χ^2^ = 2.318; p = 0.128; Cramer’s V = 0.125; p = 0.128). However, when the analysis was adjusted for baseline severity, being male was an independent risk factor for mortality (HR 0.593; CI 0.355–0.989; p = 0.045). Expanding this analysis, the mean Barthel Index score for deceased men was 39.25 ± 21.230, with a confidence interval of 29.31–49.19, whereas among deceased women, the means of the Barthel Index scores was 37.62 ± 21.158, with a confidence interval of 32.06–43.18. This result prompted a differentiated analysis by levels of functional dependence, revealing that among deceased men with severe dependence, the means of the Barthel Index scores was 13.57 ± 11.073, with a confidence interval of 3.33–23.81, whereas among women with severe functional dependence who had died during this 5-year period, the mean Barthel Index score was 27.80 ± 10.803, with a confidence interval of 14.39–41.21.

Sex did not influence whether functional capacity improved or worsened (χ^2^ = 0.001; p = 0.971; Cramer’s V = 0.003; p = 0.971) (Supplementary Table S1 in Section 1: Analysis of confounding factors, included in Module 2 of Supplementary Appendix 5), the intensity of the functional improvement observed in terms of Barthel Index scores (χ^2^ = 0.581; p = 0.446; Cramer’s V = 0.086; p = 0.446) (Supplementary Table S2 in Section 1: Analysis of confounding factors, included in Module 2 of Supplementary Appendix 5), or on whether this improvement allowed patients to achieve the level of not being functionally dependent according to the local protocol (χ^2^ = 0.018; p = 0.893; Cramer’s V = 0.012; p = 0.893) (Table 3).

**TABLE 3:** Observed associations between the confounding and concurrent-mediating factors analyzed in this study and functional capacity assessed after the Covid-19 pandemic lockdown using the Barthel Index.

Changes to the level of functional dependency during nationwide COVID-19 lockdown				
Social factor	Status	Barthel level after the lockdown^a^		
		Remains functionally dependent	No dependent according the local protocol	Odds ratio (95% confidence Interval)^b^
Confounding factors				
Sex (n = 127)	Male	55.6% (15)	44.4% (12)	1.060 (0.450–2.496)
	Female	57% (57)	43% (43)	
Educational level^c^ (n = 126)	Not education	54.1% (60)	45.9% (51)	0.428 (0.128–1.426)
	Had education	73.3% (11)	26.7% (4)	
Economic level (n = 127)	<11.200 euros/year	61.7% (37)	38.3% (23)	1.471 (0.725–2.984)
	>11.200 euros/year	52.2% (35)	47.8% (32)	
Risk level (n = 127)	High	53% (35)	47% (31)	0.732 (0.362–1.483)
	Low or medium	60.7% (37)	39.3% (24)	
Intervention level (n = 127)	High	58% (29)	42% (21)	1.092 (0.532–2.242)
	Low or medium	55.8% (43)	44.2% (34)	
Disease burden (n = 127)	≥ 5 chronic diseases	55.9% (19)	44.1% (15)	1.046 (0.474–2.309)
	< 5 chronic diseases	57% (53)	43% (40)	
Drugs (n = 126)	≥ 5 drugs	55.7% (64)	44.3% (51)	0.471 (0.119–1.865)
	< 5 drugs	72.7% (8)	27.3% (3)	
Concurrent and mediating factors				
Marital status married (n = 127)	Married	59% (23)	41% (16)	1.144 (0.533–2.457)
	Not married	55.7% (49)	44.3% (39)	
Marital status widowed (n = 127)	Widowed	53.7% (44)	46.3% (38)	0.703 (0.335–1.477)
	Not widowed	62.2% (28)	37.8% (17)	
Housing situation (n = 127)	Lives independently	40.4% (19)	59.6% (28)	**0.346 (0.164–0.728)**
	Lives with children/Others	66.3% (53)	33.7% (27)	
Community living (n = 127)	Leaves home	50% (41)	50% (41)	**0.452 (0.210–0.971)**
	Homebound	68.9% (31)	31.1% (14)	
Leaves home for walks (n = 121)	Yes	47.2% (34)	52.8% (38)	**0.434 (0.204–0.923)**
	No	67.3% (33)	32.7% (16)	
Shopping independently (n = 123)	Yes	15.8% (3)	84.2% (16)	**0.113 (0.031–0.411)**
	No	62.5% (65)	37.5% (39)	
Shops under supervision (n = 122)	Yes	29.4% (5)	70.6% (12)	**0.289 (0.095–0.880)**
	No	59% (62)	41% (43)	

^a^Levels of dependency analyzed: 1: Remains functionally dependent: Barthel score ≤60 before and after lockdown. 2: No dependent according the local protocol: no longer meet the local protocol criterion for functional dependence (Barthel >60), Barthel score ≤60 before lockdown and greater than 60 after lockdown.

^b^In bold: odds ratios that are statistically significant.

^c^Education level: Had education: having completed elementary school or a higher level of education; Not education: illiterate; able to read and write but having no formal education; and not having completed elementary school.

Within the changes in the level of dependence after the lockdown, when functional dependence persisted, sex also had no effect on survival (χ^2^ = 0.398; p = 0.528, Fisher’s exact test p-value 0.744), with 71.9% (n = 41) of women and 80% (n = 12) of men dying during this period. Similarly, although differences in mortality were observed after the lockdown between men (66.7%; n = 8) and women (39.5%; n = 17) when has no functional dependency according to the local protocol, these differences were not statistically significant (χ^2^ = 2.786; p = 0.095).

#### 8.1.3 Educational level

With regard to educational level, 57.7% (n = 64) of the individuals in this cohort who had no formal education had died within 5 years, compared to 86.7% (n = 13) among those who had received an education (χ^2^ = 4.679; p = 0.031; Fisher’s exact test p-value 0.046). Taking into account the limitations inherent in the small sample size of the group with formal education (n = 13) and the subgroup still alive at 5 years (n = 2) when interpreting this result, in this study, having formal education was a risk factor for mortality (0.425; CI 0.230–0.787; p = 0.006), a result that included adjustment for baseline severity.

Educational level did not influence whether functional capacity improved or worsened (χ^2^ = 0.001; p = 0.982; Fisher’s exact test statistic 1.000), the intensity of the functional improvement observed in terms of Barthel Index scores (χ = 1.950; p = 0.163; Fisher’s exact test 0.193), or on whether this improvement allowed them, after the lockdown, to no longer be classified as functionally dependent individuals according to the local protocol (χ^2^ = 1.997; p = 0.158; Fisher’s exact test 0.178) (Table 3). However, among those with no education, 70% (n = 42) of those who remained dependent after the lockdown (n = 60) died, compared with 43.1% (n = 22) among those who no longer met the criteria for functional dependence according to the local protocol (n = 51) (OR 0.325; CI 0.149–0.711).

#### 8.1.4 Economic status

In this study, the cutoff point for classifying a person as having a low income was set at an annual income of less than 11,200 euros. This cutoff point is not arbitrary; rather, it is the threshold established by Spanish health authorities to identify individuals with low incomes and allow them to receive pharmaceutical benefits free of charge. Among those with low income (n = 60), 70% (n = 42) died, whereas among those with higher income (n = 67), 53.7% (n = 36) died (χ^2^ = 3.535; p = 0.060; Cramer’s V = 0.167; p = 0.060). In this study, economic status was not found to be an independent risk factor for mortality (HR 1.570; CI 0.999–2.468; p = 0.050).

Economic status did not influence whether functional capacity improved or worsened (χ^2^ = 2.254; p = 0.133; Cramer’s V = 0.142; p = 0.133) (Supplementary Table S1 in Section 1: Analysis of confounding factors, included in Module 2 of Supplementary Appendix 5), nor did it influence the intensity of the functional improvement observed in terms of Barthel Index scores (χ^2^ = 0.012; p = 0.914; Cramer’s V = 0.012; p = 0.914) (Supplementary Table S2 in Section 1: Analysis of confounding factors, included in Module 2 of Supplementary Appendix 5), or on whether this improvement allowed them, after the lockdown, to no longer be classified as functionally dependent individuals according to the local protocol (χ^2^ = 1.146; p = 0.284; Cramer’s V = 0.095; p = 0.284) (Table 3).

#### 8.1.5 Risk level

Being assigned a high risk level by the healthcare system based on diagnosed chronic conditions was not associated with higher mortality in this cohort (χ^2^ = 0.809; p = 0.369). Among those assigned a high risk (n = 66), 65.2% (n = 43) died, while among those assigned a lower risk level (n = 61), 57.4% (n = 35) died. After adjusting for baseline severity, risk level was not found to be a risk factor for mortality in this study (HR 1.134; CI 0.723–1.776; p = 0.584).

However, being classified as high-risk was associated with a lower probability of survival when functional dependence persisted after the COVID-19 lockdown, compared with those who, after the lockdown, were no longer classified as functionally dependent according to the local protocol (Barthel >60; OR 0.234; CI 0.079–0.695). Among those classified as high risk who remained functionally dependent (n = 35), 80% (n = 28) died, whereas among those who were no longer classified as functionally dependent after the lockdown because they had achieved a Barthel Index score greater than 60 (n = 31), 48.4% (n = 15) died.

Risk level did not influence whether functional capacity improved or worsened (χ^2^ = 0.831; p = 0.669) (Supplementary Table S1 in Section 1: Analysis of confounding factors, included in Module 2 of Supplementary Appendix 5), nor did it influence the extent of the functional improvement observed in terms of Barthel Index scores (χ^2^ = 2.836; p = 0.092) (Supplementary Table S2 in Section 1: Analysis of confounding factors, included in Module 2 of Supplementary Appendix 5), or on whether this improvement allowed them, after the lockdown, to no longer be classified as functionally dependent individuals according to the local protocol (χ^2^ = 0.751; p = 0.386) (Table 3).

#### 8.1.6 Level of intervention

However, being assigned a high level of intervention by the health center in relation to the needs of the diagnosed chronic conditions was associated in this cohort with higher mortality (χ^2^ = 5.510; p = 0.019). Among those assigned a high intervention level (n = 50), 74% (n = 37) died, whereas among those assigned a lower intervention level (n = 77), 53.2% (n = 41) died. After adjusting for baseline severity, the level of intervention was found to be an independent risk factor for mortality (HR 1.680; CI 1.069–2.640; p = 0.024). In the group of individuals assigned a high level of intervention, the means of the Barthel Index scores was 40.34 ± 20.310, with a confidence interval of 34.57–46.11, whereas, among those assigned a lower level of intervention, this mean was 45.18 ± 18.007, with a confidence interval of 41.09–49.27.

Among those with a high level of intervention who remained functionally dependent after lockdown (n = 29), 89.7% (n = 26) died, while among those who achieved independence (n = 21), 52.4% (n = 11) died (OR 0.127; CI 0.029–0.552).

The risk intervention level did not influence whether functional capacity improved or worsened (χ^2^ = 0.054; p = 0.816) (Supplementary Table S1 in Section 1: Analysis of confounding factors, included in Module 2 of Supplementary Appendix 5), nor did it influence the extent of the functional improvement observed in terms of Barthel Index scores (χ^2^ = 0.018; p = 0.894) (Supplementary Table S2 in Section 1: Analysis of confounding factors, included in Module 2 of Supplementary Appendix 5), or on whether this improvement allowed them, after the lockdown, to no longer be classified as functionally dependent individuals according to the local protocol (χ^2^ = 0.057; p = 0.811) (Table 3).

#### 8.1.7 Burden of chronicity and number of chronic diseases included in SCPCD

With regard to the number of chronic diseases, this number was not associated with whether the Barthel Index score had improved or worsened after the lockdown (Mann-Whitney U = 365.000; Z = −0.537; p = 0.591), or with the intensity of that improvement in terms of score (Mann-Whitney U = 301.500; Z = −0.130; p = 0.897) or the level of functional dependence achieved (Mann-Whitney U = 601.000; Z = −1.043; p = 0.297). Nor was it associated with 5-year survival (Mann-Whitney U = 625.500; Z = −0.131; p = 0.896).

With regard to the burden of chronicity, and in relation to its inclusion as a covariate in the development of regression models, having a high burden of chronicity (five or more diseases recorded in the SCPCD) was associated with being assigned a high risk level (χ^2^ = 14.009; p < 0.001 and OR 5.341; CI 2.111–13.505) and a high intervention level (χ^2^ = 12.486; p < 0.001 and OR 4.256; CI 1.853–9.773). Among those with a high chronicity burden (n = 34), 79.4% (n = 27) were at high risk, and 64.7% (n = 22) were assigned a high intervention level. These percentages were 41.9% (n = 39) and 30.1% (n = 28) among those who did not have this level of chronicity burden (n = 93). However, with regard to being classified as a polymedicated person, those with a high disease burden were not significantly more likely to be classified as polymedicated (χ^2^ = 1.958; p = 0.162).

Furthermore, in this study, the burden of chronicity (having five or more chronic diseases) was not associated with higher mortality (χ^2^ = 0.212; p = 0.645). Among those with five or more chronic conditions (n = 34), 64.7% (n = 22) died, while among those diagnosed with fewer than five chronic conditions (n = 93), 60.2% (n = 56) died. After adjusting for baseline severity, disease burden was not found to be an independent risk factor for mortality (HR 0.863; CI 0.527–1.414; p = 0.559).

Disease burden did not influence whether functional capacity improved or worsened (χ^2^ = 2.446; p = 0.118; Fisher’s exact test 0.139) (Supplementary Table S1 in Section 1: Analysis of confounding factors, included in Module 2 of Supplementary Appendix 5), nor on the magnitude of the functional improvement observed in terms of Barthel Index scores (χ^2^ = 2.959; p = 0.085; Fisher’s exact test statistic 0.113) (Supplementary Table S2 in Section 1: Analysis of confounding factors, included in Module 2 of Supplementary Appendix 5), but it did influence on whether this improvement allowed them, after the lockdown, to no longer be classified as functionally dependent individuals according to the local protocol (χ^2^ = 10.432; p = 0.001; Fisher’s exact test statistic 0.002) (Table 3).

#### 8.1.8 Drugs-polymedicated

In this study, considering the low representativeness of the subgroup of individuals who were not polymedicated, being polymedicated was not associated—after adjusting the data using Fisher’s exact test—with higher mortality (χ^2^ = 4.299; p = 0.038; Fisher’s exact test statistic 0.050). Among those prescribed five or more medications (n = 115), 59.1% (n = 68) died, whereas among those prescribed fewer than five medications (n = 11), 90.9% (n = 10) died. After adjusting for baseline severity, polypharmacy was not found to be an independent risk factor for mortality (HR 0.580; CI 0.288–1.171; p = 0.129).

Polypharmacy did not influence whether functional capacity improved or worsened (χ = 3.647; p = 0.056; Fisher’s exact test 0.102) (Supplementary Table S1 in Section 1: Analysis of confounding factors, included in Module 2 of Supplementary Appendix 5), on the degree of functional improvement observed in terms of Barthel Index scores (χ^2^ = 0.557; p = 0.455; Fisher’s exact test statistic 0.711) (Supplementary Table S2 in Section 1: Analysis of confounding factors, included in Module 2 of Supplementary Appendix 5), or on whether this improvement allowed them, after the lockdown, to no longer be classified as functionally dependent individuals according to the local protocol (χ^2^ = 1.195; p = 0.274; Fisher’s exact test statistic 0.350) (Table 3).

#### 8.1.9 Body mass index

Among the health variables included in this study, body mass index did not influence the probability of improving functional capacity after lockdown (Mann-Whitney U = 1092.500; Z = −1.023; p = 0.306) (Supplementary Table S3 in Section 1: Analysis of confounding factors, included in Module 2 of Supplementary Appendix 5), on the intensity of the response as measured by the Barthel Index score (Mann-Whitney U = 663.000; Z = −0.974; p = 0.330) (Supplementary Table S4 in Section 1: Analysis of confounding factors, included in Module 2 of Supplementary Appendix 5), or on whether this improvement resulted in the individual no longer being functionally dependent (Barthel >60 according to the local protocol; Mann-Whitney U = 1834.000; Z = −0.453; p = 0.651) (Supplementary Table S5 in Section 1: Analysis of confounding factors, included in Module 2 of Supplementary Appendix 5). Nor was it associated with the probability of survival (Mann-Whitney U = 1705.000; Z = −0.726; p = 0.468). These results persisted when analyzing the influence of BMI on survival in the group of individuals who, after the lockdown, no longer meet the local protocol criterion for functional dependence (Barthel >60; Mann-Whitney U = 301.000; Z = −1.251; p = 0.211) or who remained dependent (Mann-Whitney U = 466.000; Z = −0.027; p = 0.979).

### 8.2 Concurrent and mediating factors. preliminary analyses that contribute to the development of hypotheses

Among the many concurrent and mediating factors that could influence the observed results, this study analyzed the following: 1: Marital status married versus all other marital statuses. 2: Marital status widowed versus all other marital statuses. 3: Living situation: living independently, with children, or with other people who were neither the dependent person’s children nor their partner. 4: Community living, referring to being homebound versus having the ability to leave home. 5: Ability to leave home to go for a walk or engage in leisure activities. 6: Independently doing the shopping necessary for daily living. 7: Making the necessary purchases for daily living under the supervision of another person. 8: Presence of non-family caregivers. 9: Mobility assisted by assistive devices. Complete information on these factors is provided in Section 2, “Concurrent and Mediating Factors,” of Module 2, included in Supplementary Appendix 5.

When the association between these factors and the outcomes in functional trajectories observed following the lockdown due to the COVID-19 pandemic was analyzed, it was found that being married or widowed, having non-family caregivers, or using a walker as a mobility aid were not associated with an improvement or worsening of functional ability, nor with the extent of functional improvement observed in terms of Barthel Index scores, nor with that improvement allowed them, after the lockdown, to no longer be classified as functionally dependent individuals according to the local protocol.

However, living independently in the community rather than with one’s children (χ^2^ = 4.574; p = 0.032; Fisher’s exact test p-value 0.037), and being able to shop independently (χ^2^ = 7.649; p = 0.006), were associated in this study with an improvement in functional capacity after the lockdown, whereas using a wheelchair was associated with a lower probability of observing this improvement (χ^2^ = 7.222; p = 0.007).

None of the concurrent and/or mediating factors analyzed were associated with the intensity of the functional improvement observed after lockdown.

However, maintaining the ability to leave home versus living homebound (χ^2^ = 4.222; p = 0.040), being able to leave home to go for a walk or engage in leisure activities (χ^2^ = 4.779; p = 0.029), being able to independently purchase daily necessities (χ^2^ = 14.179; p < 0.001; Fisher’s exact test <0.001), or under the supervision of another person (χ^2^ = 5.190; p = 0.023; Fisher’s exact test statistic = 0.034), or using crutches or a cane (χ^2^ = 9.737; p = 0.002), were associated with the functional status of improved their functional capacity to a level of not being functionally dependent according to the local protocol (Barthel >60, Table 2). Conversely, living with children (χ^2^ = 6.215; p = 0.013; Fisher’s exact test statistic 0.018) and using a wheelchair (χ^2^ = 8.825; p = 0.003) were associated with the functional status of maintaining functional dependence after the lockdown.

The complete results of these analyses are presented in Supplementary Table S6 to S8 in Section 2: Analysis of Concurrent and Mediating Factors, included in Module 2 of Supplementary Appendix 5. This Supplementary Appendix describes in detail the role observed for each of these factors and based on the observed results and their implications for subsequent interpretation, provides an in-depth analysis of the factors “loss or change of non-family caregivers during lockdown” and the “housing situation of living with children.”

With regard to survival (Table 4), the factors of marital status (married or widowed), community living, maintaining the ability to leave home to go for a walk or engage in leisure activities, shopping under the supervision of another person, the presence of non-family caregivers, and the use of a mobility aid (crutches-cane, walker, wheelchair), were not associated with 5-year survival and, when the results were adjusted for baseline severity, did not prove to be independent risk factors for mortality. However, the housing situation of living with children was associated with survival (χ^2^ = 4.785; p = 0.029), although this association disappeared when the data were adjusted for baseline dependency level, and living with children did not prove to be an independent risk factor for mortality (HR 0.648; CI 0.387–1.083; p = 0.098). The ability to shop independently prior to lockdown was also associated with survival (χ^2^ = 8.686; p = 0.003; Fisher’s exact test p-value 0.005) (Table 4), and this association persisted when the results were adjusted for baseline severity, showing that this factor was an independent risk factor for mortality in this study (HR 0.360; CI 0.156–0.830; p = 0.017).

**TABLE 4:** Associations between community-based living and various instrumental activities assessed in this study with 5-year survival.

Impact of housing situation. Community living and instrumental activities of daily living in the survival. Data related to groups showing improvement/worsening in functional capacity after the lockdown				
Social factor	Status	Survival		Odds ratio (confidence interval)^a^
		Alive	Deceased	
Housing situation (n = 62)	Lives independently	51.2% (22)	48.8% (21)	1.4406 (0.485–4.282)
	Lives with others	42.1% (8)	57.9% (11)	
Housing situation (n = 93)	Lives independently	51.2% (22)	48.8% (21)	**2.982 (1.249–7.117)**
	Lives with children	26% (13)	74% (37)	
Community living (n = 112)	Leaves home	42.5% (31)	57.5% (42)	1.661 (0.729–3.783)
	Homebound	30.8% (12)	69.2% (27)	
Leaves home 1 (n = 75)	Alone	68.8% (11)	31.3% (5)	**4.290 (1.310–14.054)**
	Accompanied	33.9% (20)	66.1% (39)	
Leaves home 2 (n = 53)	Homebound	32.4% (12)	67.6% (25)	**0.218 (0.062–0.770)**
	Alone	68.8% (11)	31.2% (5)	
Leaves home 3 (n = 96)	Homebound	32.4% (12)	67.6% (25)	0.936 (0.390–2.244)
	Accompanied	33.9% (20)	66.1% (39)	
Leaves home for walks or leisure activities (n = 107)	Yes	44.4% (28)	55.6% (35)	1.813 (0.844–3.897)
	No	31.8% (14)	68.2% (30)	
Shopping independently (n = 109)	Yes	70.6% (12)	29.4% (5)	**4.960 (1.601–15.365)**
	No	32.6% (30)	67.4% (62)	
Shops under supervision (n = 108)	Yes	56.2% (9)	43.8% (7)	2.299 (0.784–6.740)
	No	35.9% (33)	64.1% (59)	

Data related to the groups of individuals who, following the COVID-19 pandemic lockdown, showed an improvement or a worsening in their functional capacity, estimated by the Barthel Index. Data for individuals who maintained the same Barthel Index score after the lockdown are not included. ^a^In bold: odds ratios that are statistically significant.

## 9 Exploratory analysis of the role of mobility-related activities in the barthel index, as a basis for the hypothesis of an expanded model that allows for the assessment of the mobility domain of intrinsic capacity in individuals with functional dependence

Among the ten activities included in the Barthel Index, the role of mobility is highly variable. In some, it is an essential requirement for performing the activity; in others, mobility is just as important as balance; and in still others, the need for mobility is minimal. With the aim of considering a broadening of the criteria currently used to assess the mobility component of intrinsic ability and adapting it to the reality of vulnerable populations, this study selected for evaluation the four activities from the Barthel Index where, in the authors’ criteria, mobility is an essential requirement for their performance: walking, chair-to-bed transfers, going up and down stairs, and toilet use.

In this study, dependence on assistance for walking was not associated with 5-year survival (χ^2^ = 3.317; p = 0.069; Fisher’s exact test statistic 0.076) (Table 5). Nor was it found to be an independent risk factor for mortality (HR 1.288; CI 0.746–2.222; p = 0.363). Regarding changes in functional trajectories observed after the lockdown, being dependent on others to walk (or being unable to walk and requiring a wheelchair for mobility) influenced whether functional capacity improved or worsened after the lockdown (χ^2^ = 11.563; p = 0.001). While 77.8% (n = 70) of those who were independent or required minimal assistance with walking had improved their functional capacity after the lockdown, among those who were dependent, this percentage was 40.9% (n = 9) (Table 6). However, being dependent or independent in walking did not influence the extent of the functional improvement observed in terms of Barthel Index scores (χ^2^ = 3.280; p = 0.070) (Table 7). Despite this result, the level of dependence on walking did influence that the functional improvement observed after lockdown reached a functional level that allowed these individuals to no longer meet the local protocol’s criterion for functional dependence (Barthel >60; χ^2^ = 13.439; p < 0.001; Fisher’s exact test <0.001). Of the 26 individuals who were dependent for walking, 88.5% (n = 23) remained functionally dependent after the lockdown, whereas this percentage was 48.5% (n = 49) among the 101 individuals who were independent for walking (Table 8).

**TABLE 5:** Association between the level of dependence observed after the COVID-19 pandemic lockdown for performing basic activities of daily living in which mobility is an essential requirement and 5-year survival.

Mobility. Daily living activities. and survival				
Basic activities of daily living	Level of dependence^a^	Survival^b^		Odds ratio (confidence interval)^c^
		Alive	Deceased	
Walks independently (n = 127)	Dependent (n = 26)	23.1% (6)	76.9% (20)	0.405 (0.150–1.093)
	Independent (n = 101)	42.6% (43)	57.4% (58)	
Chair-to-bed transfers (n = 127)	Dependent (n = 53)	20.8% (11)	79.2% (42)	**0.248 (0.111–0.555)**
	Independent (n = 74)	51.4% (38)	48.6% (36)	
Up-down stairs (n = 127)	Dependent (n = 79)	26.6% (21)	73.4% (58)	**0.259 (0.121–0.553)**
	Independent (n = 48)	58.3% (28)	41.7% (20)	
Toilet use (n = 127)	Dependent (n = 23)	8.7% (2)	91.3% (21)	**0.116 (0.026–0.518)**
	Independent (n = 104)	45.2% (47)	54.8% (57)	

^a^ Level of dependence: Dependent: Cannot perform the activity or requires significant assistance to do so. Independent: performs the activity independently or with minimal assistance.

^b^ Survival at 5-year.

^c^In bold: odds ratios that are statistically significant.

**TABLE 6:** Association between the level of dependence observed after the COVID-19 pandemic lockdown for performing basic activities of daily living in which mobility is an essential requirement, and the presence, after the lockdown, of an improvement or worsening compared to the pre-lockdown Barthel Index score.

Mobility. Daily living activities. and functional response after lockdown I				
Basic activities of daily living	Level of dependence^a^	Changes in the barthel index score after lockdown		Odds ratio (confidence interval)
		Worsening in barthel index score (n = 33)	Improvement in barthel index score (n = 79)	
Walks independently (n = 112)	Dependent (n = 22)	59.1% (13)	40.9% (9)	0.198 (0.074–0.529)
	Independent (n = 90)	22.2% (20)	77.8% (70)	
Chair-to-bed transfers (n = 112)	Dependent (n = 44)	61.4% (27)	38.6% (17)	0.061 (0.022–0.171)
	Independent (n = 68)	8.8% (6)	91.2% (62)	
Up-down stairs (n = 112)	Dependent (n = 66)	45.5% (30)	54.5% (36)	0.084 (0.024–0.297)
	Independent (n = 46)	6.5% (3)	93.5% (43)	
Toilet use (n = 112)	Dependent (n = 17)	76.5% (13)	23.5% (4)	0.082 (0.024–0.279)
	Independent (n = 95)	21.1% (20)	78.9% (75)	

^a^ Level of dependence: Dependent: Not able to perform the activity or requires significant assistance to do so. Independent: Performs the activity independently or with minimal assistance.

**TABLE 7:** Association between the intensity of the response observed following the COVID-19 pandemic lockdown, with respect to functional capacity to perform basic activities of daily living as assessed by the Barthel Index, and the level of dependence for performing the activities included in that index for which mobility is an essential requirement.

Mobility. Daily living activities. and functional response after lockdown II				
Basic activities of daily living	Level of dependence^a^	Intensity of functional response observed after lockdown (in terms of barthel index Score)^b^		Odds ratio (confidence interval)^d^
		Moderate: 5–20 points (n = 39)	Clinically relevant^c^: 25–95 points (n = 40)	
Walks independently (n = 79)	Dependent (n = 9)	77.8% (7)	22.2% (2)	4.156 (0.806–21.433)
	Independent (n = 70)	45.7% (32)	54.3% (38)	
Chair-to-bed transfers (n = 79)	Dependent (n = 17)	58.8% (10)	41.2% (7)	1.626 (0.548–4.821)
	Independent (n = 62)	46.8% (29)	53.2% (33)	
Up-down stairs (n = 79)	Dependent (n = 36)	63.9% (23)	36.1% (13)	**2.986 (1.191–7.486)**
	Independent (n = 43)	37.2% (16)	62.8% (27)	
Toilet use (n = 79)	Dependent (n = 4)	100% (4)	0% (0)	__
	Independent (n = 75)	46.7% (35)	53.3% (40)	

^a^ Level of dependence: Dependent: Cannot perform the activity or requires significant assistance to perform it. Independent: Performs the activity independently or with minimal assistance.

^b^ Intensity of the observed functional response: Moderate: 5–20 points.

^c^ Clinically relevant: 25 to 95 points. Clinically relevant: mathematically implies an improvement that includes functional independence in at least one activity on the Barthel Index.

^d^In bold: odds ratios that are statistically significant.

**TABLE 8:** Association between the level of functional dependence observed after the COVID-19 pandemic lockdown for performing basic activities of daily living and the level of dependence for performing the activities on the Barthel Index in which mobility is an essential requirement.

Mobility. Daily living activities. and functional response after lockdown III				
Basic activities of daily living	Level of dependence^a^	Level of functional dependence after lockdown according to protocol^b^		Odds ratio (confidence interval)^c^
		Remains functionally dependent (n = 72)	No dependent according the local protocol (n = 55)	
Walks independently (n = 127)	Dependent (n = 26)	88.5% (23)	11.5% (3)	8.136 (2.297–28.822)
	Independent (n = 101)	48.5% (49)	51.5% (52)	
Chair-to-bed transfers (n = 127)	Dependent (n = 53)	92.5% (49)	7.5% (4)	27.163 (8.758–84.244)
	Independent (n = 74)	31.1% (23)	68.9% (51)	
Up-down stairs (n = 127)	Dependent (n = 79)	78.5% (62)	21.5% (17)	13.859 (5.752–33.389)
	Independent (n = 48)	20.8% (10)	79.2% (38)	
Toilet use (n = 127)	Dependent (n = 23)	100% (23)	0% (0)	--
	Independent (n = 104)	47.1% (49)	52.9% (55)	

^a^ Level of Dependence: Level of functional dependence for performing a specific activity of the Barthel Index: Dependent: cannot perform the activity or requires significant assistance to perform it. Independent: Performs the activity independently or with minimal assistance.

^b^ Level of functional dependence after lockdown according to protocol: Level of functional dependence after the COVID-19 pandemic lockdown according to the local protocol criterion for functional dependence: Remains functionally dependent: Barthel score ≤60 before and after lockdown. No Dependent According the Local Protocol: no longer meet the local protocol criterion for functional dependence (Barthel >60), Barthel score ≤60 before lockdown and greater than 60 after lockdown.

^c^In bold: odds ratios that are statistically significant.

In summary, from a functional perspective, it was observed that the probability of improving functional capacity during the COVID-19 pandemic lockdown was higher when the person was independent in walking, while when the person was dependent for this activity, or used a wheelchair, it was more likely that their previous functional capacity would worsen during the lockdown. This same result was observed when analyzing whether this improvement meant that patients no longer met the criteria for functional dependence according to the local protocol (Barthel >60 according to that protocol) or whether functional dependence persisted.

With regard to the second activity assessed on the Barthel Index—being dependent for chair-to-bed transfers—this was associated with 5-year survival (χ^2^ = 12.200; p < 0.001) (Table 5) and, in this study—after adjusting for baseline severity as in the other analyses—it was an independent risk factor for mortality (HR 1.858; CI 1.143–3.020; p = 0.012). Among those who were dependent for this activity (n = 53), 79.2% (n = 42) died, compared with 48.6% (n = 36) among those who were not dependent (n = 74). Regarding changes in functional trajectories observed after the lockdown, being dependent for chair-to-bed transfers influenced whether functional capacity improved or worsened after the lockdown (χ^2^ = 35.483; p < 0.001; Fisher’s exact test <0.001) (Table 6). While 91.2% (n = 62) of those who were independent or required minimal assistance had improved their functional capacity after the lockdown, among those who were dependent, this percentage was 38.6% (n = 17). Among those whose functional capacity worsened after the lockdown, 81.8% (n = 27) were dependent for chair-to-bed transfers. However, whether patients were dependent or independent in performing these transfers did not influence the extent of the functional improvement observed in terms of Barthel Index scores (χ^2^ = 0.775; p = 0.379) (Table 7). Despite this result, it was observed that being dependent on chair-to-bed transfers did influence whether that improvement allowed participants to no longer meet the local protocol criterion for functional dependence (Barthel >60; χ^2^ = 47.375; p < 0.001; Fisher’s exact test <0.001) (Table 8). Of the 53 individuals who were dependent, 92.5% (n = 49) remained functionally dependent after the lockdown, whereas this percentage was 31.1% (n = 23) among those who were independent (n = 74).

In summary, these results showed that nine out of ten people who were dependent for this activity remained functionally dependent after the lockdown, while seven out of ten people who were independent in performing it—or who required minimal assistance—achieved a level that was not functionally dependent according to the local protocol criterion for functional dependence (Barthel >60).

The third activity assessed—the ability to go up and down stairs—was also associated with 5-year survival (χ^2^ = 12.703; p < 0.001) (Table 5) and was also found to be an independent risk factor for mortality (HR 2.053; CI 1.214–3.471; p = 0.007). In this cohort, 73.4% (n = 58) of those who were dependent on others to perform this activity died during the follow-up period, compared with 41.7% (n = 20) of those who were independent in performing it or required minimal assistance. Regarding the changes observed after the lockdown, requiring assistance to perform this activity influenced whether functional ability improved or worsened (χ^2^ = 19.770; p < 0.001) (Table 6). While 93.5% (n = 43) of those who were independent or required minimal assistance to go up and down stairs (n = 46) had improved their functional capacity after the lockdown, among those who were dependent (n = 66), this percentage was 54.5% (n = 36). Being dependent in this activity also influenced the magnitude of the response in terms of Barthel Index scores (χ^2^ = 5.580; p = 0.018), observing that 62.8% (n = 27) of those who improved by between 25 and 95 points were independent in going up and down stairs, while this percentage was 36.1% (n = 13) among those who were dependent (Table 7). Finally, being dependent on assistance for going up and down stairs was associated with a lower probability of reached a functional level that allowed these individuals to no longer meet the local protocol’s criterion for functional dependence (Barthel >60; χ^2^ = 40.415; p < 0.001; Fisher’s exact test< 0.001). 78.5% (n = 62) of the individuals who were dependent for going up and down stairs (n = 79) remained functionally dependent after the lockdown, whereas this percentage was 20.8% (n = 10) among those who were independent (Barthel >60 according to the local protocol; n = 48) (Table 8).

In summary, and similar to what was observed with the activity of chair-to-bed transfers, eight out of ten people who were dependent for this activity remained functionally dependent after the lockdown, while eight out of ten people who were independent in performing this activity—or needed minimal assistance—achieved a level that was not functionally dependent according to the local protocol criterion for functional dependence (Barthel >60).

Finally, the fourth activity evaluated—toilet use—was also associated with 5-year survival (χ^2^ = 10.587; p < 0.001; Fisher’s exact test 0.001) (Table 5) and was also shown to be an independent risk factor for mortality (HR 1.845; CI 1.047–3.250; p = 0.034). In this cohort, 91.3% (n = 21) of those who were dependent on others to perform this activity (n = 23) died during the follow-up period, compared with 54.8% (n = 57) of those who were independent or required minimal assistance (n = 104). Regarding the changes observed after the lockdown, requiring assistance to perform this activity influenced whether functional ability improved or worsened (χ^2^ = 21.308; p < 0.001; Fisher’s exact test <0.001) (Table 6). While 78.9% (n = 75) of the individuals who were independent or required minimal assistance to go up and down stairs (n = 95) had improved their functional capacity after the lockdown, among those who were dependent (n = 17), this percentage was 23.5% (n = 4). None of the individuals who were dependent for this activity showed an improvement in functional ability within the range of 25–95 points after the lockdown (Table 7). Similarly, none of the individuals who were dependent on others to use the bathroom showed a level of functional improvement after the lockdown that would allow them to regain a functional level that allowed these individuals to no longer meet the local protocol’s criterion for functional dependence (Barthel >60); therefore, 100% of them remained functionally dependent (Table 8).

Among the four activities of the Barthel Index analyzed in relation to mobility, the activity of going up-down stairs was the one in which a higher percentage of independent individuals were able to improve their functional capacity, achieve a clinically significant increase in their Barthel Index score, and exceed the 60-point threshold to be considered functionally independent according to the protocol.

In contrast, use of the toilet was the activity in which dependence was associated with the highest percentage of people whose Barthel Index scores worsened after the lockdown; it was also the activity for which, in cases where the score improved, there were only moderate increases of 5–20 points, results that were insufficient to exceed the 60-point threshold, thereby maintaining these individuals, overall, as functionally dependent.

Supplementary Tables SE1 and SE2 in Appendix 6: “Mobility and the Barthel Index,” the analyses conducted for the remaining activities of the Barthel Index are presented, in relation to whether there was an improvement or worsening of functional capacity after the lockdown, and to the functional outcome of either maintaining a state of functional dependence after the lockdown or no longer meeting the criteria for functional dependence according to the local protocol (Barthel >60). At the same time, Supplementary Table SE3 presents the interrelationships between the level of dependence for walking and the level of dependence for the remaining activities of the Barthel Index, in relation to the observed outcome of improved functional capacity, or that such improvement reaches a level allowing for the determination that these individuals no longer meet the local protocol’s criterion for functional dependence (Barthel >60). Co-independence with other activities when the individual was independent in walking was associated with achieving an improvement in functional capacity following lockdown and with this improvement reaching a level that allows for the determination that these individuals no longer meet the local protocol’s criteria for functional dependence (Barthel >60).

When the survival analysis was conducted by comparing the level of functional dependence assessed after lockdown according to our health system’s protocol and the level of dependence in these four activities of the Barthel index in which mobility was an essential requirement for their performance (Table 9), it was observed that nine out of ten people who were dependent on others to walk and remained functionally dependent after the COVID-19 pandemic lockdown died during the 5-year follow-up period. However, 100% of the individuals who—despite being dependent for walking prior to lockdown and remaining so afterward—managed to improve their overall functional capacity after lockdown to a level that did not imply functional dependence, according to the criteria of the local protocol for functional dependence (Barthel >60), were still alive at 5 years (Table 9). Among individuals who were independent in walking, remaining functionally dependent after the lockdown or improving overall functional ability to the point of no longer being functionally dependent according to the criteria of the local protocol did not result in a higher or lower probability of survival (OR 0.448; CI 0.200–1.006). Among those who remained dependent, 67.3% of those who were independent in walking died, compared with 48.1% among those who achieved functional improvement such that they were no longer classified as functionally dependent according to the local protocol. At the same time, all individuals who were dependent for walking before the lockdown and who, after the lockdown, improved their functional capacity to a level that no longer met the criteria for functional dependence according to the local functional dependence protocol (Barthel >60), were still alive at 5 years. However, among individuals who remained functionally dependent after lockdown and were dependent on others for walking, 87% died during this period.

**TABLE 9:** Analysis of observed survival when contrasting the level of functional dependence obtained in the functional capacity assessment after lockdown, using the Barthel Index and applying the criteria of our health system’s protocol, with the level of dependence for performing the activities on the Barthel Index in which mobility is an essential requirement.

Survival analysis in relation to functional levels after lockdown and the mobility assessment using levels of dependency to perform activities on the barthel index				
Functional status after lockdown^a^- barthel index activity				
Remains functionally dependent after the lockdown^b^				
Mobility factor	Level of dependence^c^	Survival		Statistical test
		Alive (n = 19)	Deceased (n = 53)	Odds ratio (confidence Interval)^d^
Walks independently	Dependent	13% (3)	87% (20)	0.309 (0.080–1.196)
	Independent	32.7% (16)	67.3% (33)	
Chair-to-bed transfers	Dependent	18.4% (9)	81.6% (40)	**0.293 (0.098–0.876)**
	Independent	43.5% (10)	56.5% (13)	
Going up-down stairs	Dependent	22.6% (14)	77.4% (48)	0.292 (0.074–1.154)
	Independent	50% (5)	50% (5)	
Toilet use	Dependent	8.7% (2)	91.3% (21)	**0.179 (0.037–0.858)**
	Independent	34.7% (17)	65.3% (32)	

No dependent according the local protocol^e^				
Mobility factor	Level of dependence^b^	Survival		Statistical test
		Alive (n = 30)	Deceased (n = 25)	Odds ratio (confidence Interval)
Walks independently	Dependent	100% (3)	0% (0)	__
	Independent	51.9% (27)	48.1% (25)	
Chair-to-bed transfers	Dependent	50% (2)	50% (2)	0.821 (0.107–6.293)
	Independent	54.9% (28)	45.1% (23)	
Going up-down stairs	Dependent	41.2% (7)	58.8% (10)	0.457 (0.142–1.463)
	Independent	60.5% (23)	39.5% (15)	
Toilet use	Dependent	0% (0)	0% (0)	__
	Independent	54.5% (30)	45.5% (25)	

^a^Functional status after the Covid-19 pandemic lockdown according to the local protocol criterion for functional dependence.

^b^Remains functionally dependent after the lockdown: Barthel score ≤60 before and after lockdown.

^c^Level of Dependence: Level of functional dependence for performing a specific activity of the Barthel Index: Dependent: Cannot perform the activity or requires significant assistance to perform it. Independent: Performs the activity independently or with minimal assistance.

^d^In bold: odds ratios that are statistically significant.

^e^No Dependent According the Local Protocol: no longer meet the local protocol criterion for functional dependence (Barthel >60), Barthel score ≤60 before lockdown and greater than 60 after lockdown.

In parallel, eight out of ten people who remained dependent after the lockdown, and whose dependency profile included dependence on chair-to-bed transfers, had died during the 5-year follow-up period. This same result was observed in nine out of ten people who remained dependent after the lockdown and were also dependent on assistance with toilet use.

## 10 Discussion

The 100-day lockdown, during which the population remained in their usual homes, provided a window of opportunity to observe changes in the functional trajectories of a population that meet criteria for vulnerability. This population consisted of older adults who had several levels of functional dependence for performing basic activities of daily living and who were exposed to exceptional circumstances in which various factors—not analyzed in this study, but which have been addressed in other studies—such as stress, fear of death due to the high COVID-19 mortality rate in their age group, social isolation, or the loss of their baseline security, likely played a role in the observed outcomes (Vermote et al., 2023; Kiah et al., 2022). On the other hand, within a proactive healthcare system model, the healthcare personnel who participated in the study were familiar with this population’s baseline conditions, and prior to the lockdown, these individuals were regularly monitored by these personnel, which made it possible to detect and assess changes (Boylan et al., 2023).

These exceptional circumstances made it possible to observe that, contrary to what would be empirically expected (Blanco-Rambo et al., 2024), namely, a progressive deterioration or stabilization of the baseline situation, the majority of this population experienced changes in their functional trajectories. These changes, and their magnitude, were reflected in the Barthel Index scores obtained after the lockdown, with three out of every ten people in this cohort showing an improvement of between 25 and 75 points, a criterion established to interpret this improvement as clinically relevant, since achieving it requires an improvement in functional ability in no fewer than three activities on that index. But it was also reflected in a clinically and socially significant outcome in our context: exceeding the 60-point threshold on the Barthel Index and no longer being classified as a person with functional dependence according to our healthcare system’s protocol. This outcome, achieved by four out of every ten people in this cohort, was reached in half of the cases through improvements in functional ability ranging from 25 to 75 points. This level of functional improvement, which was statistically significant, suggesting that the results were not due to the random effect, the high percentage of people who exhibited it, and the fact that the nurse accompanying the one conducting the post-lockdown assessment was familiar with the pre-pandemic baseline situation and could clinically certify that the observed improvement was genuine, provided evidence that a large portion of this cohort showed changes in functional status following the COVID-19 pandemic lockdown.

Furthermore, when the three models were compared using logistic regression (clinical-social baseline, pre-lockdown baseline, and post-lockdown incremental model), this functional change observed after lockdown was found to have incremental predictive value, with a decrease in the AIC (as well as the BIC) of more than 10 in the incremental model compared to the clinical-social baseline model. According to Burnham and Anderson’s general rule (Burnham and Anderson, 1998), this indicated that the incremental model was the best of the three analyzed, offering a better balance between fit and complexity. On the other hand, the AUC observed was higher for the incremental model (0.921) than for the reference model (0.846).

However, the significance of this change, in terms of its association with long-term all-cause mortality, showed that improving functional capacity alone was not enough. Not even when done with intensity, with increases ranging from 25 to 75 points. Although this cohort showed that the mean survival time for individuals whose functional capacity improved after the lockdown was longer than that of individuals whose functional capacity worsened, and that the mean survival time for individuals whose functional capacity improved to a clinically relevant intensity was longer than that of those whose improvement was more moderate, the differences were not statistically significant. These results contrasted with the fact that those who managed to exceed the 60-point threshold on the Barthel Index did indeed have lower all-cause mortality compared to those who remained functionally dependent after the lockdown.

On the one hand, this result supported the criterion of functional dependence established in our protocol. It appeared to justify that measures targeting the population with functional dependence should focus primarily on those whose score on that index was 60 or lower. Furthermore, the ability to improve functional capacity and surpass the threshold that determined the need for specific health and social follow-up (Barthel <60) could potentially become—if these results are confirmed in other studies—an indirect indicator of a person’s intrinsic robustness of baseline health and biological reserve.

On the other hand, among the exploratory hypotheses that could be behind this result—in which surpassing a threshold was more important than the intensity of functional improvement itself—the very design of the Barthel Index likely provided information that may not have been sufficiently valued until now. Mathematically, exceeding the 60-point threshold implies independence in some of the activities on that index, since the maximum achievable score in a state of non-independence across the ten activities of the Barthel Index is 40 points, or 60 if we consider the need for minimal assistance.

Half of the people who crossed the 60-point threshold did not need to improve their functional capacity in a clinically relevant way. Their improvements ranged from 5 to 20 points. This result implied that they started from a better starting point, that they already had a higher baseline score, which translated into better functional ability than those who started from worse positions, and this already gave them an advantage in this race to reach the 60-point threshold, which this study has shown to be a goal. But, furthermore, on a scale ranging from 0 to 100, with a cutoff point of 60 and 5-point increments for each possible step, their maximum potential for improvement was only 35–40 points. Their range for improvement was more limited, but they may have needed less effort to reach the goal because they were already independent in some activities. Having a Barthel Index score above 60 mathematically implied that a person is not dependent for performing some of the activities listed in that index. And this finding was confirmed in this study. Nine out of ten people who exceeded the 60-point threshold with score increases on this index ranging from 5 to 20 points did so at the expense of becoming independent—or requiring minimal assistance—when walking or chair-to-bed transfers, and half did so at the expense of being independent when going up and down stairs. It is worth noting that all those who exceeded this threshold with increases of 5–20 points were people who, after the lockdown, were independent in the toilet use. Those who required a smaller increase in their score to reach that threshold were individuals who, ultimately, after the lockdown, had better mobility, assessing this mobility within the activities of the Barthel Index for which their presence is essential to perform them.

If confirmed in studies with larger cohorts, this result could also be interpreted differently. As part of an exploratory hypothesis, the 60 thresholds could potentially serve as a functional marker. This potential functional marker could make it possible to establish specific functional recovery goals at the care level—within the primary care setting and focused on addressing the needs of these individuals—but also within the scope of social and healthcare facilities (nursing homes, tertiary hospitals for the rehabilitation of chronically ill patients). Furthermore, it could also be useful for social services, within development and intervention policies carried out through day centers, and in planning social measures aimed at healthy aging.

As for the other half of the cohort that exceeded the 60-point threshold—established in our local protocol as the criterion for functional dependence—and improved their functional capacity in a clinically relevant manner, with increases in Barthel Index scores ranging from 25 to 75 points, this improvement was associated with the fact that virtually all participants in this group were independent after the lockdown or required minimal assistance with walking, chair-to-bed transfers, and the toilet use, while eight out of ten were also independent when going up and down stairs.

Greater independence in performing those activities on the Barthel Index for which mobility was an essential component allowed for greater increases in Barthel Index scores, and these increases were more strongly associated with no longer meeting the local protocol criterion for functional dependence (Barthel >60). Conversely, when the baseline status was dependence in performing these activities, the typical outcome following lockdown was a worsening of the Barthel Index score, or modest improvements in the score that were insufficient to achieve a state of functional independence according to the criteria of the local functional dependence protocol.

Furthermore, this improvement was not a one-time phenomenon for many of these individuals but rather persisted over time. This persistence over time, as reflected in the 3-year follow-up, along with the intensity of the outcome, expressed both as the percentage of participants whose improvement in the Barthel Index score was clinically significant and as the percentage of participants who no longer meet the criteria for functional dependence according to the protocol, suggested a response that went beyond a mere change in functional trajectories resulting from a reflexive adaptive reaction to an unexpected situation. Within a hypothesis-generating exploratory framework, the nature and persistence of this response over time suggested that the response observed following the lockdown could be framed within the concept of functional resilience, which would express the intrinsic capacity these individuals possessed in a specific environment and situation in the form of functional capacity.

However, within this same exploratory framework, the concept of “functional resilience” refers primarily to maintaining essential functions, and to restoring these functions to their pre-event normal level after an event, making use of one’s usual abilities. But after the lockdown, what was observed was not that many of these individuals regained their functional capacity to pre-event levels, but rather that they regained a level of functionality they did not possess before the event. In other words, they demonstrated a potential functional capacity that they did not routinely use in their daily lives. Maintaining the same exploratory framework for generating hypotheses, this extra functional capacity, which was latent, could perhaps be classified as intrinsic capacity. And perhaps this could lead to the definition of a new construct: the latent intrinsic capacity.

This new construct would represent a functional reserve within the mobility domain of intrinsic capacity, which would remain inactive under baseline conditions, such as those observed in this population prior to the COVID-19 pandemic. Its activation would require the presence of a sufficiently significant situation, such as the COVID-19 pandemic (Rivera de los Santos et al., 2011; Seah et al., 2022). This latent intrinsic capacity, if confirmed through future validation using measures that are conceptually and empirically distinct from the functional outcomes used to define it, would be one of the pillars underpinning the resilience observed in this study (Cesar et al., 2018; Grigoraş et al., 2025).

The observation that changes in functional trajectory following lockdown were evident in individuals who shared specific characteristics could support the hypothesis of a latent intrinsic capacity. The individuals in whom these changes in functional trajectory were clinically relevant mostly had a moderate level of functional dependence prior to the COVID-19 pandemic lockdown; that is, they had better functional capacity. Furthermore, these were individuals whose “mobility” domain of intrinsic capacity, as broadly defined and assessed across multiple aspects of daily life where it was evident, also exhibited a better profile. This profile, in turn, was associated with greater independence in their environment.

Other factors could also potentially support this hypothesis of a resilience based on an underlying functional capacity. Based on the results of this study—and specifically on the observation that this underlying functional capacity was evident in both men and women, and at any age, including people in their nineties and centenarians—going beyond what was conceptually expected in an era where ageism plays a significant role (Gao et al., 2025), it could be suggested that there was a latent functional capacity independent of biological conditions, waiting for the circumstances that would foster its development.

The finding in this study that the functional changes observed after lockdown were not associated with educational level, socioeconomic status, or marital status could also support the hypothesis that these functional trajectories reflect the expression of a theoretical latent intrinsic capacity. Nor were they associated with relevant health factors, such as the burden of chronic conditions, polypharmacy, obesity, or being a high-risk individual due to underlying medical conditions who was also assigned a high level of care by health services. These results suggest a general response that was not conditioned by health or socioeconomic factors, one that overcame any prior barriers and appeared to stem from a common goal shared by all these individuals: to get ahead. This could represent a potential form of resilience based on functional capacity, which, in the face of potentially adverse circumstances, allowed for the mobilization of abilities not usually used (Grigoraş et al., 2025; Li et al., 2024). These abilities could be compatible with a latent intrinsic capacity, the potential existence of which would require specific studies designed for that purpose.

Within a complex systems model based on resilience, nonlinearity, and heterogeneity, and keeping these approaches within the framework of hypotheses, these facets were able to establish dynamic interactions that became evident during the lockdown due to the COVID-19 pandemic, creating global conditions that influenced the results (Beard et al., 2019; Waris et al., 2022b; Kok et al., 2025). As described by other authors (Koivunen et al., 2024), the network formed around these personal and environmental factors became denser as the age of its components increased; in this situation, the system’s overall resilience was observed to decrease, with both the level of dependency and mobility—the latter defined as the ability to perform certain basic and instrumental activities of daily living—continuing to play a significant role (Beard et al., 2019; Qi et al., 2024).

On the other hand, mobility impairment is a key factor in the functional decline, and this functional decline has been associated in several studies with the intrinsic capacity impairment (Wang Q. et al., 2025; Cheng et al., 2025; Sánchez-Sánchez et al., 2024b). Both mobility impairment and intrinsic capacity impairment are predictors of adverse events (Zhao et al., 2024; Campbell et al., 2023) and mortality risk (Sánchez-Sánchez et al., 2024b; Li et al., 2024). However, the concept of latent intrinsic capacity could potentially be masked within the concept of resilience and may be an aspect that is not typically addressed specifically when analyzing resilience. Both concepts, resilience and intrinsic capacity, are intricately linked (Grigoraş et al., 2025), with the intrinsic capacity being a determinant to the resilience (Chhetri et al., 2021), especially physical-functional resilience. But, supporting this suggestion based on the observed results, the development of resilience might not stem solely from intrinsic capacity, but perhaps also from a hypothetical latent intrinsic capacity, that would allow resilience to reach levels unattainable through intrinsic capacity alone. This cohort made changes in their functional status that were of a magnitude not likely predicted by their intrinsic capacity; they even stopped using wheelchairs, walkers, or crutches, and these functional changes persisted over time and were associated with their long-term survival. Thus, based on the results observed in this study, it could be hypothesized that this resilience might be compatible with the existence of a latent intrinsic capacity, which may have enabled these changes in functional capacity to perform basic and instrumental activities of daily living (Wang Q. et al., 2025). However, the potential effects of this type of resilience on overall functionality and survival require further independent studies to confirm them (Grigoraş et al., 2025).

With regard to intrinsic capacity, in individuals with functional dependence, the assessment of the mobility domain through the activity of walking a specific distance may have limitations, and this limitation could result in grouping together within the same level individuals who, as this study shows, have very diverse functional profiles and trajectories, profiles that are associated with survival in very different ways. The results observed in this study regarding the Barthel Index activities in which mobility is a key factor for performance suggest, as an exploratory hypothesis, that these activities could perhaps be incorporated into the assessment of the mobility domain, thereby broadening the range of profiles that may be eligible for differentiated treatment at both the healthcare and social levels. This hypothesis requires specific studies to confirm its feasibility.

Within these profiles, and while maintaining a hypothesis-generating exploratory approach, balance is also necessary for adequate mobility, especially during transfers. Thus, mobility may be impaired when, in the absence of motor or joint problems, balance is compromised (Tan et al., 2024; Campbell et al., 2023; Cui et al., 2025). In these situations, other activities on the Barthel Index, in which mobility—understood as the act of walking—is less relevant, but balance, limb control, and posture are necessary, could also provide relevant information within a broader “mobility” domain (Zhao et al., 2024). Perhaps the concept of intrinsic ability should take these aspects into account, as they likely imply greater frailty (Cheng et al., 2025; Sánchez-Sánchez et al., 2024b; Gencer et al., 2025; Song et al., 2025).

Another factor that could support this hypothesis is the fact that the manifestation of this theoretical latent intrinsic ability observed after the lockdown was not associated with educational attainment, socioeconomic status, or marital status. Nor was it associated with health factors such as the burden of chronicity, polypharmacy, obesity, or being a high-risk individual due to underlying diseases who was also assigned a high level of intervention by health services. These results reported a general response that was not conditioned by health or socioeconomic factors, which overcame any previous barriers in pursuit of a common goal for all these people: to get through. A form of resilience based on functional capacity that was likely manifesting itself in adverse circumstances as a latent intrinsic capacity that made it possible to activate capacities that were not normally used (Grigoraş et al., 2025; Chhetri et al., 2021).

As for the intrinsic capacity, according to various studies, more than two-thirds of older adults living in the community show deterioration in intrinsic capacity (Cao et al., 2024a), and this deterioration influences mortality (Beyene et al., 2024; Sánchez-Sánchez et al., 2024a; Yu et al., 2022). Other studies have found socioeconomic risk factors associated with intrinsic capacity impairment, such as advanced age, low educational or economic status, being female, or being single, in addition to disease burden (Morse et al., 2023; Tang et al., 2025; Yu et al., 2023; Song et al., 2025). In this study, advanced age modulated the intensity of the functional response observed after lockdown. Although this was an older cohort, the changes made were sustained over time in the subgroup that remained in follow-up, showing that there is potential for functional improvement at any age, although this potential only had effects on survival under certain conditions. On the other hand, subjective age, or subjective aging, which were not analyzed in this study, may also have played a role in the results (Petashnick et al., 2022; Westerhof et al., 2023; Silva et al., 2023).

Regarding age and survival, older age was associated in this study with a lower probability of survival, but in the presence of functional dependence, it was this dependence that modulated survival, while among those who achieved a level of not being functionally dependent according to the criteria of the local functional dependence protocol (Barthel >60), age was the modulating factor, as in the general population. However, it was also observed that the effect of minimal functional dependence did not disappear, with a gradient of association between a score above 60 on the Barthel index and mortality. In relation to these results, age may have operated as a modulator of intrinsic capacity (Beard et al., 2019). At the same time, in two groups of people, age was a limiting factor in whether this response was significant enough to exceed the 60-point threshold on the Barthel Index: the oldest individuals and the younger individuals with functional dependence: the oldest-old adults (92 years of age and older) and younger people with functional dependence. Although it can be posited that, in older adults, prior poorer functional status might make it more difficult to achieve this level of response, the causes remained unclear among younger individuals.

Although some studies have found that disability increases mortality more in men than in women (Tiainen et al., 2013), this study did not observe such differences during the 5-year follow-up. Nor did these differences become apparent when the data were analyzed based on whether participants remained functionally dependent or had achieved a level of not being functionally dependent according to the criteria of the local functional dependence protocol (Barthel >60).

Similarly, having no education also implied a lower probability of survival when functional dependence remained after lockdown. Both situations were likely associated with a poorer baseline profile, in which their intrinsic capacity was likely affected (Tang et al., 2025; Yu et al., 2023; Song et al., 2025).

Broadening the scope of factors that may have contributed to the results observed in this study, prior to the pandemic, this cohort was receiving protocolized follow-up from health services, both at home and at the health center, and continued to be contacted by phone during the lockdown, a situation that may have contributed to their greater resilience (Gustafsson et al., 2022). Life experiences throughout lives likely marked by precariousness (Morse et al., 2023; Rivera de los Santos et al., 2011; Seah et al., 2022) and age itself (Losada-Baltar et al., 2022) may also have contributed to greater resilience (Sterina et al., 2022). All of these factors may have contributed to the fact that, starting from an initial reactive response to a crisis, adaptive processes were set in motion that activated latent capacities, which in many cases became structural (Álvarez and Pinto, 2023).

As alternative explanations for the observed results and the hypotheses proposed to provide answers to these findings, the persistence of functional improvement at 3 years does not entirely rule out regression to the mean, as it represents only a part of the cohort; therefore, at least in certain cases, this could be an alternative explanation for the observed changes. However, ANCOVA models were applied to adjust for baseline differences, using baseline severity—which reflected the level of functional dependence prior to the lockdown—a method that mathematically adjusts for the effect of regression to the mean. Furthermore, although it was not quantified, the objective assessment by the patient’s primary nurse regarding whether the change in functional status was real also likely contributed to minimize this impact of regression to the mean.

These same criteria also reduce the potential impact of measurement variability.

The observed results also show that not the entire population experienced changes in their functional capacity after the COVID-19 pandemic lockdown. Among the alternative hypotheses underlying these results and analyzing their causes from the perspective of Antonovsky’s Pattern of Health (Rivera de los Santos et al., 2011; Seah et al., 2022), there may be a chronic adaptive resilience that these individuals developed over the course of their lives (poverty at birth, emigration, living in substandard housing, unemployment, women who were unable to work after marriage, low or no educational attainment, and low income), which would have given them a greater capacity to coping. A resilience they were able to apply in their daily lives to survive under persistently adverse living conditions, and that may have generated them psychosocial resources, and even other types (subsistence economy), which would fall under Antonovsky’s self-described General Resistance Resources, until achieving a Sense of Coherence (SOC) in their lives (Mijač et al., 2025), which would act as a catalyst for positive perspectives even in the face of adversity. Despite their functional dependence and advanced age, these individuals may have been able to draw upon the resources provided by this psychological construct (SOC)—which likely remained at least partially latent—to foster a sense of security and empowerment that enabled them to improve their functional capacity after the lockdown caused by the COVID-19 pandemic.

However, SOC, which is a psychological construct based on three basic principles—comprehensibility (understanding what is happening), meaning (what is happening motivates me to make changes), and manageability (I can handle the situation because I have the necessary tools to do so) (Mijač et al., 2025)—may not fully explain the functional response observed in this cohort during lockdown. In this response, in addition to psychological factors, biological and social factors may also play a role within a multifactorial biopsychosocial model. Therefore, to assert that the salutogenic model is responsible for the functional response observed in this cohort, specific studies would be required in a population with functional dependence to confirm whether, within this group of people, those with a strong SOC achieve better functional outcomes in stressful situations.

On the other hand, the resources provided by the SOC would partially overlap with the construct of “latent intrinsic capacity,” in which, within the biopsychosocial hypothesis, functional, physiological, and biological reserves, along with compensatory capacity and recovery potential, may also have played a role.

In this coping process, the effort these individuals had to make likely varied depending on the social support they had available (Yu et al., 2023). Among these forms of support, the availability of non-family caregivers was affected by the lockdown. This had a greater, though not significant, impact on people living independently in the community, who were the ones most likely to receive this type of in-home care. Although the loss of these caregivers was temporary and most were replaced by new caregivers, these changes and interactions with the new caregivers may also have contributed to the observed results.

On the other hand, as some studies have shown, the level of stress that people living with children or other people were exposed to may have differed from that of those living independently, since they were not alone or isolated (Blanco-Rambo et al., 2024; Gokseven et al., 2022; Moustakopoulou et al., 2023). This highlights the importance of social factors in health and wellbeing. And perhaps this nuance could partly explain why people living with their children or with others showed poorer outcomes regarding their ability to improve their functional status, or to improve their functional capacity to a level where they would not be classified as functionally dependent according to the criteria of the local protocol for functional dependence (Barthel >60), since they were being cared for and did not need to change their situation. However, when the data were analyzed, this impression was only a small part of the overall picture revealed by the results. Taking all the results into account, it was observed that individuals who did not improve their functional capacity—or who did not manage to cease being functionally dependent according to the criteria established by the local protocol—likely had worse baseline characteristics, which were reflected not only in a higher level of functional dependence but also in greater use of mobility aids, an inability to shop independently, having to live homebound, and having to live with children or other people. Regarding this last factor, although individuals living with their children did not have greater functional impairment prior to the lockdown that limited their ability to respond, they did share the other characteristics suggestive of greater personal impairment. This situation could indicate that, even though their level of care was not altered by the lockdown, factors other than functional capacity to perform basic activities of daily living also influenced this outcome.

Among these baseline characteristics was also the use of a wheelchair. Among those who used this mobility aid, two out of three lived with their children, and seven out of ten lived with children or other people. Being an older adult who uses a wheelchair has been associated with an increased risk of sarcopenia, and this factor limits both mobility and responsiveness (Maeda et al., 2017). Being in a wheelchair, or needing to use one for mobility, may have been a relevant factor in the differences observed based on housing situation.

This suggests that the direction of the relationship between these two variables, living with children and survival, could be subject to reverse causality. Supporting this possibility, in addition to the results already discussed, an analysis of the distribution of non-family caregivers revealed that the majority of private and internal caregivers provided their services to functionally dependent individuals living with their children, rather than to those living independently. Although most public caregivers did provide their services to functionally dependent individuals living independently, the fact that families caring for their dependent parents—and thus serving as essential 24-h caregivers—had, also hired a private and/or internal caregiver, and that this hiring was done by people residing in a socioeconomically disadvantaged environment, suggested that there was a real situation underlying this that justified this criterion, and that the dependent person likely required more assistance than what was provided by public services. However, confirming the reverse causality requires specific studies designed for that purpose.

The living arrangements within the community may also have influenced this outcome. Living homebound was more common among those living with their children, and, in the absence of differences in this living arrangement compared to living independently in terms of functional dependence, this permanent confinement suggested that other underlying processes or situations made it necessary, which in all likelihood influenced the results. Home confinement has been associated with an increased risk of mortality, regardless of functional decline and comorbidities (Soones et al., 2017). On the other hand, living homebound often entails greater difficulty performing instrumental activities of daily living, limited mobility, and greater frailty, compared to those who do not live confined to their homes (Asao et al., 2024).

At this point, we could also address the confounding bias due to indication, such that individuals classified as living with their children might have lower survival rates independently of this situation, due to greater pre-existing frailty, which has been partially addressed in this study in the previous points, and which is also suggested by the attitude of the family members of dependent individuals toward hiring caregivers. The exploratory analysis of multiple covariates and factors that could influence the results, conducted in this study, has made it possible to formulate these hypotheses and identify potential associations, which may serve as a basis for other, more specific studies.

Finally, with regard to the situation of living with one’s children or independently in the community, age may have been a relevant factor in the observed results. The group of dependent individuals living with their children had a higher mean age than those living independently. Although systematic reviews continue to show uncertainty regarding the association between living with one’s children and greater disability and mortality (van der Vorst et al., 2016), older age and female gender have been associated with greater disability, and both of these factors were present in this cohort. On the other hand, a higher mean age within an aging cohort would also imply higher natural mortality.

Thus, the initial adaptive response may not have depended only on a potential latent intrinsic capacity, but likely also on a context that could have favored, limited, or nullified that capacity for response.

With regard to intrinsic capacity, within the overall cohort and among the factors in which mobility played a role, the living arrangements in the community, defined by whether residents were homebound or able to leave, represented two distinct states of functionality. Living homebound meant greater limitation of vital space. This limitation probably contributed to the fact that this group did not show a significant improvement in functional capacity, nor did it imply a relevant level of people who managed to reverse their situation and become functionally independent again. On the contrary, although leaving home was not associated with a significant increase in the number of people who improved their functional capacity, it did so regarding those who managed to become functionally independent. Leaving the house was a factor that contributed to improve their functional capacity to a level of not being functionally dependent according to the local protocol criterion for functional dependence (Barthel >60), and when the ability to do so independently was maintained, it was associated with a higher probability of survival. This finding held true even when this ability extended to going for walks or engaging in leisure activities, and not just to necessary activities.

Being able to leave the house promotes social independence. In this study, this independence—defined as the ability to purchase the products necessary for daily sustenance either independently or under the supervision of another person—was associated both with the likelihood of improved functional capacity after lockdown and with reaching the level of no longer meet the local protocol criterion for functional dependence (Barthel >60). Furthermore, the ability to shop independently was associated with a higher probability of survival.

These associations between these instrumental activities of daily living—and even the one observed between the ability to go out versus being confined at home—and survival could also be subject to reverse causality. Most people who retained the ability to leave home to carry out necessary activities or to go for a walk—whether they could do so independently or, even, when accompanied by another person—had a lower level of functional dependence than those who could not perform these activities.

With regard to the presence of non-family caregivers, although empirically this factor tends to imply a greater need for care and, consequently, a poorer baseline condition, this factor was not associated with a higher level of functional dependence or with survival. Within an exploratory, hypothesis-generating context, multiple factors could have contributed to this result, ranging from the absence of children from the home due to work for a large part of the day and the consequent hiring of a caregiver to provide companionship rather than to meet care-related needs, to hiring a caregiver to alleviate loneliness, or, within a new model of cohabitation, sharing a residence with a person who lacks sufficient resources to have their own home and lives with the dependent older adult in exchange for their care.

Along the same lines, the use of mobility aids could indicate greater functional limitation. In this study, wheelchair use was associated with a higher level of functional dependence, while the use of crutches or a cane was associated with a lower level of functional dependence. These results may also have given rise to a reverse causality that could have played a role in the observed outcomes.

With regard to the mobility domain of the intrinsic capacity, analyzed through the activities of the Barthel Index in which mobility is an essential requirement for their performance, walking ability influenced the outcomes. This was evident both in relation to the improvement in functional capacity observed after the 100-day lockdown in the usual home, and in how this improvement resulted in functional recovery of sufficient magnitude to no longer meet the local protocol criterion for functional dependence (Barthel >60). However, this result was modulated by dependence on other basic and instrumental activities (Wang Q. et al., 2025; Yu et al., 2021a; Waris et al., 2022b), in which mobility played a role, as well as by the ability to perform basic activities of daily living where mobility did not play such a relevant role, but where other factors, such as postural balance, a parameter clearly associated with mobility in older adults, played a role (Campbell et al., 2023; Cui et al., 2025). In this regard, some studies have linked reduced mobility and difficulty with personal grooming to a diminished ability to leave the home and to shop independently (Schirghuber and Schrems, 2021). Furthermore, as other studies have also shown, limitations in shopping, toilet use and personal grooming, mobility, and dressing may be early indicators of functional disability, particularly in women (Zheng and Huang, 2023; Martín Moreno et al., 2026). Thus, the result depended on many more factors than the ability to walk itself, and conveyed information necessary for the assessment of this domain of intrinsic capacity, which became multidimensional. Finally, lower intrinsic capacity has been associated with greater dependence in both basic activities of daily living and instrumental activities (Sánchez-Sánchez et al., 2024a; Wang Q. et al., 2025), as well as with mortality (Beyene et al., 2024; Sánchez-Sánchez et al., 2024a; Wang Y. et al., 2025; Cao et al., 2024a). It also implies lower resilience. This approach made it possible to discern patterns that could be useful for health and social planning, establishing subgroups on which to conduct specific actions (Tang et al., 2025; Guaraldi et al., 2023).

With mobility being a key factor in the observed outcomes, an unexpected finding emerged in this study. One sub-aspect of walking ability, specifically walking to the toilet use, was the factor where being dependent was associated with the lowest probability of improving functional ability. In fact, none of the individuals who were dependent on others to use the toilet managed to regain the level of no longer meet the local protocol criterion for functional dependence (Barthel >60) after the lockdown. Other studies have found this limitation to be highly prevalent (Gyasi et al., 2022), particularly among women, who made up the majority of the cohort in this study.

Regarding the methodology used in this study, functional capacity was assessed using an index based on basic activities of daily living. These activities included the ability to walk, but also the ability to do so in specific situations, such as moving from a chair to a bed or *vice versa*, going up or down stairs, or going to the bathroom. It also included activities involving the combination of mobility and postural balance, incorporating balance into the assessment of mobility, a criterion justified by the relevance of this combination in older adults (Sánchez-Sánchez et al., 2024a; Cui et al., 2025). Instrumental activities of daily living were also analyzed, reflecting mobility capacity (Wang Q. et al., 2025), as it has been observed that intrinsic capacity is clearly associated with limitations in performing these instrumental activities (Yu et al., 2021a; Silva et al., 2023). This resulted in an expanded analysis of the “mobility” domain of intrinsic capacity, which in other studies has been found to be predictive of overall functionality (Gustafsson et al., 2022) and is associated with intrinsic capacity (Beard et al., 2019; Yang et al., 2024; Waris et al., 2022b). This criterion was justified because, when assessing the concept of intrinsic capacity in vulnerable groups, such as people with functional dependence, the criteria commonly used to assess mobility may not have the same level of practical usefulness (Nestola et al., 2020; Cao et al., 2024b). Specifically, in situations of advanced dependency, mobility is often reduced to a limited environment, such as the home itself, meaning that assessing intrinsic capacity based on walking ability would not provide the necessary nuances for a correct evaluation, requiring new approaches (Zhao et al., 2024; Bergland et al., 2017; Makino et al., 2020).

In a context where an aging population is expected to increase the prevalence of dependency, expanding and developing the current concept of intrinsic capacity and making it applicable to other settings and situations is a priority (Wang Q. et al., 2025; Wang Y. et al., 2025; Qi et al., 2024; Campbell et al., 2023). This priority includes its relevance to social and health planning (Nestola et al., 2020; Tang et al., 2025; Yu et al., 2023; Tan et al., 2024).

With regard to the statistical methodology, the risk of an event occurring was analyzed using Cox regression, a method that allows for the simultaneous evaluation of the effect of multiple explanatory variables on the risk of that event occurring, generating a hazard ratio (HR) for each variable that measures the increase or decrease in the risk of the event occurring for each variable (Gomes et al., 2026). Compared to other methods, Cox regression allows for the quantification of risk and the control of the impact or influence of confounding factors. The assessment of proportional hazards and the assumption that censoring is independent of the risk of experiencing the event, which are fundamental pillars of the validity of a Cox regression model, were addressed through Schoenfeld residual analysis, partial likelihood, and an analysis of the correlation between the variables under study and event and censoring status (Heller and Simonoff, 1992; McLernon et al., 2023). The main results from the Cox model are derived from direct-entry (*enter*) models specified according to clinical and social criteria, while the forward-selection analyses presented in Supplementary Appendix 4 are exploratory. Completing these analyses, the incremental predictive value of the functional change observed after lockdown with respect to long-term survival was estimated using the area under the ROC curve and the AIC and BIC criteria. The AIC indicated that the post-lockdown incremental model had greater predictive value for survival than the pre-lockdown baseline and clinical-social baseline models, a finding that, given the small sample size, will need to be confirmed in further studies. On the other hand, it should be noted that the formal interaction tests did not provide statistically significant evidence of an effect moderated by sex or age. Regarding the interaction between post-lockdown Barthel score and male sex, which yielded a p-value of 0.056, this result was not statistically significant; however, given the small sample size of the male group, it should be considered suggestive or exploratory, requiring larger samples to assess the presence of possible statistical significance. Also, within the methodological and statistical framework, the role of regression to the mean was addressed from various angles, among them the use of the pre-lockdown Barthel Index score as a control variable to adjust the final results for the groups, including this variable in the ANCOVA analysis (S and chwarz, 2025). In proposing the new construct “latent intrinsic capacity,” the three basic steps for its development were followed: 1: define the construct based on an observation that does not fit what has been previously described; 2: operationalize this definition—that is, convert this concept into data that is measurable, observable, and reproducible—so that it can be assessed in clinical practice; and 3: obtain empirical evidence from the study data that this initial hypothesis is likely well-founded and supported by results that could enable its reproducibility in other studies, which must independently confirm its existence and validity (S and chwarz, 2025). With regard to the longitudinal measurement properties and the robustness of the Barthel Index, several studies show how well this index performs in these areas (Caronni and Scarano, 2025).

As limitations of this study, the population size, the sizes of some subgroups, and the use of multiple tests mean that various analyses conducted are only exploratory in nature and prevent the generalization of the observed results, limiting their comparison to populations similar to the one studied or to the formulation of exploratory hypotheses, which must be confirmed in studies with larger cohorts. Furthermore, a comparison group without functional dependence was not included. On the other hand, the recruitment of this cohort after the end of the first wave of the COVID-19 pandemic introduces a survivor selection bias, which suggests that the inferences made in this study should be limited to this context. At the same time, the exact dates of the Barthel Index assessments prior to the lockdown were not available to determine the time interval between each participant’s pre- and post-lockdown assessments; therefore, it cannot be ruled out that this situation may have influenced the results. Also, with regard to sample size, the 2023 cohort used for the long-term follow-up of changes in functional trajectories represented only 42.5% of the initial cohort. However, the context in which this study was conducted is exceptional, and these results provide information that may be relevant if integrated with other studies conducted for the same purpose.

Furthermore, the study was conducted at a single health center located in a socioeconomically disadvantaged neighborhood with a population having a low level of education. Although the results have not been broken down by sex, the male group does not allow results to be extrapolated to this group, given its small size. This cohort consisted of patients from a single health center, the majority of whom were women. On the other hand, this situation is common in population studies, because of women’s longer life expectancy and the greater frequency with which they present functional limitations.

Furthermore, it cannot be ruled out that the advanced age of this cohort may have influenced the observed results, and that these results might differ in other cohorts with different age groups.

Although we have argued that a longer interval between the pre-lockdown and post-lockdown Barthel Index scores does not necessarily imply bias—since this interval does not depend on a lack of follow-up but rather on stability in the level of dependency—within the possible limitations of this study, we cannot rule out the possibility that variability in the timing of the initial pre-lockdown assessment using the Barthel Index represents a potential limitation.

Regarding functional changes between pre- and post-lockdown assessments, we cannot rule out the possibility that the observed results were influenced to some extent by natural recovery, deterioration associated with an underlying progressive disease, changes in caregiver support, or disruptions to routine healthcare during the lockdown, a factor that was not analyzed. Nor was the possible role of cognitive decline, dementia, depression, frailty, or pain analyzed. Additionally, the inherent variability of routine measurements or regression to the mean in repeated measurements may also have played a role.

On the other hand, although the threshold-based classification used is the one outlined in our health system’s protocol, and the observed results support this classification criterion, it cannot be ruled out that other thresholds might yield different results. Furthermore, other factors that were not analyzed or for which adjustment was incomplete could be responsible for residual confounding, which may have influenced the results.

Although in the great majority of cases, the ability to make the purchases necessary for their daily needs, the use of mobility aids, and the presence of personal caregivers were adequately documented in their medical records prior to the lockdown, and the data obtained retrospectively in the survey could be verified, it cannot be ruled out that in a small percentage of cases, memory errors may have occurred, leading to recall bias in both the pre- and post-lockdown data, and to misclassification. This percentage would also include cases in which this information was not provided by the dependent person themselves, but rather by a family member or internal caregiver.

On the other hand, the study was conducted under exceptional circumstances. The COVID-19 pandemic and social distancing led to a change in the baseline conditions of this cohort, which influenced the results. This influence may have been different in other groups and populations.

With regard to the new construct proposed as an exploratory hypothesis, “latent intrinsic capacity,” its validation requires specific studies. The results obtained do not allow us to rule out a partially circular reasoning, since better functional status is already an established prognostic marker.

Finally, the study focuses on the assessment of the mobility domain of intrinsic capacity, which has not been previously described. This approach, based on the use of the Barthel Index and various instrumental activities of daily living, will need to be validated in further studies.

## 11 Conclusion

After lockdown, it was observed that the functional trajectories of individuals in this cohort with functional dependence in performing basic activities showed functional improvement that was not previously predictable. This response was observed in the majority of that population and resulted in four out of every ten people no longer meeting the criterion established in the local protocol for defining functional dependence (Barthel ≤60). In this cohort, consisting of older adults from the Orcasitas neighborhood of Madrid (Spain) with registered functional dependence who survived until the post-lockdown assessment, attaining a Barthel Index score >60, corresponding to no longer meeting the local protocol criterion for functional dependence, was associated with subsequent all-cause mortality. Whether this threshold has prognostic significance beyond its protocol-defined role requires confirmation in independent cohorts.

The results obtained help highlight the complexity associated with functional dependence, a complexity based on observations such as the fact that improving functional capacity—or showing a clinically significant improvement in terms of score—was not sufficient in this study to demonstrate an association with survival. This association with survival only became evident when a certain threshold was exceeded, which in this study was set at a Barthel Index score greater than 60 points. This result supported the criteria established in our health system’s protocol to prioritize individuals with a Barthel Index score of ≤60 points. On the other hand, the exploratory finding that the change in functional capacity observed after lockdown had incremental prognostic value—which could improve the prediction of a clinical event at the 5-year follow-up—must be confirmed in specific studies with larger cohorts.

Among exploratory hypotheses, this study helps provide evidence that this theoretical mathematical threshold—which is little known and rarely used—could be more than just a cutoff point associated with establishing a certain level of functional dependence and could potentially offer added value in estimating the risk of mortality associated with functional dependence. This exploratory approach would broaden the perspective from which the Barthel Index is typically assessed. A score of 60 would no longer be merely a cutoff point established by health and social services to determine the presence of a level of functional dependence requiring care and follow-up, but also a potential dividing line between the possibility that functional recovery could have implications for survival. Although these hypotheses need to be confirmed by other independent studies, the potential applications in health and social planning would be evident.

On the other hand, greater functional independence in those activities on the Barthel Index, for which mobility is an essential requirement played a significant role in the results obtained. If confirmed in other studies with larger cohorts, consideration could be given to including these activities in the assessment of intrinsic capacity within the mobility domain, a criterion that would allow for the establishment of intervention subgroups based on profiles on which this study has conducted some exploratory analyses.

Finally, the observed trajectories may generate hypotheses regarding physical resilience and functional reserve, but independent prospective validation is required. The changes observed in the expected functional trajectories in this cohort raise the possibility of the resilience based on the ability to modify functional performance. Underlying this resilience, which is likely modulated by the prior level of functional dependence, mobility, and age, among other personal and social factors, an exploratory hypothesis is proposed: there may be a new construct—latent intrinsic capacity—which would form part of the mobility domain of intrinsic capacity.

## Data Availability

The original contributions presented in the study are included in the article/Supplementary Material, further inquiries can be directed to the corresponding author.

## Glossary

ADLs: Activities of Daily Living
AIC: Akaike Information Criterion
ANCOVA: Analysis of Covariance
AUC: Area under the ROC curve
BIC: Bayesian Information Criterion
BMI: Body Mass Index
CI: Confidence Interval
e-SOAP: electrónico-Seguimiento de Objetivos en Atención Primaria (IT—Primary Care Objectives Tracking). It is a computer-based dashboard and healthcare quality monitoring tool used in primary care
HR: Hazard Ratio
IADLs: Instrumental Activities of Daily Living
IHC: Individual Health Card. It allows the holder to receive free medical care and determines the copayment level for pharmacy services
n: Number of cases or observations
OR: Odds Ratio
p: Probability
ROC: Receiver Operating Characteristic
SCPCD: Community of Madrid’s Strategy for the Care of People with Chronic Diseases
SOC: Sense of Coherence of Antonovsky
SPSS: SPSS® 18.0 statistical package
U: U-statistic from the Mann-Whitney U test
VIF: Variance Inflation Factor
WHO: World Health Organization
**χ** ^**2**^: Chi-square test
Z: Z-statistic from the Mann-Whitney U test

### Terms

Community living arrangements: a way of life in the community related to whether or not a person has the functional capacity to leave their home to go for a walk, shop, or engage in leisure activities, as opposed to living homebound to one’s home
Homebound: a person living in the community whose daily life is restricted to their usual residence and who does not leave their home except under mandatory circumstances
Housing situations: A way of life in the community related to one’s living arrangement, whether living independently, with one’s children, or with other people who are not one’s children

## References

[B1] Álvarez M. A. Pinto N. S. (2023). Experiential COVID-19 factors predicting resilience among Spanish adults. BMC Psychol. 11 (1), 118. 10.1186/s40359-023-01131-4 PMC10108776 37069701

[B2] Asao A. Sone T. Fujita T. Hayashi H. Kurasawa S. Sumigawa K. (2024). Association between shopping assistance and functional decline in older residents with support levels under the long-term care insurance system in Japan: a retrospective, cross-sectional study. Geriatr. (Basel) 9 (6), 162. 10.3390/geriatrics9060162 PMC11728436 39727821

[B3] Bautmans I. Knoop V. Amuthavalli Thiyagarajan J. Maier A. B. Beard J. R. Freiberger E. (2022). WHO working group on vitality capacity. WHO working definition of vitality capacity for healthy longevity monitoring. Lancet Healthy Longev. 3 (11), e789–e796. 10.1016/S2666-7568(22)00200-8 36356628 PMC9640935

[B4] Beard J. R. Jotheeswaran A. T. Cesari M. Araujo de Carvalho I. (2019). The structure and predictive value of intrinsic capacity in a longitudinal study of ageing. BMJ Open 9 (11), e026119. 10.1136/bmjopen-2018-026119 31678933 PMC6830681

[B5] Bergland A. Jørgensen L. Emaus N. Strand B. H. (2017). Mobility as a predictor of all-cause mortality in older men and women: 11.8 year follow-up in the tromsø study. BMC Health Serv. Res. 17 (1), 22. 10.1186/s12913-016-1950-0 28068995 PMC5223479

[B6] Beyene M. B. Visvanathan R. Ahmed M. Benyamin B. Beard J. R. Amare A. T. (2024). Development and validation of an intrinsic capacity score in the UK biobank study. Maturitas 185, 107976. 10.1016/j.maturitas.2024.107976 38537388

[B7] Blanco-Rambo E. Bandeira-Guimarães M. Rambo E. S. Formighieri C. Steffens T. Cadore E. L. (2024). Effects of social distancing provoked by COVID-19 pandemic in the functional capacity and cognitive function in nonagenarians and centenarians. J. Body Mov. Ther. 37, 46–50. 10.1016/j.jbmt.2023.11.013 38432840

[B8] Boylan J. M. Vagnini K. M. Rush C. L. Larson E. K. Adams M. Wilson C. L. (2023). Meaning salience and meaning in life prospectively predict lower distress during the COVID-19 pandemic. Ann. Behav. Med. 57 (6), 483–488. 10.1093/abm/kaac079 36940243

[B9] Burnham K. P. Anderson D. R. (1998). Model Selection and Inference: A Practical Information-Theoretic Approach. New York: Springer-Verlag. 10.1007/978-1-4757-2917-7

[B10] Campbell C. L. Cadar D. McMunn A. Zaninotto P. (2023). Operationalization of intrinsic capacity in older people and its association with subsequent disability, hospital admission and mortality: results from the English longitudinal study of ageing. J. Gerontol. A Biol. Sci. Med. Sci. 78 (4), 698–703. 10.1093/gerona/glac250 PMC10061563 36512074

[B11] Cao X. Yi X. Chen H. Tian Y. Li S. Zhou J. (2024a). Prevalence of intrinsic capacity decline among community-dwelling older adults: a systematic review and meta-analysis. Aging Clin. Exp. Res. 36 (1), 157. 10.1007/s40520-024-02816-5 PMC11294388 39088112

[B12] Cao X. Tian Y. Chen H. Li S. Zhou J. (2024b). The global research trends on intrinsic capacity of older adults: a bibliometric and visual analysis of papers published during 2015-2023. J. Multidiscip. Healthc. 17, 3323–3339. 10.2147/JMDH.S471324 PMC11249103 39010933

[B13] Caronni A. Scarano S. (2025). Generalizability of the barthel index and the functional Independence measure: robustness of disability measures to differential item functioning. Disabil. Rehabil. 47 (8), 2134–2145. 10.1080/09638288.2024.2391554 39221560

[B14] Cesari M. Araujo de Carvalho I. Amuthavalli Thiyagarajan J. Cooper C. Martin F. C. Reginster J. Y. (2018). Evidence for the domains supporting the construct of intrinsic capacity. J. Gerontol. A Biol. Sci. Med. Sci. 73 (12), 1653–1660. 10.1093/gerona/gly011 29408961

[B15] Cheng Y. Li W. Xiao S. Chen Y. Qi X. (2025). Intrinsic capacity and its dimensions in relation to functional ability in older adults: a systematic review and meta-analysis. Arch. Gerontol. Geriatr. 135, 105860. 10.1016/j.archger.2025.105860 40344941

[B16] Chhetri J. K. Xue Q. L. Ma L. Chan P. Varadhan R. (2021). Intrinsic capacity as a determinant of physical resilience in older adults. J. Nutr. Health Aging 25 (8), 1006–1011. 10.1007/s12603-021-1629-z PMC8035602 34545921

[B18] Consejería de Políticas Sociales y Familia (2017). Estrategia de Atención a las Personas Mayores de la Comunidad de Madrid 2017-2021. Madrid: Consejería de Políticas Sociales y Familia.

[B17] Cui Y. Zhou J. Liu Q. Ye H. Liu B. (2025). The mediating role of intrinsic capacity in balance and falls among older adults. Sci. Rep. 15 (1), 11732. 10.1038/s41598-025-96081-9 PMC11972392 40188249

[B19] Gao S. Yu Y. Xu J. Zou L. Li H. Zhong Q. (2025). Effect size estimates of risk factors for the impaired intrinsic capacity among community-dwelling older adults: a systematic review and meta-analysis. J. Am. Med. Dir. Assoc. 26 (12), 105950. 10.1016/j.jamda.2025.105950 41120043

[B20] Gencer N. S. Çavdar S. Kayhan Koçak F. O. Erol F. Savas S. (2025). Cumulative effect of intrinsic capacity domains on geriatric syndromes and functionality in older inpatients. BMC Geriatr. 25 (1), 949. 10.1186/s12877-025-06697-9 PMC12645786 41291504

[B21] Gokseven Y. Ozturk G. Z. Karadeniz E. Sari E. Tas B. G. Ozdemir H. M. (2022). The fear of COVID-19 infection in older people. J. Geriatr. Psychiatry Neurol. 35 (3), 460–466. 10.1177/08919887211002651 33745357

[B22] Gomes A. Costa B. Nunes V. Coelho C. (2026). Cox regression in survival analysis: practical insights for clinicians. Acta Med. Port. 13. 10.20344/amp.23078 41687085

[B23] Grigoraş G. Ilie A. C. Turcu A. M. Albişteanu S. M. Lungu I. D. Ștefăniu R. (2025). Resilience and intrinsic capacity in older adults: a review of recent literature. J. Clin. Med. 14 (21), 7729. 10.3390/jcm14217729 41227125 PMC12608230

[B24] Guaraldi G. Milic J. Barbieri S. Marchiò T. Caselgrandi A. Motta F. (2023). Quality of life and intrinsic capacity in patients with post-acute COVID-19 syndrome is in relation to frailty and resilience phenotypes. Sci. Rep. 13 (1), 8956. 10.1038/s41598-023-29408-z PMC10235830 37268716

[B25] Gustafsson P. E. Nilsson I. San Sebastian M. (2022). Venerable vulnerability or remarkable resilience? A prospective study of the impact of the first wave of the COVID-19 pandemic and quarantine measures on loneliness in Swedish older adults with home care. BMJ Open 12 (5), e060209. 10.1136/bmjopen-2021-060209 PMC9130667 35613816

[B26] Gyasi R. M. Aboderin I. Asiki G. (2022). Prevalence and social risk factors of functional limitations among slum-dwelling older adults: findings from the Nairobi urban health and demographic surveillance system. Gerontol. Geriatr. Med. 8, 23337214221088700. 10.1177/23337214221088700 PMC9102122 35573080

[B27] Heller G. Simonoff J. S. (1992). Prediction in censored survival data: a comparison of the proportional hazards and linear regression models. Biometrics 48 (1), 101–115. 1581480

[B28] Kiah H. S. S. Chia J. L. Mahendran R. Yu J. (2022). Older adults' perceptions of government handling of COVID-19: predictors of protective behaviors from lockdown to post-lockdown. PLoS One 17 (2), e0263039. 10.1371/journal.pone.0263039 PMC8809562 35108322

[B29] Koivunen K. Lindeman K. Välimaa M. Rantanen T. (2024). Investigating resilience through intrinsic capacity networks in older adults. J. Gerontol. A Biol. Sci. Med. Sci. 79 (10), glae048. 10.1093/gerona/glae048 38366153 PMC11372706

[B30] Kok A. A. L. Huisman M. Giltay E. J. Lunansky G. (2025). Adopting a complex systems approach to functional ageing: bridging the gap between gerontological theory and empirical research. Lancet Healthy Longev. 6 (3), 100673. 10.1016/j.lanhl.2024.100673 39884294

[B31] Lambert L. S. Newman D. A. (2023). Construct development and validation in three practical steps: recommendations for reviewers. Organ. Res. Methods 26 (4), 574–607. 10.1177/10944281221115374

[B32] Li Y. Yang T. Wang X. He X. Dong J. Qian Q. (2024). The ability of decline in intrinsic capacity to indicate the risk of mortality in older adults: a meta-analysis. Maturitas 189, 108109. 10.1016/j.maturitas.2024.108109 39293255

[B33] Losada-Baltar A. Martínez-Huertas J. Á. Jiménez-Gonzalo L. Pedroso-Chaparro M. D. S. Gallego-Alberto L. Fernandes-Pires J. (2022). Longitudinal correlates of loneliness and psychological distress during the lockdown situation due to COVID-19. Effects of age and self-perceptions of aging. J. Gerontol. B Psychol. Sci. Soc. Sci. 77 (4), 652–660. 10.1093/geronb/gbab012 33438002 PMC7928595

[B34] Maeda K. Shamoto H. Wakabayashi H. Akagi J. (2017). Sarcopenia is highly prevalent in older medical patients with mobility limitation. Nutr. Clin. Pract. 32 (1), 110–115. 10.1177/0884533616680355 27881807

[B35] Mahoney F. I. Barthel D. W. (1965). Functional evaluation: the barthel index. Md State Med. J. 14, 61–65. 14258950

[B36] Makino K. Lee S. Bae S. Shinkai Y. Chiba I. Shimada H. (2020). Predictive validity of a new instrumental activities of daily living scale for detecting the incidence of functional disability among community-dwelling older Japanese adults: a prospective cohort study. Int. J. Environ. Res. Public Health 17 (7), 2291. 10.3390/ijerph17072291 32235309 PMC7177260

[B37] Martín Moreno V. Martínez Sanz M. I. Martín Fernández A. Guerra Maroto S. Sevillano Fuentes E. Pérez Rico E. (2025). Proposal for a revised barthel index classification based on mortality risk assessment in functional dependence for basic activities of daily living. Front. Public Health 12, 1478897. 10.3389/fpubh.2024.1478897 PMC11772177 39877909

[B38] Martín Moreno V. Pérez Rico E. Martín Fernández A. Hurtado Gallar J. Sánchez González I. Sánchez Rodríguez E. (2026). Association between the different basic activities of daily living on the barthel index and community living, use of mobility aids, and the survival at 5 years. Front. Public Health 14, 1825340. 10.3389/fpubh.2026.1825340 42326935 PMC13277337

[B39] McLernon D. J. Giardiello D. Van Calster B. Wynants L. van Geloven N. van Smeden M. (2023). Assessing performance and clinical usefulness in prediction models with survival outcomes: practical guidance for cox proportional hazards models. Ann. Intern Med. 176 (1), 105–114. 10.7326/M22-0844 36571841

[B40] Mijač S. Vitale K. Lončarek K. Slivšek G. (2025). How the salutogenic pattern of health reflects in type 2 diabetes mellitus: a narrative review. Diabetology 6 (11), 124. 10.3390/diabetology6110124

[B41] Morse J. L. Fischer I. C. Na P. J. Afari N. Pietrzak R. H. (2023). Functional decline during the COVID-19 pandemic among U.S. military veterans: results from the national health and resilience in veterans study. Int. J. Geriatr. Psychiatry 38 (12), e6040. 10.1002/gps.6040 38072628

[B42] Moustakopoulou L. Adamakidou T. Plakas S. Drakopoulou M. Apostolara P. Mantoudi A. (2023). Exploring loneliness, fear and depression among older adults during the COVID-19 era: a cross-sectional study in Greek provincial towns. Healthc. (Basel) 11 (9), 1234. 10.3390/healthcare11091234 PMC10178736 37174782

[B43] Nestola T. Orlandini L. Beard J. R. Cesari M. (2020). COVID-19 and intrinsic capacity. J. Nutr. Health Aging 24 (7), 692–695. 10.1007/s12603-020-1397-1 32744562 PMC8825255

[B44] Petashnick J. R. Shrira A. Hoffman Y. Palgi Y. Kavé G. Shmotkin D. (2022). Subjective age and late-life functional status: mediating and moderating effects. J. Gerontol. B Psychol. Sci. Soc. Sci. 77 (1), 61–70. 10.1093/geronb/gbab181 34608493

[B45] Qi Y. Schaap L. A. Schalet B. D. Hoogendijk E. O. Deeg D. J. H. Visser M. (2024). The development of intrinsic capacity measures for longitudinal research: the longitudinal aging study amsterdam. Exp. Gerontol. 197, 112599. 10.1016/j.exger.2024.112599 39366460

[B46] Rivera de los Santos F. Ramos Valverde P. Moreno Rodríguez C. Hernán García M. (2011). Análisis del modelo salutogénico en España: aplicación en salud pública e implicaciones para el modelo de activos en salud [Salutogenic model analysis in Spain: application in public health and implications for asset health model]. Rev. Esp. Salud Publica 85 (2), 129–139. 10.1590/S1135-57272011000200002 21826376

[B47] Schwarz W. (2025). The ancova model for comparing two groups: a tutorial emphasizing statistical distribution theory. Front. Psychol. 16, 1600764. 10.3389/fpsyg.2025.1600764 40443728 PMC12119598

[B48] Sánchez-Sánchez J. L. Lu W. H. Gallardo-Gómez D. Del Pozo Cruz B. de Souto Barreto P. Lucia A. (2024a). Association of intrinsic capacity with functional decline and mortality in older adults-a systematic review and meta-analysis of longitudinal studies. Lancet Healthy Longev. 5 (7), e480–e492. 10.1016/S2666-7568(24)0092-8 38945130

[B49] Sánchez-Sánchez J. L. Lu W. H. Gallardo-Gómez D. Del Pozo Cruz B. de Souto Barreto P. Lucia A. (2024b). Association of intrinsic capacity with functional decline and mortality in older adults: a systematic review and meta-analysis of longitudinal studies. Lancet Healthy Longev. 5 (7), e480–e492. 10.1016/S2666-7568(24)00092-8 38945130

[B50] Schirghuber J. Schrems B. (2021). Homebound: a concept analysis. Nurs. Forum 56 (3), 742–751. 10.1111/nuf.12586 33955012 PMC8453746

[B51] Seah B. Espnes G. A. Hong W. T. Wang W. (2022). Salutogenic healthy ageing programme embracement (SHAPE)- an upstream health resource intervention for older adults living alone and with their spouses only: complex intervention development and pilot randomized controlled trial. BMC Geriatr. 22 (1), 932. 10.1186/s12877-022-03605-3 36460959 PMC9719210

[B52] Shin J. H. Sagong H. Yoon J. Y. (2025). The relationship between trajectories of intrinsic capacity and differences in the risk of functional ability decline in community-dwelling older adults: a socio-ecological approach. Arch. Gerontol. Geriatr. 131, 105772. 10.1016/j.archger.2025.105772 39884088

[B53] Silva N. Rajado A. T. Esteves F. Brito D. Apolónio J. Roberto V. P. (2023). ALFAScore consortium. Measuring healthy ageing: current and future tools. Biogerontology 24 (6), 845–866. 10.1007/s10522-023-10041-2 37439885 PMC10615962

[B54] Song F. Chhetri J. K. Hu M. Li B. Xia J. Yin C. (2025). Mapping multimorbidity cluster-specific intrinsic capacity impairment patterns and mortality risks in a community-dwelling aging population cohort. J. Nutr. Health Aging 29 (11), 100687. 10.1016/j.jnha.2025.100687 PMC12482628 40974695

[B55] Soones T. Federman A. Leff B. Siu A. L. Ornstein K. (2017). Two-year mortality in homebound older adults: an analysis of the national health and aging trends study. J. Am. Geriatr. Soc. 65 (1), 123–129. 10.1111/jgs.14467 27641001 PMC5258674

[B56] Sterina E. Hermida A. P. Gerberi D. J. Lapid M. I. (2022). Emotional resilience of older adults during COVID-19: a systematic review of studies of stress and well-being. Clin. Gerontol. 45 (1), 4–19. 10.1080/07317115.2021.1928355 34080527 PMC8639827

[B57] Tan F. Wei X. Zhang J. Zhao Y. Tong X. Michel J. P. (2024). The assessment and detection rate of intrinsic capacity deficits among older adults: a systematic review and meta-analysis. BMC Geriatr. 24 (1), 485. 10.1186/s12877-024-05088-w 38831281 PMC11149255

[B58] Tang L. Binti Rasudin N. S. Dong Y. Yusuf A. (2025). Determinants of intrinsic capacity among older adults in low- and middle-income countries: a scoping review. Belitung Nurs. J. 11 (6), 661–673. 10.33546/bnj.4007 PMC12648234 41312041

[B59] Tiainen K. Luukkaala T. Hervonen A. Jylhä M. (2013). Predictors of mortality in men and women aged 90 and older: a nine-year follow-up study in the vitality 90+ study. Age Ageing 42 (4), 468–475. 10.1093/ageing/aft030 23486170

[B60] van der Vorst A. Zijlstra G. A. Witte N. Duppen D. Stuck A. E. Kempen G. I. (2016). Limitations in activities of daily living in community-dwelling people aged 75 and over: a systematic literature review of risk and protective factors. PLoS One 11 (10), e0165127. 10.1371/journal.pone.0165127 27760234 PMC5070862

[B61] Vermote B. Morbée S. Soenens B. Vansteenkiste M. Waterschoot J. Beyers W. (2023). How do late adults experience meaning during the COVID-19 lockdown? The role of intrinsic goals. J. Happiness Stud. 24 (5), 1759–1780. 10.1007/s10902-023-00657-z 37293323 PMC10157136

[B62] Wang Q. Liang Y. T. Xu Y. He Y. K. Li T. F. Qin Q. R. (2025a). Association of intrinsic capacity with ADL and IADL trajectories: evidence from a nationwide longitudinal study. Arch. Gerontol. Geriatr. 136, 105897. 10.1016/j.archger.2025.105897 40409130

[B63] Wang Y. Tang N. Shao M. Song J. Su Q. Gao Y. (2025b). Transitions and trajectories in intrinsic capacity states over time: a systematic review. BMC Geriatr. 25 (1), 407. 10.1186/s12877-025-05938-1 PMC12135290 40468176

[B64] Waris M. Upadhyay A. D. Chatterjee P. Chakrawarty A. Kumar P. Dey A. B. (2022a). Establishment of clinical construct of intrinsic capacity in older-adults and its prediction of functional decline. Clin. Interv. Aging 17, 1569–1580. 10.2147/CIA.S71793 PMC9594878 36304176

[B65] Waris M. Upadhyay A. D. Chatterjee P. Chakrawarty A. Kumar P. Dey A. B. (2022b). Establishment of clinical construct of intrinsic capacity in older adults and its prediction of functional decline. Clin. Interv. Aging 17, 1569–1580. 10.2147/CIA.S371793 PMC9594878 36304176

[B66] Westerhof G. J. Nehrkorn-Bailey A. M. Tseng H. Y. Brothers A. Siebert J. S. Wurm S. (2023). Longitudinal effects of subjective aging on health and longevity: an updated meta-analysis. Psychol. Aging 38 (3), 147–166. 10.1037/pag0000737 36972091 PMC10192139

[B67] Yang Y. Ma G. Wei S. Wei X. Yan B. Yuan Y. (2024). Adverse outcomes of intrinsic capacity in older adults: a scoping review. Arch. Gerontol. Geriatr. 120, 105335. 10.1016/j.archger.2024.105335 38422884

[B68] Yu R. Amuthavalli T. J. Leung J. Lu Z. Kwok T. Woo J. (2021a). Validation of the construct of intrinsic capacity in a longitudinal Chinese cohort. J. Nutr. Health Aging 25 (6), 808–815. 10.1007/s12603-021-1637-z PMC12876751 34179938

[B69] Yu J. Si H. Qiao X. Jin Y. Ji L. Liu Q. (2021b2021b). Predictive value of intrinsic capacity on adverse outcomes among community-dwelling older adults. Geriatr. Nurs. 42 (6), 1257–1263. 10.1016/j.gerinurse.2021.08.010 34555568

[B70] Yu R. Lai E. T. C. Leung G. Ho S. C. Woo J. (2022). Intrinsic capacity and 10-year mortality: findings from a cohort of older people. Exp. Gerontol. 167, 111926. 10.1016/j.exger.2022.111926 35963450

[B71] Yu R. Lai D. Leung G. Woo J. (2023). Trajectories of intrinsic capacity: determinants and associations with disability. J. Nutr. Health Aging 27 (3), 174–181. 10.1007/s12603-023-1881-5 36973922 PMC12880068

[B72] Zhao Y. Jiang Y. Tang P. Wang X. Guo Y. Tang L. (2024). Adverse health effects of declined intrinsic capacity in middle-aged and older adults: a systematic review and meta-analysis. Age Ageing 53 (7), afae162. 10.1093/ageing/afae162 39058916

[B73] Zheng W. Huang Z. (2023). Onset of ADL and IADL limitation among Chinese middle-aged and older adults. PLoS One 18 (7), e0287856. 10.1371/journal.pone.0287856 37459324 PMC10351716

