# Supplementary material for: Functional status following the COVID-19 lockdown and five-year survival among older adults with pre-existing functional dependence: a cohort study
Source: Front Aging. 2026 Oct 22;7:1868924. doi: 10.3389/fragi.2026.1868924 (PMC13736780; doi:10.3389/fragi.2026.1868924)
Supplement: Supplementary file 1 [file DataSheet1.pdf]

Name and surname:

CIPA:

Address:

Phone:

....

QUESTIONNAIRE

Question 1: Marital status. 1: Married. 2: Widower. 3: Single. 4: Separated or divorced.

Question 2: Education:

1.- Insufficient education:

1: Cannot read or write 2: No studies 3: Incomplete primary education

2.- Primary Education:

1: Elementary School. 2: Vocational Training

3.- Medium Studies:

1: High School. 2: Master's Degree. 3: Expert

4.- Higher Education: ....

Question 3: You live with your partner 1: YES; 2: NO

Question 4: Is your partner the primary caregiver 1: YES; 2: NO

Question 5: Is he the main caregiver of his partner 1: YES; 2: NO

Question 6: Number of residents in the home: .... Boys .... Women

Note: number of people who live regularly in the house, even if they are not registered.

Question 7: Total number of children: .... Men .... Women

Note: number of children that the dependent has had, whether natural, adopted or from another couple, whether or not they live at the home at the moment.

Question 8: Number of children living together: ....

Note: numbers of male children currently living in the dependent's home.

Question 9: Number of daughters living together: ....

Note: daughter numbers that currently live in the dependent's home.

Question 10: Does child 1 who lives with him work? 1: YES; 2: NO

Note: child 1 in relation to his age.

Question 11: Age of child 1 who lives together: ...... years

Question 12: Is the child 1 separated, divorced or similar? 1: YES; 2: NO

Question 13: Child 1 who lives with 1 has his or her own home: 1: YES; 2: NO

Question 14: Does child 2 who lives with work: 1: YES; 2: NO

Question 15: Age of child 2 who lives together: .... years.

Question 16: Is the child 2 separated, divorced or similar? 1: YES; 2: NO

Question 17: Child 2 who lives with has his or her own home: 1:YES; 2: NO

Question 18: Other cohabitants who children (specify relationship AND AGE) are not:

1. ……………………………………………………………………………………………………….
2. ……………………………………………………………………………………………………….
3. ……………………………………………………………………………………………………….
4. ……………………………………………………………………………………………………….
5. ……………………………………………………………………………………………………….
6. ……………………………………………………………………………………………………….

Question 19: Does the dependent private caregiver have: 1: YES; 2: NO

Note: private caregiver: any person who cares for the dependent on a paid basis and does not belong to the family, whether or not they are registered with Social Security.

Question 19.a: Is a private caregiver a internal caregiver? 1: YES; 2: NO

Question 20: Hours per week of private caregiver: .... hours

Question 21: Does the dependent caregiver of the public system 1: YES; 2: NO

Note: public system caregiver: any person who cares for the dependent in a remunerated way from public funds of the different administrations, whether this caregiver is a family member or an external person.

Question 22: Hours per week of public caregiver: .... hours

Question 23: Caregiver changes during the pandemic:

1: INT/PRIV/PUB suspension:

2: Decrease INT/PRIV/PUB hours:

3: Increase INT/PRIV/PUB hours:

4: Change of person performing INT/PRIV/PUB care:

Question 24: You are going to day center 1: YES; 2: NO

Question 25: You have telecare 1: YES; 2: NO

Question 26: Number of times you have used telecare between January 1 and today: ……. times

Question 27: Number of times you have used telecare between March 15 and today: ....times

Question 28: Healthcare 1: Public 2: Private 3: Both 4: MUFACE

Question 29: Architectural barriers:

1: None. 2: House without elevator. 3: Two-story house, live upstairs

Question 30: Do you have a pet 1: YES; 2: NO

Question 31: What pet...

Question 32: Take care of your pet 1: YES; 2: NO

BEFORE THE COVID-19 PANDEMIC:

Question 33: Pre-pandemic: Leave home **1: YES; 2: NO**

Note: ask if before the beginning of the confinement due to the Covid-19 pandemic (March 15, 2020) you left home, either alone or accompanied, for situations other than medical consultations, such as walking, going shopping, to the hairdresser, etc... This type of outing includes being in a wheelchair.

Question 34: Pre-pandemic: Usually leave the house

1: solo **2: accompanied** **3: does not come out**

Question 35: Pre-pandemic: Do you leave home if you don't have to go shopping

1: YES; 2: NO

Question 36: Pre-pandemic: Make purchases autonomously:

Note: "autonomous": when the clerk decides what to buy and performs the act of choosing and paying for the selected good.

1: YES; 2: NO

Question 37: Pre-pandemic: Make purchases autonomously but supervised

Note: "autonomous but supervised": when the clerk decides what to buy and performs the act of choice but cannot make the payment for the selected good due to cognitive or mechanical-functional problems.

1: YES; 2: NO

Question 38: Pre-pandemic: WHEELCHAIR: **1: YES; 2: NO**

Question 39: Pre-pandemic: WALKER: **1: YES; 2: NO**

Question 40: Pre-pandemic: CRUTCHES/CANE USE: **1: YES; 2: NO**

AFTER THE COVID-19 PANDEMIC:

Question 41: POST-PANDEMIC: Leave home

Note: ask if before the beginning of the confinement due to the pandemic (March 15, 2020) you left home, either alone or accompanied, for situations other than medical consultations, such as walking, going shopping, to the hairdresser, etc... This type of outing includes being in a wheelchair.

1: YES; 2: NO

Question 42: POST-PANDEMIC: Do you usually leave the house

1: solo 2: accompanied 3: does not come out

Question 43: POST-PANDEMIC: Do you leave home if you don't have to go shopping

1: YES; 2: NO

Question 44: POST-PANDEMIC: Make purchases autonomously

Note: "autonomous": when the clerk decides what to buy and performs the act of choosing and paying for the selected good.

1: YES; 2: NO

Question 45: POST-PANDEMIC: Make purchases autonomously but supervised

Note: "autonomous but supervised": when the clerk decides what to buy and performs the act of choice but cannot make the payment for the selected good due to cognitive or mechanical-functional problems.

1: YES; 2: NO

Question 46: POST-PANDEMIC: WHEELCHAIR: 1: YES; 2: NO

Question 47: POST-PANDEMIC: WALKER: 1: YES; 2: NO

Question 48: POST-PANDEMIC: USE OF CRUTCHES/CANE: 1: YES; 2: NO
