# Supplementary material for: Functional status following the COVID-19 lockdown and five-year survival among older adults with pre-existing functional dependence: a cohort study
Source: Front Aging. 2026 Oct 22;7:1868924. doi: 10.3389/fragi.2026.1868924 (PMC13736780; doi:10.3389/fragi.2026.1868924)
Supplement: Supplementary file 2 [file DataSheet2.pdf]

BARTHEL INDEX		
**Activities that Require Mobility to be Performed**		
**Activity**	**Description**	**Score**
**Walking - Moving Around**	Independent, can enter and exit using crutches-cane<br>Needs assistance from another person or uses a walker<br>Independent in a wheelchair (50 m)<br>Unable to get around	15<br>10<br>5<br>0
**Chair-to-Bed Transfers**	Independent<br>Needs minimal assistance, physical or verbal<br>Needs significant assistance, can remain seated<br>Totally dependent, cannot remain seated	15<br>10<br>5<br>0
**Going Up and Down Stairs**	Independent<br>Needs physical or verbal assistance; may use crutches-cane<br>Dependent	10<br>5<br>0
**Toilet Use**	Independent (entering-exiting, personal hygiene, dressing)<br>Needs assistance<br>Totally dependent	10<br>5<br>0
**Activities Requiring Mobility and Balance**		
**Dressing and undressing**	Independent, including buttons, zippers, and laces<br>Needs help, but can do at least half without help<br>Dependent	10<br>5<br>0
**Grooming**	Independent in washing hands, face, and teeth, combing hair, and shaving<br>Dependent	5<br>0
**Bathing**	Independent<br>Dependent	5<br>0
**Activities for which Mobility is not Essential**		
**Feeding**	Independent (able to use any utensil)<br>Needs help cutting, spreading jam or butter, using condiments, etc.<br>Dependent	10<br>5<br>0
**Bladder control**	Continent for at least 7 days<br>Occasionally incontinent (once or less per day)<br>Incontinent, or catheterized patient who is unable to change their bag	10<br>5<br>0
**Bowel control**	Continent<br>Occasionally incontinent (once a week or less)<br>Incontinent, or requires assistance in administering an enema	10<br>5<br>0

Barthel Index	
**Score**	**Classification of functional dependence level**
0-35	Severe Dependence
40-60	Moderate Dependence
