# Supplementary material for: Functional status following the COVID-19 lockdown and five-year survival among older adults with pre-existing functional dependence: a cohort study
Source: Front Aging. 2026 Oct 22;7:1868924. doi: 10.3389/fragi.2026.1868924 (PMC13736780; doi:10.3389/fragi.2026.1868924)
Supplement: Supplementary file 3 [file DataSheet3.pdf]

Appendix 3: Orcasitas Study: Descriptive and ANCOVA analysis.

Section 1: Orcasitas Study: Descriptive statistics for pre-lockdown Barthel scores regarding the outcomes "improvement or worsening in Barthel score after the Covid-19 pandemic lockdown."

Descriptive Statistics

	Orcasitas Study: Descriptive statistics for pre-lockdown Barthel scores regarding the outcomes "improvement or worsening in Barthel score after the Covid-19 pandemic lockdown."			Statistical	Standard error
Barthel Index Score Prior Lockdown	Improvement	Mean		44.19	2.098
		95% confidence interval for the mean	Lower limit	40.01	
			Upper limit	48.37	
		Mean trimmed at the 5%		45.67	
		Median		50.00	
		Variance		347.669	
		Standard deviation		18.646	
		Minimum		0	
		Maximum		60	
		Range		60	
		Interquartile range		25	
		Skewness		-1.057	.271
		Kurtosis		-.082	.535
	Worsening	Mean		43.79	3.054
		95% confidence interval for the mean	Lower limit	37.57	
			Upper limit	50.01	
		Mean trimmed at the 5%		45.04	
		Median		50.00	
		Variance		307.860	
		Standard deviation		17.546	
		Minimum		5	
		Maximum		60	
		Range		55	
		Interquartile range		28	
		Skewness		-1.060	.409
		Kurtosis		.015	.798

Section 2: ANCOVA for pre-lockdown Barthel scores regarding the outcomes "improvement or worsening in Barthel score after the Covid-19 pandemic lockdown."

Inter-subject factors

		Value label	N
Sex	1	Female	86
	2	Male	24

Descriptive statistics[to]

Dependent variable: Orcasitas study improves or worsens Barthel score due to lockdown

Sex	Mean	Standard deviation	N
Women	1.29	3.082	86
Male	1.29	3.052	24
Total	1.29	3.062	110

a. Weighted Least Squares Regression - Weighted by Orcasitas Study Pre-Lockdown Barthel Score

Levene Contrast on the Equality of Variances Error[a.b]

Dependent variable: Orcasitas study improves or worsens Barthel score due to lockdown

F	DL1	DL2	Sig.
.039	1	108	.843

Contrast the null hypothesis that the variance error of the dependent variable is the same across all groups.

a. Design: Intersection + Age + Sex

b. Weighted Least Squares Regression - Weighted by Orcasitas Study Pre-Lockdown Score

Evidence of inter-subject effects[c]

Dependent variable: Orcasitas study improves or worsens Barthel score due to confinement

Origin	Sum of squares type III	dl	Mean square	F	Sig.
Corrected model	38.617[a]	2	19.309	2.101	.127
Intersection	.000	1	.000	.000	.997
Age	38.551	1	38.551	4.195	.043
Sex	.255	1	.255	.028	.868
Error	983.363	107	9.190		
Total	9271.000	110			
Total corrected	1021.980	109			

a. R squared = .038 (R squared corrected = .020)

c. Weighted Least Squares Regression - Weighted by Orcasitas Study Pre-Lockdown Barthel Score

Evidence of inter-subject effects[c]

Dependent variable: Orcasitas study improves or worsens Barthel score due to lockdown

Origin	Partial eta squared	Non-centrality parameter Parameter	Observed power[b]
Corrected model	.038	4.202	.423
Intersection	.000	.000	.050
Age	.038	4.195	.528
Sex	.000	.028	.053

b. Calculated with alpha = .05

c. Weighted Least Squares Regression - Weighted by Orcasitas Study Pre-Lockdown Barthel Score

Estimates of parameters[c]

Dependent variable: Orcasitas study improves or worsens Barthel score due to lockdown

Parameter	B	Std error.	t	Sig.	95% confidence interval	
					Lower limit	Upper limit
Intersection	-.011	.640	-.017	.986	-1.280	1.258
Age	.015	.007	2.048	.043	.000	.029
[sex=1]	.018	.106	.167	.868	-.192	.227
[sex=2]	0[b]	.	.	.	.	.

Estimates of parameters[c]

Dependent variable: Orcasitas study improves or worsens Barthel score due to confinement

Parameter	Partial eta squared	Non-centrality parameter Parameter	Power Observed[a]
Intersection	.000	.017	.050
Age	.038	2.048	.528
[sex=1]	.000	.167	.053
[sex=2]	.	.	.

a. Calculated with alpha = .05
b. The parameter has been set to zero because it is redundant.
c. Weighted Least Squares Regression - Weighted by Orcasitas Study Pre-Lockdown Barthel Score

General estimable function[a,b]

Parameter	Contrast		
	L1	L2	L3
Intersection	1	0	0
Age	0	1	0
[sexo=1]	0	0	1
[sexo=2]	1	0	-1

a. Design: Intersection + Age + Sex
b. Weighted Least Squares Regression - Weighted by Orcasitas Study Pre-Lockdown Barthel Score

Contrast coefficients (L´Matrix)

Tests for misfit[b]

Dependent variable: Orcasitas study improves or worsens Barthel score due to lockdown

Origin	Sum of squares	dl	Mean square	F	Sig.	Partial eta squared
Lack of fit	285.162	39	7.312	.712	.874	.290
Pure error	698.201	68	10.268			

b. Weighted Least Squares Regression - Weighted by Orcasitas Study Pre-Lockdown Barthel Score

Tests for misfit[b]

Dependent variable: Orcasitas study improves or worsens Barthel score due to lockdown

Origin	Non-centrality parameter	Power Observed[a]
Lack of fit	27.773	.616

a. Calculated with alpha = .05

b. Weighted Least Squares Regression - Weighted by Orcasitas Study Pre-Lockdown Barthel Score

Custom Hypothesis Test

Contrast Results (K Matrix)[a]

Contrast of sex differences			Dependent variable
			Orcasitas study improves or worsens Barthel score due to lockdown
Level 2 - Level 1	Contrast estimation		-.018
	Hypothesized value		0
	Difference (Estimated - Hypothetical)		-.018
	Std error.		.106
	Sig.		.868
	95% confidence interval for difference	Lower limit	-.227
		Upper limit	.192

a. Weighted Least Squares Regression - Weighted by Orcasitas Study Pre-Lockdown Barthel Score

Test results[b]

Dependent variable: Orcasitas study improves or worsens Barthel score due to lockdown

Origin	Sum of squares	dl	Mean square	F	Sig.	Partial eta squared
Contrast	.255	1	.255	.028	.868	.000
Error	983.363	107	9.190			

b. Weighted Least Squares Regression - Weighted by Orcasitas Study Pre-Lockdpwn Barthel Score

Test results[b]

Dependent variable: Orcasitas study improves or worsens Barthel score due to lockdown

Origin	Non-centrality parameter	Power Observed[a]
Contrast	.028	.053

a. Calculated with alpha = .05

b. Weighted Least Squares Regression - Weighted by Orcasitas Study Pre-Lockdown Barthel Score

Estimated Marginal Means

Estimates[b]

Dependent variable: Orcasitas study improves or worsens Barthel score due to lockdown

Sex	Media	Std error.	95% confidence interval	
			Lower limit	Upper limit
Women	1.296[a]	.049	1.200	1.393
Male	1.279[a]	.094	1.093	1.464

a. The covariates that appear in the model are evaluated in the following values: Age = 86.08.

b. Weighted Least Squares Regression - Weighted by Orcasitas Study Pre-Lockdown Barthel Score

Pairwise comparisons[b]

Dependent variable: Orcasitas study improves or worsens Barthel score due to lockdown

(I)sex	(J)sex	Mean difference (I-J)	Std error.	Sig	95% confidence interval for difference[a]	
					Lower limit	Upper limit
Women	Male	.018	.106	.868	-.192	.227
Male	Female	-.018	.106	.868	-.227	.192

Based on the estimated marginal means.

a. Fit for multiple comparisons: Bonferroni.

b. Weighted Least Squares Regression - Weighted by Orcasitas Study Pre-Lockdown Barthel Score

Univariate contrasts[b]

Dependent variable: Orcasitas study improves or worsens Barthel score due to lockdown

	Sum of squares	dl	Mean square	F	Sig.	Partial eta squared
Contrast	.255	1	.255	.028	.868	.000
Error	983.363	107	9.190			

Each F-test contrasts the simple sex effect in each combination of levels with the rest of the effects shown.

b. Weighted Least Squares Regression - Weighted by Orcasitas Study Pre-Lockdown Barthel Score

Univariate contrasts[b]

Dependent variable: Orcasitas study improves or worsens Barthel score due to lockdown

	Non-centrality parameter	Power Observed[a]
Contrast	.028	.053

Each F-test contrasts the simple sex effect in each combination of levels with the rest of the effects shown.

a. Calculated with alpha = .05

b. Weighted Least Squares Regression - Weighted by Orcasitas Study Pre-Lockdown Barthel Score

Section 3: Orcasitas Study: Descriptive statistics for "Intensity of improvement in functional capacity to perform basic activities of daily living following the Covid-19 pandemic lockdown." The degree of improvement was classified into two levels. according to the criteria for clinical significance outlined in the Materials and Methods section: 1: improvement of 5 to 20 points; 2: improvement of 25 to 95 points. A score of 95 points represents the maximum possible level of improvement on a scale of 0 to 100 with 5-point intervals."

Descriptive Statistics

	Orcasitas Study: Descriptive statistics for "Intensity of improvement in functional capacity to perform basic activities of daily living following the Covid-19 pandemic lockdown."			Statistical	Standard error
Barthel Index Score Prior Lockdown	Improves their Barthel Index score by 5 to 20 points	Mean		48.46	2.762
		95% confidence interval for the mean	Lower limit	42.87	
			Upper limit	54.05	
		Mean trimmed at the 5%		50.38	
		Median		55.00	
		Variance		297.571	
		Standard deviation		17.250	
		Minimum		0	
		Maximum		60	
		Range		60	
		Interquartile range		20	
		Skewness		-1.667	.378
		Kurtosis		1.871	.741
	Improves their Barthel Index score by 25 to 95 points	Mean		40.03	3.039
		95% confidence interval for the mean	Lower limit	33.88	
			Upper limit	46.17	
		Mean trimmed at the 5%		41.08	
		Median		45.00	
		Variance		369.358	
		Standard deviation		19.219	
		Minimum		0	
		Maximum		60	
		Range		60	
		Interquartile range		34	
		Skewness		-.676	.374
		Kurtosis		-.758	.733

Section 4: Orcasitas Study: Descriptive statistics on the "Magnitude of change in Barthel Index scores at the individual level between the pre-lockdown baseline and the assessments conducted after the lockdown and in 2023":

Statisticians

		Differences between pre-lockdown Barthel and Barthel June 2020	Differences between pre-lockdown Barthel score and Barthel 2023	Differences between Barthel score June 2020 and June 2023
N	Valid	127	54	54
	Lost	0	73	73

Orcasitas study: differences between pre-lockdown Barthel and Barthel June 2020

		Frequency	Percentage	Valid percentage	Cumulative percentage
Valid	-40	1	.8	.8	.8
	-35	1	.8	.8	1.6
	-30	4	3.1	3.1	4.7
	-25	2	1.6	1.6	6.3
	-20	3	2.4	2.4	8.7
	-15	2	1.6	1.6	10.2
	-10	10	7.9	7.9	18.1
	-5	10	7.9	7.9	26.0
	0	15	11.8	11.8	37.8
	5	8	6.3	6.3	44.1
	10	11	8.7	8.7	52.8
	15	11	8.7	8.7	61.4
	20	9	7.1	7.1	68.5
	25	16	12.6	12.6	81.1
	30	7	5.5	5.5	86.6
	35	6	4.7	4.7	91.3
	40	3	2.4	2.4	93.7
	45	2	1.6	1.6	95.3
	50	1	.8	.8	96.1
	55	1	.8	.8	96.9
	60	1	.8	.8	97.6
	65	1	.8	.8	98.4
	70	1	.8	.8	99.2
	75	1	.8	.8	100.0
	Total	127	100.0	100.0	

Differences between pre-lockdown Barthel score and Barthel 2023

		Frequency	Percentage	Valid percentage	Cumulative percentage
Valid	-65	1	.8	1.9	1.9
	-50	1	.8	1.9	3.7
	-45	1	.8	1.9	5.6
	-40	1	.8	1.9	7.4
	-35	2	1.6	3.7	11.1
	-30	1	.8	1.9	13.0
	-20	2	1.6	3.7	16.7
	-15	2	1.6	3.7	20.4
	-10	3	2.4	5.6	25.9
	-5	4	3.1	7.4	33.3
	0	9	7.1	16.7	50.0
	5	6	4.7	11.1	61.1
	10	5	3.9	9.3	70.4
	15	2	1.6	3.7	74.1
	20	6	4.7	11.1	85.2
	25	3	2.4	5.6	90.7
	30	3	2.4	5.6	96.3
	35	1	.8	1.9	98.1
	60	1	.8	1.9	100.0
	Total	54	42.5	100.0	
Lost	System	73	57.5		
Total		127	100.0		

Differences between Barthel scores June 2020 and June 2023

		Frequency	Percentage	Valid percentage	Cumulative percentage
Valid	-55	2	1.6	3.7	3.7
	-50	3	2.4	5.6	9.3
	-40	2	1.6	3.7	13.0
	-35	4	3.1	7.4	20.4
	-30	2	1.6	3.7	24.1
	-25	4	3.1	7.4	31.5
	-20	4	3.1	7.4	38.9
	-15	8	6.3	14.8	53.7
	-10	3	2.4	5.6	59.3
	-5	6	4.7	11.1	70.4
	0	4	3.1	7.4	77.8
	5	6	4.7	11.1	88.9
	10	3	2.4	5.6	94.4
	15	2	1.6	3.7	98.1
	45	1	.8	1.9	100.0
	Total	54	42.5	100.0	
Lost	System	73	57.5		
Total		127	100.0		

Case Processing Summary

	Sex	Cases					
		Valid		Lost		Total	
		N	Percentage	N	Percentage	N	Percentage
Differences between pre-lockdown Barthel score and Barthel score June 2020	Female	100	100.0%	0	.0%	100	100.0%
	Male	27	100.0%	0	.0%	27	100.0%

Descriptives

	Sex			Statistician	Std error.
Differences between pre-lockdown Barthel score and Barthel score June 2020 (after lockdown)	Female	Mean		10.79	2.169
		Confidence interval for the mean at 95%	Lower limit		
			Upper limit		
		Average cut to 5%		10.50	
		Medium		10.00	
		Variance		470.430	
		Desv. typ.		21.689	
		Minimum		-40	
		Maximum		76	
		Range		116	
		Interquartile Amplitude		30	
		Asymmetry		.218	.241
		Curtosis		.351	.478
	Male	Mean		12.41	4.112
		Confidence interval for the mean at 95%	Lower limit		
			Upper limit		
		Average cut to 5%		11.71	
		Medium		15.00	
		Variance		456.481	
		Desv. typ.		21.365	
		Minimum		-30	
		Maximum		70	
		Range		100	
		Interquartile Amplitude		30	
		Asymmetry		.442	.448
		Curtosis		.954	.872

M-Estimators

	Sex	Huber's M-estimator[a]	Tukey's biweighted[b]	Hampel M-Estimator[c]	Andrews[d]
Differences between pre-lockdown Barthel score and Barthel score June 2020	Female	10.44	10.10	10.54	10.07
	Male	13.04	12.25	11.81	12.36

a. The weighting constant is 1.339.

b. The weighting constant is 4.685.

c. The weighting constants are 1.700. 3.400 and 8.500.

d. The weighting constant is 1.340*pi.

Percentiles

		Sex	Percentiles						
			5	10	25	50	75	90	95
Weighted Average (Definition 1)	Differences between pre-lockdown Barthel score and Barthel score June 2020	Female	-29.75	-15.00	-5.00	10.00	25.00	35.00	45.00
		Male	-26.00	-12.00	-5.00	15.00	25.00	34.00	62.00
Tukey's Kisses	Differences between pre-lockdown Barthel score and Barthel score June 2020	Women			-5.00	10.00	25.00		
		Male			-2.50	15.00	25.00		

Case Processing Summary

	Sex	Cases					
		Valid		Lost		Total	
		N	Percentage	N	Percentage	N	Percentage
Differences between pre-lockdown Barthel score and Barthel score 2023	Female	45	45.0%	55	55.0%	100	100.0%
	Male	9	33.3%	18	66.7%	27	100.0%

Descriptives

	Sex			Statistician	Std error.
Differences between pre-lockdown Barthel score and Barthel score 2023	Female	Mean		-.02	3.509
		Confidence interval for the mean at 95%	Lower limit		
			Upper limit		
		Average cut to 5%		.54	
		Medium		.00	
		Variance		554.113	
		Desv. typ.		23.540	
		Minimum		-65	
		Maximum		60	
		Range		125	
		Interquartile Amplitude		23	
		Asymmetry		-.531	.354
		Curtosis		1.072	.695
	Male	Media		8.33	6.346
		Confidence interval for the mean at 95%	Lower limit		
			Upper limit		
		Average cut to 5%		8.70	
		Medium		10.00	
		Variance		362.500	
		Desv. typ.		19.039	
		Minimum		-20	
		Maximum		30	
		Range		50	
		Interquartile Amplitude		38	
		Asymmetry		-.212	.717
		Curtosis		-1.694	1.400

M-Estimators

	Sex	Huber's M-estimator[a]	Tukey's biweighted[b]	Hampel M-Estimator[c]	Andrews[d]
Differences between pre-lockdown Barthel score and Barthel score 2023	Female	2.80	5.37	3.50	5.48
	Male	8.53	8.61	8.33	8.61

a. The weighting constant is 1.339.

b. The weighting constant is 4.685.

c. The weighting constants are 1.700. 3.400 and 8.500.

d. The weighting constant is 1.340*pi.

Percentiles

		Sex	Percentiles						
			5	10	25	50	75	90	95
Weighted Average (Definition 1)	Differences between pre-lockdown Barthel score and Barthel score 2023	Female	-47.30	-37.00	-7.50	.00	15.00	25.00	33.50
		Male	-20.00	-20.00	-10.00	10.00	27.50		
Tukey's Kisses	Differences between pre-lockdown Barthel score and Barthel score 2023	Women			-5.00	.00	15.00		
		Male			-10.00	10.00	25.00		

Case Processing Summary

	Sex	Cases					
		Valid		Lost		Total	
		N	Percentage	N	Percentage	N	Percentage
Differences between Barthel score June 2020 after lockdown and Barthel score 2023	Female	45	45.0%	55	55.0%	100	100.0%
	Male	9	33.3%	18	66.7%	27	100.0%

Descriptives

Sex				Statistician	Std error.
Differences between Barthel score June 2020 after lockdown and Barthel score June 2023	Female	Mean		-14.11	3.143
		Confidence interval for the mean at 95%	Lower limit	-20.45	
			Upper limit	-7.78	
		Average cut to 5%		-14.23	
		Medium		-15.00	
		Variance		444.646	
		Desv. typ.		21.087	
		Minimum		-55	
		Maximum		45	
		Range		100	
		Interquartile Amplitude		33	
		Asymmetry		.064	.354
		Curtosis		.212	.695
	Male	Media		-14.44	5.679
		Confidence interval for the mean at 95%	Lower limit	-27.54	
			Upper limit	-1.35	
		Average cut to 5%		-13.83	
		Medium		-15.00	
		Variance		290.278	
		Desv. typ.		17.038	
		Minimum		-50	
		Maximum		10	
		Range		60	
		Interquartile Amplitude		20	
		Asymmetry		-.902	.717
		Curtosis		1.851	1.400

M-Estimators

	Sex	Huber's M-estimator[a]	Tukey's biweighted[b]	Hampel M-Estimator[c]	Andrews[d]
Differences between Barthel score June 2020 after lockdown and Barthel score June 2023	Female	-13.36	-13.88	-13.87	-13.93
	Male	-12.86	-11.31	-12.71	-11.20

a. The weighting constant is 1.339.

b. The weighting constant is 4.685.

c. The weighting constants are 1.700. 3.400 and 8.500.

d. The weighting constant is 1.340*pi.

Percentiles

		Sex	Percentiles						
			5	10	25	50	75	90	95
Weighted Average (Definition 1)	Differences between Barthel score June 2020 after lockdown and Barthel score June 2023	Female	-53.50	-44.00	-30.00	-15.00	2.50	10.00	15.00
		Male	-50.00	-50.00	-22.50	-15.00	-2.50	.	.
Tukey's Kisses	Differences between Barthel score June 2020 after lockdown and Barthel score June 2023	Women			-30.00	-15.00	.00		
		Male			-20.00	-15.00	-5.00		

Section 5: Orcasitas Study: Descriptive statistics for "Remains functionally dependent after lockdown due to the Covid-19 lockdown (Barthel score of 60 or lower) versus no longer meeting the criteria for functional dependence established in the protocol (Barthel score >60)."

Descriptive Statistics

	Orcasitas Study: Descriptive statistics for "After lockdown due to the Covid-19 pandemic. the individual remains functionally dependent or has a functional capacity score above 60 on the Barthel Index."			Statistical	Standard error
Barthel Index Score Prior Lockdown	Barthel >60 after lockdown	Mean		35.86	2.388
		95% confidence interval for the mean	Lower limit	31.10	
			Upper limit	40.62	
		Mean trimmed at the 5%		36.51	
		Median		40.00	
		Variance		410.713	
		Standard deviation		20.266	
		Minimum		0	
		Maximum		60	
		Range		60	
		Interquartile range		35	
		Skewness		-.418	.283
		Kurtosis		-1.179	.559
	Remains functionally dependent	Mean		52.98	1.564
		95% confidence interval for the mean	Lower limit	49.85	
			Upper limit	56.12	
		Mean trimmed at the 5%		54.70	
		Median		60.00	
		Variance		134.574	
		Standard deviation		11.601	
		Minimum		9	
		Maximum		60	
		Range		51	
		Interquartile range		10	
		Skewness		-2.324	.322
		Kurtosis		5.867	.634
