# Supplementary material for: Functional status following the COVID-19 lockdown and five-year survival among older adults with pre-existing functional dependence: a cohort study
Source: Front Aging. 2026 Oct 22;7:1868924. doi: 10.3389/fragi.2026.1868924 (PMC13736780; doi:10.3389/fragi.2026.1868924)
Supplement: Supplementary file 4 [file DataSheet4.pdf]

Appendix 4: Cox Regression and Descriptive Analysis Related to Survival:

Section 1: "Data for Each Covariate Using Cox Regression":

Section 1-A: Improvement or worsening of the Barthel Index score following the Covid-19 pandemic lockdown:

Case Process Overview

		N	Percentage
Cases available in the analysis	Event[a]	69	54.3%
	Censored	43	33.9%
	Total	112	88.2%
Excluded cases	Cases with missing values	15	11.8%
	Cases with negative time	0	.0%
	Cases censored before the event earlier in a stratum	0	.0%
	Total	15	11.8%
Total		127	100.0%

a. Dependent variable: survival in months up to 60 months

Encodings of categorical variables[b]

		Frequency	(1)
Barthel improves or worsens	1=improvement	79	-.500
	2=Worsens	33	.500

a. Encoding Difference Parameters

b. Category variable: Barthel improves or worsens after lockdown (Orcasitas Study improves or worsens Barthel score after lockdown)

Status of stratum[a]

Stratum	Layer labels	Event	Censored	Percentage censored
1	Barthel severe dependence	26	6	18.8%
2	Barthel moderate dependence	43	37	46.3%
Total		69	43	38.4%

a. The strata variable is Orcasitas study Barthel level cut-off point 40 pre-lockdown

Status of stratum[a]

Stratum	Layer labels	Event	Censored	Percentage censored
1	Barthel severe dependence	26	6	18.8%
2	Barthel moderate dependence	43	37	46.3%
Total		69	43	38.4%

Encodings of categorical variables[b]

		Frequency	(1)
Barthel improves or worsens	1=improvement	79	1
	2=Worsens	33	0

a. Coding Indicator Parameters

b. Category variable: Barthel improves or worsens (Orcasitas study improves or worsens Barthel score due to confinement)

Omnibus tests on model coefficients

-2 Log of likelihood
499.777

Omnibus tests on model a coefficients

-2 Log of likelihood	Global (score)			Change from the previous step		
	Chi-square	dl	Sig.	Chi-square	dl	Sig.
497.914	1.957	1	.162	1.863	1	.172

a. Initial block number 1. Method = Enter

Omnibus tests on model a coefficients

Change from the previous block		
Chi-square	dl	Sig.
1.863	1	.172

a. Initial block number 1. Method = Enter

Variables in the equation

	B	SE	Wald	dl	Sig.	Exp(B)	95.0% CI for Exp(B) Lower	95.0% CI for Exp(B) Upper
Barthel score improves or worsens	-.354	.254	1.937	1	.164	.702	.427	1.155

Means of the covariates

	Mean
Barthel score improves or worsens	.705

Section 1-B: Functional dependence-not-dependence after lockdown according to local protocol covariate:

Case Process Overview

		N	Percentage
Cases available in the analysis	Event[a]	78	61.4%
	Censored	49	38.6%
	Total	127	100.0%
Excluded cases	Cases with missing values	0	.0%
	Cases with negative time	0	.0%
	Cases censored before the event earlier in a stratum	0	.0%
	Total	0	.0%
Total		127	100.0%

a. Dependent variable: survival in months up to 60 months

Status of stratum[a]

Stratum	Layer labels	Event	Censored	Percentage censored
1	Barthel severe dependence	32	6	15.8%
2	Barthel moderate dependence	46	43	48.3%
Total		78	49	38.6%

Case Process Overview

		N	Percentage
Cases available in the analysis	Event[a]	78	61.4%
	Censored	49	38.6%
	Total	127	100.0%
Excluded cases	Cases with missing values	0	.0%
	Cases with negative time	0	.0%
	Cases censored before the event earlier in a stratum	0	.0%
	Total	0	.0%
Total		127	100.0%

a. The strata variable is Orcasitas study Barthel level cut-off point 40 pre-lockdown

Encodings of categorical variables[b]

		Frequency	(1)
Barthel maintains dependence or not post-lockdown	1=Dependent: Barthel 60 or lower	72	1
	2=Not-Dependent: Barthel higher than 60	55	0

a. Coding Indicator Parameters

b. Category variable: Barthel maintains dependence or not post-lockdown (Orcasitas Study maintains or does not maintain dependence after lockdown)

Omnibus tests on model coefficients

-2 Log of likelihood
578.416

Omnibus tests on model a coefficient

-2 Log of likelihood	Global (score)			Change from the previous step		
	Chi-square	dl	Sig.	Chi-square	dl	Sig.
574.164	4.184	1	.041	4.251	1	.039

a. Initial block number 1. Method = Enter

Omnibus tests on model a coefficient

Change from the previous block		
Chi-square	dl	Sig.
4.251	1	.039

a. Initial block number 1. Method = Enter

Variables in the equation

	B	SE	Wald	dl	Sig.	Exp(B)	95.0% CI for Exp(B) Lower	95.0% CI for Exp(B) Upper
Maintains dependence or not post-lockdown	.528	.260	4.117	1	.042	1.695	1.018	2.823

Means of the covariates

	Mean
Maintains dependence or not post-lockdown	.567

Section 1-C: Level of improvement in the Barthel Index score after lockdown covariate:

Case Process Overview

		N	Percentage
Cases available in the analysis	Event[a]	45	35.4%
	Censored	34	26.8%
	Total	79	62.2%
Excluded cases	Cases with missing values	48	37.8%
	Cases with negative time	0	.0%
	Cases censored before the event earlier in a stratum	0	.0%
	Total	48	37.8%
Total		127	100.0%

a. Dependent variable: survival in months up to 60 months

Status of stratum[a]

Stratum	Layer labels	Event	Censored	Percentage censored
1	Barthel severe dependence	17	5	22.7%
2	Barthel moderate dependence	28	29	50.9%
Total		45	34	43.0%

Case Process Overview

		N	Percentage
Cases available in the analysis	Event[a]	45	35.4%
	Censored	34	26.8%
	Total	79	62.2%
Excluded cases	Cases with missing values	48	37.8%
	Cases with negative time	0	.0%
	Cases censored before the event earlier in a stratum	0	.0%
	Total	48	37.8%
Total		127	100.0%

a. The strata variable is Orcasitas study Barthel level cut-off point 40 pre-lockdown

Encodings of categorical variables[b]

		Frequency	(1)
Two levels improve Barthel score[to]	1=improvement between 5 and 20 points	39	1
	2=improvement between 25 and 95 points	40	0

a. Coding Indicator Parameters

b. Category variable: two-level Barthel score improvement (5-20 points vs. 25-95 points. cut-off point 25)

Omnibus tests on model coefficients

-2 Log of likelihood
298.211

Omnibus tests on model a coefficient

-2 Log of Likelihood	Global (score)			Change from the previous step		
	Chi-square	dl	Sig.	Chi-square	dl	Sig.
291.483	6.818	1	.009	6.728	1	.009

a. Initial block number 1. Method = Enter

Omnibus tests on model a coefficient

Change from the previous block		
Chi-square	dl	Sig.
6.728	1	.009

a. Initial block number 1. Method = Enter

Variables in the equation

	B	SE	Wald	dl	Sig.	Exp(B)	95.0% CI for Exp(B)	
							Lower	Upper
Two Levels Improve Barthel Score (5-20 vs 25-95)	.831	.325	6.517	1	.011	2.295	1.213	4.343

Means of the covariates

	Mean
Two Levels Improve Barthel Score	.494

Section 2: Descriptive Analysis Related to Survival:

Table E1: Orcasitas Study: Descriptive analysis regarding survival for the primary covariate "improvement or worsening of the Barthel Index score after lockdown. compared to the pre-lockdown score."

Descriptive Statistics

	Orcasitas Study: Descriptive analysis regarding survival for the primary covariate "improvement or worsening of the Barthel Index score after lockdown," compared to the pre-lockdown score			Statistical	Standard error
survival in months up to 60 months	Worsening	Mean		35.24	3.631
		95% confidence interval for the mean	Lower limit	27.85	
			Upper limit	42.64	
		Mean trimmed at the 5%		35.41	
		Median		36.00	
		Variance		435.189	
		Standard deviation		20.861	
		Minimum		6	
		Maximum		61	
		Range		55	
		Interquartile range		46	
		Skewness		.042	.409
		Kurtosis		-1.652	.798
	Improvement	Mean		40.61	2.471
		95% confidence interval for the mean	Lower limit	35.69	
			Upper limit	45.53	
		Mean trimmed at the 5%		41.50	
		Median		45.00	
		Variance		482.216	
		Standard deviation		21.959	
		Minimum		3	
		Maximum		61	
		Range		58	
		Interquartile range		45	
		Skewness		-.471	.271
		Kurtosis		-1.472	.535

Table E2: Orcasitas Study: Descriptive analysis regarding survival for the primary covariate "level of improvement in the Barthel Index score after lockdown".

Descriptive Statistics

	Orcasitas Study: Descriptive analysis regarding survival for the primary covariate "level of improvement in the Barthel Index score after lockdown"			Statistical	Standard error
survival in months up to 60 months	Improve between 5 and 20 points	Mean		36.51	3.545
		95% confidence interval for the mean	Lower limit	29.34	
			Upper limit	43.69	
		Mean trimmed at the 5%		36.99	
		Median		37.00	
		Variance		489.993	
		Standard deviation		22.136	
		Minimum		3	
		Maximum		61	
		Range		58	
		Interquartile range		47	
		Skewness		-.174	.378
		Kurtosis		-1.611	.741
	Improve between 25 and 95 points	Mean		44.60	3.369
		95% confidence interval for the mean	Lower limit	37.79	
			Upper limit	51.41	
		Mean trimmed at the 5%		45.75	
		Median		61.00	
		Variance		453.887	
		Standard deviation		21.305	
		Minimum		7	
		Maximum		61	
		Range		54	
		Interquartile range		39	
		Skewness		-.821	.374
		Kurtosis		-1.088	.733

Table E3: Orcasitas Study: Descriptive analysis regarding survival for the primary covariate “functional dependence or has no functional dependency according the local protocol criterion for functional dependence (Barthel >60).”

Descriptive Statistics

	Orcasitas Study: Descriptive analysis regarding survival for the primary covariate “functional dependence-independence after lockdown according to our protocol”			Statistical	Standard error
survival in months. up to 60 months	Dependent: Barthel 60 or lower	Mean		34.13	2.548
		95% confidence interval for the mean	Lower limit	29.04	
			Upper limit	39.21	
		Mean trimmed at the 5%		34.23	
		Median		33.00	
		Variance		467.350	
		Standard deviation		21.618	
		Minimum		5	
		Maximum		61	
		Range		56	
		Interquartile range		49	
		Skewness		.058	.283
		Kurtosis		-1.675	.559
	Not Dependent: Barthel higher than 60	Mean		45.44	2.771
		95% confidence interval for the mean	Lower limit	39.88	
			Upper limit	50.99	
		Mean trimmed at the 5%		46.88	
		Median		61.00	
		Variance		422.325	
		Standard deviation		20.551	
		Minimum		3	
		Maximum		61	
		Range		58	
		Interquartile range		36	
		Skewness		-.915	.322
		Kurtosis		-.771	.634

Section 3: Management of Censored Cases:

3.1.- Correlations observed between the main variables included in the study and the status at the end of the follow-up period of still living in the community or having died:

Group statisticians

	Orcasitas (community living) versus deceased 2025	N	Mean	Deviation typ.	Std. error of the mean
Sex	alive	35	1,17	,382	,065
	deceased	78	1,26	,439	,050
Age over or under 90 years	alive	35	1,23	,426	,072
	deceased	78	1,41	,495	,056
Orcasitas study income level according to TSI	alive	35	1,63	,490	,083
	deceased	78	1,46	,502	,057
Orcasitas study high or not high risk level	alive	35	1,46	,505	,085
	deceased	78	1,45	,501	,057
Orcasitas study high or not high intervention level	alive	35	1,74	,443	,075
	deceased	78	1,53	,503	,057
Orcasitas study to have or not to have education	alive	35	1,03	,169	,029
	deceased	77	1,17	,377	,043
Marital Status Widowed vs. non-widowed	alive	35	1,40	,497	,084
	deceased	78	1,29	,459	,052
Marital Status Married or Not Married	alive	35	1,66	,482	,081
	deceased	78	1,73	,446	,051
Chronicity Burden Cut-off Point 5 Diseases	alive	35	1,29	,458	,077
	deceased	78	1,28	,453	,051
Lives independently versus lives with children or other people	alive	35	1,49	,507	,086
	deceased	78	1,69	,465	,053
Lives independently versus lives with children	alive	30	1,40	,498	,091
	deceased	65	1,63	,486	,060
use or not of mobility aids	alive	35	1,23	,426	,072
	deceased	78	1,15	,363	,041
Having caregiver or not having caregiver	alive	34	1,68	,475	,081
	deceased	76	1,72	,450	,052

Independent sample testing

		Levene test for equality of variances	
		F	Sig.
Sex	Equal variances have been assumed	4,468	,037
Age over or under 90 years	Equal variances have been assumed	19,034	,000
Orcasitas study income level according to TSI	Equal variances have been assumed	3,710	,057
Orcasitas study high or not high risk level	Equal variances have been assumed	,025	,874
Orcasitas study high or not high intervention level	Equal variances have been assumed	22,406	,000
Orcasitas study to have or not to have education	Equal variances have been assumed	23,943	,000
Marital Status Widowed vs. non-widowed	Equal variances have been assumed	3,600	,060
Marital Status Married or Not Married	Equal variances have been assumed	2,162	,144
Chronicity Burden Cut-off Point 5 Diseases	Equal variances have been assumed	,006	,937
Lives independently versus lives with children or others	Equal variances have been assumed	5,886	,017
Lives independently versus lives with children	Equal variances have been assumed	,291	,591
use or not of mobility aids	Equal variances have been assumed	3,417	,067
Having caregiver or not having caregiver	Equal variances have been assumed	,913	,342

Independent sample testing

		T-test for equality of means						
							95% Confidence Interval for Difference	
		t	dl	Sig. (bilateral)	Mean difference	Standard error. of the difference	Lower	Upper
Sex	Equal variances have been assumed	-.988	111	.325	-.085	.086	-.255	.085
Age over or under 90 years	Equal variances have been assumed	-1.880	111	.063	-.182	.097	-.373	.010
Orcasitas study income level according to TSI	Equal variances have been assumed	1.648	111	.102	.167	.101	-.034	.368
Orcasitas study high or not high risk level	Equal variances have been assumed	.082	111	.934	.008	.102	-.194	.211
Orcasitas study high or not high intervention level	Equal variances have been assumed	2.200	111	.030	.217	.099	.022	.413
Orcasitas study to have or not to have education	Equal variances have been assumed	-2.103	110	.038	-.140	.067	-.272	-.008
Marital Status Widowed vs. non-widowed	Equal variances have been assumed	1.097	111	.275	.105	.096	-.085	.295
Marital Status Married or Not Married	Equal variances have been assumed	-.791	111	.431	-.074	.093	-.258	.111
Chronicity Burden Cut-off Point 5 Diseases	Equal variances have been assumed	.040	111	.968	.004	.092	-.180	.187
Lives independently versus lives with children or others	Equal variances have been assumed	-2.125	111	.036	-.207	.097	-.399	-.014
Lives independently versus lives with children	Equal variances have been assumed	-2.133	93	.036	-.231	.108	-.446	-.016
use or not of mobility aids	Equal variances have been assumed	.958	111	.340	.075	.078	-.080	.229
Having caregiver or not having caregiver	Equal variances have been assumed	-.500	108	.618	-.047	.094	-.234	.140

3.2.- Correlations observed between the main variables included in the study and the status at the end of the follow-up period of continuing to live in the community or living in a nursing home:

Group statisticians

	Orcasitas (community), versus Nursing home 2025	N	Mean	Deviation typ.	Std. error of the mean
Sex	Orcasitas (community)	35	1,17	,382	,065
	Nursing home	14	1,07	,267	,071
Age over or under 90 years	Orcasitas (community)	35	1,23	,426	,072
	Nursing home	14	1,14	,363	,097
Orcasitas study income level according to TSI	Orcasitas (community)	35	1,63	,490	,083
	Nursing home	14	1,64	,497	,133
Orcasitas study high or not high risk level	Orcasitas (community)	35	1,46	,505	,085
	Nursing home	14	1,71	,469	,125
Orcasitas study high or not high intervention level	Orcasitas (community)	35	1,74	,443	,075
	Nursing home	14	1,71	,469	,125
Orcasitas study to have or not to have education	Orcasitas (community)	35	1,03	,169	,029
	Nursing home	14	1,07	,267	,071
Marital Status Widowed vs. non-widowed	Orcasitas (community)	35	1,40	,497	,084
	Nursing home	14	1,57	,514	,137
Marital Status Married or Not Married	Orcasitas (community)	35	1,66	,482	,081
	Nursing home	14	1,57	,514	,137
Chronicity Burden Cut-off Point 5 Diseases	Orcasitas (community)	35	1,29	,458	,077
	Nursing home	14	1,14	,363	,097
Lives independently versus lives with children or other people	Orcasitas (community)	35	1,49	,507	,086
	Nursing home	14	1,64	,497	,133
Lives independently versus lives with children	Orcasitas (community)	30	1,40	,498	,091
	Nursing home	9	1,44	,527	,176
use or not of mobility aids	Orcasitas (community)	35	1,23	,426	,072
	Nursing home	14	1,36	,497	,133
Having caregiver or not having caregiver	Orcasitas (community)	34	1,68	,475	,081
	Nursing home	13	1,77	,439	,122

Independent sample testing

		Levene test for equality of variances	
		F	Sig.
Sex	Equal variances have been assumed	3,810	,057
Age over or under 90 years	Equal variances have been assumed	2,026	,161
Orcasitas study income level according to TSI	Equal variances have been assumed	,035	,852
Orcasitas study high or not high risk level	Equal variances have been assumed	6,207	,016
Orcasitas study high or not high intervention level	Equal variances have been assumed	,153	,698
Orcasitas study to have or not to have studies	Equal variances have been assumed	1,809	,185
Marital Status Widowed vs. non-widowed	Equal variances have been assumed	,111	,740
Marital Status Married or Not Married	Equal variances have been assumed	,850	,361
Chronicity Burden Cut-off Point 5 Diseases	Equal variances have been assumed	5,730	,021
Lives independently versus lives with children or other people	Equal variances have been assumed	2,847	,098
Lives independently versus lives with children	Equal variances have been assumed	,155	,696
use or not of mobility aids	Equal variances have been assumed	2,563	,116
Having caregiver or not having caregiver	Equal variances have been assumed	1,813	,185

Independent sample testing

		T-test for equality of means						
		t	dl	Sig. (bilateral)	Mean difference	Standard error. of the difference	95% Confidence Interval for Difference	
							Lower	Upper
Sex	Equal variances have been assumed	.893	47	.377	.100	.112	-.125	.325
Age over or under 90 years	Equal variances have been assumed	.662	47	.511	.086	.130	-.175	.346
Orcasitas study income level according to TSI	Equal variances have been assumed	-.092	47	.927	-.014	.156	-.327	.299
Orcasitas study high or not high risk level	Equal variances have been assumed	-1.641	47	.108	-.257	.157	-.572	.058
Orcasitas study high or not high intervention level	Equal variances have been assumed	.201	47	.842	.029	.142	-.258	.315
Orcasitas study to have or not to have education	Equal variances have been assumed	-.674	47	.504	-.043	.064	-.171	.085
Marital Status Widowed vs. non-widowed	Equal variances have been assumed	-1.081	47	.285	-.171	.159	-.491	.148
Marital Status Married or Not Married	Equal variances have been assumed	.552	47	.583	.086	.155	-.226	.398
Chronicity Burden Cut-off Point 5 Diseases	Equal variances have been assumed	1.041	47	.303	.143	.137	-.133	.419
Lives independently versus lives with children or others	Equal variances have been assumed	-.985	47	.330	-.157	.160	-.478	.164
Lives independently versus lives with children	Equal variances have been assumed	-.232	37	.818	-.044	.192	-.433	.344
use or not of mobility aids	Equal variances have been assumed	-.910	47	.368	-.129	.141	-.413	.156
Having caregiver or not having caregiver	Equal variances have been assumed	-.611	45	.544	-.093	.152	-.398	.213

3.3.- Correlations observed between the main variables included in the study and the status at the end of the follow-up period of residing in a nursing home or having died:

Group statisticians

	Deceased versus Nursing Home 2025	N	Mean	Deviation typ.	Std. error of the mean
Sex	deceased	78	1,26	,439	,050
	Nursing Home	14	1,07	,267	,071
Age over or under 90 years	deceased	78	1,41	,495	,056
	Nursing Home	14	1,14	,363	,097
Orcasitas study income level according to TSI	deceased	78	1,46	,502	,057
	Nursing Home	14	1,64	,497	,133
Orcasitas study high or not high risk level	deceased	78	1,45	,501	,057
	Nursing Home	14	1,71	,469	,125
Orcasitas study high or not high intervention level	deceased	78	1,53	,503	,057
	Nursing Home	14	1,71	,469	,125
Orcasitas study to have or not to have education	deceased	77	1,17	,377	,043
	Nursing Home	14	1,07	,267	,071
Marital Status Widowed vs. non-widowed	deceased	78	1,29	,459	,052
	Nursing Home	14	1,57	,514	,137
Marital Status Married or Not Married	deceased	78	1,73	,446	,051
	Nursing Home	14	1,57	,514	,137
Chronicity Burden Cut-off Point 5 Diseases	deceased	78	1,28	,453	,051
	Nursing Home	14	1,14	,363	,097
Live independently vs. live with children or others	deceased	78	1,69	,465	,053
	Nursing Home	14	1,64	,497	,133
Live independently vs. live with children	deceased	65	1,63	,486	,060
	Nursing Home	9	1,44	,527	,176
use or not of mobility aids	deceased	78	1,15	,363	,041
	Nursing Home	14	1,36	,497	,133
Having caregiver or not having caregiver	deceased	76	1,72	,450	,052
	Nursing Home	13	1,77	,439	,122

Independent sample testing

		Levene test for equality of variances	
		F	Sig.
Sex	Equal variances have been assumed	15,684	,000
Age over or under 90 years	Equal variances have been assumed	41,155	,000
Orcasitas study income level according to TSI	Equal variances have been assumed	4,060	,047
Orcasitas study high or not high risk level	Equal variances have been assumed	11,003	,001
Orcasitas study high or not high intervention level	Equal variances have been assumed	15,199	,000
Orcasitas study to have or not to have education	Equal variances have been assumed	4,259	,042
Marital Status Widowed vs. non-widowed	Equal variances have been assumed	2,087	,152
Marital Status Married or Not Married	Equal variances have been assumed	2,967	,088
Chronicity Burden Cut-off Point 5 Diseases	Equal variances have been assumed	7,065	,009
Lives independently versus lives with children or other people	Equal variances have been assumed	,432	,513
Lives independently versus lives with children	Equal variances have been assumed	,421	,519
use or not of mobility aids	Equal variances have been assumed	8,234	,005
Having caregiver or not having caregiver	Equal variances have been assumed	,525	,471

Independent sample testing

		T-test for equality of means					95% Confidence Interval for Difference	
		t	dl	Sig. (bilateral)	Mean difference	Standard error. of the difference	Lower	Upper
Sex	Equal variances have been assumed	1.521	90	.132	.185	.122	-.057	.427
Age over or under 90 years	Equal variances have been assumed	1.926	90	.057	.267	.139	-.008	.543
Orcasitas study income level according to TSI	Equal variances have been assumed	-1.247	90	.216	-.181	.145	-.470	.108
Orcasitas study high or not high risk level	Equal variances have been assumed	-1.844	90	.068	-.266	.144	-.552	.021
Orcasitas study high or not high intervention level	Equal variances have been assumed	-1.305	90	.195	-.189	.145	-.476	.098
Orcasitas study to have or not to have education	Equal variances have been assumed	.923	89	.358	.097	.105	-.112	.307
Marital Status Widowed vs. non-widowed	Equal variances have been assumed	-2.039	90	.044	-.277	.136	-.546	-.007
Marital Status Married or Not Married	Equal variances have been assumed	1.202	90	.233	.159	.133	-.104	.423
Chronicity Burden Cut-off Point 5 Diseases	Equal variances have been assumed	1.087	90	.280	.139	.128	-.115	.394
Lives independently versus lives with children or others	Equal variances have been assumed	.363	90	.717	.049	.136	-.221	.320
Lives independently versus lives with children	Equal variances have been assumed	1.067	72	.290	.186	.175	-.162	.534
use or not of mobility aids	Equal variances have been assumed	-1.817	90	.072	-.203	.112	-.426	.019
Having caregiver or not having caregiver	Equal variances have been assumed	-.338	87	.736	-.046	.135	-.313	.222

.

Section 4: Data associated with the Cox regression for the covariate "functional dependence or has no functional dependency according the local protocol criterion for functional dependence (Barthel >60)":

Case Process Overview

		N	Percentage
Cases available in the analysis	Event[a]	75	59.1%
	Censored	47	37.0%
	Total	122	96.1%
Excluded cases	Cases with missing values	5	3.9%
	Cases with negative time	0	.0%
	Cases censored before the event earlier in a stratum	0	.0%
	Total	5	3.9%
Total		127	100.0%

a. Dependent variable: survival in months up to 60 months

Status of stratum[a]

Stratum	Layer labels	Event	Censored	Percentage censored
1	Barthel severe dependence	31	6	16.2%
2	Barthel moderate dependence	44	41	48.2%
Total		75	47	38.5%

a. The strata variable is Orcasitas study Barthel level cut-off point 40 pre-lockdown

Encodings of categorical variables[b.c.d.e.f.g.h.i.j]

		Frequency	(1)
Polymedicated	1=Yes	111	1
	2=No	11	0
TSI Cut Income Level 11200[a]	1=less than 11.200 euros	60	1
	2=greater than 11.200 euros	62	0
Maintains dependence or not post-lockdown	1=Dependent: Barthel 60 or lower	71	1
	2=Not-dependent: Barthel over 60	51	0
High or not high risk level[a]	1=high	63	1
	2=medium or low	59	0
High or non-high intervention level[a]	1=high	49	1
	2=medium. low or unassigned	73	0
Education or not[a]	1=insufficient studies	107	1
	2=studies	15	0
Burden of chronic diseases[a]	1=less than 5 diseases	91	1
	2=5 or more diseases	31	0
Whether or not to have a caregiver	1=No caregiver	35	1
	2=Has some kind of caregiver	87	0
Use or not of devices helps mobility[a]	1=Use a mobility aid	98	1
	2=does not use any mobility aids	24	0

a. Coding Indicator Parameters

b. Category variable: Polypharmacy (Polymedicated Orcasitas Study 1: including protocol. 2: not included in protocol)

c. Category variable: income levelTSIcut11200 (Orcasitas study income level according to TSI)

d. Category variable: Barthel maintains dependence or not post-lockdown (Orcasitas study maintains or does not maintain dependence after lockdown)

e. Category variable: high or not high risk level (Orcasitas study high or not high risk level)

f. Category variable: high or not high intervention level (Orcasitas study high or not high intervention level)

g. Category variable: education or not (Orcasitas study to have or not to have education)

h. Category variable: disease burden (Orcasitas study chronicity load cut-off point 5 diseases)

i. Category variable: having or not having a caregiver (Having some type of caregiver or not having one)

Omnibus tests on model a coefficient

-2 Log of Likelihood	Global (score)			Change from the previous step		
	Chi-square	dl	Sig.	Chi-square	dl	Sig.
525.975	23.299	9	.006	.000	1	.999

a. Initial block number 9. Method = Enter

Omnibus tests on model a coefficient

Change from the previous block		
Chi-square	dl	Sig.
.000	1	.999

a. Initial block number 9. Method = Enter

Variables in the equation

	B	Std	Wald	dl	Sig.	Exp(B)	95.0% CI for Exp(B)	
							Lower	Upper
Maintains dependence or not post-lockdown	.625	.293	4.549	1	.033	1.867	1.052	3.315
Education or not	-.840	.328	6.537	1	.011	.432	.227	.822
Polymedicated	-.859	.414	4.293	1	.038	.424	.188	.955
Intervention level	.777	.312	6.208	1	.013	2.175	1.180	4.008
Income level	.421	.250	2.844	1	.092	1.524	.934	2.487
Risk level	.047	.327	.021	1	.884	1.049	.553	1.989
Burden of chronicity	-.009	.310	.001	1	.977	.991	.539	1.820
Mobility aid devices	.313	.342	.836	1	.361	1.367	.699	2.671
Caregiver or not caregiver	.000	.267	.000	1	.999	1.000	.593	1.688

Correlation matrix between regression coefficients

	Maintains dependence or not post-lockdown	Education level	Polymedicated	Intervention level
Education level	-.010			
Polymedicated	-.150	-.049		
Intervention level	.007	-.115	-.128	
Income level	.120	.124	-.100	-.039
Risk level	.195	.119	-.276	-.499
Chronicity burden	-.022	.200	-.001	.153
Mobility aid devices	-.154	-.098	.035	.172
Caregiver or not	.090	.003	-.127	-.065

Correlation matrix between regression coefficients

	Income Level	Risk level	Chronicity burden	Mobility aid devices
Education level				
Polymedicated				
Intervention level				
Income level				
Risk level	.174			
Chronicity burden	.230	.217		
Mobility aid devices	.094	-.035	.252	
Caregiver or not	.016	.139	-.078	-.113

Means of the covariates

	Mean
Maintains dependence or not post-lockdown	.582
Education level	.877
Polymedicated	.910
Intervention level	.402
Income level	.492
Risk level	.516
Chronicity burden	.746
Mobility aid devices	.803
Caregiver or not	.287

Section 5: Collinearity for the "functional dependence or has no functional dependency according the local protocol criterion for functional dependence (Barthel >60)" covariate: Variance Inflation Factor (VIF):

Descriptive statistics

	Media	Standard error	N
Survival in months up to 60 months	38.89	21.852	126
Maintains or does not remain dependent after lockdown	1.44	.498	126
Have or not to have education	1.12	.325	126
High or not high intervention level	1.60	.491	126

Correlations

		Survival in months up to 60 months	Maintain dependence or not after lockdown	To have or not to have an education	High or non-high level of intervention
Pearson correlation	Survival in months up to 60 months	1.000	.265	-.282	.262
	Maintain dependence or not after lockdown	.265	1.000	-.126	.027
	To have or not to have an education	-.282	-.126	1.000	-.052
	High or non-high level of intervention	.262	.027	-.052	1.000
Sig. (unilateral)	Survival in months up to 60 months	.	.001	.001	.002
	Maintain dependence or not after lockdown	.001	.	.080	.382
	To have or not to have an education	.001	.080	.	.280
	High or non-high level of intervention	.002	.382	.280	.
N	Survival in months up to 60 months	126	126	126	126
	Maintain dependence or not after lockdown	126	126	126	126
	To have or not to have an education	126	126	126	126
	High or non-high level of intervention	126	126	126	126

Variables entered/removed[b]

Model	Variables Entered	Variables removed	Method
1	Orcasitas study remains dependent or not after the lockdown[a]	.	Introduce
2	Orcasitas study to have or not to have a education	.	Introduce
3	Orcasitas study intervention level high or not high[a]	.	Introduce

a. All requested variables entered.

b. Dependent variable: survival in months up to 60 months

Model Overview

Model	R	R squared	Corrected R-squared	Standard error. of the estimate
1	.265a	.070	.063	21.157
2	.365b	.133	.119	20.513
3	.438c	.192	.172	19.884

a. Predictor variables: (Constant). Orcasitas study remains dependent or not after lockdown

b. Predictor variables: (Constant). Orcasitas Study remains dependent or not after lockdown. Orcasitas Study has or does not have education

c. Predictor variables: (Constant). Orcasitas study remains dependent or not after lockdown. Orcasitas study has or does not have education. Orcasitas study high or not high intervention level

Model Overview

Model	Change statistics				
	Change in R squared	F change	DL1	DL2	Sig. F change
1	.070	9.348	1	124	.003
2	.063	8.904	1	123	.003
3	.059	8.898	1	122	.003

ANOVA[d]

Model		Sum of squares	dl	Mean square	F	Sig.
1	Regression	4184.297	1	4184.297	9.348	.003[a]
	Residual	55502.147	124	447.598		
	Total	59686.444	125			
2	Regression	7930.767	2	3965.384	9.424	.000[b]
	Residual	51755.677	123	420.778		
	Total	59686.444	125			
3	Regression	11449.079	3	3816.360	9.652	.000[c]
	Residual	48237.366	122	395.388		
	Total	59686.444	125			

a. Predictor variables: (Constant). Orcasitas study remains dependent or not after lockdown

b. Predictor variables: (Constant). Orcasitas Study remains dependent or not after lockdown. Orcasitas Study has or does not have education

c. Predictor variables: (Constant). Orcasitas study remains dependent or not after lockdown. Orcasitas study has or does not have education. Orcasitas study high or not high intervention level

d. Dependent variable: survival in months up to 60 months

Coefficient

Model		Non-standardized coefficients		Standardized coefficients
		B	Standard error.	Beta
1	(Constant)	22.197	5.775	
	Orcasitas study maintains or does not remain dependent after lockdown	11.619	3.800	.265
2	(Constant)	43.195	8.993	
	Orcasitas study maintains or does not remain dependent after lockdown	10.224	3.714	.233
	Orcasitas study to have or not to have education	-16.973	5.688	-.253
3	(Constant)	25.258	10.590	
	Orcasitas study maintains or does not remain dependent after lockdown	10.003	3.601	.228
	Orcasitas study to have or not to have education	-16.158	5.521	-.240
	Orcasitas study high or not high intervention level	10.818	3.627	.243

a. Dependent variable: survival in months up to 60 months

Coefficient

Model		t	Sig.	95.0% confidence interval for B	
				Lower limit	Upper limit
1	(Constant)	3.843	.000	10.766	33.629
	Orcasitas study maintains or does not remain dependent after lockdown	3.058	.003	4.098	19.141
2	(Constant)	4.803	.000	25.394	60.997
	Orcasitas study maintains or does not remain dependent after lockdown	2.753	.007	2.872	17.576
	Orcasitas study to have or not to have education	-2.984	.003	-28.232	-5.714
3	(Constant)	2.385	.019	4.293	46.222
	Orcasitas study maintains or does not remain dependent after lockdown	2.778	.006	2.874	17.132
	Orcasitas study to have or not to have education	-2.927	.004	-27.087	-5.229
	Orcasitas study high or not high intervention level	2.983	.003	3.639	17.997

a. Dependent variable: survival in months up to 60 months

Coefficient

Model		Correlations		
		Zero Order	Partial	Semi-partial
1	(Constant)			
	Orcasitas study maintains or does not remain dependent after lockdown	.265	.265	.265
2	(Constant)			
	Orcasitas study maintains or does not remain dependent after lockdown	.265	.241	.231
	Orcasitas study to have or not to have education	-.282	-.260	-.251
3	(Constant)			
	Orcasitas study maintains or does not remain dependent after lockdown	.265	.244	.226
	Orcasitas study to have or not to have education	-.282	-.256	-.238
	Orcasitas study high or not high intervention level	.262	.261	.243

a. Dependent variable: survival in months up to 60 months

Coefficient

Model		Collinearity statistics	
		Tolerance	FIV
1	(Constant)		
	Orcasitas study maintains or does not remain dependent after lockdown	1.000	1.000
2	(Constant)		
	Orcasitas study maintains or does not remain dependent after lockdown	.984	1.016
	Orcasitas study to have or not to have education	.984	1.016
3	(Constant)		
	Orcasitas study maintains or does not remain dependent after lockdown	.984	1.017
	Orcasitas study to have or not to have education	.982	1.019
	Orcasitas study high or not high intervention level	.997	1.003

a. Dependent variable: survival in months up to 60 months

Excluded variables[c]

Model		Beta inside	t	Sig.	Partial correlation
1	Orcasitas study to have or not to have education	-.253a	-2.984	.003	-.260
	Orcasitas study high or not high intervention level	.255a	3.040	.003	.264
2	Orcasitas study high or not high intervention level	.243b	2.983	.003	.261

a. Predictor variables in the model: (Constant). Orcasitas Study is or is not dependent after lockdown

b. Predictor variables in the model: (Constant). Orcasitas Study is or is not dependent after lockdown. Orcasitas Study has or does not have education

c. Dependent variable: survival in months up to 60 months

Excluded variables[c]

Model		Collinearity statistics		
		Tolerance	FIV	Minimum tolerance
1	Orcasitas study to have or not to have education	.984	1.016	.984
	Orcasitas study high or not high intervention level	.999	1.001	.999
2	Orcasitas study high or not high intervention level	.997	1.003	.982

c. Dependent variable: survival in months up to 60 months

Correlations of the coefficients[a]

Model			Orcasitas study maintains or does not remain dependent after lockdown	Orcasitas study to have or not to have education	Orcasitas study high or not high intervention level
1	Correlations	Orcasitas study maintains or does not remain dependent after lockdown	1.000		
	Covariances	Orcasitas study maintains or does not remain dependent after lockdown	14.442		
2	Correlations	Orcasitas study maintains or does not remain dependent after lockdown	1.000	.126	
		Orcasitas study to have or not to have education	.126	1.000	
	Covariances	Orcasitas study maintains or does not remain dependent after lockdown	13.796	2.660	
		Orcasitas study to have or not to have education	2.660	32.355	
3	Correlations	Orcasitas study maintains or does not remain dependent after lockdown	1.000	.125	-.021
		Orcasitas study to have or not to have education	.125	1.000	.049
		Orcasitas study high or not high intervention level	-.021	.049	1.000
	Covariances	Orcasitas study maintains or does not remain dependent after lockdown	12.969	2.479	-.269
		Orcasitas study to have or not to have education	2.479	30.478	.991
		Orcasitas study high or not high intervention level	-.269	.991	13.152

a. Dependent variable: survival in months up to 60 months

Diagnoses of collinearity[to]

Model	Dimension	Self-values	Condition Index	Proportions of variance (Constant)	Proportions of variance: Orcasitas study maintains or does not remain dependent after lockdown
1	1	1.945	1.000	.03	.03
	2	.055	5.961	.97	.97
2	1	2.869	1.000	.00	.01
	2	.104	5.241	.00	.57
	3	.027	10.300	.99	.42
3	1	3.791	1.000	.00	.01
	2	.105	6.006	.00	.64
	3	.084	6.725	.00	.07
	4	.021	13.563	1.00	.28

a. Dependent variable: survival in months up to 60 months

Diagnoses of collinearity[a]

Model	Dimension	Proportions of variance: Orcasitas study to have or not to have education	Proportions of variance: Orcasitas study high or not high intervention level
1	1		
	2		
2	1	.01	
	2	.31	
	3	.68	
3	1	.00	.01
	2	.24	.02
	3	.26	.66
	4	.50	.31

a. Dependent variable: survival in months up to 60 months

Section 6: Schoenfeld method for the "functional dependence or has no functional dependency according the local protocol criterion for functional dependence (Barthel >60)" covariate after lockdown:

Sample Design Information

			N
Unweighted counts	Valid	Subjects	126
		Cases	126
	Invalid cases		1
	Total cases		127
Population Subject Size			190,000
Stage 1	Strata		1
	Units		126
Degrees of Freedom of Sampling Design			125

Descriptive statistics

	Media	Standard error	N
Survival in months up to 60 months	25.22	16.689	78
Schoenfeld's residue for [Barthel maintains dependence or not post-lockdown=1]	.0000	.65580	77
Schoenfeld's residue to have education or not	.0000	.51015	77
Schoenfeld residue for high or no intervention level	.0000	.71373	77

Correlations

		Survival in months up to 60 months	Schoenfeld's residue for [Barthel maintains dependence or not post-lockdown =1]	Schoenfeld's residue to have education or not	Schoenfeld residue for high or no intervention level
Survival in months up to 60 months	Pearson correlation	1	.103	-.093	.173
	Sig. (bilateral)		.373	.423	.133
	N	78	77	77	77
Schoenfeld's residue for [Barthel maintains dependence or not post-lockdown =1]	Pearson correlation	.103	1	.022	.127
	Sig. (bilateral)	.373		.848	.270
	N	77	77	77	77
Schoenfeld's residue to have education or not	Pearson correlation	-.093	.022	1	-.077
	Sig. (bilateral)	.423	.848		.507
	N	77	77	77	77
Schoenfeld residue for high or no intervention level	Pearson correlation	.173	.127	-.077	1
	Sig. (bilateral)	.133	.270	.507	
	N	77	77	77	77

Censorship and Event Information

Baseline Strata		Total	
		Unweighted	Weighted
Orcasitas Barthel level study cut-off point 40 pre-lockdown	Barthel severe or total dependence	37	37.000
	Barthel mild or moderate dependence	89	153.000
Total		126	190.000

Event stratum variable: Orcasitas study survival June 2025 at 60 months (5 years) = 2

Censorship and Event Information

Baseline Strata		Event		Censored
		Unweighted	Weighted	Unweighted
Orcasitas Barthel level study cut-off point 40 pre-lockdown	Barthel severe or total dependence	31	31.000	6
	Barthel mild or moderate dependence	46	76.000	43
Total		77	107.000	49

Event stratum variable: Orcasitas study survival June 2025 at 60 months (5 years) = 2

Censorship and Event Information

Baseline Strata		Censored	Percentage censored	
		Weighted	Unweighted	Weighted
Orcasitas Barthel level study cut-off point 40 pre-lockdown	Barthel severe or total dependence	6.000	16.2%	16.2%
	Barthel mild or moderate dependence	77.000	48.3%	50.3%
Total		83.000	38.9%	43.7%

Event stratum variable: Orcasitas study survival June 2025 at 60 months (5 years) = 2

Contrasts of model effects

Origin	DL1	DL2	F de Wald	Sig.
Barthel maintains dependence or not post-lockdown	1.000	125.000	2.557	.112
Education or not	1.000	125.000	5.649	.019
High or no intervention level	1.000	125.000	5.879	.017

Survival time variable: survival in months up to 60 months

Event stratum variable: Orcasitas study survival June 2025 at 60 months (5 years) = 2

Baseline strata variable: Orcasitas study Barthel level cut-off point 40 pre-lockdown

Model: Barthel maintains dependence or not post-lockdown. education or not. intervention level high or not

Section 7: Cox regression models incorporating interaction terms:

Case Process Overview

		N	Percentage
Cases available in the analysis	Event[a]	77	60.6%
	Censored	48	37.8%
	Total	125	98.4%
Excluded cases	Cases with missing values	2	1.6%
	Cases with negative time	0	.0%
	Cases censored before the event earlier in a stratum	0	.0%
	Total	2	1.6%
Total		127	100.0%

a. Dependent variable: survival in months up to 60 months

Encodings of categorical variables[b.c.d.e.f.g.h.i]

		Frequency	(1)	(2)
sex[a]	1=female	98	1	
	2=male	27	0	
Polymedicated	1=Yes	114	1	
	2=No	11	0	
Barthel post-lockdown	1=severe dependence	32	1	0
	2=moderate dependence	39	0	1
	3= cease to be dependent	54	0	0
Maintains dependency or not post-lockdown	1=Dependent: Barthel 60 or lower	71	1	
	2=Not-dependent: Barthel over 60	54	0	
Intervention level	1=high	50	1	
	2=medium. low or unassigned	75	0	
Education level	1=insufficient education	110	1	
	2=Education	15	0	
Chronicity burden	1=less than 5 diseases	92	1	
	2=5 or more diseases	33	0	
Age	1=under 90 years old	83	1	
	2=90 or more years old	42	0	

a. Coding Indicator Parameters

b. Category variable: sex

c. Category variable: Polymedicated (Polymedicated Orcasitas Study 1: including protocol. 2: not included in protocol)

d. Category variable: Barthel postlockdown

e. Category variable: Maintains dependence or not postlockdown

f. Category variable: Intervention level

g. Category variable: Education level

h. Category variable: Chronicity burden (cut-off point 5 diseases)

i. Category variable: age (age over or under 90 years)

Omnibus tests on model coefficients[a]

-2 Log of Likelihood	Global (score)			Change from the previous step		
	Chi-square	dl	Sig.	Chi-square	dl	Sig.
655.571	35.622	8	.000	1.189	1	.276

a. Initial block number 6. Method = Enter

Omnibus tests on model a coefficient

Change from the previous block		
Chi-square	dl	Sig.
1.189	1	.276

a. Initial block number 6. Method = Enter

Variables in the equation

	B	Sd	Wald	dl	Sig.	Exp(B)	95.0% CI for Exp(B)	
							Lower	Upper
Maintains dependence or not post-lockdown	1.152	.415	7.713	1	.005	3.163	1.403	7.130
Education level	-.748	.321	5.417	1	.020	.474	.252	.889
Polymedicated	-.579	.370	2.450	1	.117	.560	.271	1.157
Barthel post-lockdown*sex			4.123	2	.127			
Variable name Barthel post-lockdown(1)*sex	-.057	.386	.022	1	.882	.944	.443	2.014
Variable name Barthel post-lockdown(2)*sex	-.818	.429	3.644	1	.056	.441	.190	1.022
Barthel post-lockdown*age			.569	2	.752			
Variable name Barthel post-lockdown (1)*age older 90	-.269	.358	.566	1	.452	.764	.379	1.541
Variable name Barthel post-lockdown (2)*age older 90	-.083	.412	.041	1	.840	.920	.410	2.063
Chronicity burden*Intervention level	.306	.274	1.243	1	.265	1.358	.793	2.325

Correlation matrix between regression coefficients

	Maintains dependence or not post-lockdown	Education level	Polymedicated	Barthel post-lockdown (1)*sex
Education level	.069			
Polymedicated	-.020	-.144		
Barthel pos-l-ockdown (1)*sex	-.642	.078	.176	
Barthel post-lockdown(2)*sex	-.503	-.065	-.034	.408
Barthel post-lockdown (1)*age over 90	-.431	-.115	.048	.088
Barthel post-lockdown (2)*age older 90	-.368	.003	.010	.319
Chronicity burden*Intervention level	.014	.060	-.181	-.007

Correlation matrix between regression coefficients

	Barthel post-lockdown (2)*sex	Barthel post-lockdown (1)*age over 90	Barthel post-lockdown (2)*age older 90
Education level			
Polymedicated			
Barthel post-lockdown (1)*sex			
Barthel post-lockdown(2)*sex			
Barthel post-lockdown (1)*age over 90	.293		
Barthel post-lockdown (2)*age older 90	-.292	.194	
Chronicity burden*Intervention level	.112	.059	-.131

Means of the covariates

	Mean
Sex	.784
Barthel postlockdown(1)	.256
Barthel postlockdown(2)	.312
Intervention level	.400
Chronicity burden	.736
Age	.664
Maintains dependence or not postlockdown	.568
Education level	.880
Polymedicated	.912
Barthel postlockdown(1)*sex	.208
Barthel postlockdown(2)*sex	.240
Barthel postlockdown(1)*age	.136
Barthel postlockdown(2)*age	.208
Chronicity burden*Intervention level	.224

Section 8: Data associated with the Cox regression for the covariate "level of improvement in the Barthel Index score after lockdown":

Case Process Overview

		N	Percentage
Cases available in the analysis	Event[a]	43	33.9%
	Censored	32	25.2%
	Total	75	59.1%
Excluded cases	Cases with missing values	52	40.9%
	Cases with negative time	0	.0%
	Cases censored before the event earlier in a stratum	0	.0%
	Total	52	40.9%
Total		127	100.0%

a. Dependent variable: survival in months up to 60 months

Status of stratum[a]

Stratum	Layer labels	Event	Censored	Percentage censored
1	Barthel severe or total dependence	17	5	22.7%
2	Barthel mild or moderate dependence	26	27	50.9%
Total		43	32	42.7%

a. The strata variable is Orcasitas study Barthel level cut-off point 40 pre-lockdown

Encodings of categorical variables[b.c.d.e.f.g.h.i.j]

		Frequency	(1)
sexo[a]	1=woman	59	1
	2=male	16	0
Two levels improve Barthel score[a]	1=improvement between 5 and 20 points	37	1
	2=improvement between 25 and 95 points	38	0
Polymedicated	1=Yes	67	1
	2=No	8	0
High or no risk level	1=high	40	1
	2=medium or low	35	0
High or no intervention level	1=high	32	1
	2=medium. low or unassigned	43	0
Education or not[a]	1=insufficient education	65	1
	2=education	10	0
Burden of chronic diseases[a]	1=less than 5 diseases	55	1
	2=5 or more diseases	20	0
Caregiver or not	1=No caregiver	25	1
	2=Has caregiver	50	0
Use or not of devices helps mobility[a]	1=Use a mobility aid	60	1
	2=does not use any mobility aids	15	0

a. Coding Indicator Parameters

b. Category variable: sex

c. Category variable: two levels improvement score (two levels improvement score cut-off point 25)

d. Category variable: Polypharmacy (Polymedicated Protocol Orcasitas Study 1: included protocol. 2: not included in protocol)

e. Category variable: high or not high risk level (Orcasitas study high or not high risk level)

f. Category variable: high or no intervention level (Orcasitas study. high or not high intervention level)

g. Category variable: education or not (Orcasitas study to have or not to have education)

h. Category variable: burden of chronic diseases (Orcasitas Study chronicity burden cut-off point 5 diseases)

i. Category variable: non-family caregiver or non-caregiver (Have some type of caregiver or not have one)

j. Category variable: use or not of mobility aids (use or not of mobility aids)

Variables that are not in equation[a]

	Scoring	dl	Sig.
Two Levels Barthel Score Improvement (5-20 vs 25-95)	6.932	1	.008
Burden of chronic diseases	.029	1	.865
Use or not of mobility aid devices	2.592	1	.107
Education or not	3.171	1	.075
High or not high risk level	.666	1	.415
High or non-high intervention level	5.468	1	.019
Whether or not you have a Caregiver	.109	1	.741
Polymedicated	2.998	1	.083
Sex	4.365	1	.037

a. Residual chi-square = 28.227 with 9 gl Sig. = .001

Omnibus tests on the coefficients of model[b]

Step	-2 Log of Likelihood	Global (score)		
		Chi-square	dl	Sig.
1a	261.089	20.543	3	.000

a. Variables introduced in step number 1: sex

b. Initial block number 9. Method = Steps Forward (Likelihood Ratio)

Omnibus tests on the coefficients of model[b]

Step	Change from the previous step			Change from the previous block		
	Chi-square	dl	Sig.	Chi-square	dl	Sig.
1a	3.741	1	.053	3.741	1	.053

a. Variables introduced in step number 1: sex

b. Initial block number 9. Method = Steps Forward (Likelihood Ratio)

Variables in the equation

		B	SE	Wald	dl	Sig.
Step 1	Two Levels Improve Barthel Score	1.038	.337	9.483	1	.002
	High intervention level or not	.757	.323	5.476	1	.019
	Sex	-.749	.372	4.060	1	.044

Variables in the equation

		Exp(B)	95.0% CI for Exp(B) Lower	Upper
Step 1	Two Levels Improve Barthel Score	2.824	1.458	5.467
	High intervention level or not	2.131	1.131	4.016
	Sex	.473	.228	.980

Variables that are not in equation[a]

		Scoring	dl	Sig.
Step 1	Burden of chronic diseases	.834	1	.361
	Use or not of mobility aids	2.398	1	.121
	Education or not	1.740	1	.187
	High or not high risk level	.044	1	.833
	Caregiver or not caregiver	.328	1	.567
	Polymedicated	4.324	1	.038

a. Residual chi-square = 9.636 with 6 gl Sig. = .141

Model if the term is removed

Term removed	Chi-square of loss	dl	Sig.
_ Step 1 Sex	3.741	1	.053

Correlation matrix between regression coefficients

	Two Levels Improve Barthel Score	High or no intervention level
High or no intervention level	.090	
Sex	-.051	.214

Means of the covariates

	Mean
Two Levels Improve Barthel Score	.493
Burden of chronic diseases	.733
Use or not of mobility aids	.800
Education or not	.867
High or not high risk level	.533
High or non-high intervention level	.427
Whether or not to have a caregiver	.333
Polymedicated	.893
Sex	.787

Section 9: Collinearity for the covariate "level of improvement in the Barthel Index score after lockdown":

Descriptive statistics

	Mean	Standard deviation	N
Survival in months up to 60 months	40.61	21.959	79
Two Levels Improvement Score Cut-off Point 25	1.51	.503	79
Sex	1.22	.414	79
Orcasitas study high or not high intervention level	1.58	.496	79

Correlations

		Survival in months up to 60 months	Two Levels Improvement Score Cut-off Point 25
Pearson correlation	Survival in months up to 60 months	1.000	.185
	Two Levels Improvement Score Cut-off Point 25	.185	1.000
	Sex	-.233	.086
	Orcasitas study high or not high intervention level	.296	-.015
Sig. (unilateral)	Survival in months up to 60 months	.	.051
	Two Levels Improvement Score Cut-off Point 25	.051	.
	Sex	.019	.226
	Orcasitas study high or not high intervention level	.004	.448
N	Survival in months up to 60 months	79	79
	Two Levels Improvement Score Cut-off Point 25	79	79
	Sex	79	79
	Orcasitas study high or not high intervention level	79	79

Correlations

		Sex	Orcasitas study high or not high intervention level
Pearson correlation	Survival in months up to 60 months	-.233	.296
	Two Levels Improvement Score Cut-off Point 25	.086	-.015
	Sex	1.000	-.119
	Orcasitas study high or not high intervention level	-.119	1.000
Sig. (unilateral)	Survival in months up to 60 months	.019	.004
	Two Levels Improvement Score Cut-off Point 25	.226	.448
	Sex	.	.149
	Orcasitas study high or not high intervention level	.149	.
N	Survival in months up to 60 months	79	79
	Two Levels Improvement Score Cut-off Point 25	79	79
	Sex	79	79
	Orcasitas study high or not high intervention level	79	79

Variables entered/removed[b]

Model	Variables Entered	Variables removed	Method
1	Two Levels Improvement Score Cut-off Point 25A	.	Introduce
2	Sex	.	Introduce
3	Orcasitas study intervention level high or not high[to]	.	Introduce

a. All requested variables entered.

b. Dependent variable: survival in months up to 60 months

Model Overview

Model	R	R squared	Corrected R-squared	Standard error. of the estimate
1	.185a	.034	.022	21.719
2	.311b	.097	.073	21.141
3	.413c	.171	.138	20.393

a. Predictor variables: (Constant). two levels improve score cut-off point 25
b. Predictor variables: (Constant). two levels improvement score cut-off point 25. sex
c. Predictor variables: (Constant). two levels improve cut-off point score 25. sex. Orcasitas Study intervention level high or not high

Model Overview

Model	Change statistics				
	Change in R squared	F change	DL1	DL2	Sig. F change
1	.034	2.738	1	77	.102
2	.063	5.268	1	76	.024
3	.074	6.677	1	75	.012

ANOVA[d]

Model		Sum of squares	dl	Mean square	F	Sig.
1	Regression	1291.492	1	1291.492	2.738	.102[a]
	Residual	36321.344	77	471.706		
	Total	37612.835	78			
2	Regression	3646.136	2	1823.068	4.079	.021[b]
	Residual	33966.700	76	446.930		
	Total	37612.835	78			
3	Regression	6423.009	3	2141.003	5.148	.003[c]
	Residual	31189.827	75	415.864		
	Total	37612.835	78			

a. Predictor variables: (Constant). two levels improve score cut-off point 25
b. Predictor variables: (Constant). two levels improvement score cut-off point 25. sex
c. Predictor variables: (Constant). two levels improve cut-off point score 25. sex. Orcasitas Study intervention level high or not high
d. Dependent variable: survival in months up to 60 months

Coefficient

Model		Non-standardized coefficients		Standardized coefficients
		B	Standard error.	Beta
1	(Constant)	28.426	7.757	
	Two Levels Improvement Score Cut-off Point 25	8.087	4.888	.185
2	(Constant)	43.213	9.925	
	Two Levels Improvement Score Cut-off Point 25	9.027	4.775	.207
	Sex	-13.334	5.809	-.251
3	(Constant)	21.883	12.641	
	Two Levels Improvement Score Cut-off Point 25	9.085	4.606	.208
	Sex	-11.617	5.643	-.219
	Orcasitas study high or not high intervention level	12.107	4.685	.274

a. Dependent variable: survival in months up to 60 months

Coefficient

Model		t	Sig.	95.0% confidence interval for B	
				Lower limit	Upper limit
1	(Constant)	3.664	.000	12.979	43.872
	Two Levels Improvement Score Cut-off Point 25	1.655	.102	-1.645	17.819
2	(Constant)	4.354	.000	23.444	62.981
	Two Levels Improvement Score Cut-off Point 25	1.891	.062	-.483	18.538
	Sex	-2.295	.024	-24.904	-1.764
3	(Constant)	1.731	.088	-3.300	47.066
	Two Levels Improvement Score Cut-off Point 25	1.972	.052	-.091	18.261
	Sex	-2.059	.043	-22.858	-.376
	Orcasitas study high or not high intervention level	2.584	.012	2.773	21.441

a. Dependent variable: survival in months up to 60 months

Coefficient

Model		Correlations		
		Zero Order	Partial	Semi-partial
1	(Constant)			
	Two Levels Improvement Score Cut-off Point 25	.185	.185	.185
2	(Constant)			
	Two Levels Improvement Score Cut-off Point 25	.185	.212	.206
	Sex	-.233	-.255	-.250
3	(Constant)			
	Two Levels Improvement Score Cut-off Point 25	.185	.222	.207
	Sex	-.233	-.231	-.216
	Orcasitas study high or not high intervention level	.296	.286	.272

a. Dependent variable: survival in months up to 60 months

Coefficient

Model		Collinearity statistics	
		Tolerance	FIV
1	(Constant)		
	Two Levels Improvement Score Cut-off Point 25	1.000	1.000
2	(Constant)		
	Two Levels Improvement Score Cut-off Point 25	.993	1.007
	Sex	.993	1.007
3	(Constant)		
	Two Levels Improvement Score Cut-off Point 25	.993	1.007
	Sex	.979	1.022
	Orcasitas study high or not high intervention level	.986	1.014

a. Dependent variable: survival in months up to 60 months

Excluded variables[c]

Model		Beta inside	t	Sig.	Partial correlation
1	Sex	-.251a	-2.295	.024	-.255
	Orcasitas study high or not high intervention level	.299a	2.788	.007	.305
2	Orcasitas study high or not high intervention level	.274b	2.584	.012	.286

a. Predictor variables in the model: (Constant). two levels improve score cut-off point 25

b. Predictor variables in the model: (Constant). two levels improve score cut-off point 25. sex

c. Dependent variable: survival in months up to 60 months

Excluded variables[c]

Model		Collinearity statistics		
		Tolerance	FIV	Minimum tolerance
1	Sex	.993	1.007	.993
	Orcasitas study high or not high intervention level	1.000	1.000	1.000
2	Orcasitas study high or not high intervention level	.986	1.014	.979

c. Dependent variable: survival in months up to 60 months

Correlations of the coefficients[a]

Model			Two Levels Improvement Score Cut-off Point 25	Sex	Orcasitas study high or not high intervention level
1	Correlations	Two Levels Improvement Score Cut-off Point 25	1.000		
	Covariances	Two Levels Improvement Score Cut-off Point 25	23.888		
2	Correlations	Two Levels Improvement Score Cut-off Point 25	1.000	-.086	
		Sex	-.086	1.000	
	Covariances	Two Levels Improvement Score Cut-off Point 25	22.801	-2.380	
		Sex	-2.380	33.747	
3	Correlations	Two Levels Improvement Score Cut-off Point 25	1.000	-.085	.005
		Sex	-.085	1.000	.118
		Orcasitas study high or not high intervention level	.005	.118	1.000
	Covariances	Two Levels Improvement Score Cut-off Point 25	21.216	-2.199	.104
		Sex	-2.199	31.843	3.113
		Orcasitas study high or not high intervention level	.104	3.113	21.952

a. Dependent variable: survival in months up to 60 months

Diagnoses of collinearity[a]

Model	Dimension	Self-values	Condition Index	Proportions of variance: (Constant)	Proportions of variance: Two Levels Improvement Score Cut-off Point 25
1	1	1.949	1.000	.03	.03
	2	.051	6.187	.97	.97
2	1	2.870	1.000	.01	.01
	2	.092	5.576	.00	.52
	3	.038	8.671	.99	.47
3	1	3.779	1.000	.00	.01
	2	.107	5.930	.00	.01
	3	.089	6.499	.00	.75
	4	.024	12.473	1.00	.23

a. Dependent variable: survival in months up to 60 months

Diagnoses of collinearity[to]

Model	Dimension	Proportions of variance: Sex	Proportions of variance: Orcasitas study high or not high intervention level
1	1		
	2		
2	1	.01	
	2	.56	
	3	.42	
3	1	.01	.01
	2	.43	.43
	3	.25	.09
	4	.32	.47

a. Dependent variable: survival in months up to 60 months

Section 10: Schoenfeld method for the covariate "level of improvement in the Barthel Index score after lockdown":

Descriptive statistics

	Mean	Standard error	N
Survival in months up to 60 months	25.22	16.689	78
Schoenfeld's residual for [two levels of Barthel score improvement (5-20; 25-95) = 1]	.0000	.77033	45
Schoenfeld's residue for sex	.0000	.70356	45
Schoenfeld residue for high or no intervention level	.0000	.77585	45

Correlations

		Survival in months up to 60 months	Schoenfeld's Residue for [two levels improves score Barthel=1]	Schoenfeld's residue for sex	Schoenfeld's residue for interventional level
Survival in months up to 60 months	Pearson correlation	1	.009	.140	.182
	Sig. (bilateral)		.955	.359	.233
	N	78	45	45	45
Schoenfeld's Residue for [two levels improves score Barthel=1]	Pearson correlation	.009	1	-.143	-.107
	Sig. (bilateral)	.955		.347	.484
	N	45	45	45	45
Schoenfeld's residue for sex	Pearson correlation	.140	-.143	1	-.013
	Sig. (bilateral)	.359	.347		.933
	N	45	45	45	45
Schoenfeld residue for high or no intervention level	Pearson correlation	.182	-.107	-.013	1
	Sig. (bilateral)	.233	.484	.933	
	N	45	45	45	45

Sample Design Information

			N
Unweighted counts	Valid	Subjects	79
		Cases	79
	Invalid cases		48
	Total cases		127
Population Subject Size			131.000
Stage 1	Strata		1
	Units		79
Degrees of Freedom of Sampling Design			78

Censorship and Event Information

Baseline Strata		Total	
		Unweighted	Weighted
Orcasitas Barthel level study cut-off point 40 pre-lockdown	Barthel severe or total dependence	22	22.000
	Barthel mild or moderate dependence	57	109.000
Total		79	131.000

Event stratum variable: Orcasitas study survival June 2025 at 60 months (5 years) = 2

Censorship and Event Information

Baseline Strata		Event		Censored
		Unweighted	Weighted	Unweighted
Orcasitas Barthel level study cut-off point 40 pre-lockdown	Barthel severe or total dependence	17	17.000	5
	Barthel mild or moderate dependence	28	53.000	29
Total		45	70.000	34

Event stratum variable: Orcasitas study survival June 2025 at 60 months (5 years) = 2

Censorship and Event Information

Baseline Strata		Censored	Percentage censored	
		Weighted	Unweighted	Weighted
Orcasitas Barthel level study cut-off point 40 pre-lockdown	Barthel severe or total dependence	5.000	22.7%	22.7%
	Barthel mild or moderate dependence	56.000	50.9%	51.4%
Total		61.000	43.0%	46.6%

Event stratum variable: Orcasitas study survival June 2025 at 60 months (5 years) = 2

Contrasts of model effects

Origin	DL1	DL2	F de Wald	Sig.
Two Levels Improve Barthel Score	1.000	78.000	7.780	.007
Sex	1.000	78.000	5.512	.021
High or not high intervention level	1.000	78.000	4.971	.029

Survival time variable: survival in months up to 60 months

Event stratum variable: Orcasitas study survival June 2025 at 60 months (5 years) = 2

Baseline strata variable: Orcasitas study Barthel level cut-off point 40 pre-lockdown

Model: two levels improve Barthel score. sex. high intervention level or no

Estimates of parameters[b]

Parameter		95% confidence interval	
	B	Lower	Upper
[two levels improve score Barthel=1]	.939	.269	1.608
[two levels improve score Barthel=2]	.000[a]	.	.
Sex	.790	.120	1.461
High or not high intervention level	-.697	-1.319	-.075

Survival time variable: survival in months up to 60 months

Event stratum variable: Orcasitas study survival June 2025 at 60 months (5 years) = 2

Baseline strata variable: Orcasitas study Barthel level cut-off point 40 pre-lockdown

Model: two levels improve Barthel score. sex. high intervention level or no

a. Set to zero because this parameter is redundant.

b. Tie-breaking method: Efron

Estimates of b-parameters

Parameter	Hypothesis testing		
	t	dl	Sig.
[two levels improve score Barthel=1]	2.789	78.000	.007
[two levels improve score Barthel=2]	.	.	.
Sex	2.348	78.000	.021
High or not high intervention level	-2.230	78.000	.029

Survival time variable: survival in months up to 60 months

Event stratum variable: Orcasitas study survival June 2025 at 60 months (5 years) = 2

Baseline strata variable: Orcasitas study Barthel level cut-off point 40 pre-lockdown

Model: two levels improve Barthel score. sex. high intervention level or no

b. Tie-breaking method: Efron

Pattern Values

		Survival Time Interval		Sex	High or not high intervention level
		Home	End		
Reference Standard	1	.000	[a]	1.21	1.60

An unspecified predictor has been assigned the value of this predictor in the reference pattern.

Each survival time interval is defined as Start < Survival Time <= End.

Model: two levels improves Barthel score. sex. intervention level high or not.

a. No limits

Pattern Values

		Two Levels Improvement Score Cut-off Point 25
Reference Standard	1	improvement between 25 and 95 points

An unspecified predictor has been assigned the value of this predictor in the reference pattern.

Each survival time interval is defined as Start < Survival Time <= End.

Model: two levels improves Barthel score. sex. intervention level high or not.

Section 11: "Incremental Prognostic Value of Functional Change."

- Subsection A: Generalized linear regression models including all clinical and social covariates analyzed in this study:

A.1. Model 1: Analysis for the clinical-social base model.

Model Information

Dependent variable	Orcasitas study: June 2025 survival at 60 months (5 years)[a]
Probability distribution	Binomial
Link Function	Logit

a. The procedure models deceased as the answer and treats vivo as the reference category.

Case Processing Summary

	N	Percentage
Includes	122	96.1%
Excluded	5	3.9%
Total	127	100.0%

Categorical variable information

			N	Percentage
Dependent variable	Orcasitas study: June 2025 survival at 60 months (5 years)	alive	47	38.5%
		deceased	75	61.5%
		Total	122	100.0%

Continuous variable information

		N	Minimum	Maximum	Mean	Standard deviation
Covariable	High or not high risk level	122	1	2	1.48	.502
	High or not high intervention level	122	1	2	1.60	.492
	Chronicity Burden	122	1	2	1.25	.437
	Polymedicated	122	1	2	1.09	.288
	Age	122	66	102	86.62	6.413
	Sex	122	1	2	1.21	.411
	Have or not to have education	122	1	2	1.12	.330
	Income level	122	1	2	1.51	.502
	Having caregiver or not	122	1	2	1.71	.454
	Use or not of mobility aids	122	1	2	1.20	.399
	Marital status married or not married	122	1	2	1.69	.465
	Marital status widowed or non-widowed	122	1	2	1.36	.482
	Lives independently versus lives with children or other people	122	1	2	1.63	.484

Goodness of fit[b]

	Value	dl	Value/dl
Deviation	118.836	106	1.121
Escalated Deviance	118.836	106	
Pearson's Chi-square	104.788	106	.989
Pearson's chi-square scaled	104.788	106	
Log likelihood[a]	-60.804		
Akaike Information Criterion (AIC)	149.609		
Finite Sample Corrected AIC (AICC)	153.534		
Bayesian Information Criterion (BIC)	188.865		
Consistent AIC (CAIC)	202.865		

Dependent variable: Orcasitas study survival June 2025 at 60 months (5 years)

Model: (Intersection). high-not-high risk level. high-not-high interventional level. chronicity burden. polymedicated. age. sex. education. income-level. caregiver. use of mobility aid devices. marital status married. marital status widowed. lives independently vs lives with children or other people

a. The full log-likelihood function is displayed and used to calculate the reporting criteria.

b. The information criteria are in "the smaller the better" form.

Omnibus Contrast[a]

Chi-square of the likelihood ratio	dl	Sig.
38.263	13	.000

Dependent variable: Orcasitas study survival June 2025 at 60 months (5 years)

Model: (Intersection). high-not-high risk level. high-not-high risk interventional level. chronicity burden. polymedicated. age. sex. education. income-level. caregiver. use of mobility aid devices. marital status married. marital status widowed. lives independently vs lives with children or other people

a. Compare the fitted model to the model with only the intersection.

Contrasts of model effects

Origin	Type III		
	Wald's chi-square	dl	Sig.
(Intersection)	1.810	1	.178
Risk level high or not high	.031	1	.861
Intervention level high or not high	6.212	1	.013
Chronicity burden	1.112	1	.292
Polymedicated	4.739	1	.029
Age	5.943	1	.015
Sex	1.748	1	.186
Education	4.698	1	.030
Income level	1.225	1	.268
Caregiver	.279	1	.597
Use or not of mobility aid devices	1.788	1	.181
Marital status married	1.566	1	.211
Marital status widowed	2.041	1	.153
Lives independently vs lives with children or other people	1.007	1	.316

Dependent variable: Orcasitas study survival June 2025 at 60 months (5 years)

Model: (Intersection). high-not-high risk level. high-not-high intervention level. chronicity burden. polymedicated. age. sex. education. income level. caregiver. use of mobility aid devices. marital status married. marital status widowed. lives independently vs lives with children or other people

Parameter estimates

Parameter	B	Std. Error	Wald confidence interval 95% Lower	Upper	Hypothesis testing Wald's chi-square	dl	Sig.
(Intersection)	-7.943	5.9035	-19.513	3.628	1.810	1	.178
Risk level high or not high	.093	.5326	-.951	1.137	.031	1	.861
Intervention level high or not high	-1.288	.5166	-2.300	-.275	6.212	1	.013
Chronicity burden	-.604	.5731	-1.727	.519	1.112	1	.292
Polymedicated	3.134	1.4399	.312	5.957	4.739	1	.029
Age	.106	.0434	.021	.191	5.943	1	.015
Sex	.824	.6231	-.397	2.045	1.748	1	.186
Education	2.170	1.0010	.208	4.132	4.698	1	.030
Income level	-.532	.4809	-1.475	.410	1.225	1	.268
Caregiver	.286	.5420	-.776	1.349	.279	1	.597
Use or not of mobility aid devices	-.789	.5903	-1.946	.368	1.788	1	.181
Marital status married	-1.345	1.0750	-3.452	.762	1.566	1	.211
Marital status widowed	-1.419	.9937	-3.367	.528	2.041	1	.153
Lives independently vs lives with children or other people	.506	.5044	-.482	1.495	1.007	1	.316
(Scale)	1a						

Dependent variable: Orcasitas study survival June 2025 at 60 months (5 years)

Model: (Intersection). high-not-high risk level. high-not-high intervention level. chronicity burden. polymedicated. age. sex. education. income level. caregiver. use of mobility aid devices. marital status married. marital status widowed. lives independently vs lives with children or other people

a. Fixed at the value shown.

A.2. Model 2: Analysis for the Line of Base model.

Model Information

Dependent variable	Orcasitas study: June 2025 survival at 60 months (5 years)[a]
Probability distribution	Binomial
Link Function	Logit

a. The procedure models deceased as the answer and treats vivo as the reference category.

Case Processing Summary

	N	Percentage
Includes	122	96.1%
Excluded	5	3.9%
Total	127	100.0%

Categorical variable information

			N	Percentage
Dependent variable	Orcasitas study: June 2025 survival at 60 months (5 years)	alive	47	38.5%
		deceased	75	61.5%
		Total	122	100.0%

Continuous variable information

		N	Minimum	Maximum	Mean	Standard deviation
Covariable	High or not high risk level	122	1	2	1.48	.502
	High or not high intervention level	122	1	2	1.60	.492
	Chronicity Burden	122	1	2	1.25	.437
	Polymedicated	122	1	2	1.09	.288
	Age	122	66	102	86.62	6.413
	Sex	122	1	2	1.21	.411
	Have or not to have education	122	1	2	1.12	.330
	Income level	122	1	2	1.51	.502
	Having caregiver or not	122	1	2	1.71	.454
	Use or not of mobility aids	122	1	2	1.20	.399
	Marital status married or not married	122	1	2	1.69	.465
	Marital status widowed or not widowed	122	1	2	1.36	.482
	Lives independently vs lives with children or other people	122	1	2	1.63	.484
	Barthel score pre-lockdown	122	0	60	42.84	19.123

Goodness of fit[b]

	Value	dl	Value/dl
Deviation	108.878	106	1.027
Escalated Deviance	108.878	106	
Pearson's Chi-square	103.400	106	.975
Pearson's chi-square scaled	103.400	106	
Log likelihood[a]	-55.132		
Akaike Information Criterion (AIC)	140.264		
Finite Sample Corrected AIC (AICC)	144.792		
Bayesian Information Criterion (BIC)	182.324		
Consistent AIC (CAIC)	197.324		

Dependent variable: Orcasitas study survival June 2025 at 60 months (5 years)

Model: (Intersection). high-not-high risk level. high-not-high intervention level. chronicity burden. Polymedicated. Age. sex. education. income level. caregiver. use of mobility aid devices. marital status married. marital status widowed. lives independently vs lives with children or other people. Barthel score pre-lockdown

a. The full log-likelihood function is displayed and used to calculate the reporting criteria.

b. The information criteria are in "the smaller the better" form.

Omnibus Contrast[a]

Chi-square of the likelihood ratio	dl	Sig.
50.994	14	.000

Dependent variable: Orcasitas study survival June 2025 at 60 months (5 years)

Model: (Intersection). high-not-high risk level. high-not-high intervention level. chronicity burden. Polymedicated. Age. sex. education. income level. caregiver. use of mobility aid devices. marital status married. marital status widowed. lives independently vs lives with children or other people. Barthel score pre-lockdown

a. Compare the fitted model to the model with only the intersection.

Contrasts of model effects

Origin	Type III		
	Wald's chi-square	dl	Sig.
(Intersection)	1.581	1	.209
Risk level high or not high	.465	1	.495
Intervention level high or not high	5.506	1	.019
Chronicity burden	.553	1	.457
Polymedicated	3.569	1	.059
Age	7.351	1	.007
Sex	2.268	1	.132
Education	4.968	1	.026
Income level	.155	1	.693
Caregiver	.046	1	.829
Use or not of mobility aid devices	2.493	1	.114
Marital status married	1.369	1	.242
Marital status widowed	2.266	1	.132
Lives independently vs lives with children or other people	.157	1	.692
Barthel score pre-lockdown	9.662	1	.002

Dependent variable: Orcasitas study survival June 2025 at 60 months (5 years)

Model: (Intersection). high-not-high risk level. high-not-high intervention level. chronicity burden. Polymedicated. Age. sex. education. income level. caregiver. use of mobility aid devices. marital status married. marital status widowed. lives independently vs lives with children or other people. Barthel score pre-lockdown

Parameter estimates

Parameter	B	Std. Error	Wald confidence interval 95%		Hypothesis testing		
			Lower	Upper	Wald's chi-square	dl	Sig.
(Intersection)	-8.357	6.6470	-21.385	4.670	1.581	1	.209
Risk level high or not high	.395	.5796	-.741	1.531	.465	1	.495
Intervention level high or not high	-1.288	.5488	-2.363	-.212	5.506	1	.019
Chronicity burden	-.460	.6179	-1.671	.751	.553	1	.457
Polymedicated	3.466	1.8344	-.130	7.061	3.569	1	.059
Age	.139	.0513	.039	.240	7.351	1	.007
Sex	1.010	.6704	-.304	2.323	2.268	1	.132
Education	2.251	1.0097	.272	4.230	4.968	1	.026
Income level	-.207	.5260	-1.238	.824	.155	1	.693
Caregiver	-.124	.5741	-1.249	1.002	.046	1	.829
Use or not of mobility aid devices	-1.029	.6516	-2.306	.248	2.493	1	.114
Marital status married	-1.320	1.1284	-3.532	.891	1.369	1	.242
Marital status widowed	-1.576	1.0471	-3.629	.476	2.266	1	.132
Lives independently vs lives with children or other people	.213	.5371	-.840	1.265	.157	1	.692
Barthel score pre-lockdown	-.056	.0179	-.091	-.021	9.662	1	.002
(Scale)	1a						

Dependent variable: Orcasitas study survival June 2025 at 60 months (5 years)
Model: (Intersection). high-not-high risk level. high-not-high intervention level. chronicity burden. Polymedicated. Age. sex. education. income level. caregiver. use of mobility aid devices. marital status married. marital status widowed. lives independently vs lives with children or other people. Barthel score pre-lockdown
a. Fixed at the value shown.

A.3. Model 3: Analysis for the Incremental Model June–July 2020.

Model Information

Dependent variable	Orcasitas study: June 2025 survival at 60 months (5 years)[a]
Probability distribution	Binomial
Link Function	Logit

a. The procedure models deceased as the answer and treats vivo as the reference category.

Case Processing Summary

	N	Percentage
Includes	122	96.1%
Excluded	5	3.9%
Total	127	100.0%

Categorical variable information

			N	Percentage
Dependent variable	Orcasitas study: June 2025 survival at 60 months (5 years)	alive	47	38.5%
		deceased	75	61.5%
		Total	122	100.0%

Continuous variable information

		N	Minimum	Maximum	Mean	Standard deviation
Covariable	High or not high risk level	122	1	2	1.48	.502
	High or not high intervention level	122	1	2	1.60	.492
	Chronicity Burden	122	1	2	1.25	.437
	Polymedicated	122	1	2	1.09	.288
	Age	122	66	102	86.62	6.413
	Sex	122	1	2	1.21	.411
	Education	122	1	2	1.12	.330
	Income level	122	1	2	1.51	.502
	Having caregiver or not	122	1	2	1.71	.454
	Use or not of mobility aids	122	1	2	1.20	.399
	Marital status married or not married	122	1	2	1.69	.465
	Marital status widowed or not widowed	122	1	2	1.36	.482
	Lives independently vs lives with children or other people	122	1	2	1.63	.484
	Barthel score post-lockdown	122	0	95	53.69	25.824

Goodness of fit[b]

	Value	dl	Value/dl
Deviation	102.297	107	.956
Escalated Deviance	102.297	107	
Pearson's Chi-square	131.226	107	1.226
Pearson's chi-square scaled	131.226	107	
Log likelihood[a]	-51.149		
Akaike Information Criterion (AIC)	132.297		
Finite Sample Corrected AIC (AICC)	136.826		
Bayesian Information Criterion (BIC)	174.358		
Consistent AIC (CAIC)	189.358		

Dependent variable: Orcasitas study survival June 2025 at 60 months (5 years)

Model: (Intersection). high-not-high risk level. high-not-high intervention level. chronicity burden. polymedicated. age. sex. education. income level. caregiver. use of mobility aid devices. marital status married. marital status widowed. lives independently vs lives with children or other people. Barthel score post-lockdown

a. The full log-likelihood function is displayed and used to calculate the reporting criteria.

b. The information criteria are in "the smaller the better" form.

Omnibus Contrast[a]

Chi-square of the likelihood ratio	dl	Sig.
60.347	14	.000

Dependent variable: Orcasitas study survival June 2025 at 60 months (5 years)

Model: (Intersection). high-not-high risk level. high-not-high intervention level. chronicity burden. polymedicated. age. sex. education. income level. caregiver. use of mobility aid devices. marital status married. marital status widowed. lives independently vs lives with children or other people. Barthel score post-lockdown

a. Compare the fitted model to the model with only the intersection.

Contrasts of model effects

Origin	Type III		
	Wald's chi-square	dl	Sig.
(Intersection)	.007	1	.934
Risk level high or not high	.186	1	.666
Intervention level high or not high	6.327	1	.012
Chronicity burden	1.516	1	.218
Polymedicated	3.421	1	.064
Age	4.335	1	.037
Sex	4.143	1	.042
Education	3.025	1	.082
Income level	1.057	1	.304
Caregiver	.126	1	.723
Use or not of mobility aid devices	2.560	1	.110
Marital status married	2.086	1	.149
Marital status widowed	5.306	1	.021
Lives independently vs lives with children or other people	.445	1	.505
Barthel score post-lockdown	15.256	1	.000

Dependent variable: Orcasitas study survival June 2025 at 60 months (5 years)

Model: (Intersection). high-not-high risk level. high-not-high intervention level. chronicity burden. polymedicated. age. sex. education. income level. caregiver. use of mobility aid devices. marital status married. marital status widowed. lives independently vs lives with children or other people. Barthel score post-lockdown

Parameter estimates

Parameter	B	Std. Error	Wald confidence interval 95%		Hypothesis testing		
			Lower	Upper	Wald's chi-square	dl	Sig.
(Intersection)	.585	7.0343	-13.202	14.372	.007	1	.934
Risk level high or not high	.272	.6298	-.963	1.506	.186	1	.666
Intervention level high or not high	-1.494	.5940	-2.658	-.330	6.327	1	.012
Chronicity burden	-.850	.6907	-2.204	.503	1.516	1	.218
Polymedicated	3.278	1.7722	-.196	6.751	3.421	1	.064
Age	.102	.0490	.006	.198	4.335	1	.037
Sex	1.559	.7661	.058	3.061	4.143	1	.042
Education	1.882	1.0817	-.239	4.002	3.025	1	.082
Income level	-.590	.5742	-1.716	.535	1.057	1	.304
Caregiver	-.219	.6175	-1.430	.991	.126	1	.723
Use of mobility aid devices	-1.104	.6902	-2.457	.248	2.560	1	.110
Marital status married	-1.734	1.2003	-4.086	.619	2.086	1	.149
Marital status widowed	-2.700	1.1720	-4.997	-.403	5.306	1	.021
Lives independently vs lives with children or other people	-.396	.5938	-1.560	.768	.445	1	.505
Barthel score post-lockdown	-.057	.0146	-.086	-.028	15.256	1	.000
(Scale)	1a						

Dependent variable: Orcasitas study survival June 2025 at 60 months (5 years)
Model: (Intersection). high-not-high risk level. high-not-high intervention level. chronicity burden. polymedicated. age. sex. education. income level. caregiver. use of mobility aid devices. marital status married. marital status widowed. lives independently vs lives with children or other people. Barthel score post-lockdown
a. Fixed at the value shown.

- Subsection B: Analysis of the incremental predictive value of the change in functional status observed after lockdown using the area under the ROC curve.

Case Process Overview

Orcasitas study: June 2025 survival at 60 months (5 years)	N valid (according to list)
Positive	47
Negative	75
Lost	5

Lower values in the contrast outcome variable indicate greater evidence of a positive real state.

a. The positive real state is alive.

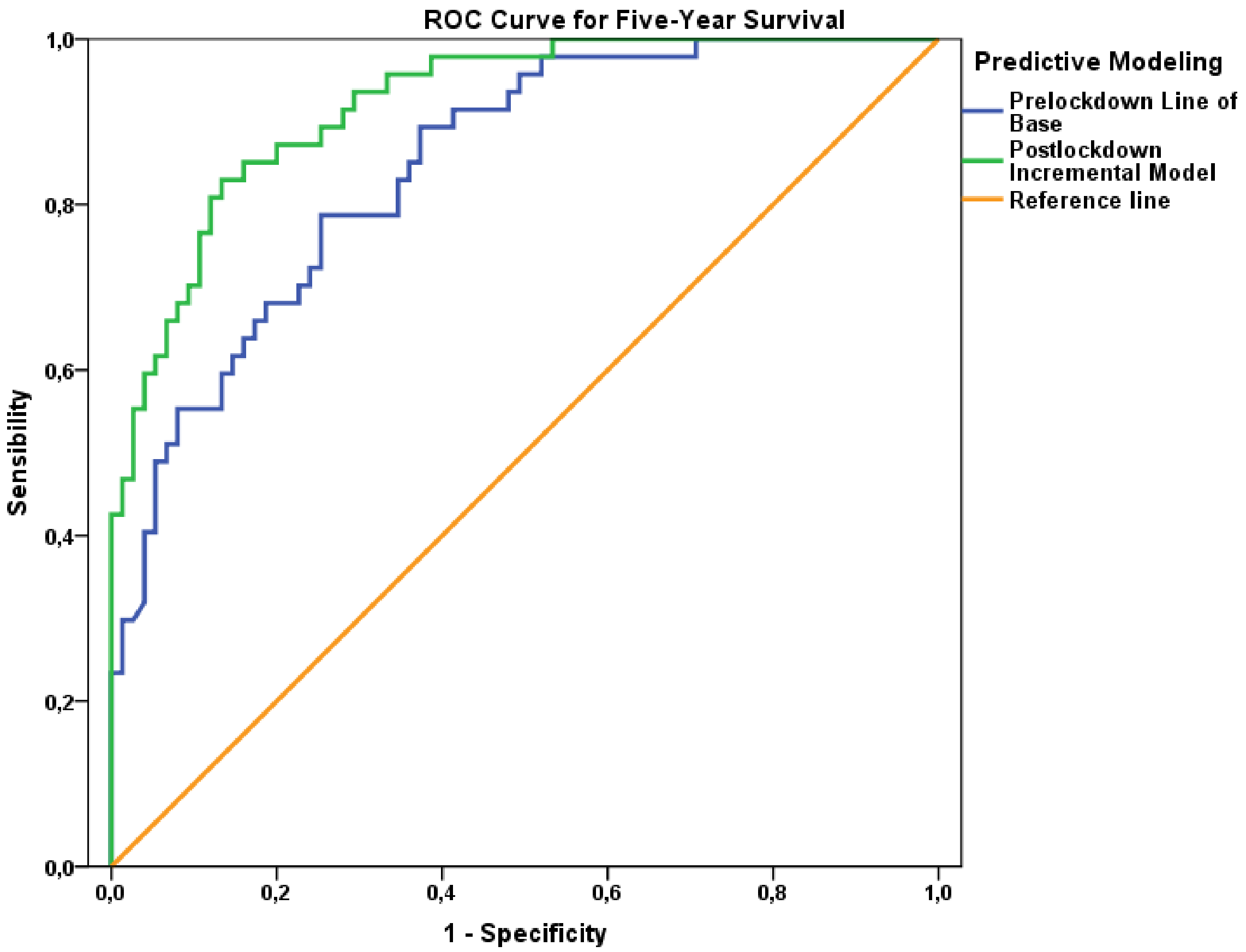

Area under the curve

Contrast result variables	Area	Std. Error[a]	Asymptotic sig.[b]	95% asymptotic confidence interval	
				Lower limit	Upper limit
Pre-lockdown baseline predicted probability	.846	.034	.000	.778	.913
Predicted probability incremental model 2-3	.921	.023	.000	.876	.967

The contrasting outcome variable(s): Predicted Probability pre-lockdown baseline has at least one tie between the positive real-state group and the negative real-state group. Statistics may be biased.

a. Under the non-parametric assumption

b. Null hypothesis: true area = 0.5

Coordinates of the curve

Contrast result variables	Positive if it is less than or equal[a]	Sensibility	1 - Specificity
Pre-lockdown baseline predicted probability	.0000000	.000	.000
	.0453790	.021	.000
	.0692318	.043	.000
	.0788485	.064	.000
	.0837048	.085	.000
	.0943882	.106	.000
	.1053280	.128	.000
	.1204782	.149	.000
	.1344959	.170	.000
	.1388969	.191	.000
	.1417993	.213	.000
	.1438380	.234	.000
	.1638458	.234	.013
	.1897412	.255	.013
	.2084047	.277	.013
	.2253052	.298	.013
	.2311952	.298	.027
	.2317456	.319	.040
	.2508699	.340	.040
	.2765237	.362	.040
	.2960675	.383	.040
	.3113142	.404	.040
	.3201187	.404	.053
	.3292806	.426	.053
	.3400278	.447	.053
	.3559479	.468	.053
	.3771204	.489	.053
	.3921161	.489	.067
	.3939836	.511	.067
	.4021899	.511	.080
	.4138615	.532	.080
	.4178556	.553	.080
	.4195238	.553	.093
	.4211714	.553	.107
	.4238307	.553	.120
	.4273012	.553	.133

	.4308165	.574	.133
	.4356109	.596	.133
	.4409253	.596	.147
	.4484371	.617	.147
	.4545394	.617	.160
	.4627490	.638	.160
	.4731432	.638	.173
	.4825969	.660	.173
	.4964521	.660	.187
	.5059566	.681	.187
	.5117967	.681	.200
	.5167963	.681	.213
	.5186476	.681	.227
	.5247518	.702	.227
	.5305442	.702	.240
	.5420027	.723	.240
	.5565548	.723	.253
	.5688142	.745	.253
	.5819816	.766	.253
	.5945510	.787	.253
	.6141670	.787	.267
	.6278922	.787	.280
	.6311585	.787	.293
	.6378192	.787	.307
	.6497378	.787	.320
	.6572168	.787	.333
	.6586720	.787	.347
	.6599718	.809	.347
	.6675787	.830	.347
	.6831835	.830	.360
	.6926030	.851	.360
	.6937361	.851	.373
	.7000745	.872	.373
	.7067261	.894	.373
	.7230583	.894	.387
	.7427718	.894	.400
	.7504220	.894	.413
	.7547782	.915	.413

	.7604071	.915	.427
	.7653735	.915	.440
	.7684901	.915	.453
	.7773435	.915	.467
	.7872199	.915	.480
	.8007392	.936	.480
	.8121092	.936	.493
	.8191643	.957	.493
	.8266443	.957	.507
	.8289518	.957	.520
	.8335979	.979	.520
	.8398125	.979	.533
	.8445851	.979	.547
	.8554458	.979	.560
	.8643173	.979	.573
	.8703094	.979	.587
	.8866574	.979	.600
	.8996609	.979	.613
	.9108793	.979	.627
	.9215874	.979	.640
	.9254320	.979	.653
	.9301395	.979	.667
	.9321406	.979	.680
	.9361941	.979	.693
	.9426004	.979	.707
	.9508743	1.000	.707
	.9573492	1.000	.720
	.9603652	1.000	.733
	.9647565	1.000	.747
	.9672474	1.000	.760
	.9721594	1.000	.773
	.9774496	1.000	.787
	.9794586	1.000	.800
	.9817096	1.000	.813
	.9828374	1.000	.827
	.9835285	1.000	.840
	.9854922	1.000	.853
	.9878704	1.000	.867

	.9890480	1.000	.880
	.9899681	1.000	.893
	.9913210	1.000	.907
	.9936374	1.000	.920
	.9969158	1.000	.933
	.9985216	1.000	.947
	.9990774	1.000	.960
	.9997420	1.000	.973
	.9998611	1.000	.987
	1.0000000	1.000	1.000
Predicted probability incremental model 2-3	.0000000	.000	.000
	.0000000	.021	.000
	.0000000	.043	.000
	.0015284	.064	.000
	.0047783	.085	.000
	.0088132	.106	.000
	.0124637	.128	.000
	.0163447	.149	.000
	.0192123	.170	.000
	.0296812	.191	.000
	.0425896	.213	.000
	.0529706	.234	.000
	.0626845	.255	.000
	.0656302	.277	.000
	.0708746	.298	.000
	.0923167	.319	.000
	.1226252	.340	.000
	.1445268	.362	.000
	.1559097	.383	.000
	.1617142	.404	.000
	.1678064	.426	.000
	.1779982	.426	.013
	.1941798	.447	.013
	.2066610	.468	.013
	.2109271	.468	.027
	.2132153	.489	.027
	.2175438	.511	.027
	.2263705	.532	.027

	.2483849	.553	.027
	.2756054	.553	.040
	.2900114	.574	.040
	.3004483	.596	.040
	.3138082	.596	.053
	.3215753	.617	.053
	.3382124	.617	.067
	.3580822	.638	.067
	.3637564	.660	.067
	.3655431	.660	.080
	.3691538	.681	.080
	.3799088	.681	.093
	.3902514	.702	.093
	.3989170	.702	.107
	.4130816	.723	.107
	.4298582	.745	.107
	.4426464	.766	.107
	.4603591	.766	.120
	.4878363	.787	.120
	.5056912	.809	.120
	.5205621	.809	.133
	.5349135	.830	.133
	.5482329	.830	.147
	.5579018	.830	.160
	.5597387	.851	.160
	.5635391	.851	.173
	.5727180	.851	.187
	.5931427	.851	.200
	.6245100	.872	.200
	.6579758	.872	.213
	.6735625	.872	.227
	.6774681	.872	.240
	.6856903	.872	.253
	.6943416	.894	.253
	.7037514	.894	.267
	.7100115	.894	.280
	.7286462	.915	.280
	.7584148	.915	.293

	.7736048	.936	.293
	.7772313	.936	.307
	.7804156	.936	.320
	.7893839	.936	.333
	.7977665	.957	.333
	.8215668	.957	.347
	.8442322	.957	.360
	.8453792	.957	.373
	.8496297	.957	.387
	.8555618	.979	.387
	.8698256	.979	.400
	.8859531	.979	.413
	.8974635	.979	.427
	.9055330	.979	.440
	.9066140	.979	.453
	.9145024	.979	.467
	.9228938	.979	.480
	.9309020	.979	.493
	.9443870	.979	.507
	.9538764	.979	.520
	.9568505	.979	.533
	.9582484	1.000	.533
	.9650530	1.000	.547
	.9750065	1.000	.560
	.9794617	1.000	.573
	.9821620	1.000	.587
	.9849405	1.000	.600
	.9853844	1.000	.613
	.9858704	1.000	.627
	.9896595	1.000	.640
	.9937683	1.000	.653
	.9946815	1.000	.667
	.9955649	1.000	.680
	.9968931	1.000	.693
	.9978498	1.000	.707
	.9981052	1.000	.720
	.9983343	1.000	.733
	.9985011	1.000	.747

	.9993240	1.000	.760
	1.0000000	1.000	.773
	1.0000000	1.000	.787
	1.0000000	1.000	.800
	1.0000000	1.000	.813
	1.0000000	1.000	.827
	1.0000000	1.000	.840
	1.0000000	1.000	.853
	1.0000000	1.000	.867
	1.0000000	1.000	.880
	1.0000000	1.000	.893
	1.0000000	1.000	.907
	1.0000000	1.000	.920
	1.0000000	1.000	.933
	1.0000000	1.000	.947
	1.0000000	1.000	.960
	1.0000000	1.000	.973
	1.0000000	1.000	.987
	1.0000000	1.000	1.000

The contrasting outcome variable(s): Predicted Probability pre-lockdown baseline has at least one tie between the positive real-state group and the real-state negative group.

a. The lowest cut-off value is the minimum observed contrast value minus 1. while the highest cut-off value is the maximum observed contrast value plus 1. All other cut-off values are the average of two ordered and consecutive observed contrast values.
