# Supplementary material for: Functional status following the COVID-19 lockdown and five-year survival among older adults with pre-existing functional dependence: a cohort study
Source: Front Aging. 2026 Oct 22;7:1868924. doi: 10.3389/fragi.2026.1868924 (PMC13736780; doi:10.3389/fragi.2026.1868924)
Supplement: Supplementary file 5 [file DataSheet5.pdf]

Appendix 5.

Module 1: Data Behind the Figures.

- Data behind the Figure 7: Difference in Barthel Index scores before and after home confinement due to the COVID-19 pandemic:

Difference between pre-lockdown and post-lockdown Barthel scores during the Covid-19 pandemic

		Frequency	Percentage	Valid percentage	Cumulative percentage
Valid	-40	1	.8	.8	.8
	-35	1	.8	.8	1.6
	-30	4	3.1	3.1	4.7
	-25	2	1.6	1.6	6.3
	-20	3	2.4	2.4	8.7
	-15	2	1.6	1.6	10.2
	-10	10	7.9	7.9	18.1
	-5	10	7.9	7.9	26.0
	0	15	11.8	11.8	37.8
	5	8	6.3	6.3	44.1
	10	11	8.7	8.7	52.8
	15	11	8.7	8.7	61.4
	20	9	7.1	7.1	68.5
	25	16	12.6	12.6	81.1
	30	7	5.5	5.5	86.6
	35	6	4.7	4.7	91.3
	40	3	2.4	2.4	93.7
	45	2	1.6	1.6	95.3
	50	1	.8	.8	96.1
	55	1	.8	.8	96.9
	58	1	.8	.8	97.6
	65	1	.8	.8	98.4
	70	1	.8	.8	99.2
	76	1	.8	.8	100.0
	Total	127	100.0	100.0	

- Data behind the Figure 9:

Age of individuals who improved their functional capacity while in lockdown at their usual residence due to the COVID-19 pandemic:

Age

		Frequency	Percentage	Valid percentage	Cumulative percentage
Valid	66	1	1.3	1.3	1.3
	68	1	1.3	1.3	2.5
	69	2	2.5	2.5	5.1
	71	1	1.3	1.3	6.3
	72	1	1.3	1.3	7.6
	75	1	1.3	1.3	8.9
	76	2	2.5	2.5	11.4
	79	2	2.5	2.5	13.9
	80	1	1.3	1.3	15.2
	81	4	5.1	5.1	20.3
	82	5	6.3	6.3	26.6
	83	4	5.1	5.1	31.6
	84	6	7.6	7.6	39.2
	85	6	7.6	7.6	46.8
	86	8	10.1	10.1	57.0
	87	3	3.8	3.8	60.8
	88	4	5.1	5.1	65.8
	89	5	6.3	6.3	72.2
	90	4	5.1	5.1	77.2
	91	7	8.9	8.9	86.1
	92	4	5.1	5.1	91.1
	93	1	1.3	1.3	92.4
	94	1	1.3	1.3	93.7
	95	1	1.3	1.3	94.9
	97	1	1.3	1.3	96.2
	98	1	1.3	1.3	97.5
	99	1	1.3	1.3	98.7
	102	1	1.3	1.3	100.0
	Total	79	100.0	100.0	

Age of individuals whose functional capacity worsened during lockdown at their usual residence due to the COVID-19 pandemic:

Age

		Frequency	Percentage	Valid percentage	Cumulative percentage
Valid	77	1	3.0	3.0	3.0
	80	1	3.0	3.0	6.1
	83	2	6.1	6.1	12.1
	84	1	3.0	3.0	15.2
	85	5	15.2	15.2	30.3
	86	3	9.1	9.1	39.4
	87	5	15.2	15.2	54.5
	88	2	6.1	6.1	60.6
	89	1	3.0	3.0	63.6
	90	1	3.0	3.0	66.7
	91	4	12.1	12.1	78.8
	92	1	3.0	3.0	81.8
	93	2	6.1	6.1	87.9
	95	2	6.1	6.1	93.9
	96	1	3.0	3.0	97.0
	98	1	3.0	3.0	100.0
	Total	33	100.0	100.0	

- Data behind the Figure 10:

- Age of individuals who remained functionally dependent following the lockdown due to the Covid-19 pandemic:

Age

		Frequency	Percentage	Valid percentage	Cumulative percentage
Valid	68	1	1.4	1.4	1.4
	69	2	2.8	2.8	4.2
	71	1	1.4	1.4	5.6
	72	1	1.4	1.4	6.9
	77	1	1.4	1.4	8.3
	80	1	1.4	1.4	9.7
	81	2	2.8	2.8	12.5
	82	1	1.4	1.4	13.9
	83	4	5.6	5.6	19.4
	84	3	4.2	4.2	23.6
	85	8	11.1	11.1	34.7
	86	5	6.9	6.9	41.7
	87	7	9.7	9.7	51.4
	88	3	4.2	4.2	55.6
	89	4	5.6	5.6	61.1
	90	4	5.6	5.6	66.7
	91	5	6.9	6.9	73.6
	92	4	5.6	5.6	79.2
	93	5	6.9	6.9	86.1
	94	1	1.4	1.4	87.5
	95	3	4.2	4.2	91.7
	96	1	1.4	1.4	93.1
	98	2	2.8	2.8	95.8
	99	2	2.8	2.8	98.6
	102	1	1.4	1.4	100.0
	Total	72	100.0	100.0	

- Age of individuals who. following the lockdown due to the Covid-19 pandemic. improved their functional capacity to the level of functional independence established in the protocol:

Age

		Frequency	Percentage	Valid percentage	Cumulative percentage
Valid	66	1	1.8	1.8	1.8
	75	1	1.8	1.8	3.6
	76	2	3.6	3.6	7.3
	79	2	3.6	3.6	10.9
	80	1	1.8	1.8	12.7
	81	2	3.6	3.6	16.4
	82	4	7.3	7.3	23.6
	83	3	5.5	5.5	29.1
	84	5	9.1	9.1	38.2
	85	4	7.3	7.3	45.5
	86	6	10.9	10.9	56.4
	87	3	5.5	5.5	61.8
	88	4	7.3	7.3	69.1
	89	3	5.5	5.5	74.5
	90	3	5.5	5.5	80.0
	91	6	10.9	10.9	90.9
	92	3	5.5	5.5	96.4
	95	1	1.8	1.8	98.2
	97	1	1.8	1.8	100.0
	Total	55	100.0	100.0	

Module 2: Analysis of confounding, concurrent and mediating factors.

Section 1. Analysis of confounding factors.

Table S1: Associations between the confounding factors analyzed and the changes observed in functional capacity after 100 days of lockdown in the usual residence due to the Covid-19 pandemic, in terms of improvement or worsening of the Barthel Index score compared to pre-lockdown scores. 1: Levels of dependency analyzed: Improves vs Worsens functional capacity after lockdown. 2: Education level: Had education: having completed elementary school or a higher level of education; Not education: illiterate; able to read and write but having no formal education; and not having completed elementary school.

Changes to the Barthel Index Scores After Nationwide Covid-19 Lockdown. Confounding factors				
**Social Factor**	**Status**	**Improves Functional Capacity After Lockdown[1] (n=79)**	**Worsens Functional Capacity After Lockdown[1] (n=33)**	**Odds Ratio (Confidence Interval)**
**Sex (n=112)**	**Male**	70.8% (17)	29.2% (7)	0.982 (0.364-2.648)
	**Female**	70.5% (62)	29.5% (26)	
**Educational Level[2] (n=111)**	**Not Education**	71.1% (69)	28.9% (28)	0.986 (0.285-3.406)
	**Had Education**	71.4% (10)	28.6% (4)	
**Economic Level (n=112)**	**<11.200 euros/year**	77.4% (41)	22.6% (21)	1.888 (0.819-4.353)
	**>11.200 euros/year**	64.4% (38)	35.6% (21)	
**Risk Level Healthcare System (n=112)**	**High**	68.9% (42)	31.1% (19)	0.836 (0.369-1.898)
	**Low or Medium**	72.5% (37)	27.5% (14)	
**Intervention Level Healthcare System (n=112)**	**High**	71.7% (33)	28.3% (13)	1.104 (0.482-2.529)
	**Low or Medium**	69.7% (46)	30.3% (20)	
**Burden of chronicity (n=112)**	**≥ 5 Chronic Diseases**	71% (22)	29% (9)	0.972 (0.391-2.415)
	**< 5 Chronic Diseases**	70.4% (57)	29.6% (24)	
**Drugs-Polymedicated (n=111)**	**≥ 5 Drugs**	68% (70)	32% (33)	—
	**< 5 Drugs**	100% (8)	0% (0)	

Table S2: Associations between the confounding factors analyzed and the changes observed in functional capacity after 100 days of lockdown in the usual residence due to the Covid-19 pandemic, in terms of the intensity of the functional improvement observed in the Barthel Index score. 1: Intensity of the observed functional response: Moderate: 5–20 points. : Clinically relevant: 25 to 95 points. Clinically relevant: mathematically implies an improvement that includes functional independence in at least one activity on the Barthel Index. **2: Education level: Had education**: having completed elementary school or a higher level of education; Not education: illiterate; able to read and write but having no formal education; and not having completed elementary school.

Changes to the Level of Functional Dependency During Nationwide Covid-19 Lockdown Confounding factors				
**Social Factor**	**Status**	**Intensity of Functional Response Observed After Lockdown (in Terms of Barthel Index Score)[1]**		**Odds Ratio (Confidence Interval)**
		**Moderate: 5–20 points (n=39)**	**Clinically relevant: 25–95 points (n=40)**	
**Sex (n=79)**	**Male**	41.2% (7)	58.8% (10)	1.524 (0.514-4.518)
	**Female**	51.6% (32)	48.4% (30)	
**Educational Level[2] (n=79)**	**Not Education**	46.4% (32)	53.6% (37)	0.371 (0.088-1.553)
	**Had Education**	70% (7)	30% (3)	
**Economic Level (n=79)**	**<11.200 euros/year**	48.8% (20)	51.2% (21)	0.952 (0.394-2.303)
	**>11.200 euros/year**	50% (19)	50% (19)	
**Risk Level Healthcare System (n=79)**	**High**	40.5% (17)	59.5% (25)	0.464 (0.188-1.141)
	**Low or Medium**	59.5% (22)	40.5% (15)	
**Intervention Level Healthcare System (n=79)**	**High**	48.5% (16)	51.5% (17)	0.941 (0.385-2.302)
	**Low or Medium**	50% (23)	50% (23)	
**Burden of chronicity (n=79)**	**≥ 5 Chronic Diseases**	50% (11)	50% (11)	0.966 (0.361-2.583)
	**< 5 Chronic Diseases**	49.1% (28)	50.9% (29)	
**Drugs-Polymedicated (n=78)**	**≥ 5 Drugs**	48.6% (34)	51.4% (36)	0.567 (0.126-2.555)
	**< 5 Drugs**	62.5% (5)	37.5% (3)	

Table S3: Orcasitas Study: Descriptive statistics for pre-lockdown Barthel scores regarding the outcomes "improvement or worsening in Barthel score after the Covid-19 pandemic lockdown."

Descriptive Statistics

	Orcasitas Study: Descriptive statistics for pre-lockdown Barthel scores regarding the outcomes "improvement or worsening in Barthel score after the Covid-19 pandemic lockdown."			Statistical	Standard error
Body Mass Index	Improvement	Mean		28,1054	,53008
		95% confidence interval for the mean	Lower limit	27,0499	
			Upper limit	29,1609	
		Mean trimmed at the 5%		28,1598	
		Median		28,9050	
		Variance		21,917	
		Standard deviation		4,68156	
		Minimum		15,06	
		Maximum		42,67	
		Range		27,61	
		Interquartile range		6,19	
		Skewness		-,278	,272
		Kurtosis		1,152	,538
	Worsening	Mean		29,4150	,87143
		95% confidence interval for the mean	Lower limit	27,6377	
			Upper limit	31,1923	
		Mean trimmed at the 5%		29,2764	
		Median		28,8450	
		Variance		24,301	
		Standard deviation		4,92956	
		Minimum		21,09	
		Maximum		41,42	
		Range		20,33	
		Interquartile range		6,78	
		Skewness		,322	,414
		Kurtosis		-,169	,809

Table S4: Orcasitas Study: Descriptive statistics for "Intensity of improvement in functional capacity to perform basic activities of daily living following the Covid-19 pandemic lockdown." The degree of improvement was classified into two levels. according to the criteria for clinical significance outlined in the Materials and Methods section: 1: improvement of 5 to 20 points; 2: improvement of 25 to 95 points. A score of 95 points represents the maximum possible level of improvement on a scale of 0 to 100 with 5-point intervals."

Descriptive Statistics

	Orcasitas Study: Descriptive statistics for "Intensity of improvement in functional capacity to perform basic activities of daily living following the Covid-19 pandemic lockdown."			Statistical	Standard error
Body Mass Index	Improves their Barthel Index score by 5 to 20 points	Mean		28,2777	,72906
		95% confidence interval for the mean	Lower limit	26,8018	
			Upper limit	29,7536	
		Mean trimmed at the 5%		28,5120	
		Median		29,0000	
		Variance		20,729	
		Standard deviation		4,55295	
		Minimum		15,06	
		Maximum		37,01	
		Range		21,95	
		Interquartile range		6,34	
		Skewness		-,969	,378
		Kurtosis		,887	,741
	Improves their Barthel Index score by 25 to 95 points	Mean		27,9331	,77824
		95% confidence interval for the mean	Lower limit	26,3576	
			Upper limit	29,5085	
		Mean trimmed at the 5%		27,8485	
		Median		28,1500	
		Variance		23,621	
		Standard deviation		4,86009	
		Minimum		15,77	
		Maximum		42,67	
		Range		26,90	
		Interquartile range		5,36	
		Skewness		,293	,378
		Kurtosis		1,790	,741

Table S5: Orcasitas Study: Descriptive statistics for "Remains functionally dependent after lockdown due to the Covid-19 lockdown (Barthel score of 60 or lower) versus no longer meeting the criteria for functional dependence established in the protocol and achieving independence (Barthel score >60)."

Descriptive Statistics

	Orcasitas Study: Descriptive statistics for "After lockdown due to the Covid-19 pandemic. the individual remains functionally dependent or has a functional capacity score above 60 on the Barthel Index."			Statistical	Standard error
Body Mass Index	Barthel >60 after lockdown	Mean		28,6863	,62200
		95% confidence interval for the mean	Lower limit	27,4454	
			Upper limit	29,9271	
		Mean trimmed at the 5%		28,7440	
		Median		29,0000	
		Variance		27,082	
		Standard deviation		5,20400	
		Minimum		15,06	
		Maximum		41,42	
		Range		26,36	
		Interquartile range		6,93	
		Skewness		-,191	,287
		Kurtosis		,162	,566
	Remains functionally dependent	Mean		28,4520	,54145
		95% confidence interval for the mean	Lower limit	27,3665	
			Upper limit	29,5375	
		Mean trimmed at the 5%		28,4367	
		Median		28,9700	
		Variance		16,124	
		Standard deviation		4,01551	
		Minimum		19,29	
		Maximum		42,67	
		Range		23,38	
		Interquartile range		5,50	
		Skewness		,241	,322
		Kurtosis		2,162	,634

Section 2. Analysis of Concurrent and Mediating Factors.

2.1. Marital status: married.

In this study, being married—as opposed to other marital statuses—was not associated with five-year survival (χ^2= 1.362; p= 0.243). Being married was also not an independent risk factor for mortality (HR 0.850; CI 0.513–1.407; p=0.527). Being married did not influence whether functional capacity improved or worsened (χ^2= 0.855; p= 0.355) (Table S6), the intensity of the functional improvement observed in terms of Barthel Index scores (χ^2 = 0.006; p= 0.941) (Table S7), or on whether that improvement allowed patients to achieve the level of functional independence specified in the protocol (χ^2= 0.119; p= 0.730) (Table S8).

Table S6: Association between the concurrent and mediating factors analyzed and the changes observed in functional capacity after 100 days of lockdown in the usual residence due to the Covid-19 pandemic, in terms of improvement or worsening of the Barthel Index score compared to pre-lockdown scores. 1: Levels of dependency analyzed: Remains functionally dependent after the lockdown: Barthel score of 60 or lower. Achieves functional independence after the lockdown according to the protocol: Barthel score >60.

Changes in Barthel Index Scores After Nationwide Covid-19 Lockdown I<br>Functional capacity improves or worsens				
**Social Factor**	**Status**	**Improves Functional Capacity After Lockdown[1] (n=79)**	**Worsens Functional Capacity After Lockdown[1] (n=33)**	**Odds Ratio (Confidence Interval)**
**Marital Status (n=112)**	**Married**	64.9% (24)	35.1% (13)	0.671 (0.288-1.566)
	**Not Married**	73.3% (55)	26.7% (20)	
**Marital Status (n=112)**	**Widowed**	74.6% (53)	25.4% (18)	1.699 (0.740-3.898)
	**Not Widowed**	63.4% (26)	36.6% (15)	
**Housing Situation (n=112)**	**Lives Independently**	83.7% (36)	16.3% (7)	**3.110 (1.209-7.999)**
	**Lives with Children/Others**	62.3% (43)	37.7% (26)	
**Community Living (n=112)**	**Leaves Home**	75.3% (55)	24.7% (18)	1.910 (0.827-4,407)
	**Homebound**	61.5% (24)	38.5% (15)	
**Leaves Home for Walks or Leisure (n=107)**	**Yes**	79.4% (50)	20.6% (13)	2.198 (0.925-5.224)
	**No**	63.6% (28)	36.4% (16)	
**Shopping Independently (n=109)**	**Yes**	100% (17)	0% (0)	—
	**No**	67.4% (62)	32.6% (30)	
**Shops Under Supervision (n=108)**	**Yes**	93.8% (15)	6.3% (1)	6.563 (0.826-52.131)
	**No**	69.6% (64)	30.4% (28)	

Table S7: Association between the concurrent and mediating factors analyzed and the changes observed in functional capacity after 100 days of lockdown in the usual residence due to the Covid-19 pandemic, in terms of the intensity of the functional response as assessed by the Barthel Index. 1: Intensity of the observed functional response: Moderate: 5–20 points. : Clinically relevant: 25 to 95 points. Clinically relevant: mathematically implies an improvement that includes functional independence in at least one activity on the Barthel Index.

**Changes in Barthel Index Score After Nationwide Covid-19 Lockdown II**				
**Social Factor**	**Status**	**Intensity of Functional Response Observed After Lockdown (in Terms of Barthel Index Score)[1]**		**Odds Ratio (Confidence Interval)**
		**Moderate: 5–20 points (n=39)**	**Clinically relevant: 25–95 points (n=40)**	
**Marital Status (n=79)**	**Married**	50% (12)	50% (12)	1.037 (0.397-2.706)
	**Not Married**	49.1% (27)	50.9% (28)	
**Marital Status (n=79)**	**Widowed**	47.2% (25)	52.8% (28)	0.765 (0.299-1.961)
	**Not Widowed**	53.8% (14)	46.2% (12)	
**Housing Situation (n=79)**	**Lives Independently**	58.3% (21)	41.7% (15)	1.944 (0.792-4.722)
	**Lives with Children/Others**	41.9% (18)	58.1% (25)	
**Community Living (n=79)**	**Leaves Home**	47.3% (26)	52.7% (29)	0.759 (0.290-1.985)
	**Homebound**	54.2% (13)	45.8% (11)	
**Leaves Home for Walks or Leisure (n=78)**	**Yes**	46% (23)	54% (27)	0.738 (0.292-1.867)
	**No**	53.6% (15)	46.4% (13)	
**Shopping Independently (n=79)**	**Yes**	35.3% (6)	64.7% (11)	0.479 (0.159-1.459)
	**No**	53.2% (33)	46.8% (29)	
**Shops Under Supervision (n=79)**	**Yes**	26.7% (4)	73.3% (11)	0.301 (0.087-1.047)
	**No**	54.7% (35)	45.3% (29)	

Table S8: Association between the concurrent and mediating factors analyzed and the changes observed in functional capacity following 100 days of lockdown in the usual residence due to the Covid-19 pandemic, in terms of the level of functional dependence reached after the lockdown, as assessed using the Barthel Index, or functional independence according to the protocol. 1: Levels of dependency analyzed: Remains functionally dependent after the lockdown: Barthel score of 60 or lower. Achieves functional independence after the lockdown according to the protocol: Barthel score >60. 2: Education level: Had education: having completed elementary school or a higher level of education; Not education: illiterate; able to read and write but having no formal education; and not having completed elementary school.

**Changes to the Level of Functional Dependency After Nationwide Covid-19 Lockdown**				
**Social Factor**	**Status**	**Remains Functionally Dependent After the Lockdown[1]**	**Achieves Functional Independence After the Lockdown[1]**	**Odds Ratio (Confidence Interval)**
**Marital Status Married**	**Married**	59% (23)	41% (16)	1.144 (0.533-2.457)
	**Not Married**	55.7% (49)	44.3% (39)	
**Marital Status Widowed**	**Widowed**	53.7% (44)	46.3% (38)	0.703 (0.335-1.477)
	**Not Widowed**	62.2% (28)	37.8% (17)	
**Housing Situation**	**Lives Independently**	40.4% (19)	59.6% (28)	**0.346 (0.164-0-728)**
	**Lives with Children/Others**	66.3% (53)	33.8% (27)	
**Community Living**	**Leaves Home**	50% (41)	50% (41)	**0.452 (0.210-0.971)**
	**Homebound**	68.9% (31)	31.1% (14)	
**Leaves Home for Walks or Leisure**	**Yes**	47.2% (34)	52.8% (38)	**0.434 (0.204-0.923)**
	**No**	67.3% (33)	32.7% (16)	
**Shopping Independently**	**Yes**	15.8% (3)	84.2% (16)	**0.113 (0.031-0.411)**
	**No**	62.5% (65)	37.5% (39)	
**Shops Under Supervision**	**Yes**	29.4% (5)	70.6% (12)	**0.289 (0.095-0.880)**
	**No**	59% (62)	41% (43)	

2.2. Marital status: widowed.

In this study, the social status of being widowed, compared with other marital statuses, was not associated with five-year survival (χ^2= 3.124; p= 0.077). Being widowed was also not an independent risk factor for mortality (HR 1.281; CI 0.782–2.1007; p= 0.326). Being a widower did not influence whether functional capacity improved or worsened (χ^2= 1.578; p= 0.209) (Table S6), the extent of the functional improvement observed in terms of Barthel Index scores (χ^2= 0.311; p= 0.577) (Table S7), or on whether that improvement allowed patients to achieve the level of functional independence specified in the protocol (χ^2= 0.868; p= 0.352) (Table S8).

2.3. Housing situation.

The housing situation—living independently in the community, either alone or with a partner, versus living with children or with other people who are not children—was not associated in this study with five-year survival (χ^2= 3.375; p= 0.066). The housing situation were also not an independent risk factor for mortality (HR 0.718; CI 0.440–1.170; p=0.184). However, it did influence whether functional ability improved or worsened (χ^2= 5.839; p= 0.016; Fisher's exact test p= 0.019) (Table S6), with 83.7% (n=36) of those living independently (n=43) showing an improvement in functional capacity after the lockdown, compared with 62.3% (n=43) among those living with their children or with other people who were not their partners (n=69). However, living with children or with other people who were not children did not influence the intensity of the functional improvement observed in terms of Barthel Index scores (χ^2= 2.127; p= 0.145) (Table S7). But it did influence whether that improvement allowed them to achieve the level of functional independence specified in the protocol (χ^2= 8.042; p= 0.005) (Table S8). The protocol-defined level of functional independence was achieved by 59.6% (n=28) of those living independently, compared to 33.8% (n=27) of those living with their children or with other people.

People who lived with their children and remained functionally dependent after confinement had a lower probability of survival (OR 2.857; CI 1.071–7.616). 75.5% (n=40) of those who lived with their children or other people and remained functionally dependent (n=53) died during this period, compared to 51.9% (n=14) of those who became functionally independent after lockdown (n=27) (χ^2= 4.492; p= 0.034).

After these results, the type of housing situation of not living independently but with other people was broken down, analyzing living with children versus living independently. This analysis showed that living with children was associated with higher mortality (χ^2= 4.785; p= 0.029) (Table 3 of the manuscript), although in the regression analysis, when adjusted for baseline severity, this factor did not show itself to be an independent risk factor for mortality (HR 0.648; CI 0.387–1.083; p= 0.098). At the end of the five-year follow-up period, 71.9% of those living with their children had died, compared with 51.1% of those living independently in the community. Regarding changes in functional capacity observed after the lockdown, the results were similar: 83.7% (n=36) of those living independently in the community showed an improvement in functional capacity, compared with 64% (n=32) of those living with their children (χ^2= 4.574; p= 0.032; Fisher's exact test 0.037). It was also observed that among those whose functional capacity worsened, 28% (n=7) lived independently and 72% (n=18) lived with their children. Regarding the extent of functional improvement observed in terms of Barthel Index scores, the differences between living with children and living independently were not significant (χ^2= 2.125; p= 0.145; Fisher's exact test p= 0.224). However, differences based on housing situation were again significant with regard to whether the improvement in functional capacity reached the level of functional independence established in the protocol: 59.6% (n=28) of those living independently reached this functional level, whereas among those living with their children, this percentage was 35.1% (n=20) (χ= 6.215; p= 0.013; Fisher's exact test statistic 0.018). Among those who remained dependent, 66.1% (n=37) lived with their children and 33.9% (n=19) lived independently.

However, dependent individuals living with their children or other people did not exhibit a higher level of functional dependence when their functional capacity was assessed using the Barthel Index. Among those living independently in the community (n=47), 23.4% (n=11) had severe functional dependence, whereas among those living with their children or others (n=80), this percentage was 33.8% (n=27) (OR 0.600; CI 0.264–1.360). When only living with children was considered in the analysis (n=57), 35.1% (n=20) had severe dependency, and the difference in this circumstance was also not significant (OR 0.565; CI 0.238–1.345).

2.4. Community living arrangements.

Community living arrangements prior to the lockdown due to the Covid-19 pandemic, whether individuals were able to leave their homes or were homebound, were not associated with survival (χ^2= 1.642; p= 0.200) (Table 3 of the manuscript). Among those living homebound (n=45), 68.9% (n=31) died, while among those who were able to leave their homes (n=82), 57.3% (n=47) died. Community living was not found in this study to be an independent risk factor for mortality (HR 0.795; CI 0.499–1.267; p= 0.335). In this cohort, the ability to leave home or living homebound did not influence whether functional ability improved or worsened after lockdown (χ^2= 2.331; p= 0.127) (Table S6), nor on the magnitude of functional improvement as measured by scores (χ^2= 0.318; p= 0.573) (Table S7), but it did influence whether this improvement reached the level of functional independence established in the protocol (χ^2= 4.222; p= 0.040) (Table S8). Of those who achieved this level of functional independence as defined by the protocol, 74.5% (n=41) were individuals who, prior to the lockdown, were able to leave their homes, compared to 25.5% (n=14) of those who remained homebound.

The people in this cohort who were able to leave their homes before the lockdown and after the lockdown achieved functional independence according to the protocol had a higher probability of survival than those who, despite having this same ability, remained functionally dependent after the lockdown (OR 3.850; CI 1.520–9.750). Among those who remained functionally dependent after the lockdown (n=41), 73.2% (n=30) died, while among those who achieved functional independence (n=41), 41.5% (n=17) died.

Regarding people who were homebound, 74.2% (n=23) of those who remained functionally dependent (n=31) and 57.1% (n=8) of those who regained their independence (n=14) after lockdown died (OR 0.464; CI 0.123-1.753).

Among those who left their homes alone, without needing to be accompanied by others (n=16), 93.8% (n=15) improved their functional capacity during confinement to the point of achieving functional independence. Of these, 73.3% (n=11) were still alive after five years. Being able to leave home alone, without an accompanying person, was associated with a higher probability of survival compared to those who lived homebound (Table 3 of the manuscript), a group in which 68.9% (n=31) died (OR 4.557; CI 1.325-15.664).

Related to the level of dependency, prior to the lockdown, 47.4% (n=18) of individuals with severe dependency were able to leave their homes, compared to 71.9% (n=64) of those with moderate dependency (χ^2= 7.011; p= 0.008).

2.5. Ability to leave home to go for a walk or engage in leisure activities.

In addition to being able to leave home for essential activities, people could also do so to go for a walk or engage in leisure activities. When this latter factor was analyzed, having this ability before the lockdown was not associated with survival (χ^2= 2.348; p= 0.125) (Table 3 of the manuscript). Among those who retained this ability (n=72), 55.6% (n=40) died, while among those who did not retain it (n=49), 69.4% (n=34) died. In this study, having this ability was not shown to be an independent risk factor for mortality (HR 0.843; CI 0.522–1.361; p= 0.484). In this cohort, having the ability to leave home to go for a walk or engage in leisure activities did not influence whether functional ability improved or worsened after lockdown (χ^2= 3.244; p= 0.072) (Table S6), nor on the magnitude of functional improvement as measured by the Barthel Index score (χ^2= 0.412; p= 0.521) (Table S7), but it did influence whether this improvement reached the level of functional independence established in the protocol (χ^2= 4.779; p= 0.029) (Table S8). Of those who achieved this level of functional independence as defined by the protocol, 70.4% (n=38) were individuals who, prior to the lockdown, had maintained the ability to leave home to go for walks or engage in leisure activities, compared with 29.6% (n=16) among those who had not maintained this ability.

People who were able to leave their homes for walks or leisure activities and managed to improve their functional capacity to the point of becoming independent during lockdown showed a higher probability of survival than those who remained functionally dependent after lockdown (OR 0.303; CI 0.114-0.807). Of those who remained dependent (n=34), 70.6% (n=24) died, compared to 42.1% (n=16) of those who became independent (n=38).

Among those who left home to go for a walk or engage in leisure activities, 80.6% (n=58) had moderate dependence and 19.4% (n=14) had severe dependence (χ^2= 9.038; p= 0.003). Depending on how they engaged in this activity, 87.5% (n=14) of those who went out independently and 75% (n=51) of those who went out accompanied by another person had moderate dependence (χ^2= 7.274; p= 0.026).

2.6. Ability to shop independently.

Among the instrumental activities assessed, having the ability to shop independently prior to lockdown was associated with survival (χ^2= 8.686; p= 0.003; Fisher's exact test= 0.005) (Table 3 of the manuscript). Among those who could independently make the purchases necessary for their daily livelihood (n=19), 31.6%

(n=6) died, while among those who could not (n=104), 67.3% (n=70) died. In this study, having this ability was shown to be an independent risk factor for mortality (HR 0.360; CI 0.156–0.830; p= 0.017). 89.5% (n=17) of the individuals in this cohort who, prior to the lockdown, were able to independently purchase what they needed improved their functional capacity after the lockdown, while among those who lacked this ability, 59.6% (n=62) improved (χ^2= 7.649; p= 0.006) (Table S6). However, the lockdown did not influence the intensity of functional improvement as measured by the Barthel Index score (χ^2= 1.816; p= 0.190) (Table S7), but it did influence whether this improvement reached the level of functional independence established in the protocol (χ^2= 14.179; p< 0.001; Fisher's exact test <0.001) (Table S8). 84.2% (n=16) of the participants who were able to shop independently reached the level of functional independence specified in the protocol, compared to 37.5% (n=39) among those who did not possess this ability.

With regard to the level of functional dependence, 78.9% (n=15) of the individuals who shopped independently had a moderate level of dependence, while among those who did not independently purchase the items necessary for their daily needs, 68.3% (n=71) had this level of dependence. These percentages were 21.1% (n=4) and 31.7% (n=33), respectively, among those with severe dependency (χ^2= 0.871; p=0.351; Fisher's exact test 0.425).

2.7. Ability to shop under supervision.

Shopping under the supervision of another person—another of the instrumental activities assessed—was not associated with survival (χ^2= 3.437; p= 0.064; Fisher's exact test statistic= 0.105) (Table 3 of the manuscript). Among those who shopped under the supervision of another person (n=17), 41.2% (n=7) died, while among those who did not shop accompanied by another person (n=105), 64.8% (n=68) died. In this study, this shopping arrangement did not prove to be an independent risk factor for mortality (HR 0.500; CI 0.228–1.094; p= 0.083). Although 93.8% (n=15) of those who shopped with another person before the lockdown improved their functional capacity after the lockdown, compared with 69.6% (n=64) among those who did not have this option, this factor did not influence the outcome of whether functional capacity improved or worsened after the lockdown (χ^2 = 4.059; p=0.044; Fisher's exact test statistic= 0.064) (Table S6) . Nor did it influence the degree of functional improvement as shown by the Barthel Index score (χ^2= 3.817; p=0.051; Fisher's exact test statistic= 0.083) (Table S7). However, it did influence whether this improvement reached the level of functional independence established in the protocol (χ^2= 5.190; p= 0.023; Fisher's exact test statistic= 0.034)

(Table S8). 70.6% (n=12) of the participants who were able to shop under the supervision of another person achieved the level of functional independence specified in the protocol, compared to 41% (n=43) among those who did not have this shopping arrangement.

Among those who were able to shop under the supervision of another person, 70.6% (n=12) had moderate dependence and 29.4% (n=5) had severe dependence. These percentages were 70.5% (n=74) and 29.5% (n=31), respectively, among those who did not shop in this manner; the differences were not statistically significant.

2.8. Presence of non-family caregivers.

The presence of non-family caregivers could indicate greater functional decline. Therefore, this factor was analyzed, and it was found that its presence was not associated with survival (χ^2= 0.066; p= 0.797) (Table S9). Among those who had some type of personal assistant for household chores or personal care, or a live-in caregiver (n=88), 62.5% (n=55) died, while among those who did not have any type of caregiver (n=35), 60% (n=21) died. In this study, having a non-family caregiver was not found to be an independent risk factor for mortality (HR 0.941; CI 0.567–1.563; p= 0.815). This factor also did not influence the outcome of whether functional capacity had improved or worsened after lockdown (χ^2= 0.096; p= 0.757) (Table S10), the degree of functional improvement as assessed by the Barthel Index score (χ^2= 2.667; p= 0.102) (Table S11), or whether this improvement reached the level of functional independence established in the protocol (χ^2= 0.364; p= 0.564). Although, overall, having a non-family caregiver was not associated with achieving this functional independence, when each type of caregiver was analyzed separately, it was observed that, in the case of having a private caregiver, having this type of caregiver was associated with remaining dependent after the lockdown (χ^2= 8.875; p= 0.003). This result was not observed when the participant had a public caregiver (χ^2= 0.525; p= 0.469) or a live-in caregiver (χ^2= 3.682; p= 0.055; Fischer's exact test p-value 0.067) (Table S12).

Table S9: Impact on latent intrinsic capacity and survival of the presence of caregivers in the home and use of mobility assistance devices.

Impact on Latent Intrinsic Capacity and Survival of the Presence of Caregivers in the Home and Use of Mobility Assistance Devices				
**Assistance Factor**		**Survival**		**Statistical test**
**Remains Dependent After Lockdown**		**Alive**	**Deceased**	**Odds ratio (Confidence interval**
**Public Assistant**	**Yes**	23.7% (9)	76.3% (29)	0.745 (0.261-2.129)
	**No**	29.4% (10)	70.6% (24)	
**Private Caregiver**	**Yes**	29.4% (10)	70.6% (24)	1.343 (0.470-3.838)
	**No**	23.7% (9)	76.3% (29)	
**Internal Caregiver**	**Yes**	27.8% (5)	72.2% (13)	0.371 (0.117-1.177)
	**No**	25.9% (14)	74.1% (14)	
**Wheelchair**	**Yes**	16.1% (5)	83,9% (26)	0.371 (0.117-1.177)
	**No**	34.1% (14)	65.9% (27)	
**Walker**	**Yes**	25.9% (7)	74.1% (20)	0.963 (0.325-2.849)
	**No**	26.7% (12)	73.3% (33)	
**Crutches-Cane**	**Yes**	36.4% (8)	63.6% (14)	2.026 (0.677-6.065)
	**No**	22% (11)	78% (39)	
**Becoming Independent After Lockdown**	**Level**	**Alive**	**Deceased**	**Odds ratio (Confidence interval**
**Public Assistant**	**Yes**	56.3% (18)	43.8% (14)	1.071 (0.360-3.193)
	**No**	54.5% (12)	45.5% (10)	
**Private Caregiver**	**Yes**	54.5% (6)	45.5% (5)	0.939 (0.247-3.578)
	**No**	56.1% (23)	43.9% (18)	
**Internal Caregiver**	**Yes**	50% (3)	50% (3)	0,808 (0.147-4.423)
	**No**	55.3% (26)	44.7% (21)	
**Wheelchair**	**Yes**	60% (6)	40% (4)	1.313 (0.326-5.291)
	**No**	53.3% (24)	46.7% (21)	
**Walker**	**Yes**	61.1% (11)	38.9% (7)	1.489 (0.473-4.683)
	**No**	51.4% (19)	48.6% (18)	
**Crutches-Cane**	**Yes**	40.6% (13)	59.4% (19)	**0.241 (0,075-0.776)**
	**No**	73.9% (17)	26.1% (6)	

Table S10: Analysis of the associations between the type of non-family caregiver for the dependent person and the type of mobility aid used, with the presence, after the Covid-19 pandemic lockdown, of an improvement or worsening in functional capacity, compared to pre-lockdown functional capacity as assessed by the Barthel Index.

**Home Caregivers, Mobility Assistance Devices, and Changes in Barthel Score After the Nationwide Covid-19 Lockdown I. Improvement/Worsening in Functional Capacity**				
**Factor**	**Status**	**Worsens Functional Capacity During Lockdown (n=33)**	**Improves Functional Capacity During Lockdown (n=78)**	**Odds Ratio (Confidence Interval)**
**Public Assistant (n=111)**	**Yes (n=61)**	26.2% (16)	73.8% (45)	1.449 (0.640-3.280)
	**No (n=50)**	34% (17)	66% (33)	
**Private Caregiver (n=109)**	**Yes (n=34)**	41.2% (14)	58.8% (20)	0.485 (0.205-1.144)
	**No (n=75)**	25.3% (19)	74.7% (56)	
**Internal Caregiver (n=110)**	**Yes (n=18)**	38.9% (7)	61.1% (11)	0.619 (0.216-1.770)
	**No (n=92)**	28.3% (26)	71.7% (66)	
**Wheelchair (n=112)**	**Yes (n=37)**	45.9% (17)	54.1% (20)	**0.319 (0.136-0.747)**
	**No (n=75)**	21.3% (16)	78.7% (59)	
**Walker (n=112)**	**Yes (n=38)**	26.3% (10)	73.7% (28)	1.263 (0.527-3.026)
	**No (n=74)**	31.1% (23)	68.9% (51)	
**Crutches-Cane (n=112)**	**Yes (n=50)**	22% (11)	78% (39)	1.950 (0.835-4.551)
	**No (n=62)**	35.5% (22)	64.5% (40)	

Table S11: Analysis of the associations between the type of non-family caregiver of the dependent person and the type of mobility aid used, and the intensity of the improvement in functional ability observed following the lockdown due to the Covid-19 pandemic. 1: Intensity of the observed functional response: Moderate: 5–20 points. : Clinically relevant: 25 to 95 points. Clinically relevant: mathematically implies an improvement that includes functional independence in at least one activity on the Barthel Index.

**Home Caregivers, Mobility Assistance Devices, and Changes in Barthel Score After the Nationwide Covid-19 Lockdown II. Intensity of Functional Response Observed After Lockdown**				
**Factor**	**Status**	**Intensity of Functional Response Observed After Lockdown (in Terms of Barthel Index Score)[1]**		**Odds Ratio (Confidence Interval)**
		**Moderate: 5–20 points (n=39)**	**Clinically relevant: 25–95 points (n=40)**	
**Public Assistant (n=78)**	**Yes (n=45)**	57.8% (26)	42.2% (19)	2.105 (0.843-5.257)
	**No (n=33)**	39.4% (13)	60.6% (20)	
**Private Caregiver (n=76)**	**Yes (n=20)**	45% (9)	55% (11)	0.818 (0.294-2.280)
	**No (n=56)**	50% (28)	50% (289	
**Internal Caregiver (n=77)**	**Yes (n=11)**	54.5% (6)	45.5% (5)	1.200 (0.333-4.321)
	**No (n=66)**	50% (33)	50% (33)	
**Wheelchair (n=79)**	**Yes (n=20)**	55% (11)	45% (9)	1.353 (0.489-3.747)
	**No (n=59)**	47.5% (28)	52.5% (31)	
**Walker (n=79)**	**Yes (n=28)**	39.3% (11)	60.7% (17)	0.532 (0.208-1.358)
	**No (n=51)**	54.9% (28)	45.1% (23)	
**Crutches-Cane (n=79)**	**Yes (n=39)**	51.3% (20)	48.7% (19)	1.163 (0.481-2.813)
	**No (n=40)**	47.5% (19)	52.5% (21)	

Table S12: Analysis of the associations between the type of non-family caregiver for the dependent person and the type of mobility aid used, in relation to the functional status observed following the lockdown due to the Covid-19 pandemic, regarding whether functional dependence persisted or whether the person achieved the functional level of independence as established in the protocol. 1: Levels of dependency analyzed: Remains functionally dependent after the lockdown: Barthel score of 60 or lower. Achieves functional independence after the lockdown according to the protocol: Barthel score >60.

Home Caregivers, Mobility Assistance Devices, and Changes in Barthel Level After the Nationwide Covid-19 Lockdown III. Remains Functional Dependence—Achieves Functional Independence				
**Factor**	**Status**	**Remains Functionally Dependent After the Lockdown[1]**	**Achieves Functional Independence After the Lockdown[1]**	**Odds Ratio (Confidence Interval)**
**Public Assistant (n=126)**	**Yes (n=70)**	54.3% (38)	45.7% (32)	0.768 (0.377-1.568)
	**No (n=56)**	60.7% (34)	39.3% (22)	
**Private Caregiver (n=124)**	**Yes (n=45)**	75.6% (34)	24.4% (11)	**3.335 (1.483-7.501)**
	**No (n=79)**	48.1% (38)	51.9% (41)	
**Internal Caregiver (n=125)**	**Yes (n=24)**	75% (18)	25% (6)	2.611 (0.957-7.121)
	**No (n=101)**	53.5% (54)	46.5% (47)	
**Wheelchair (n=127)**	**Yes (n=41)**	75.6% (31)	24.4% (10)	**3.402 (1.485-7.795)**
	**No (n=86)**	47.7% (41)	52.3% (45)	
**Walker (n=127)**	**Yes (n=45)**	60% (27)	40% (18)	1.233 (0.590-2.580)
	**No (n=82)**	54.9% (45)	45.1% (37)	
**Crutches-Cane (n=127)**	**Yes (n=54)**	40.7% (22)	59.3% (32)	**0.316 (0.152-659)**
	**No (n=73)**	68.5% (50)	31.5% (23)	

During the lockdown, restrictions on movement and social contact were imposed, which limited the availability of home care providers for dependent individuals or led to changes in their care arrangements. In this cohort, 71.5% (n=88) reported having some type of caregiver, while 28.5% (n=35) reported not having one. A single person could have different types of caregivers (Table S6 in Appendix 6). Four people did not respond. Analyzing the groups living independently or with their children, 77.8% (n=35) of those living independently in the community (n=45) had some type of personal assistant, while 58.9% (n=33) of those living with their children had this type of care assistance (χ^2= 4.030; p= 0.045; OR 0.410; CI 0.170–0.990).

Across the entire cohort, during the lockdown there was a complete suspension (37.1%; n=26) or a partial suspension (reduction in care hours, 22.8%; n=16) of the service provided by caregivers assigned by public services (n=70); this occurred in 17.8% (n=8) of cases involving private caregivers; and in one case, both a public and a private caregiver were lost. All internal caregivers continued to provide their services. Suspension of the public or private caregiver was not associated with survival (χ^2= 1.701; p= 0.637). Among those who lost their public caregiver, 38.5% (n=10) had a private caregiver, whom they retained, increasing the number of care hours provided by this caregiver in 20% (n=2) of the cases. In addition, another 38.5% (n=10) hired a new public

caregiver, meaning that the effective loss of a public caregiver during the lockdown ultimately occurred in 22.8% (n=16) of the individuals who had been assigned this support resource. Similarly, the loss of a private caregiver was replaced by the hiring of a new private caregiver in 37.5% (n=3) of cases. Finally, the individual who lost both their public and private caregivers hired new caregivers during the lockdown. In summary, during the lockdown, there was ultimately a loss of support care provided by 12.5% (n=11) of the caregivers (n=88) assigned to the individuals in this cohort.

When analyzing these results by type of non-family caregiver and housing situation, 61.1% (n=22) of those who lost their public caregiver during the lockdown were dependent individuals living independently in the community, and 38.9% (n=14) were individuals living with their children. However, 62.5% (n=5) of those who lost their private caregiver were people living with their children, compared to 6.4% (n=3) among those living independently. At the same time, 63.6% (n=14) of those living independently rehired another public caregiver, a situation that occurred in 50% (n=7) of those living with their children.

In the same context, when these two groups were compared, 87.5% (n=7) of internal caregivers and 64% (n=16) of private caregivers provided their services to people with functional dependence who lived with their children; these percentages were 12.5% (n=1) and 36% (n=9), respectively, among those who lived independently. As for public caregivers, 40% (n=24) provided care to people living with their children, and 60% (n=36) to people living independently in the community.

Regarding the number of hours per week that these public caregivers provided their services, 54.4% (n=37) worked 1 to 5 hours, 25% (n=17) worked 6 to 10 hours, and 11.8% (n=8) worked 11 to 15 hours. In the remaining 8.8% (n=6), care was provided for a greater number of hours. Two patients did not report the number of hours of care provided by the public caregiver. With regard to private caregivers, they provided supplemental care to public caregivers in 48.9% (n=22) of cases, and in 51.1% (n=23) of cases, they were the sole caregiver for the dependent person. The number of hours of care was not recorded. Internal caregivers (n=24) resided in the dependent person's home and had one and a half days off per week, during which time they were replaced by another internal caregiver or by family members.

Finally, having some type of caregiver was not associated with the level of dependency ($\chi^2 = 0.124$; $p = 0.725$) or with the Barthel Index score (Mann-Whitney $U = 1{,}318.500$; $Z = -1.265$; $p = 0.206$). 73.7% (n=28) of individuals with severe dependency had some type of caregiver, compared to 70.6% (n=60) among those with moderate

dependency. When each type of caregiver was analyzed individually, no significant differences were observed either. Among individuals with severe dependency, 57.9% (n=22) had a public caregiver, 44.7% (n=17) had a private caregiver, and 23.7% (n=9) had a live-in caregiver. These percentages were 54.5% (n=48), 32.6% (n=28), and 17.2% (n=15) among individuals with moderate dependency, respectively.

2.9. Mobility assisted by support devices.

Mobility can be assisted by aid devices, which can facilitate the performance of basic activities of daily living, or reflect the loss of mobility itself. Therefore, the influence of the use of these devices on the response observed during the Covid-19 pandemic lockdown was analyzed (Tables S3-S5 in Appendix 6).

Among the mobility aids analyzed, wheelchair use was not associated with survival (χ^2= 3.530; p= 0.060) (Table S9). In this study, the need for a wheelchair for mobility was not found to be an independent risk factor for mortality (HR 1.376; CI 0.864–2.194; p=0.179). However, wheelchair use was associated with an increased likelihood of worsened functional ability (χ^2= 7.222; p= 0.007). Among those who used a wheelchair, 45.9% (n=17) had worsened their functional capacity after the lockdown, compared to 21.3% (n=16) among those who did not use a wheelchair (Table S10). However, it did not influence the intensity of functional improvement as measured by the Barthel Index score (χ^2= 0.340; p= 0.560) (Table S11), but it did influence whether this improvement reached the level of functional independence established in the protocol (χ^2 = 8.825; p= 0.003). It was observed that 75.6% (n=31) of wheelchair users remained functionally dependent after the lockdown, compared to 47.7% (n=41) among those who did not use a wheelchair (Table S12).

With regard to the use of a walker, its use was not associated with survival (χ^2= 0.059; p= 0.808) (Table S9). In this study, the need to use a walker for mobility was not found to be an independent risk factor for mortality (HR 1.121; CI 0.700–1.795; p= 0.635). The use of crutches or a cane was not associated with the likelihood of improved functional capacity (χ^2= 0.274; p= 0.600) (Table S10), with the magnitude of the functional response observed after confinement (χ^2= 1.764; p= 0.184) (Table S11), nor with whether this improvement reached the level of functional independence established in the protocol (χ^2= 0.310; p= 0.577) (Table S12).

To complete the analysis of mobility aids, the use of crutches-cane was not associated with survival (χ^2= 0.004; p= 0.951) (Table S9). In this study, the need to use crutches-cane for mobility was not found to be an independent risk factor for mortality (HR 0.845; CI 0.530–1.350; p= 0.482). The use of crutches-cane was not associated with the probability of improved functional capacity (χ^2= 2.421; p= 0.120) (Table S10), nor with the magnitude of the functional response observed after lockdown (χ^2= 0.113; p= 0.737) (Table S11), but it did influence whether this improvement reached the level of functional independence established in the protocol (χ^2= 9.737; p= 0.002) (Table S12). Among those who used crutches-cane, 59.3% (n=32) showed an improvement in functional ability after the lockdown that allowed them to no longer be classified as functionally dependent according to the protocol, while among those who did not use these devices, this percentage was 31.5% (n=23).

With regard to the burden of chronicity, although 91.2% (n=31) of the patients with a high disease burden (n=34) used some type of mobility aid, compared with 76.3% (n=71) of those with a lower disease burden (n=93), the differences were not significant (χ^2= 3.465; p= 0.063).

On the other hand, the use of a mobility aid was not associated in this study with a higher level of functional dependence (χ^2= 0.549; p= 0.459) or a lower Barthel Index score (Mann-Whitney U= 1133.500; Z= -0.877; p= 0.380). 76.3% (n=29) of people with severe dependency used some type of mobility aid, compared to 82% (n=73) of those with moderate dependency. When each type of device was analyzed individually, 34.2% (n=13) of people with severe dependency used a walker, compared to 36% (n=32) among those with moderate dependency; the difference was not statistically significant. However, 47.4% (n=18) of those with severe dependency used a wheelchair, compared to 25.8% (n=23) among those with moderate dependency (χ^2= 5.644; p= 0.018). This relationship was reversed with regard to the use of crutches or canes (χ^2= 12.885; p< 0.001). While only 18.4% (n=7) of those with severe dependency used crutches or a cane to aid their mobility, this percentage was 52.8% (n=47) among those with moderate dependency.
