# Supplementary material for: Functional status following the COVID-19 lockdown and five-year survival among older adults with pre-existing functional dependence: a cohort study
Source: Front Aging. 2026 Oct 22;7:1868924. doi: 10.3389/fragi.2026.1868924 (PMC13736780; doi:10.3389/fragi.2026.1868924)
Supplement: Supplementary file 6 [file DataSheet6.pdf]

Appendix 6, Tables E1-E3:

Table E1: Association between the level of dependence in performing basic activities of daily living and changes observed in the Barthel index score during the 100-day nationwide Covid-19 lockdown. Comparison between pre- and post-lockdown Barthel index scores.

Mobility, Activities of Daily Living, and Latent Intrinsic Capacity. Changes in Barthel Score During Nationwide Covid-19 Lockdown				
**Basic Activities of Daily Living**	**Level of Dependence**	**Changes in the Barthel Index Score during Nationwide Covid-19 Lockdown**		
		**Worsening in Functional Capacity Score**	**Improvement in Functional Capacity Score**	**Odds Ratio (Confidence Interval)**
**Walks Independently**	**Dependent**	59.1% (13)	40.9% (9)	**0.198 (0.074-0.529)**
	**Independent**	22.2% (20)	77.8% (70)	
**Chair-to-Bed Transfers**	**Dependent**	61.4% (27)	38.6% (17)	**0.061 (0.022-0.171)**
	**Independent**	8.8% (6)	91.2% (62)	
**Up-Down Stairs**	**Dependent**	45.5% (30)	54.5% (36)	**0.084 (0.024-0.297)**
	**Independent**	6.5% (3)	93.5% (43)	
**Toilet Use**	**Dependent**	76.5% (13)	23.5% (4)	**0.082 (0.024-0.279)**
	**Independent**	21.1% (20)	78.9% (75)	
**Dressing-Undressing**	**Dependent**	69.7% (23)	30.3% (10)	**0.063 (0.023-0.171)**
	**Independent**	12.7% (10)	87.3% (69)	
**Grooming**	**Dependent**	54% (27)	46% (23)	**0.091 (0.033-0.2509**
	**Independent**	9.7% (6)	90.3% (56)	
**Bathing**	**Dependent**	39.2% (31)	60.8% (48)	**0.100 (0.022-0.447)**
	**Independent**	6.1% (2)	93.9% (31)	
**Feeding**	**Dependent**	64.5% (20)	35.5% (11)	**0.105 (0.041-0.271)**
	**Independent**	16% (13)	84% (68)	
**Bladder**	**Dependent**	71.4% (25)	28.6% (10)	**0.046 (0.016-0.131)**
	**Independent**	10.4% (8)	89.6% (69)	
**Bowels**	**Dependent**	68.4% (13)	31.6% (6)	**0.126 (0.043-0.375)**
	**Independent**	21.5% (20)	78.5% (73)	

Table E2: Association between the level of functional dependence in performing basic activities of daily living and the probability of improving overall functional capacity to achieve functional independence observed during the 100-day nationwide Covid-19 lockdown.

Mobility, Activities of Daily Living, and Latent Intrinsic Capacity				
**Basic Activities of Daily Living**	**Level of Dependence**	**Changes in the Level of Functional Dependence During the Nationwide Covid-19 Lockdown**		**Statistical Test**
		**Remains Dependent After Lockdown**	**Becoming Independent After Lockdown**	**Odds Ratio (Confidence Interval)**
**Walks Independently**	**Dependent**	88.5% (23)	11.5% (3)	**8.136 (2.297-28.822)**
	**Independent**	48.5% (49)	51.5% (52)	
**Chair-to-Bed Transfers**	**Dependent**	92.5% (49)	7.5% (4)	**27.163 (8.758-84.244)**
	**Independent**	31.1% (23)	68.9% (51)	
**Up-Down Stairs**	**Dependent**	78.5% (62)	21.5% (17)	**13.859 (5.752-33.389)**
	**Independent**	20.8% (10)	79.2% (38)	
**Toilet Use**	**Dependent**	100% (23)	0% (0)	**--**
	**Independent**	47.1% (49)	52.9% (55)	
**Dressing-Undressing**	**Dependent**	100% (40)	0% (0)	**--**
	**Independent**	36.8% (32)	63.2% (55)	
**Grooming**	**Dependent**	88.5% (54)	11.5% (7)	**20.571 (7.911-53.496)**
	**Independent**	27.3% 18)	72.7% (48)	
**Bathing**	**Dependent**	71.7% (66)	28.3% (6)	**12.269 (4.562-32.995)**
	**Independent**	17.1% (6)	82.9% (29)	
**Feeding**	**Dependent**	94.7% (36)	5.3% (2)	**26.500 (5.999-117.052)**
	**Independent**	40.4% (36)	59.6% (53)	
**Bladder**	**Dependent**	95.5% (42)	4.5% (2)	**37.100 (8.382-164.209)**
	**Independent**	36.1% (30)	63.9% (53)	
**Bowels**	**Dependent**	92.3% (24)	7.7% (2)	**13.250 (2.973-59.052)**
	**Independent**	47.5% (48)	52.5% (53)	

Table 9: Assessment of intrinsic capacity from the perspective of the ability to perform the basic activities of daily living included in the Barthel Index. Analysis of the relationships between the level of dependence in walking and the level of dependence in performing the other activities of the Barthel Index, in relation to the results observed following the lockdown in terms of improved functional ability or achieving the level of functional independence established in the protocol.

**INTRINSIC CAPACITY**<br>**Relevance of the Domain "Independence in Walking" in Functional Improvement and Recovery After Lockdown**								
**Change in Functional Capacity**			**Improves Functional Capacity After Lockdown**			**Achieve Functional Independence After Lockdown**		
**Mobility Importance Rating**	**Mobility Factor**	**Level of dependence**	**Level of Dependence for Walking**		**Odds Ratio (Confidence Interval)**	**Level of Dependence for Walking**		**Odds Ratio (Confidence Interval)**
			**Dependent**	**Independent**		**Dependent**	**Independent**	
**Mobility**	**Chair-to-Bed Transfers**	**Dependent**	55.6% (5)	17.1% (12)	**6.042**	0% (0)	7.7% (4)	—
		**Independent**	44.4% (4)	82.9% (58)	**(1.411-25.867)**	100% (3)	92.3% (48)	
	**Going Up and Down Stairs**	**Dependent**	88.9% (8)	40% (28)	**12.000**	66.7% (2)	28.8% (15)	4.933
		**Independent**	11.1% (1)	60% (42)	**(1.422-101.296)**	33.3% (1)	71.2% (37)	(0.415-58.575)
	**Toilet Use**	**Dependent**	22.2% (2)	2.9% (2)	**9.714**	0% (0)	0% (0)	—
		**Independent**	77.8% (7)	97.1% (68)	**(1.179-80.024)**	100% (3)	100% (52)	
**Mobility and Balance**	**Dressing-Undressing**	**Dependent**	33.3% (3)	10% (7)	4.500	0% (0)	0% (0)	—
		**Independent**	66.7% (6)	90% (63)	(0.917-22.083)	100% (3)	100% (52)	
	**Grooming**	**Dependent**	44.4% (4)	27.1% (19)	2.147	0% (0)	13.5% (7)	—
		**Independent**	55.6% (5)	72.9% (51)	(0.521-8.852)	100% (3)	86.5% (45)	
	**Bathing**	**Dependent**	77.8% (7)	58.6% (41)	2.476	66.7% (2)	46.2% (24)	2.333
		**Independent**	22.2% (2)	41.4% (29)	(0.479-12.786)	33.3% (1)	53.8% (28)	(0.199-27.355)
**Activities Where Mobility and Balance Are Less Important**	**Feeding**	**Dependent**	44.4% (4)	10% (7)	**7.200**	0% (0)	3.8% (2)	—
		**Independent**	55.6% (5)	90% (63)	**(1.560-33.224)**	100% (3)	96.2% (50)	
	**Bladder**	**Dependent**	44.4% (4)	8.6% (6)	**8.533**	33.3% (1)	1.9% (1)	**25.500**
		**Independent**	55.6% (5)	91.4% (64)	**(1.796-40.548)**	66.7% (2)	98.1% (51)	**(1.136-572.368)**
	**Bowels**	**Dependent**	22.2% (2)	5.7% (4)	4.714	0% (0)	3.8% (2)	—
		**Independent**	77.8% (7)	94.3% (66)	(0.728-30.516)	100% (3)	96.2% (50)	
